# Lint‐O cooperates with L(3)mbt in target gene suppression to maintain homeostasis in fly ovary and brain

**DOI:** 10.15252/embr.202153813

**Published:** 2022-08-22

**Authors:** Hitomi Yamamoto‐Matsuda, Keita Miyoshi, Mai Moritoh, Hikari Yoshitane, Yoshitaka Fukada, Kuniaki Saito, Soichiro Yamanaka, Mikiko C Siomi

**Affiliations:** ^1^ Department of Biological Sciences, Graduate School of Science The University of Tokyo Tokyo Japan; ^2^ Department of Chromosome Science National Institute of Genetics, Research Organization of Information and Systems Shizuoka Japan; ^3^ Department of Genetics School of Life Science, SOKENDAI Shizuoka Japan

**Keywords:** fertility, gene regulation, L(3)mbt, Lint‐O, piRNA, Chromatin, Transcription & Genomics, RNA Biology

## Abstract

Loss‐of‐function mutations in *Drosophila lethal(3)malignant brain tumor* [l(3)mbt] cause ectopic expression of germline genes and brain tumors. Loss of L(3)mbt function in ovarian somatic cells (OSCs) aberrantly activates germ‐specific piRNA amplification and leads to infertility. However, the underlying mechanism remains unclear. Here, ChIP‐seq for L(3)mbt in cultured OSCs and RNA‐seq before and after L(3)mbt depletion shows that L(3)mbt genomic binding is not necessarily linked to gene regulation and that L(3)mbt controls piRNA pathway genes in multiple ways. Lack of known L(3)mbt co‐repressors, such as Lint‐1, has little effect on the levels of piRNA amplifiers. Identification of L(3)mbt interactors in OSCs and subsequent analysis reveals CG2662 as a novel co‐regulator of L(3)mbt, termed “L(3)mbt interactor in OSCs” (Lint‐O). Most of the L(3)mbt‐bound piRNA amplifier genes are also bound by Lint‐O in a similar fashion. Loss of Lint‐O impacts the levels of piRNA amplifiers, similar to the lack of L(3)mbt. The *lint‐O*‐deficient flies exhibit female sterility and tumorous brains. Thus, L(3)mbt and its novel co‐suppressor Lint‐O cooperate in suppressing target genes to maintain homeostasis in the ovary and brain.

## Graphical abstract

Lint‐O, a novel L(3)mbt interactor, cooperates with L(3)mbt to suppress piRNA amplification genes and other gene targets to maintain homeostasis in *Drosophila* ovary and brain.

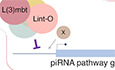

## Introduction

Temperature‐sensitive mutations introduced into the *Drosophila* tumor suppressor *lethal(3)malignant brain tumor* [*l(3)mbt*] cause malignant growth of adult optic neuroblasts and ganglion mother cells in the larval brain (Gateff *et al*, 1993; Janic *et al*, 2010). At restrictive temperatures, the mutant flies die at the larval stage, while at permissive temperatures, they are viable but infertile (Coux *et al*, 2018). The expression of L(3)mbt in follicle cells in the ovary is particularly crucial for normal oogenesis to occur.

Genome‐wide RNA sequencing (RNA‐seq) in the *l(3)mbt* tumorous brain has identified genes under the control of this tumor suppressor, collectively known as the malignant brain tumor signature (MBTS) (Janic *et al*, 2010). MBTS includes germline genes required for the production and amplification of PIWI‐interacting RNAs (piRNAs), such as one of the PIWI members *aubergine* (*aub*) and the DEAD‐box RNA helicase *vasa*. Forced attenuation of the aberrant expression of these genes in brain tumors makes the tumorous tissue return to normal (Janic *et al*, 2010). This indicates that the function of L(3)mbt in repressing piRNA factors in nongonadal somatic tissues is directly linked to tumor suppression, particularly in the brain.

The piRNAs are a subset of small RNAs enriched in the germline where they silence transposons (Iwasaki *et al*, 2015; Czech *et al*, 2018; Yamashiro & Siomi, 2018; Ozata *et al*, 2019). Upon abrogation of piRNA function, transposons under the control of piRNAs can move within the germline genome. This causes DNA damage, which leads to failure in germline development, resulting in infertility (Schüpbach & Wieschaus, 1991; Klattenhoff *et al*, 2007). Thus, piRNA‐mediated transposon silencing is indispensable for a wide range of animals to produce offspring through sexual reproduction.

In cultured ovarian somatic cells (OSCs), which correspond to follicle cells in the ovary, all piRNAs are loaded onto Piwi, one of three PIWI members, giving rise to the piRNA‐induced silencing complex (piRISC) (Saito *et al*, 2009). Piwi‐piRISC is then localized to the nucleus where it represses transposons transcriptionally by reducing the level of RNA polymerase II at the target loci and/or inducing heterochromatinization around these loci (Yin & Lin, 2007; Klenov *et al*, 2011; Shpiz *et al*, 2011; Wang & Elgin, 2011; Sienski *et al*, 2012; Yashiro *et al*, 2018; Onishi *et al*, 2020, 2021). By contrast, in ovarian germ cells (OGCs), piRNAs are loaded onto all three PIWI members: Piwi, Aub, and Argonaute3 (AGO3). Piwi‐bound piRNAs in both OSCs and OGCs are biased toward the antisense orientation of transposon mRNAs and, as in OSCs, Piwi‐piRISC in OGCs represses transposons transcriptionally by targeting nascent transcripts while they are being synthesized on the genome (Saito *et al*, 2006; Vagin *et al*, 2006; Czech *et al*, 2018). Aub‐bound piRNAs are also biased toward the antisense orientation, but Aub‐piRISC silences transposons post‐transcriptionally in the cytoplasm by cleaving transposon mRNAs, depending on the endonuclease activity that Aub exhibits (Iwasaki *et al*, 2015; Czech *et al*, 2018). AGO3 also has endonuclease activity, but AGO3‐bound piRNAs are mostly sense to transposon mRNAs; thus, AGO3 cleaves transposon transcripts in the antisense orientation. RNAs fragmented by AGO3 are subsequently consumed as precursors for Aub‐bound piRNAs. In this way, Aub‐piRISCs are amplified. Similarly, Aub‐dependent RNA cleavage amplifies AGO3‐piRISCs. Aub and AGO3 continue this chain reaction, known as the ping‐pong cycle, thereby accumulating a great number of piRISCs in the OGCs (Brennecke *et al*, 2007; Gunawardane *et al*, 2007; Czech *et al*, 2018).

OSCs do not operate the ping‐pong cycle because of the lack of Aub and AGO3 expression. Other factors essential for piRNA amplification, such as Vasa, are also not expressed in OSCs (Malone *et al*, 2009; Saito *et al*, 2009). However, *l(3)mbt* is expressed in OSCs (Sumiyoshi *et al*, 2016). As noted above, piRNA factors were aberrantly expressed in the *l(3)mbt* brains; thus, we reasoned that *l(3)mbt* depletion in OSCs would allow the cells to express Aub, AGO3, and Vasa, along with other germ‐specific ping‐pong factors, amplifying piRNAs that act in post‐transcriptional silencing. Indeed, CRISPR‐Cas9‐mediated genome editing to knock out *l(3)mbt* in OSCs facilitated the expression of Aub and AGO3 (Sumiyoshi *et al*, 2016). Vasa and other piRNA amplifiers have also been detected in L(3)mbt‐lacking OSCs (*i.e*., Δmbt‐OSCs), where Aub and AGO3 initiate the ping‐pong cycle in a manner dependent on Vasa. We hypothesized that L(3)mbt controls genes involved in the piRNA amplification in OSCs, similar to the manner in which it suppresses germline genes in the brain to inhibit tumorigenesis. However, the mechanism by which L(3)mbt controls the target genes in both tissues remains unknown.

In this study, we conducted chromatin immunoprecipitation sequencing (ChIP‐seq) for L(3)mbt in cultured OSCs (Saito *et al*, 2009) concurrently with RNA‐seq in the presence and absence of L(3)mbt and found that L(3)mbt controls the expression of piRNA pathway genes in multiple fashions. This function of L(3)mbt does not largely depend on known L(3)mbt co‐repressors, such as Lint‐1 and Myb (Lewis *et al*, 2004; Georlette *et al*, 2007; Meier *et al*, 2012). We then sought and analyzed L(3)mbt interactors in OSCs and identified CG2662 as a novel L(3)mbt co‐repressor, which was termed “L(3)mbt‐interacting protein in OSCs” (Lint‐O). Comparison of L(3)mbt and Lint‐O ChIP‐seq reads revealed that the L(3)mbt‐bound piRNA amplifier genes were mostly bound with Lint‐O. The piRNA amplifiers derepressed by L(3)mbt depletion were similarly derepressed by Lint‐O depletion. We also found that Lint‐O was unstable in the absence of L(3)mbt, but not *vice versa*, suggesting distinct functionalities of L(3)mbt and Lint‐O despite their tight relationship in gene regulatory function. The *lint‐O* knockout flies, *Lint‐O* ^*KO*^, produced in this study exhibited female sterility at permissive temperatures and developed tumorous brain at restrictive temperatures, similar to the *l(3)mbt‐*deficient flies. We argue that Lint‐O is a novel co‐suppressor of L(3)mbt that cooperates tightly with L(3)mbt to regulate genes, such as piRNA amplification genes, to maintain female fertility and suppress brain tumors.

## Results

### OSCs express two L(3)mbt variants, L(3)mbt‐L and L(3)mbt‐S

**Figure EV1:**
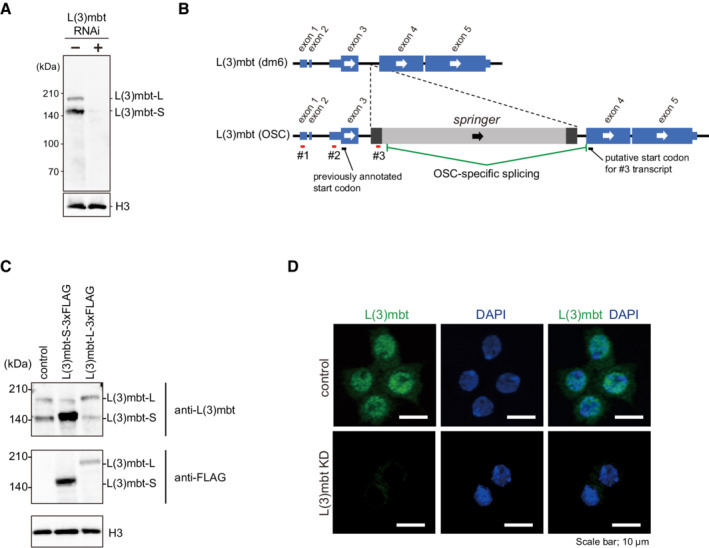
Two isoforms of L(3)mbt, L(3)mbt‐L, and L(3)mbt‐S, in OSCs Western blotting using an anti‐L(3)mbt antibody that we raised in this study detected L(3)mbt as a doublet in the OSC nuclear lysates. The doublet disappeared upon L(3)mbt RNAi. Histone H3 (H3) served as a loading control.The exon‐intron structures of the *l(3)mbt* gene in dm6 (provided by UCSC) and in OSCs (Sienski *et al*, 2012). Note that the *l(3)mbt* gene in OSCs has an LTR‐type transposon, *springer*. RACE detected three different 5′ ends of *l(3)mbt* mRNA (#1, #2, and #3).Top: Western blotting using the anti‐L(3)mbt antibody shows that L(3)mbt‐L‐3xFLAG and L(3)mbt‐S‐3xFLAG exogenously expressed in OSCs co‐migrate with endogenous L(3)mbt‐L and L(3)mbt‐S (control), respectively. Middle: Western blotting was performed using anti‐FLAG antibody. Bottom: Histone H3 (H3) was detected as a loading control.Immunofluorescence analysis using the anti‐L(3)mbt antibody detected L(3)mbt (green) mostly in the OSC nuclei. The L(3)mbt signals disappeared upon L(3)mbt RNAi (KD). DAPI (blue) indicates the nuclei. Scale bar: 10 μm. Source data are available online for this figure.

To conduct genome‐wide ChIP‐seq for L(3)mbt in OSCs, we first produced an anti‐L(3)mbt monoclonal antibody. Western blotting using this antibody detected L(3)mbt as a doublet in OSC lysates (Fig EV1A). Both bands disappeared upon RNA interference (RNAi) treatment for L(3)mbt (Fig EV1A), suggesting that both are L(3)mbt. Hereafter, we refer to the ~ 190 and ~ 150 kDa bands as L(3)mbt‐L and L(3)mbt‐S, respectively.

A previous study showed that the *l(3)mbt* gene bears multiple isoforms of which the expression levels change during development (Wismar *et al*, 1995). We thus inferred that L(3)mbt‐L and L(3)mbt‐S may correspond to two isoforms. To examine whether this inference is correct, we performed rapid amplification of cDNA ends (RACE) experiments using total RNAs isolated from OSCs. Three different 5′ ends of *l(3)mbt* mRNA were detected (#1–3, Fig EV1B). Two distal 5′ ends (#1 and #2) were located within the 5′‐untranslated region (UTR) of the *l(3)mbt* RNA transcript annotated in FlyBase (FBtr0085175). By contrast, the most proximal 5′ end (#3) was not within the annotated *l(3)mbt* sequence. Rather, it was found to be within the 5′ long terminal repeat (LTR) of *springer* retrotransposon inserted into the third intron of *l(3)mbt* in the OSC genome (Sienski *et al*, 2012). This *l(3)mbt* isoform consists of a portion of *springer* (294 bases: nucleotides #152–445) and exons 4 and 5 of the *l(3)mbt* gene (Fig EV1B). Western blotting showed that L(3)mbt expressed from the authentic cDNA and its truncated version in OSCs comigrated with endogenous L(3)mbt‐L and L(3)mbt‐S, respectively (Fig EV1C). Thus, L(3)mbt‐L corresponds to the full‐length (FL) L(3)mbt, and L(3)mbt‐S is its truncated form, which highly likely starts with Met325 of L(3)mbt‐L. The level of piRNAs against *springer* is negligible in OSCs (Sienski *et al*, 2012). Therefore, the allele encoding L(3)mbt‐S is spared from piRNA‐mediated regulation, enabling the expression in OSCs. Immunofluorescence of OSCs using anti‐L(3)mbt antibody detected the L(3)mbt signals nearly exclusively in the nucleus (Fig EV1D), suggesting that both L(3)mbt isoforms are localized to the nucleus, similarly to L(3)mbt in other cell types such as neuroblasts (Richter *et al*, 2011) and cultured Kc cells (Meier *et al*, 2012).

### L(3)mbt controls piRNA amplifiers in multiple ways in OSCs

**Figure 1:**
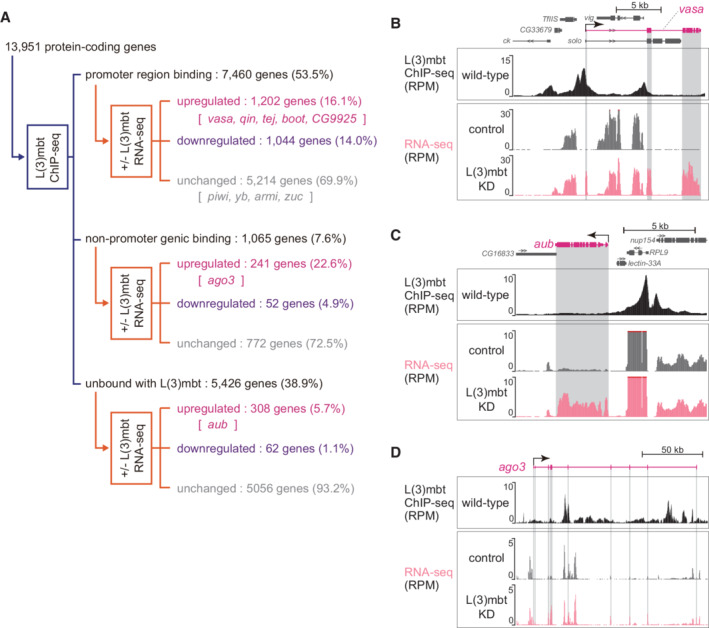
L(3)mbt controls target gene expression in complex fashions A
The 13,951 protein‐coding genes of *Drosophila* were classified into “promoter region binding,” “nonpromoter genic binding,” and “unbound with L(3)mbt” in accordance with the L(3)mbt ChIP‐seq reads, and were subsequently divided into “upregulated,” “downregulated,” and “unchanged” in accordance with the RNA‐seq reads from OSCs before and after L(3)mbt depletion [+/− L(3)mbt]. Representatives of piRNA factors are indicated within the groups. ChIP‐seq was performed twice technically and RNA‐seq three times.B–D
The genomic regions harboring *vasa* (B), *aub* (C), and *ago3* (D). The gene structure is shown at the top. The arrow depicts the TSS and the orientation of transcription. The L(3)mbt ChIP‐seq reads in normal OSCs (wild‐type) and RNA‐seq reads from L(3)mbt‐depleted [L(3)mbt KD] and control OSCs are shown under the gene structure. The shading in gray corresponds to exons. The *y‐*axis shows the number of reads per million mapped reads (RPM).

**Figure EV2:**
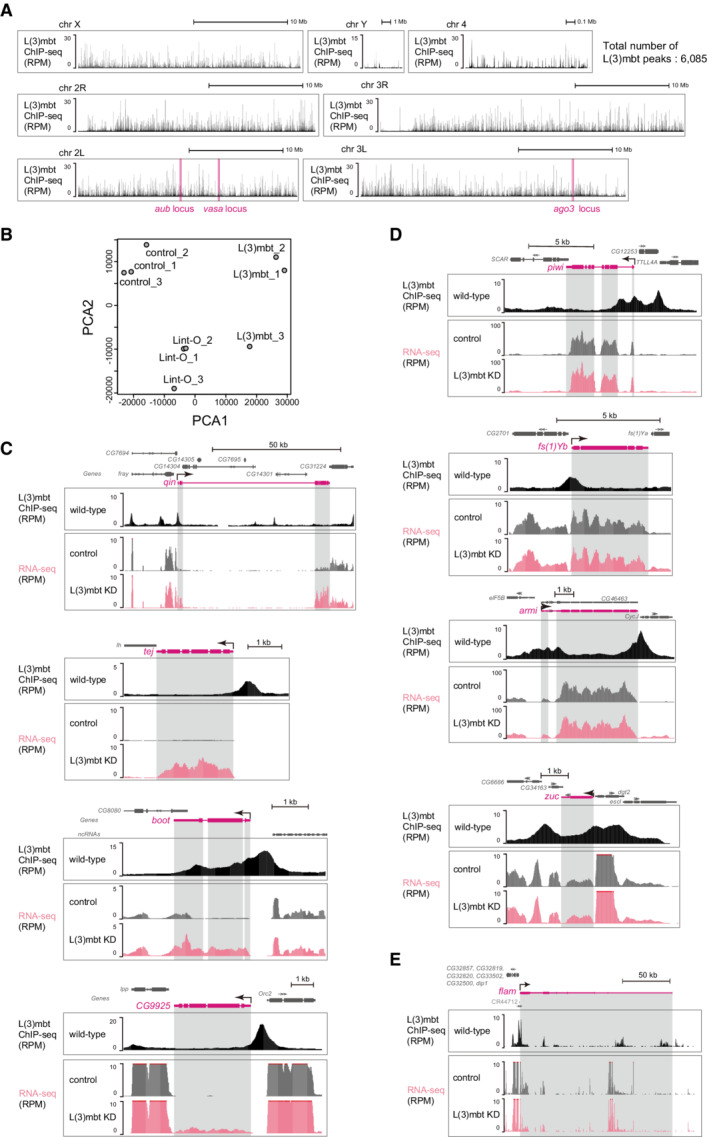
Genomic distribution of L(3)mbt and the effects of L(3)mbt loss on the expression levels of piRNA factors in OSCs A
Genomic browser views of L(3)mbt ChIP‐seq signals. All fly chromosomes are shown. The *vasa*, *ago3*, and *aub* loci are indicated. The total number of L(3)mbt ChIP peaks is shown in upper right.B
PCA shows the correlation in the RNA‐seq libraries. Control: GFP siRNA was used.C–E
The genomic regions harboring *qin*, *tej*, *boot*, and *CG9925* (C), *piwi*, *yb*, *armi*, and *zuc* (D), and *flam* (E).

We performed L(3)mbt ChIP‐seq in OSCs using the anti‐L(3)mbt monoclonal antibody. The experiment was conducted twice, and statistical analysis confirmed the high correlation between the two libraries (Appendix Fig S1A). An overview of the ChIP‐seq reads mapped on the *Drosophila* genome is presented in Fig EV2A. We then extracted ChIP‐seq reads corresponding to 13,951 protein‐coding genes in Flybase by allowing the reads to map the promoter region of each gene and analyze. Note that the promoter region in this study refers to the genomic region spanning from 0.35 kilobase (kb) upstream of the transcriptional start site (TSS) to 0.1 kb downstream of the TSS, based on the previously reported DNA‐binding status of L(3)mbt in *Drosophila* larval tissues (Richter *et al*, 2011). This analysis showed that L(3)mbt can bind to 8,525 of 13,951 protein‐coding genes (61.1%) (Fig 1A). We further classified these genes according to where in each gene L(3)mbt is bound. This revealed that 7,460 genes had promoter region binding of L(3)mbt (53.5% in 13,951 genes). We designated this group as “promoter region binding.” The remaining 1,065 genes were designated as “nonpromoter genic binding” (7.6%). The 5,426 genes that did not belong to either group were designated as “unbound with L(3)mbt” (38.9%).

Comparison of the ChIP‐seq reads with the OSC RNA‐seq reads before and after L(3)mbt depletion revealed that the expression levels of 1,202 genes among the 7,460 “promoter region binding” genes were upregulated by L(3)mbt loss (16.1%), while 1,044 genes were downregulated (14.0%) (Fig 1A). Note that RNA‐seq was conducted three times, and principal component analysis (PCA) confirmed the high correlation in the libraries (Fig EV2B). We previously showed that seven germ‐specific piRNA biogenesis genes ‐ *vasa*, *aub*, *ago3*, *qin*, *tejas* (*tej*), *bootlegger* (*boot*), and *CG9925* ‐ were among the genes prominently upregulated by L(3)mbt depletion (Sumiyoshi *et al*, 2016). In this study, we found that five of these genes ‐ *vasa*, *qin*, *tej*, *boot*, and *CG9925* ‐ were within the “upregulated” category of the “promoter region binding” group (Fig 1A). The patterns of binding of L(3)mbt to these genes and the RNA‐seq signals at the loci are shown in Fig 1B (*vasa*) and Fig EV2C (*qin*, *tej*, *boot*, and *CG9925*).

The key somatic piRNA factors, *piwi*, *female sterile(1)Yb* (*yb*), *armitage* (*armi*), and *zucchini* (*zuc*), all of which are intrinsically expressed in normal OSCs, belonged to the “promoter region binding” group (Fig 1A). Their expression levels were unchanged by the loss of L(3)mbt (Fig EV2D), agreeing with our previous observation (Sumiyoshi *et al*, 2016). We previously showed that Piwi expression in OSCs depends on the transcriptional factor Traffic jam (Tj) (Saito *et al*, 2009), indicating that as long as Tj is functional, Piwi will be expressed regardless of the presence or absence of L(3)mbt. It seems that the regulation of *piwi* via Tj takes precedence over the regulation via L(3)mbt.

Likewise, the promoter of *flamenco* (*flam*), the piRNA cluster responsible for bearing most piRNAs within OSCs (Brennecke *et al*, 2007), was bound with L(3)mbt via the promoter, but the expression level was not impacted by the lack of L(3)mbt (Fig EV2E). An earlier study showed that the expression of *flam* depends on the transcriptional factor Cubitus interruptus (Goriaux *et al*, 2014). L(3)mbt binds to a relatively wide range of genes, but for genes functioning in OSCs, there appear to be activators that override the repression by L(3)mbt.

The *aub* and *ago3* genes were classified into the “unbound with L(3)mbt” and “nonpromoter genic binding” categories, respectively (Fig 1A). Both genes were upregulated by L(3)mbt depletion (Fig 1C and D), as expected based on our earlier observation (Sumiyoshi *et al*, 2016). At the *aub* locus, the L(3)mbt binding was concentrated in the upstream part of *aub*, but all sites were distant from the TSS (Fig 1C). From the perspective of neighboring genes, the L(3)mbt signals were rich in the promoter regions of *nup154* and *RPL9*. However, the expression levels were not changed by the lack of L(3)mbt (Fig 1C). In this regard, *nup154* and *RPL9* can be considered as equivalents of *piwi* and *yb*. The L(3)mbt ChIP signals at the *ago3* gene were biased to introns but not to the promoter (Fig 1D). It seems that L(3)mbt genomic binding is not always linked to gene regulation and that L(3)mbt regulates piRNA pathway genes in multiple fashions.

L(3)mbt ChIP‐seq reads and OSC RNA‐seq reads before and after L(3)mbt depletion were also classified based on the distance from most proximal TSS (Appendix Fig S1B and C). Note that the “L(3)mbt in the TSS region” group corresponds to the “promoter region binding” group in Fig 1A.

### Known L(3)mbt co‐suppressors are likely irrelevant for L(3)mbt‐driven gene regulation in OSCs

**Figure 2:**
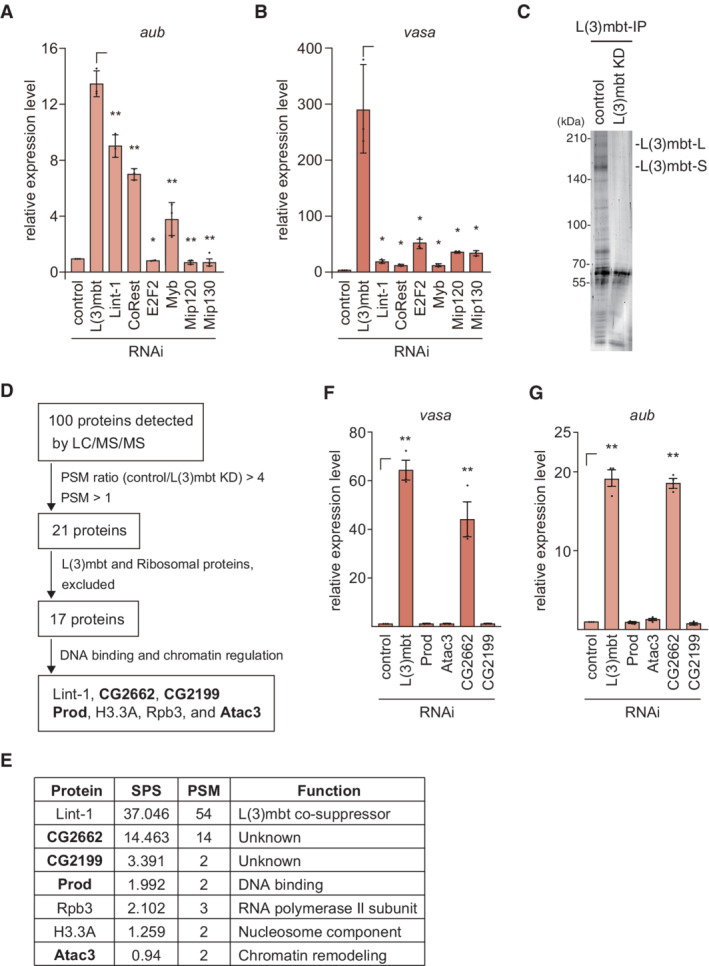
CG2662/Lint‐O binds to and functions with L(3)mbt in gene regulation A, B
The mRNA levels of *aub* (A) and *vasa* (B) were quantified upon the depletion (RNAi) of L(3)mbt, Lint‐1, CoRest, E2F2, Myb, Mip120, and Mip130, and were compared to those in normal OSCs (control). Data represent the mean ± SE (*n* = 3 biological replicates). The *P* values were calculated with the *t*‐test. *P < 0.05, **P < 0.01. All *t*‐tests were performed against samples with⎾ symbol.C
Proteins immunoprecipitated with the anti‐L(3)mbt antibody from the OSC lysates before (control) and after L(3)mbt knockdown (KD) were silver‐stained. The bands corresponding to L(3)mbt‐L and L(3)mbt‐S are indicated.D
Flow chart of the identification of L(3)mbt interactors in OSCs. All peptides obtained from LC–MS/MS are listed in Appendix Table S1.E
Summary of the sum of the −log posterior error probability (Sum Pep Score: SPS), peptide spectra match values (PSM), and known functions of the proteins in (D). Mass spectrometry analysis was performed on biological duplicates.F, G
The mRNA levels of *vasa* (F) and *aub* (G) were quantified upon the depletion (RNAi) of L(3)mbt, Atac3, Prod, CG2662/Lint‐O, and CG2199, and were compared to those in normal OSCs (control). Data represent the mean ± SE (*n* = 3 biological replicates). The *P* values were calculated with the *t*‐test. **P < 0.01. All *t*‐tests were performed against samples with⎾ symbol. Source data are available online for this figure.

L(3)mbt resides in two repressive chromatin complexes, the LINT complex (Meier *et al*, 2012) and the dREAM complex [*a.k.a*. the Myb–MuvB (MMB) complex] (Lewis *et al*, 2004; Georlette *et al*, 2007; Blanchard *et al*, 2014). An earlier genetic study showed that the L(3)mbt function in the ovaries requires Lint‐1, a component of the LINT complex but is independent of the dREAM complex (Coux *et al*, 2018). To understand in which of these two complexes L(3)mbt exerts its function in OSCs, we depleted components of the two complexes by RNAi (Appendix Fig S2A and B) and examined how these treatments affected the levels of *aub*, *ago3*, *piwi*, *and vasa*. The expression of *aub* was upregulated by the loss of Lint‐1 and CoRest, both of which are LINT complex components, but this upregulation was milder than that following L(3)mbt loss (Fig 2A). Upon depletion of E2F2, Mip120, and Mip130, all of which are contained within the dREAM complex, the level of *aub* was barely changed. Knockdown of Myb in the dREAM complex slightly upregulated *aub*, but the effect was not as great as that of Lint‐1 or CoRest knockdown (Fig 2A). These findings suggest that L(3)mbt function in regulating the *aub* gene in OSCs is independent of the dREAM complex but dependent on the LINT complex, similar to the circumstances in the ovaries, but with lower extent. The changes in the level of *ago3* under these conditions were similar to those of *aub* (Appendix Fig S2C). The levels of *piwi* remained stable under each condition, as expected (Appendix Fig S2D). In sharp contrast, the increases in the expression of *vasa* following the loss of all of these known L(3)mbt co‐factors were much lower than that following L(3)mbt depletion (Fig 2B). One plausible scenario is that, when L(3)mbt controls target genes via the promoter (*e.g*., *vasa*), it is almost independent of both LINT and dREAM complexes, but when L(3) controls target genes via regions other than the promoter (*e.g*., *aub* and *ago3*), it may subtly depend on the LINT complex.

### Lint‐O is a novel co‐repressor of L(3)mbt in OSCs

Our findings above prompted us to identify novel L(3)mbt co‐suppressor(s). To this end, we isolated the L(3)mbt complex from OSCs before and after L(3)mbt RNAi (Fig 2C) and forwarded the materials for mass spectrometric analysis. Peptides corresponding to 100 proteins were obtained from normal OSCs (Fig 2D and Appendix Table S1). The number of peptides corresponding to 21 proteins among these 100 proteins was reduced four‐fold or more by L(3)mbt depletion (Appendix Table S1). Exclusion of the ribosomal proteins and L(3)mbt reduced the number of candidate proteins to 17. Gene Ontology (GO) analysis categorized 7 of the 17 proteins as DNA‐binding and chromatin regulation factors (Fig 2D and E).

The effects of Lint‐1 deficiency on *aub*, *vasa*, and *ago3* were already examined (Fig 2A and B and Appendix Fig S2C). Histone H3.3A and RNA polymerase II subunit 3 (Rpb3) seemed to be rather general factors and were therefore not considered to be strong candidates. We then examined the contribution of four other factors, CG2662, CG2199, proliferation disrupter (Prod), and Ada2a‐containing complex component 3 (Atac3), in the regulation of *vasa* and *aub*. Quantitative reverse‐transcription PCR (RT–qPCR) showed that the level of *vasa* was increased upon the depletion of CG2662, similar to that of L(3)mbt, but was unchanged by the depletion of CG2199, Prod, or Atac3 (Fig 2F and Appendix Fig S2E). The level of *aub* was also influenced by CG2662 loss as efficiently as L(3)mbt loss (Fig 2G). These findings suggested that CG2662 is a co‐suppressor of L(3)mbt more closely related to it than any known L(3)mbt co‐suppressors, including Lint‐1. Thus, we termed CG2662 “L(3)mbt‐interacting protein in OSCs” (Lint‐O) and continued our investigation to reveal the functional relationship between L(3)mbt and Lint‐O.

### The L(3)mbt‐Lint‐O interaction is necessary for piRNA pathway gene regulation

**Figure 3:**
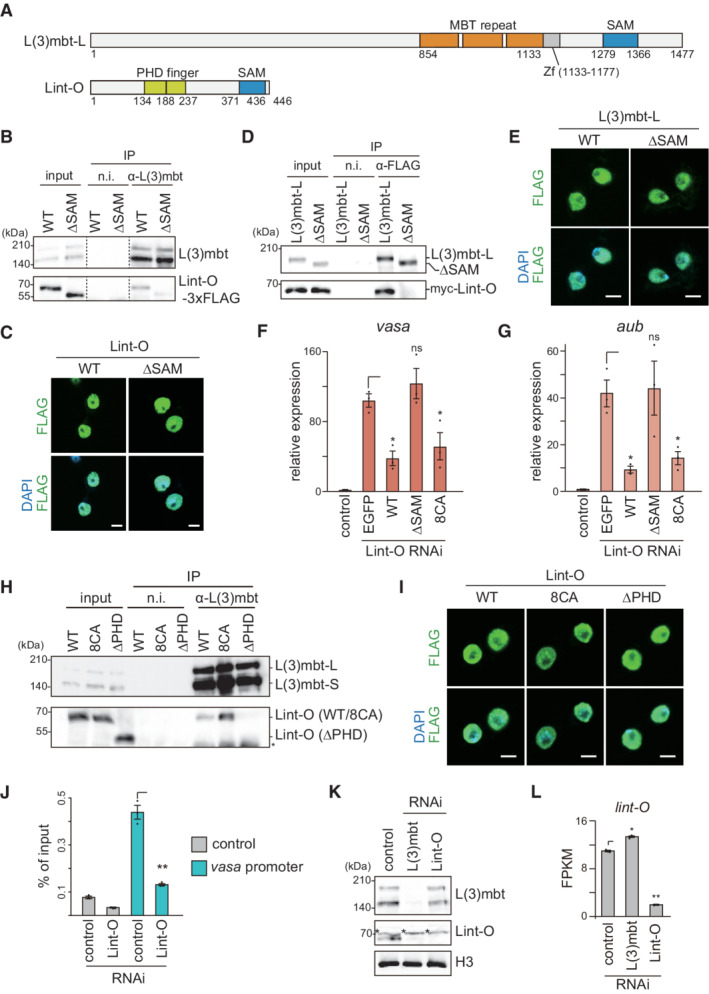
The L(3)mbt‐Lint‐O interaction is crucial for the repression of target genes A
Domain structures of L(3)mbt and Lint‐O. L(3)mbt has three MBT repeats (orange), a C2H2‐type zinc finger (Zf) (gray), and a SAM domain (blue). Lint‐O has two PHD finger domains (green) and a SAM domain (blue).B
Immunoprecipitation (IP)/western blotting shows that WT Lint‐O, but not its ΔSAM mutant (Fig EV3A), co‐immunoprecipitated with L(3)mbt from the OSC lysates. n.i., nonimmune IgG.C
Subcellular localization of WT Lint‐O and its ΔSAM mutant (green). Both proteins were localized to the nuclei (DAPI: blue). Scale bar: 5 μm.D
IP/western blotting shows that WT L(3)mbt‐L, but not its ΔSAM mutant (Fig EV3B), co‐immunoprecipitated with Lint‐O from the OSC lysates. n.i., nonimmune IgG.E
Subcellular localization of WT L(3)mbt‐L and its ΔSAM mutant (green). Both proteins were localized to the nuclei (DAPI: blue). Scale bar: 5 μm.F, G
The mRNA levels of *vasa* (F) and *aub* (G) were quantified upon ectopic expression of EGFP, and WT Lint‐O and its ΔSAM and 8CA mutants (Fig EV3A), in Lint‐O‐lacking OSCs (Lint‐O RNAi) and were compared to those in normal OSCs (control). Data represent the mean ± SE (*n* = 3 biological replicates). The *P* values were calculated with the *t*‐test. *P < 0.05. All *t*‐tests were performed against samples with⎾ symbol.H
IP/western blotting shows that L(3)mbt co‐immunoprecipitated with WT Lint‐O and its 8CA mutant, but not with ΔPHD mutant (Fig EV3A). An asterisk shows the background.I
Subcellular localization of WT Lint‐O and its 8CA and ΔPHD mutants (green). All proteins were localized to the nuclei (DAPI: blue). Scale bar: 5 μm.J
ChIP–qPCR shows that L(3)mbt binding to the *vasa* promoter was weakened after the loss of Lint‐O. Data represent the mean ± SE (*n* = 3 biological replicates). The *P* values were calculated with the *t*‐test. **P < 0.01. All *t*‐tests were performed against samples with⎾ symbol.K
Western blotting showing the amounts of L(3)mbt, Lint‐O, and histone H3 (H3) in normal (control), L(3)mbt‐depleted (RNAi), and Lint‐O‐depleted (RNAi) OSCs. Asterisks show background signals as in Fig EV3E.L
The *lint‐O* mRNA levels in normal (control), L(3)mbt‐depleted (RNAi), and Lint‐O‐depleted (RNAi) OSCs are shown by fragments per kilobase million (FPKM). Data represent the mean ± SE (*n* = 3 biological replicates). The *P* values were calculated with the *t*‐test. *P < 0.05, **P < 0.01. All *t*‐tests were performed against samples with⎾ symbol. Source data are available online for this figure.

L(3)mbt contains three malignant brain tumor (MBT) repeats responsible for binding methylated histone marks (Wismar *et al*, 1995; Bonasio *et al*, 2010), a C2H2‐type zinc finger, and a sterile alpha motif (SAM) domain that acts in protein–protein interactions (Kim & Bowie, 2003) (Fig 3A). Lint‐O consists of two plant homeodomain (PHD) finger domains and a SAM domain (Fig 3A). The PHD finger is often found in chromatin‐associated proteins that read histone H3 marks such as H3K4me2/3 to regulate target gene expression (Sanchez & Zhou, 2011).

**Figure EV3:**
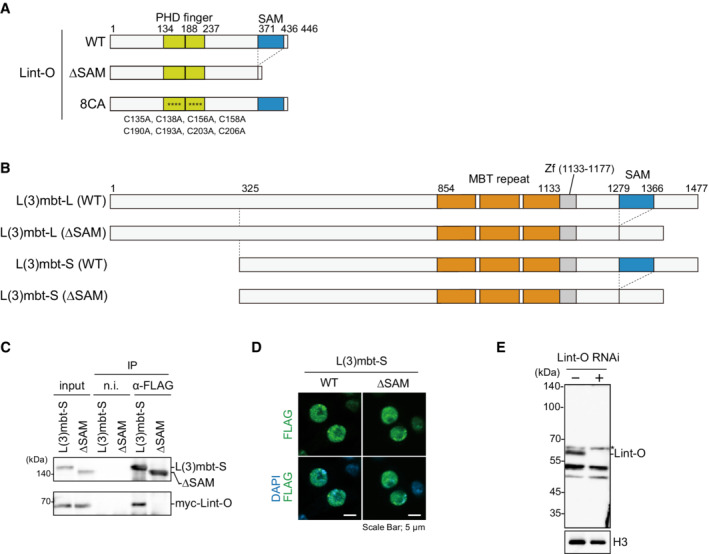
Doman structures of L(3)mbt, Lint‐O and their mutants and behavior of L(3)mbt‐S in OSCs Domain structures of WT Lint‐O and its ΔSAM and 8CA mutants. The ΔSAM mutant is composed of Met1‐Val371 and Ser437‐Asp446. The Cys‐to‐Ala mutations in the 8CA mutant are indicated at the bottom (asterisks in the structure).Domain structures of WT L(3)mbt‐L, WT L(3)mbt‐S, and their ΔSAM mutants. The ΔSAM mutant of L(3)mbt‐L is composed of Met1‐Leu1278 and Val1367‐Ser1477 of WT L(3)mbt‐L. The ΔSAM mutant of L(3)mbt‐S is composed of Met325‐Leu1278 and Val1367‐Ser1477 of WT L(3)mbt‐L.IP/western blotting shows that WT L(3)mbt‐S, but not its ΔSAM mutant, co‐immunoprecipitated with Lint‐O from the OSC lysates. n.i., nonimmune IgG.Subcellular localization of WT L(3)mbt‐S and its ΔSAM mutant (green). Scale bar: 5 μm.Western blotting using the anti‐Lint‐O antibodies raised in this study. The Lint‐O band (~60 kDa) was observed in normal OSCs (Lint‐O RNAi−) but not in Lint‐O‐depleted OSCs (Lint‐O RNAi+). Histone H3 (H3) was detected as a loading control. An asterisk shows the background. Source data are available online for this figure.

To confirm the association between L(3)mbt and Lint‐O, we ectopically expressed wild‐type (WT) Lint‐O, tagged with 3x FLAG at the C‐terminus, in OSCs. The L(3)mbt complex immunopurified from the OSC lysates was then probed with anti‐FLAG and anti‐L(3)mbt antibodies. WT Lint‐O signal was detected along with that of L(3)mbt (Fig 3B). However, when the Lint‐O mutant lacking the SAM domain, ΔSAM (Fig EV3A), was expressed instead of WT Lint‐O, the mutant failed to co‐immunoprecipitate with L(3)mbt (Fig 3B). The ΔSAM mutant was localized to the nucleus as was the WT control (Fig 3C). In subsequent experiments, both L(3)mbt‐L and L(3)mbt‐S, but not their ΔSAM mutants, bound to Lint‐O (Figs 3D and EV3B and C), although all those proteins were localized to the nucleus (Figs 3E and EV3D). These findings confirm the L(3)mbt‐Lint‐O association in OSCs and indicate that their interaction depends on the SAM domain of each of the two proteins.

We next investigated whether the L(3)mbt‐Lint‐O interaction is important for regulating target gene expression. RT–qPCR showed that *vasa* and *aub* were upregulated in Lint‐O‐lacking OSCs (Fig 3F and G). We then ectopically expressed RNAi‐resistant WT Lint‐O in these cells. This treatment rescued the defects caused by the loss of endogenous Lint‐O (Fig 3F and G). When the ΔSAM mutant, which was also RNAi‐resistant, was expressed, the *vasa* and *aub* genes were not re‐silenced (Fig 3F and G). These findings indicated that the L(3)mbt‐Lint‐O interaction via the SAM domain is essential for the cooperative function of L(3)mbt and Lint‐O in regulating the gene targets.

These rescue assays were also performed using another Lint‐O mutant, 8CA, where eight cysteines within the two PHD finger domains were altered to alanine (Fig EV3A). This mutant was not expected to re‐repress *vasa* and *aub* because other PHD finger‐containing proteins have been shown to anchor zinc ions via the cysteines and this ion anchoring is essential for these proteins to exert their functions (Kalkhoven *et al*, 2002). However, contrary to our expectations, the 8CA mutant re‐repressed *vasa* and *aub* to an extent similar to that of the WT (Fig 3F and G). Thus, Lint‐O may not require zinc ions to regulate its target genes. The 8CA mutant was able to interact with L(3)mbt (Fig 3H) and localize to the nucleus (Fig 3I). However, when the PHD finger domains were removed from Lint‐O (Fig EV3A), the ΔPHD mutant was unable to interact with L(3)mbt (Fig 3H) although its nuclear localization was unaffected by the lack of PHD domains (Fig 3I). These results support the idea that the ability of Lint‐O to associate with chromatin via the PHD domains influences the L(3)mbt‐Lint‐O interaction. This idea was further supported by the observation that the binding activity of L(3)mbt to the *vasa* promoter was significantly weakened by the absence of Lint‐O (Fig 3J). The binding activity of Lint‐O to L(3)mbt appeared to be slightly increased by the 8CA mutation (Fig 3H). This may be because the mutation stabilized the binding of Lint‐O to chromatin.

In the course of our study, we noticed that, in the absence of L(3)mbt, endogenous Lint‐O was barely detected by western blotting using anti‐Lint‐O antibodies raised in this study (Figs 3K and EV3E). The level of *lint‐O* mRNA was not impacted by the lack of L(3)mbt (Fig 3L). By contrast, Lint‐O depletion little influenced the level of L(3)mbt (Fig 3K). Thus, the L(3)mbt‐Lint‐O interaction is required for the stabilization of Lint‐O but not of L(3)mbt. These results support the idea that L(3)mbt and Lint‐O have distinct functions even though they bind to each other and cooperate in gene regulation in OSCs.

### L(3)mbt and Lint‐O cooperate in regulating the expression of piRNA amplifiers in OSCs

**Figure 4:**
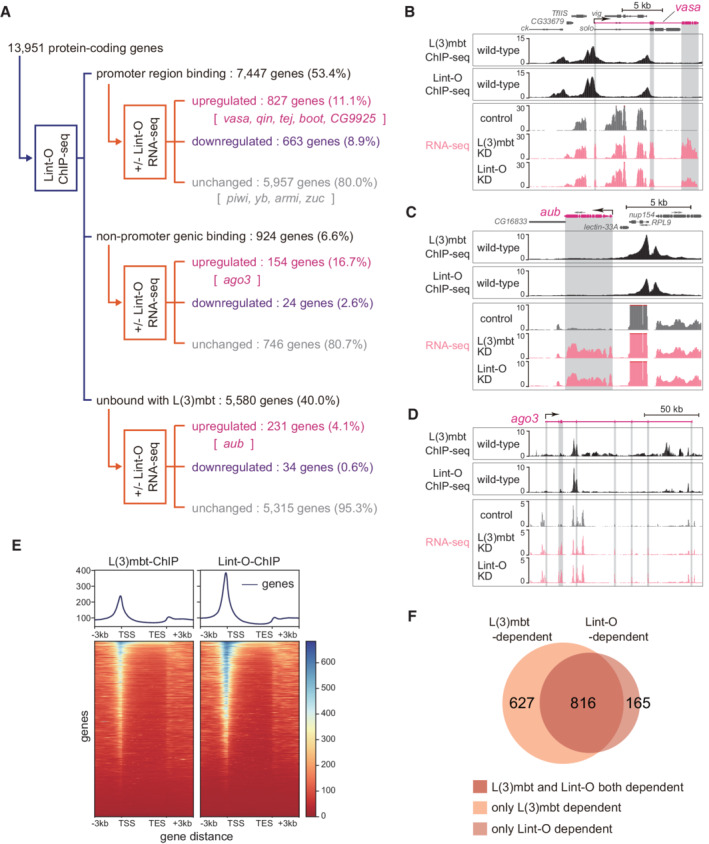
L(3)mbt and Lint‐O share genomic binding to orchestrate gene control in OSCs A
The 13,951 protein‐coding genes of *Drosophila* were classified into “promoter region binding,” “nonpromoter genic binding,” and “unbound with Lint‐O” in accordance with the Lint‐O ChIP‐seq reads, and were subsequently divided into “upregulated,” “downregulated,” and “unchanged” in accordance with the RNA‐seq reads from the OSCs before and after Lint‐O depletion (+/− Lint‐O). Representatives of piRNA factors are indicated. ChIP‐seq was performed twice technically and RNA‐seq three times.B–D
The genomic regions harboring *vasa* (B), *aub* (C), and *ago3* (D) are indicated. The L(3)mbt and Lint‐O ChIP‐seq reads and the RNA‐seq reads from normal (control), L(3)mbt‐depleted, and Lint‐1‐depleted OSCs are shown. The L(3)mbt ChIP‐seq reads and the RNA‐seq reads from normal (control) and L(3)mbt‐depleted were also shown in Fig 1B–D.E
Heatmaps show the L(3)mbt ChIP and Lint‐O ChIP scores calculated by deeptools (Ramírez *et al*, 2016) within each gene body and the extended regions (*i.e*., 3 kb upstream of the TSS and 3 kb downstream of the TES). The length of all the genes is normalized to be a constant value, 5 kb. The summary plots show the average score.F
Venn diagram showing the overlap between L(3)mbt‐ and Lint‐O‐regulated genes.

**Figure EV4:**
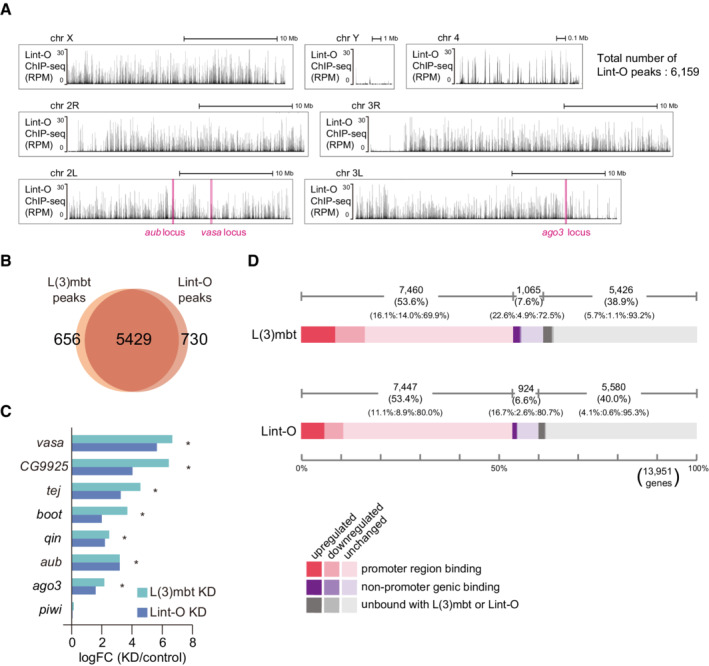
Lint‐O OSC ChIP‐seq and RNA‐seq before and after Lint‐O depletion in OSCs Genomic browser views of the Lint‐O ChIP‐seq reads. All fly chromosomes are shown. The *y*‐axis shows the number of RPM. The *vasa*, *ago3*, and *aub* loci are indicated. The total number of Lint‐O ChIP peaks is shown in upper right.Overlap between Lint‐O ChIP peaks in (A) and L(3)mbt ChIP peaks (Fig EV2A).
*vasa*, *CG9925*, *tej*, *boot*, *qin*, *aub*, and *ago3* were identified as upregulated genes (* *q*‐value <0.05) in RNA‐seq analysis. The log fold change of the differential expression for each piRNA pathway gene was calculated based on the RNA‐seq data from normal (control), L(3)mbt‐depleted (KD), and Lint‐O‐depleted (KD) OSCs. The *y*‐axis shows the ratio of the read counts between L(3)mbt‐ and Lint‐O‐depleted OSCs and the normal OSCs.Overview and comparison of the gene classification in Figs 1A and 4A.

We next conducted Lint‐O ChIP‐seq in OSCs using the anti‐Lint‐O antibodies. The experiment was conducted twice, and statistical analysis confirmed the high correlation between the two libraries (Appendix Fig S3A). An overview of ChIP‐seq reads mapped on the *Drosophila* genome is presented in Fig EV4A. The degree of overlap between Lint‐O peaks and L(3)mbt peaks is indicated in Fig EV4B. Further analysis showed that Lint‐O bound to 7,447 protein‐coding genes via their promoter regions (53.4% of a total of 13,951 genes) (“promoter region binding” in Fig 4A). Comparison of the ChIP‐seq reads with the OSC RNA‐seq reads before and after Lint‐O depletion (Fig EV2B) revealed that 827 genes of the 7,447 “promoter region binding” group were upregulated by the loss of Lint‐O (11.1%), whereas 663 genes were downregulated (8.9%) (Fig 4A). The *vasa*, *qin*, *tej*, *boot*, and *CG9925* genes belonged to the “upregulated” category as they belonged to the equivalent in L(3)mbt ChIP‐seq (Fig 1A). The expression levels of the other 5,957 genes were little impacted by Lint‐O depletion (80.0%) (Fig 4A). The *piwi*, *yb*, *armi*, and *zuc* genes belonged to this category as they belonged to the counterpart in Fig 1A.

A total of 924 of 13,951 protein‐coding genes were classified into the “nonpromoter genic binding” group (6.6%) (Fig 4A). Of these, 154 genes were upregulated by the loss of Lint‐O (16.7%) and the *ago3* gene was in this category. In addition, 5,580 genes were “unbound with Lint‐O” (40.0%) (Fig 4A). Of these, 231 genes were upregulated by the loss of Lint‐O (4.1%), and *aub* belonged to this category. The browser views of Lint‐O ChIP‐seq and RNA‐seq in the presence and absence of Lint‐O at the *vasa*, *aub*, and *ago3* loci are shown in Fig 4B–D. Basically, the Lint‐O genomic binding patterns are nearly identical to those of L(3)mbt, further indicating the collaborative functions of the two proteins in piRNA amplifier gene regulation.

The five “promoter region binding” genes (*i.e*., *vasa*, *CG9925*, *tej*, *boot*, and *qin*) and two “nonpromoter genic binding” genes (*i.e*., *aub* and *ago3*) were similarly upregulated in L(3)mbt‐ and Lint‐O‐depleted OSCs (Fig EV4C). In each case, the lack of Lint‐O appeared less influential than that of L(3)mbt. Such a trend was also observed in the number of genes affected by the lack of L(3)mbt/Lint‐O (based on RNA‐seq) (Fig EV4D); namely, the number of genes influenced by the lack of L(3)mbt was slightly higher than that influenced by the lack of Lint‐O. This may be related to the observation that Lint‐O becomes unstable upon the lack of L(3)mbt (Fig 3K).

Lint‐O ChIP‐seq reads and OSC RNA‐seq reads before and after Lint‐O depletion was also classified based on the distance from most proximal TSS (Appendix Fig S3B). We also analyzed the binding frequencies of L(3)mbt and Lint‐O to the TSS and transcriptional end sites (TES) of the protein‐coding genes. The histogram indicated that both proteins tend to bind strongly to the TSS and weakly to the TES and downstream regions (Fig 4E). L(3)mbt and Lint‐O may rarely repress target genes by binding to introns (*e.g*., *ago3*) or upstream regions far from the TSS (*e.g*., *aub*). The regulation of *aub* and/or *ago3* by L(3)mbt and Lint‐O may also be indirectly mediated by transcription factors or other regulators that are directly activated by the loss of L(3)mbt/Lint‐O.

A previous study showed that brain tumors in *l(3)mbt* mutant flies originated from deregulation of target genes in the Salvador‐Warts‐Hippo (SWH) pathway (Richter *et al*, 2011). Another study asserted that inappropriate expression of Nanos (Nos), a translational repressor and key regulator of germline fate, in ovarian follicular (somatic) cells was the main cause of defects in germline development observed in the *l(3)mbt* mutants (Coux *et al*, 2018). ChIP‐seq and RNA‐seq at two genes under the control of the SWH pathway, *drosophila inhibitor of apoptosis 1* and *expanded*, showed that both L(3)mbt and Lint‐O bound to the promoters of the two genes, albeit weakly, but the mRNA levels were barely changed by the loss of L(3)mbt or Lint‐O (Appendix Fig S3C). This suggested that dependence on the L(3)mbt/Lint‐O functions varies among cells. The *nos* promoter was bound with L(3)mbt and Lint‐O relatively strongly and the mRNA level was upregulated following L(3)mbt or Lint‐O depletion, but the expression was still far lower than that of *nos* in the ovary in the FlyAtlas 2 (Leader *et al*, 2018) (Appendix Fig S3D and E). The expression of *nos* may be activated when OSCs reside in the vicinity of germ cells in the ovary.

### L(3)mbt and Lint‐O may function independently of each other in gene regulation

MBTS is not restricted to germ‐specific piRNA factors (Janic *et al*, 2010). For the next part of our study, we collected 1,443 protein‐coding genes under the control of L(3)mbt [*i.e*., genes that were bound with L(3)mbt, irrespective of whether this was promoter binding or nonpromoter binding, and upregulated by the L(3)mbt depletion] (Fig 1A) and 981 genes under the control of Lint‐O (*i.e*., genes that were bound with Lint‐O, irrespective of whether this was promoter binding and nonpromoter binding, and upregulated by the loss of Lint‐O) (Fig 4A), and compared the gene pools. The Venn diagram showed that 816 genes were shared between the two groups (Fig 4F). We attempted to sort these genes according to GO terms, but the statistical analysis did not enrich for significant terms. We next focused on 627 genes that were suppressed via the L(3)mbt binding but in a Lint‐O‐independent matter. These genes could be controlled by the combination of L(3)mbt and co‐factors other than Lint‐O [*i.e*., “L(3)mbt‐dependent and Lint‐O‐independent”]. However, no significant GO terms were found. A similar result was obtained for “L(3)mbt‐independent and Lint‐O‐dependent” genes (165 genes). We also focused on downregulated genes. We found that 439 genes were downregulated by depletion of both L(3)mbt and Lint‐O (Appendix Fig S3F). Again, no significant terms were found. L(3)mbt/Lint‐O appears to regulate a wide variety of intracellular pathway genes in OSCs.

### Lint‐O is essential for ovarian morphogenesis and oocyte development

**Figure 5:**
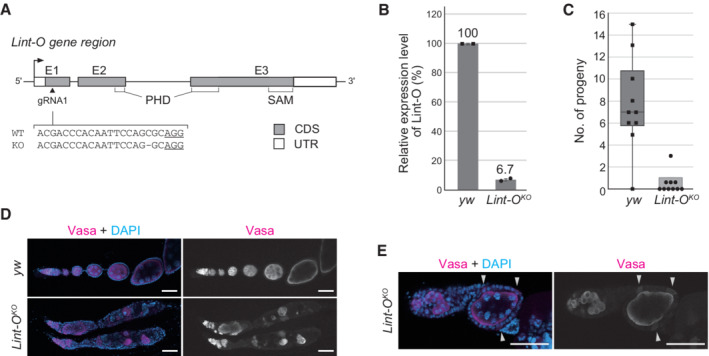
The lack of Lint‐O in flies leads to abnormal ovarian morphology and female sterility CRISPR‐mediated generation of *lint‐O* knockout (KO). Genomic structure of *lint‐O* is shown with gRNA targeting *lint‐O* exon 1 (triangle). Sequences of *lint‐O* DNA in WT (*y w*) (complementary to the gRNA sequence) and the KO mutant, *Lint‐O* ^*KO*^, are shown. PAM: protospacer adjacent motif (underlined). E1‐3: Exons 1–3. CDS: protein‐coding sequence. UTR: untranslated region.RT–qPCR analysis shows the mRNA levels of *lint‐O* in *y w* and *Lint‐O* ^*KO*^ ovaries. Data are expressed as mean and error bars represent SD. *n* = 2 biological replicates.The numbers of progeny in *y w* and *Lint‐O* ^*KO*^ are shown. Ten independent crosses were performed. Boxplot central bands, upper edges of boxes, lower edges of boxes, upper whiskers, and lower whiskers show median, third quartile, first quartile, maxima, and minima, respectively.Confocal images of *y w* and *Lint‐O* ^*KO*^ ovaries immunostained for Vasa. Scale bar: 50 μm.Confocal images of *y w* and *Lint‐O* ^*KO*^ ovarioles immunostained for Vasa. Vasa was ectopically expressed in follicle cells (white arrowheads). Scale bar: 50 μm.

To investigate the importance of Lint‐O function in flies, we generated *lint‐O*‐deficient mutants by applying the CRISPR‐Cas9 system. A guide RNA was designed to introduce an indel mutation in the *lint‐O* coding sequence (Fig 5A). A mutant fly harboring a single‐nucleotide deletion was obtained. In this mutant, the *lint‐O* expression was severely attenuated (Fig 5B); thus, the *lint‐O* mutant was designated *Lint‐O* ^*KO*^. The mutant flies were viable at permissive temperatures but exhibited female infertility at 29°C (Fig 5C) as the temperature‐sensitive *l(3)mbt* mutant, *L(3)mbt* ^*ts1*^, showed female sterility at permissive temperatures (Coux *et al*, 2018).

**Figure EV5:**
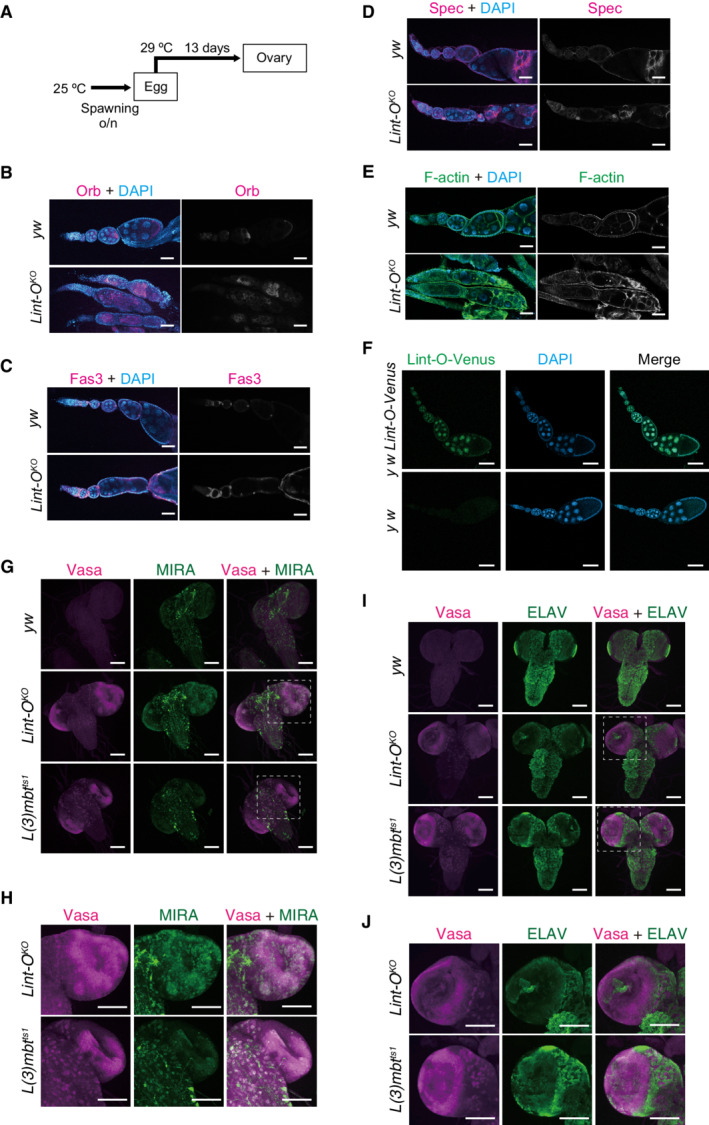
Lint‐O ^***KO***^ **ovaries and larval brains** A
Schema depicting the experiment. Eggs were transferred to 29°C after spawning at 25°C and incubated for 13 days. Ovaries were dissected from adult flies.B–E
Confocal images of brains of *y w*, *Lint‐O* ^*KO*^, and *L(3)mbt* ^*ts1*^ larvae (grown at 29°C) for Orb (B), Fas3 (C), Spectrin (Spec) (D), and F‐actin (E). *Lint‐O* ^*KO*^ ovariole showed defects in follicle cell layer integrity. DAPI (blue): nuclei. Scale bars: 50 μm.F
The *Lint‐O‐Venus* fly line (*y w Lint‐O‐Venus*) was generated using the CRISPR /Cas9 system. The Venus sequence was inserted before the stop codon of Lint‐O. The Lint‐O‐Venus signal (green) was detected in the nucleus of both germ and follicle cells in the ovaries. Nuclei were stained with DAPI (blue). Wild‐type (*y w*) was used as a negative control. Scale bars:  50 μm.G
Confocal images of *y w*, *Lint‐O* ^*KO*^, and *L(3)mbt* ^*ts1*^ immunostained for Vasa (magenta) and MIRA (green). Merged images were also presented in Fig 6B. Scale bars: 100 μm.H
Enlarged views of insets in (G). Scale bars:  50 μm.I
Confocal images of *y w*, *Lint‐O* ^*KO*^, and *L(3)mbt* ^*ts1*^ immunostained for Vasa (magenta) and ELAV (green). Merged images were also presented in Fig 6B. Scale bars: 100 μm.J
Enlarged views of insets in (I). Scale bars: 100 μm.

The *Lint‐O* ^*KO*^ ovaries appeared to be morphologically abnormal at 29°C. To investigate this in more detail, we immunostained the mutant ovaries with antibodies specific for Vasa, oo18 RNA‐binding protein (Orb), Fasciclin 3 (Fas3), and Spectrin (Spec), and with phalloidin, a chemical that specifically binds to filamentous actin (F‐actin). Vasa has been shown to be ectopically expressed in cultured OSCs upon L(3)mbt depletion and somatic cells in *L(3)mbt* ^*ts1*^ ovaries (Sumiyoshi *et al*, 2016; Coux *et al*, 2018). Similarly, Vasa was detected in somatic (follicle) cells in the *Lint‐O* ^*KO*^ ovaries at 29°C (Figs 5D and E and EV5A).

The expression of Orb in the *yellow white* (*y w*) ovaries that we employed as a WT control was restricted to the developing oocyte at the posterior of egg chambers (Fig EV5B), as reported previously (Lantz *et al*, 1994). However, in the ovaries of *Lint‐O* ^*KO*^, Orb was mislocalized to the anterior region of the egg chamber, where a few cells had gathered abnormally (Fig EV5B). Fas3 is a cell adhesion molecule abundant in polar cells during the late stages of oogenesis (Ruohola *et al*, 1991). The polar cells in *y w* localize to the edge of the anterior–posterior axis of the follicular cells (Fig EV5C). By contrast, the egg chamber of *Lint‐O* ^*KO*^ had excessive and ectopic polar cells. It seemed that ovarioles were fused during ovary morphogenesis in the absence of *lint‐O* expression. Spec localizes to somatic cell membranes and the spectrosome/fusome while the basic F‐actin polymer generates a dynamic cytoskeletal network (Theurkauf *et al*, 1993; Lin *et al*, 1994). Spec and F‐actin immunostaining signals indicated that the egg chambers were indeed fused in *Lint‐O* ^*KO*^ at restrictive temperatures (Fig EV5D and E). These findings indicate that Lint‐O plays an essential role in ovarian morphogenesis and oocyte development, similar to L(3)mbt (Coux *et al*, 2018).

When fly ovaries were immunostained with anti‐Lint‐O antibody, no signal was detected. Therefore, the gene encoding Venus was knocked in at the *lint‐O* genomic loci. Fluorescent signals were detected in both germ and somatic cells (Fig EV5F). It is not yet known whether the ovarian phenotype described above is due to loss of Lint‐O expression in germ cells, somatic cells, or both. We infer that somatic cell expression of Lint‐O may be important for the maintenance of ovarian morphology, similar to L(3)mbt (Coux *et al*, 2018).

### L(3)mbt and Lint‐O cooperatively control target genes in the brain to suppress tumorigenesis

**Figure 6:**
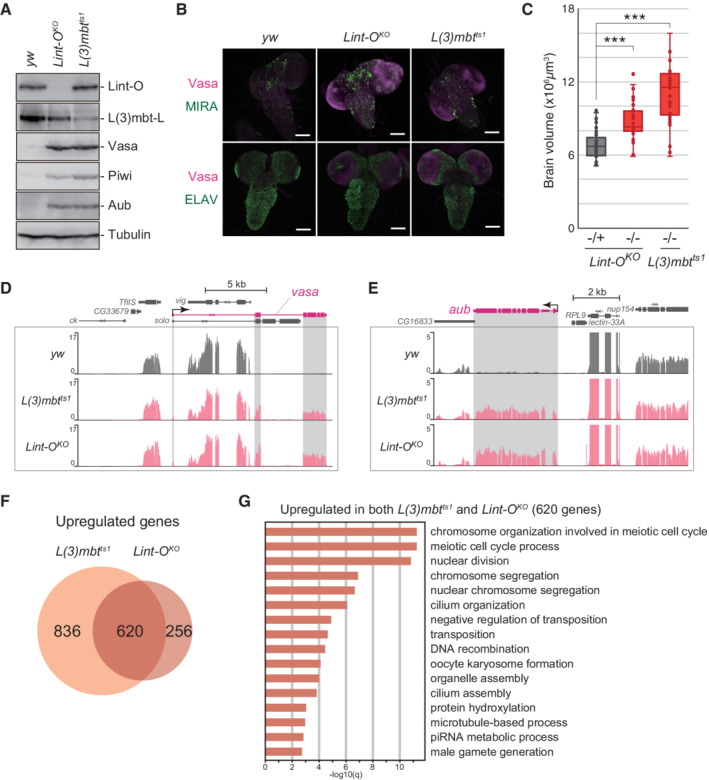
L(3)mbt and Lint‐O cooperatively control target genes in the brain A
Western blotting shows that Vasa, Piwi, and Aub were ectopically expressed in *Lint‐O* ^*KO*^ and *L(3)mbt* ^*ts1*^ larval brains at 29°C. Anti‐Lint‐O, anti‐L(3)mbt, anti‐Vasa, andi‐Piwi, anti‐Aub, and anti‐Tubulin antibodies were used.B
Confocal images of *y w*, *Lint‐O* ^*KO*^, and *L(3)mbt* ^*ts1*^ immunostained for Vasa (magenta), MIRA (green), and ELAV (green). Scale bar: 100 μm.C
Quantification of brain lobe volume for the following genotypes: *Lint‐O* ^*KO*^ −/+ (*n* = 42 biological replicates), *Lint‐O* ^*KO*^ −/− (*n* = 36 biological replicates), and *L(3)mbt* ^*ts1*^ −/− (*n* = 34 biological replicates). Boxplot central bands, upper edges of boxes, lower edges of boxes, upper whiskers and lower whiskers show median, third quartile, first quartile, maxima, and minima, respectively. *P* values were calculated by the Student's *t*‐test. (***P‐value < 0.001).D, E
The genomic regions harboring the *vasa* (D) and *aub* (E) genes. The RNA‐seq reads in *y w*, *L(3)mbt* ^*ts1*^, and *Lint‐O* ^*KO*^ brains are shown. The shading in gray corresponds to exons. The *y‐*axis shows the number of RPM. RNA‐seq samples were biological triplicates.F
Venn diagram shows that 620 protein‐coding genes are shared with the *l(3)mbt* and *lint‐O* libraries.G
High‐ranking GO terms for the 620 protein‐coding genes in (F). Source data are available online for this figure.

When L(3)mbt was depleted in the third‐stage (L3) larvae, germline genes such as *vasa*, *piwi*, and *aub* were ectopically expressed in the brain, resulting in tumorigenesis (Janic *et al*, 2010; Richter *et al*, 2011). Remarkably, western blotting showed that Vasa, Piwi, and Aub proteins were ectopically expressed in the brain of *Lint‐O* ^*KO*^ L3 larvae (Fig 6A). Immunofluorescence detected Vasa relatively strongly in Miranda (MIRA)‐positive neuroblast cells (Betschinger *et al*, 2006) but not in ELAV‐positive neuronal cells (Robinow & White, 1991) (Figs 6B and EV5G–J). Lint‐O may repress Vasa expression in undifferentiated cells but not in terminally differentiated cells. The *l(3)mbt* mutants ectopically expressed Vasa particularly in the outer proliferative center (OPC) and in the central brain (CB) neuroblasts (Janic *et al*, 2010; Richter *et al*, 2011). Similar cell specificity was observed for the *Lint‐O* ^*KO*^ L3 larval brain (Fig EV5G–J). In addition, the *Lint‐O* ^*KO*^ brain was enlarged, as was the *L(3)mbt* ^*ts1*^ brain, although it was slightly smaller (Fig 6C). These results support the intriguing concept that Lint‐O plays an important role in L(3)mbt‐mediated inhibition of tumorigenesis in the brain.

We next performed genome‐wide RNA‐seq in the L3 brains from *y w*, *L(3)mbt* ^*ts1*^, and *Lint‐O* ^*KO*^. The mapping of the RNA‐seq reads onto *vasa* and *aub* confirmed their derepression in *L(3)mbt* ^*ts1*^ and *Lint‐O* ^*KO*^ (Fig 6D and E). Computational analysis revealed that 620 genes were upregulated commonly in *L(3)mbt* ^*ts1*^ and *Lint‐O* ^*KO*^ (Fig 6F). GO analysis of these genes showed that the terms with high statistical values [*q*‐value (−log10) > 10] included “chromosome organization,” “meiotic cell cycle,” and “nuclear division.” The term of “piRNA metabolic process” also appeared [*q*‐value (−log 10) = 2.86] (Fig 6G). These results indicate that L(3)mbt/Lint‐O tends to regulate genes that function in specific intracellular pathways in the brain, such as the piRNA pathway. In agreement with this, not only the *vasa* and *aub* genes but also other germline‐specific piRNA factors (*e.g*., *piwi*, *spindle‐E*, *krimper*, *tej*, *sister of Yb*, *vreteno*, and *nxf2*) appeared in the gene set (GSE181802). On the other hand, no significant GO terms were found for the L(3)mbt‐dependent and Lint‐O‐independent genes (836 genes) and the L(3)mbt‐independent and Lint‐O‐dependent genes (256 genes). These findings suggest that L(3)mbt and Lint‐O may regulate some specific genes independently from each other, but they do not seem to be aimed at regulating any special pathways.

## Discussion

The loss‐of‐function mutations in the *Drosophila l(3)mbt* gene cause malignant tumor growth in the brain. This finding was first reported in 1982 (Gateff, 1982). Since then, a number of studies have been conducted to elucidate the function of L(3)mbt and to determine its causal relationship with tumorigenesis. Through this, multiple co‐factors, such as Lint‐1, CoRest, and Myb, were identified (Lewis *et al*, 2004; Georlette *et al*, 2007; Meier *et al*, 2012). However, until the present study, Lint‐O had never been reported to be an L(3)mbt co‐suppressor, showing the complexity of the L(3)mbt‐mediated gene regulatory pathway. What was the key leading to the discovery of Lint‐O in this study? One plausible answer is the use of OSCs, a cultured cell line composed solely of somatic (follicular) cells derived from *Drosophila* ovary, not from the brain.

Upon the lack of L(3)mbt, OSCs aberrantly induced the expression of germ‐specific genes including piRNA amplifiers and activated the ping‐pong pathway, in which piRNAs innately generated in naïve OSCs were actually amplified (Sumiyoshi *et al*, 2016). The *Drosophila* brain autonomously initiated piRNA biogenesis upon the loss of *l(3)mbt* function (Janic *et al*, 2010). Strikingly, when piRNA biogenesis was forcibly attenuated, the tumorigenesis ceased. We previously attempted L(3)mbt knockdown in nongonadal somatic Schneider 2 (S2) cells to examine whether it activated piRNA biogenesis. However, piRNA biogenesis was not carried out because necessary factors were not sufficiently activated. This implies that OSCs and the brain, particularly CB neuroblasts that became Vasa‐positive upon *l(3)mbt* deficiency (Janic *et al*, 2010; Richter *et al*, 2011; this study), have some commonality, but S2 cells do not. At present, it is unclear what this commonality consists of materially, besides the two main players L(3)mbt and Lint‐O. However, the susceptibility of both cells to the loss of function of L(3)mbt and Lint‐O is likely to be almost identical. In other words, the degree and mechanism of cooperative gene regulation between L(3)mbt and Lint‐O, and the balance with other gene regulatory means, are considered to be equivalent in the two cases. If there was a CB‐derived cultured cell line, we could carry out similar experiments in both cells in parallel and compare the outcomes to obtain an understanding of the commonality. However, no such cell line appears to be available in the field of *Drosophila* research.

In this study, by performing genome‐wide ChIP‐seq and RNA‐seq in parallel in OSCs, we were able to understand the implications of the genomic binding of L(3)mbt, such as which binding is actually responsible for the expression of the gene targets. Similar assays can be conducted using the ovary, for example, but its tissue is composed of germarium and egg chambers at different stages of development. Egg chambers are a mixture of nurse cells and oocytes, with follicular cells surrounding them. The follicular cells normally lack Vasa and Aub, and only in abnormal situations such as the depletion of L(3)mbt and/or Lint‐O, these proteins are aberrantly upregulated. The nurse cells express Vasa irrespective of the presence or absence of L(3)mbt or Lint‐O. With this complexity, it would not be easy to interpret the experimental results accurately and obtain a correct understanding. This reminds us of the usefulness of *in vivo* and *ex vivo* systems and the importance of combining the two.

Lint‐1 was present in the L(3)mbt interactors in OSCs. This led us to speculate that L(3)mbt interacts with not only Lint‐O but also Lint‐1 to assemble a highly ordered complex to regulate the target genes in a cooperative manner. However, the *lint‐O* brain was tumorigenic, similar to the *l(3)mbt* brain (but it showed slightly less brain enlargement). This implies that L(3)mbt and Lint‐1, likely as the LINT complex with CoRest, contribute less to the regulation of germline genes in the brain. The situation could be similar in OSCs. Another plausible idea is that, in the absence of Lint‐O, Lint‐1 may become unstable as Lint‐O became unstable in the absence of L(3)mbt. Given our present understanding, this is not an unreasonable proposition, but further research is required to properly understand the relationship among these three proteins.

The *l(3)mbt* gene is conserved from nematodes to humans, although the domain structures may not be strictly identical among them (Bonasio *et al*, 2010). Humans express four L(3)mbt orthologs, L3MBTL1 to L3MBTL4. Of these, L3MBTL1, L3MBTL3, and L3MBTL4 contain three MBT repeats, a zinc finger, and a SAM domain, similar to *Drosophila* L(3)mbt, but L3MBTL2 contains four MBT repeats and a zinc finger but lacks the SAM domain (Boccuni *et al*, 2003; Bateman, 2019). According to their peptide sequences, the protein sizes of human L3MBTLs are approximately half that of the fly L(3)mbt (Boccuni *et al*, 2003; Bateman, 2019). These interspecies differences suggest that L(3)mbt orthologs may exert their function(s) in different ways in individual organisms.

Are there any human orthologs of Lint‐O? Previous proteomics analysis identified the sterile alpha motif domain containing 13 (SAMD13) as an interactor of human L(3)MBTL3 (Hauri *et al*, 2016; Huttlin *et al*, 2017). SAMD13 has a SAM domain but lacks the PHD domain that Lint‐O has, but it is recognized as a human ortholog of Lint‐O based on a BLAST search. Although this study did not go so far as to remark the function of the PHD domain of Lint‐O, we found that the PHD domain is required for the functional expression of Lint‐O. In this regard, SAMD13 may not be the human version of Lint‐O. One possibility is that factors that bind to SAMD13 may have a PHD domain or something similar. However, this is also a matter of conjecture, and further research is needed before any conclusions are drawn. It is still unclear whether loss of L3MBTLs causes brain tumors or infertility in humans. It is also unclear whether loss of SAMD13 causes serious damage to the human brain and germline. Future research results on these interesting issues are awaited.

Previous studies showed that L(3)mbt binds to insulator elements of several insulator factors such as CP190, CTCF, and BEAF‐32 (Richter *et al*, 2011; Bortle *et al*, 2012). Although these factors did not appear as interacting factors for L(3)mbt in OSCs in this study (Fig 2E), it is possible that L(3)mbt and Lint‐O regulate gene expression through higher chromatin organization in addition to binding to the gene body. *ago3* may be one of the genes regulated by chromatin organization.

## Materials and Methods

### Cell culture

OSCs (Saito *et al*, 2009; RRID:CVCL_IY73) were cultured at 26°C in Shields and Sang M3 Insect Medium (US Biological) supplemented with 10% fly extract (Saito *et al*, 2009), 10% fetal bovine serum (Funakoshi), 10 mU/ml insulin, and 0.6 mg/ml glutathione.

### Plasmid construction

To construct L(3)mbt‐L‐3xFLAG, L(3)mbt‐S‐3xFLAG, myc‐Lint‐O‐ and Lint‐O‐3xFLAG‐expressing plasmids, a full‐length L(3)mbt‐L/‐S/Lint‐O cDNA was amplified from cDNA library produced from total RNAs of OSCs using First Strand cDNA Synthesis Kit (Roche). The 5′‐end of L(3)mbt‐L/S was determined by 5′ RACE using the SMARTer RACE 5′/3′ Kit (Clontech). Then, amplified cDNA was cloned into vectors that have the promoter of actin and myc/3xFLAG tags using NEBuilder HiFi DNA Assembly Cloning Kit (NEB). For the rescue assays, Lint‐O‐3xFLAG expressing plasmids were modified to be siRNA‐resistant using inverse PCR and infusion. The ∆SAM and ∆PHD mutants were produced by inverse PCR on L(3)mbt‐L/S‐3xFLAG and Lint‐O‐3xFLAG (siRNA‐resistant) and ligation. The Lint‐O‐8CA mutant was produced by mutagenesis using inverse PCR on Lint‐O‐3xFLAG (siRNA‐resistant) and ligation. Primers used are summarized in Appendix Table S2.

### RNAi and plasmid transfection

For performing RNAi, up to 6 × 10^6^ trypsinized OSCs were prepared and suspended in 20 μl of Solution SF from the Cell Line Nucleofector Kit SF (Lonza Bioscience) to which 400 pmol siRNA duplex was added. Transfection was performed with the Nucleofector 96‐well Shuttle Device (Lonza Bioscience). For performing transfection of the plasmids along with siRNAs, up to 1 × 10^7^ trypsinized OSCs were prepared and suspended in buffer [180 mM sodium phosphate buffer for Church and Gilbert hybridization (pH 7.2) containing 15 mM MgCl_2_, 5 mM KCl, and 50 mM D‐mannitol], modified from the original protocol (Nye *et al*, 2014). A total of 6 μg of the plasmids and 600 pmol of the siRNA duplex were added to the suspended cells, and transfection was performed with the Nucleofector 2b Device (Lonza Bioscience) using the N‐020 program. Transfected cells were cultured at 26°C in a fresh OSC medium for 48 h for further experiments. For the rescue assays, RNAi‐treated cells were incubated for 48 h before co‐transfection of the plasmids and siRNA duplex. After co‐transfection, the cells were cultured for another 48 h. The sequences of siRNA duplexes are listed in Appendix Table S2.

### Production of antibodies

The recombinant L(3)mbt peptide (Lys401‐Thr600) tagged with glutathione S‐transferase [GST‐L(3)mbt] was purified from *E. coli* and used for mouse immunization. The production and selection of hybridomas were performed as described in a previous report (Nishida *et al*, 2015). To produce the anti‐Lint‐O antibody, a part of the protein (Gln340‐Ser353) was used to immunize rabbits. The antibody was purified from the serum and then dialyzed against PBS. The Lint‐O peptide and antibody were prepared by Eurofins Scientific. A Lint‐O peptide (Met1‐Arg130) fused to 6 x His was expressed in and purified from *E. coli*, and then injected into 8‐week‐old female Tsl:BALB/cCr[GF] mice (Sankyo Lab) for immunization. The blood serum was used for experiments using flies. Mouse procedures were approved by the Animal Research Committee of the University of Tokyo (Animal plan16‐3).

### Nuclear extraction and immunoprecipitation

Nuclear lysates were prepared and immunoprecipitation was performed as described previously (Onishi *et al*, 2020). Briefly, cells were suspended in hypotonic buffer [10 mM HEPES‐KOH (pH 7.3), 10 mM KCl, 1.5 mM MgCl_2_, 0.5 mM dithiothreitol (DTT), 2 μg/ml pepstatin A, 2 μg/ml leupeptin, and 0.5% aprotinin], and the cells were fractured by passing them three times through a 25‐gauge needle. The nuclear fraction was first centrifuged at 400 *g* for 10 min, washed with hypotonic buffer by centrifugation at 13,600 *g* for 20 min, and resuspended in chromatin co‐immunoprecipitation (co‐IP) buffer [50 mM HEPES‐KOH (pH 7.3), 200 mM KCl, 1 mM EDTA, 1% Triton X‐100, 0.1% sodium deoxycholate, 2 μg/ml pepstatin A, 2 μg/ml leupeptin, and 0.5% aprotinin]. The nuclear fraction was sonicated on ice using a Branson Digital Sonifier and then centrifuged at 20,000 *g* for 20 min at 4°C. The supernatant was collected as the nuclear extract. For immunoprecipitation, the nuclear lysate was incubated with antibodies bound to Dynabeads Protein G (Thermo Fisher Scientific) at 4°C for 2 h. The beads were washed five times with co‐IP buffer. Proteins were eluted from the beads by heating at 95°C for 3 min in buffer containing SDS. After electrophoresis, the samples for LC–MS/MS were visualized by silver staining using SilverQuest (Invitrogen) or processed for western blotting.

### Western blotting

Western blotting was performed as described previously (Miyoshi *et al*, 2005). For primary antibodies, anti‐L(3)mbt (culture supernatant from the hybridoma) (this study), anti‐Lint‐O (rabbit blood serum, 1:10 dilution) (this study), anti‐FLAG (SIGMA M2, 1:5,000 dilution), anti‐Vasa (8E12, purified, 1:1,000 dilution) (Nishida *et al*, 2015), anti‐Piwi (4D2 cultured supernatant) (Saito *et al*, 2006), anti‐Aub (4D10, purified, 1:1,000 dilution) (Nishida *et al*, 2015), anti‐Tubulin [E7 culture supernatant, Developmental Studies Hybridoma Bank (DSHB)], anti‐histone H3 (Abcam ca#ab1791, 1:2,000 dilution), and anti‐Myc monoclonal (Fujifilm Wako Pure Chemical 9E10, 1:1,000 dilution) antibodies were used.

### Immunofluorescence

Immunofluorescence was performed as described previously (Saito *et al*, 2010). The primary antibodies used in this study were anti‐L(3)mbt (1:500 dilution) and anti‐FLAG monoclonal (MBL FLA‐1, 1:1,000 dilution, and SIGMA M2, 1:1,000 dilution) antibodies. The secondary antibodies used were Alexa 488‐conjugated anti‐mouse antibody (Thermo Fisher Scientific, 1:500 dilution). The cells were mounted with the VECTASHIELD Mounting Medium and stained with DAPI (Vector Laboratories), followed by observation using an LSM 710 or LSM 980 laser scanning confocal microscope (Carl Zeiss). Immunostaining of ovaries was performed following standard procedures. Fixation of ovaries was carried out with 4% paraformaldehyde phosphate buffer. Mouse anti‐Vasa (1:500 dilution) (Nishida *et al*, 2015), mouse anti‐Spectrin (1 μg/ml, 3A9, DSHB), mouse anti‐Fas3 (1 μg/ml, 7G10, DSHB), and mouse anti‐Orb (1 μg/ml, 4H8, DSHB) antibodies were used as primary antibodies. The specificity of the staining for F‐actin was verified by Phalloidin‐iFluor488 (1:1,000 dilution, #23115, AAT Bioquest). Larval brains were dissected in cold PBS and fixed in 4% paraformaldehyde for 40 min. Samples were incubated with primary antibodies diluted in PBS containing 0.3% Triton X‐100 and 5% BSA (PBSTB) overnight at 4°C. Primary antibodies used were as follows: mouse anti‐Vasa (8E12, 1:50 dilution) (Nishida *et al*, 2015), rat anti‐MIRA (1:500 dilution, ab197788, Abcam), and rat anti‐ELAV (1:100 dilution, 7E8A10, DSHB). Brains were washed three times for 10 min in PBST and then incubated in secondary antibodies overnight at 4°C. Secondary antibodies were used as follows: Alexa 546‐conjugated anti‐mouse IgG (1:1,000 dilution, Molecular Probes) or Alexa 488‐conjugated anti‐rat IgG (1:1,000 dilution, Molecular Probes). Samples were incubated with secondary antibodies overnight at 4°C. After washing three times for 10 min in PBST, samples were mounted in the VECTASHIELD Mounting Medium and stained with DAPI (Vector Laboratories). Images of ovaries and larval brains were collected using a confocal microscope (Zeiss LSM900).

### RNA extraction and RT–qPCR

Total RNAs were extracted from OSCs with the ISOGEN II reagent (Nippon Gene). After digestion of the contaminated DNAs by DNase (Life Technologies) and subsequent purification, a cDNA library was prepared using the reverse‐transcription kit ReverTra Ace (Toyobo). For RT–qPCR, cDNAs or DNA products from ChIP were amplified using the StepOnePlus Real‐Time PCR System (Applied Biosystems) with PCR enzymes and the PowerUp SYBR Green Master Mix (Thermo Fisher Scientific). The efficiency of qPCR amplification was calculated based on the slope of the standard curve. After determining the amplification efficiency values (between 95% and 105%), the relative steady‐state RNA levels were calculated from the threshold cycle for amplification. The sequences of primers are listed in Appendix Table S2.

### Preparation of protein samples for LC–MS/MS

For the preparation of immunoprecipitated samples for LC–MS/MS, the anti‐L(3)mbt antibody used for immunoprecipitation was cross‐linked to beads by dimethyl pimelimidate (Thermo Fisher Scientific). Immunoprecipitation was performed using control OSCs and L(3)mbt knockdown (KD) OSCs. The cells were washed after incubation six times with 800 mM NaCl in HEMG buffer [25 mM HEPES‐KOH (pH 7.3), 12.5 mM MgCl_2_, 0.1% Nonidet P‐40, 0.1 mM EDTA, 20% glycerol, 2 μg/ml pepstatin A, 2 μg/ml leupeptin, and 0.5% aprotinin] and then twice with 200 mM NaCl in HEMG buffer. The immunoprecipitants were eluted in elution buffer containing 125 mM Tris–HCl (pH 6.8), 4% SDS, and 0.01% bromophenol blue by heating at 70°C for 10 min. The products were precipitated in acetone containing 20% trichloroacetic acid. The same experiment was performed again to generate a replicate. The pellets containing immunoprecipitated samples were lysed in 100 μl of PTS buffer [100 mM NH_4_HCO_3_, 12 mM sodium deoxycholate, 12 mM sodium N‐lauroylsarcosinate, and phosphatase inhibitor cocktails 2 and 3 (Sigma‐Aldrich)]. Each sample in the PTS buffer was reduced with 10 mM DTT at 60°C for 30 min and then alkylated by incubation with 22 mM iodoacetamide at 37°C for 30 min in the dark. Next, the samples were diluted with 100 mM NH_4_HCO_3_ solution up to a volume of 500 μl and digested with 0.4 μg of trypsin (Roche) by incubation at 37°C for 18 h in the dark. After the digestion, an equal volume of ethyl acetate was added to the samples, and the mixture was acidified with 0.5% TFA and mixed to transfer the detergents into the organic phase. After the samples were centrifuged at 15,700 *g* for 1 min at room temperature, the aqueous phases containing the peptides were collected. The samples were concentrated by a centrifugal evaporator (Eyela) and desalted using a MonoSpin C18 column (GL Sciences). The eluted products were dried prior to LC–MS/MS analysis.

### LC–MS/MS analysis

The dried and desalted peptides were dissolved in distilled water containing 2% acetonitrile and 0.1% TFA. The LC–MS/MS analyses were performed using a mass spectrometer (Q Exactive Plus, Thermo Fisher Scientific) equipped with a nano ultra‐high‐performance liquid chromatography system (Dionex Ultimate 3000, Thermo Fisher Scientific). The peptides were loaded onto the LC–MS/MS system with a trap column (0.3 × 5 mm, L‐column, octadecylsilyl groups; Chemicals Evaluation and Research Institute) and a capillary column (0.1 × 150 mm, L‐column, octadecylsilyl groups, Chemicals Evaluation and Research Institute) at a flow rate of 10 μl/min. The loaded peptides were separated by a gradient using the mobile phases A (1% formic acid in distilled water) and B (1% formic acid in acetonitrile) at a flow rate of 300 nl/min (2% to 35% B in 133 min, 35% to 50% B in 20 min, 50% to 95% B in 2 min, 95% B for 10 min, 95% to 2% B in 0.1 min and 2% B for 10 min). The eluted peptides were electrosprayed (2.0 kV) and introduced into the MS equipment (positive ion mode, data‐dependent MS/MS). Each of the most intense precursor ions (the top 10) was isolated and fragmented by higher collision energy dissociation with normalized collision energy (27%). For full MS scans, the scan range was set to 350–1,500 *m/z* at a resolution of 70,000, and the AGC target was set to 3e6 with a maximum injection time of 60 ms. For the MS/MS scans, the precursor isolation window was set to 1.6 *m/z* at a resolution of 17,500 and the AGC target was set to 5e5 with a maximum injection time of 60 ms. The Orbitrap mass analyzer was operated with the “lock mass” option to perform shotgun detection with high accuracy. The raw spectra were extracted using Proteome Discoverer 2.2 (Thermo Fisher Scientific) and searched against the *Drosophila* UniProt database (TaxID = 7,227 and subtaxonomies) with the following settings: the parameter for the cleavage was set to trypsin, and a maximum missed number of cleavages of two were allowed. The mass tolerances were set to 10 ppm for the precursor ion and 0.02 Da for the fragment ion. As for protein modifications, we set carbamidomethylation (+ 57.021 Da) at Cys as a fixed modification of the peptide, oxidation (+ 15.995 Da) at Met as a dynamic (nonfixed) modification of the peptide, and acetylation (+ 42.011 Da) at the N‐terminus as a dynamic modification of the protein terminus. The amount of each peptide was semi‐quantified using the peak area with Precursor Ions Quantifier in Proteome Discoverer 2.2.

### ChIP and ChIP‐seq library preparation

For chromatin immunoprecipitation, cultured OSCs (1 × 10^7^ cells/reaction) were fixed by incubation with an OSC medium including 0.75% formaldehyde for 5 min at room temperature, following which glycine was added to the medium (final concentration, 125 mM) to stop the fixation. Cells were scraped and suspended with hypotonic buffer [25 mM HEPES‐KOH (pH 7.3), 1.5 mM MgCl_2_, 10 mM KCl, 1 mM DTT, 0.1% NP‐40, 2 μg/ml pepstatin A, 2 μg/ml leupeptin, and 0.5% aprotinin] and the nuclei were centrifuged into pellets at 1,000 *g* for 10 min. The nuclei were diluted in sonication buffer [50 mM HEPES‐KOH (pH 7.3), 140 mM NaCl, 1% Triton X‐100, 0.1% sodium deoxycholate, 0.1% SDS, 1 mM EDTA, 2 μg/ml pepstatin A, 2 μg/ml leupeptin, and 0.5% aprotinin] and sonicated with a Covaris S220 Focused ultrasonicator for 10 min at 4°C. The settings used for the sonication were as follows: peak power 140, duty factor 5.0, and 200 cycle/burst. Next, the lysates were centrifuged at 20,000 *g* for 20 min, and the supernatants were collected for immunoprecipitation. Anti‐L(3)mbt and anti‐Lint‐O antibodies were added to the supernatants and incubated for 16 h at 4°C. Dynabeads Protein G (Thermo Fisher Scientific) was added to each sample and incubated for 1 h at 4°C. After incubation, beads were washed once with low‐salt buffer [20 mM Tris–HCl (pH 8.0), 150 mM NaCl, 2 mM EDTA, 0.1% Triton X‐100, 0.1% SDS, and 1 mM phenylmethylsulfonyl fluoride (PMSF)], high‐salt buffer [20 mM Tris–HCl (pH 8.0), 500 mM NaCl, 2 mM EDTA, 0.1% Triton X‐100, 0.1% SDS, and 1 mM PMSF], and LiCl buffer [20 mM Tris–HCl (pH 8.0), 200 mM LiCl, 2 mM EDTA, 1% NP‐40, 1% sodium deoxycholate, and 1 mM PMSF], and twice with TE buffer [20 mM Tris–HCl (pH 8.0), 1 mM EDTA, and 1 mM PMSF]. The beads were dissolved in elution buffer [50 mM Tris–HCl (pH 8.0), 10 mM EDTA, and 1% SDS] and incubated at 65°C for 30 min. The supernatant containing the immunoprecipitated materials was collected, to which NaCl was added to a final concentration of 200 mM, and the supernatant was incubated at 65°C for 8 h for decrosslinking. Next, 2 μl of RNase A (10 mg/ml) was added to the samples and incubated at 37°C for 30 min to digest the RNAs, and 5 μl of proteinase K (20 mg/ml) was added and incubated at 55°C for 1 h to digest the proteins. The DNA was purified with the FastGene Gel/PCR Extraction Kit (Nippon Genetics), in accordance with the manufacturer's protocol and eluted in nuclease‐free water. DNA products from ChIP were amplified using the StepOnePlus Real‐Time PCR System (Applied Biosystems) with PCR enzymes and the PowerUp SYBR Green Master Mix (Thermo Fisher Scientific) and qualified for ChIP‐seq libraries. ChIP‐seq libraries were prepared in accordance with the instructions accompanying the NEBNext Ultra II FS DNA Library Prep Kit for Illumina (New England BioLabs). In the adaptor ligation step, the original adaptor reagent was diluted 10‐fold. After adapter ligation, the DNA was purified with AMPure XP beads (Beckman Coulter) for size selection. Then, the DNA was PCR‐amplified with Illumina primers for 13–16 cycles and the library fragments of ~ 325 bp (insert plus adaptor and the PCR primer sequences) were purified with AMPure XP beads (Beckman Coulter). Duplicate samples were prepared for each condition. The purified DNA was captured on an Illumina flow cell for cluster generation. The libraries were sequenced on the Illumina HiSeq in accordance with the manufacturer's protocols. The ratio of the abundances of the peptides [control/L(3)mbt KD > 4] and the peptide spectrum matches (> 1) were used for candidate selection.

### RNA‐seq library preparation

The ribosomal RNAs were excluded from the total RNAs using a Ribo‐Zero rRNA Removal Kit (Human/Mouse/Rat), in accordance with Illumina's protocol, and the RNA‐seq libraries were prepared following the instructions accompanying the TruSeq Stranded mRNA Library Prep Kit (Illumina). Poly‐A selected mRNAs were submitted for library preparation. Triplicate samples were prepared for each RNAi‐treated condition. Libraries were sequenced on the Illumina HiSeq following the manufacturer's protocol.

### Computational analysis of RNA‐seq and ChIP‐seq

For ChIP‐seq analysis, the sequence reads were mapped to the genome of *Drosophila melanogaster* (dm6) using Bowtie 2 (Langmead & Salzberg, 2012). Only unique mapped reads were used in the analysis and PCR amplicons were excluded using Picard tools (https://broadinstitute.github.io/picard/). Peak calling was completed with MACS2 (Zhang *et al*, 2008) with default settings. From the output file of peak coordinate, the peaks with log10‐converted *q*‐value over −100 were removed. Pearson's correlation coefficient between L(3)mbt ChIP duplicate reads mapped on merged peaks was 0.983 and that between Lint‐O ChIP duplicate reads mapped on merged peaks was 0.981. Common peaks, not merged peaks, between the duplicate samples were selected and used for further analysis. Peaks were annotated with in‐house scripts according to dmel‐all‐r6.43.gtf (ftp://ftp.flybase.org/genomes/dmel/current/gtf/). For heatmaps and summary plots, ChIP scores were calculated by deeptools (Ramírez *et al*, 2016). Bigwig files of ChIP were produced by the bamCoverage program of deeptools and matrix data were calculated by the computeMatrix program of deeptools using bigwig files and plotted. Motif detection was performed using the peak bed files produced by MACS2 and findMotifsGenome.pl program of Homer (Heinz *et al*, 2010). For RNA‐seq analysis, sequence reads mapped to rRNAs were excluded and the remaining reads were mapped to the genome of *Drosophila melanogaster* (dm6) using HISAT2 (Kim *et al*, 2015). Differential expression analysis was performed using the triplicate samples and DESeq2 package in R (Love *et al*, 2014) The resulting lists of differentially expressed genes [FDR (false discovery rate) < 0.01] were categorized into upregulated and downregulated genes by the fold change of the expression and applied for further analysis (Appendix Table S3). PCA analysis of RNA‐seq samples of OSC was performed in R by using the dataset of reads for each gene normalized by million mapped reads. GO analysis of the biological process shown in Fig 6G was performed using clusterProfiler package in R (Yu *et al*, 2012; Wu *et al*, 2021).

### Fly stock

The *yellow white* (*y^1^ w^1118^*) strain was used as a WT strain. The mutants used in this study were: *y^1^ w^1118^*, *y^1^ w^1118^ Lint‐O^KO^/FM7*, *Kr > GFP* (this study), w*; L(3)mbt^ts1^/TM6b (gift from C. Gonzalez). A mutant allele of *Lint‐O^KO^* was generated using the transgenic CRISPR‐Cas9 technique as previously described (Kondo & Ueda, 2013) The following strains were used for the mutagenesis: *y^1^ v^1^ nos‐phiC31; attP40* host (NIG‐FLY stock TBX‐0002), *y^2^ cho^2^ v^1^; Sp hs‐hid/CyO* (NIG‐FLY stock TBX‐0009), *y^1^ w^1118^*; +; *attP2{nos‐cas9}* (Kondo *et al*, 2020), and *Df(1)JA27/FM7c*, *P{w[+mC]=GAL4‐Kr.C}DC1*, *P{w[+mC]=UAS‐GFP S65T}DC5*, *sn[+]* (Bloomington Drosophila Stock Center #5193). Mutant files were validated by PCR and sequencing of the target region. Oligo DNA sequences are shown in Appendix Table S2. Genotypes of flies used in this study are shown in Appendix Table S4.

### RNA extraction from flies and RT–qPCR

Total RNAs were extracted from ovaries using ISOGEN (Nippon Gene). RT–qPCR was performed as reported previously (Saito *et al*, 2009). In accordance with the manufacturer's instructions, 1 μg of total RNA from each sample was used to reverse‐transcribe target sequences using a Transcriptor First Strand cDNA Synthesis Kit (Roche). The resulting cDNAs were amplified with a TB Green Premix Ex Taq II (Takara). The primers used are shown in Appendix Table S2.

### Generation of the Lint‐O‐Venus strain

The *Lint‐O‐Venus* strain was generated by CRISPR/Cas9‐mediated targeted transgene integration (Kondo *et al*, 2020). The gene encoding Venus fluorescent protein was inserted immediately in front of the stop codon of the *Lint‐O* gene so that Lint‐O‐Venus was translated as a C‐terminal fusion protein. The donor vector (pBS‐Lint‐Oarm‐Venus‐3xP3‐dsRed‐Express2) carried approximately 800 bp and 500 bp homology arms on the left and left sides of a knock‐in cassette comprising genes encoding Venus and 3xP3‐dsRed‐Express2 flanked by loxP sites from pPV‐RF3 (Kondo *et al*, 2020). The donor and gRNA vector (pBFv‐U6.2‐Lint‐O‐gRNA5) containing a guide RNA were co‐injected into fertilized eggs laid by *nos*‐Cas9 flies. Transformants were selected by eye‐specific red fluorescence of the 3xP3‐dsRed‐Express2 transgene. Homozygous Lint‐O‐Venus strain was used for the microscopy analysis. DNA oligo sequences for plasmid vectors are shown in Appendix Table S2.

### Female fertility assay

Adults after eclosion were immediately transferred to 29°C and incubated for 2 days. Single females were then incubated with three *y* ^*1*^ *w* ^*1118*^ control males at 29°C for 5 days for mating. Ten independent crosses were performed. The number of adult offspring per cross was counted.

### L3 larval brain

L3 larval brains were isolated from *y* ^*1*^ *w* ^*1118*^, *y* ^*1*^ *w* ^*1118*^ *Lint‐O* ^*KO*^ */FM7 Kr > GFP*, *y* ^*1*^ *w* ^*1118*^ *Lint‐O* ^*KO*^, and *L(3)mbt* ^*ts1*^ grown at 29°C for 5–6 days after spawning at 25°C, and used for western blotting, volume calculation, immunostaining, and RNA‐seq analysis. The orthogonal major and minor axes across the brain lobes were measured using an SZX16 stereo microscope and cellSens standard (Olympus). The volume (*V*) of brain lobes was calculated as *V* = *4/3πab* ^*2*^, where *a* is the major semiaxis and *b* is the minor semiaxis. Significance was calculated by the Student's *t*‐test.

## Author contributions

**Hitomi Yamamoto‐Matsuda:** Data curation; investigation; writing – original draft; writing – review and editing. **Keita Miyoshi:** Funding acquisition; investigation; writing – review and editing. **Mai Moritoh:** Investigation; writing – review and editing. **Hikari Yoshitane:** Funding acquisition; investigation; methodology. **Yoshitaka Fukada:** Funding acquisition; methodology. **Kuniaki Saito:** Conceptualization; supervision; funding acquisition; writing – review and editing. **Soichiro Yamanaka:** Data curation; supervision; investigation; writing – original draft; writing – review and editing. **Mikiko, C Siomi:** Conceptualization; supervision; funding acquisition; writing – original draft; writing – review and editing.

In addition to the CRediT author contributions listed above, the contributions in detail are:

HY‐M and MM performed biochemical experiments. HY‐M and SY performed bioinformatic analysis. HY and YF performed mass spectrometric analysis. KM and KS produced *lint‐O* mutant flies and analyzed them. HY‐M, KM, KS, SY, and MCS conceived the project and designed the experiments. All authors contributed to the writing of the manuscript.

## Disclosure and competing interest statement

The authors declare that they have no conflict of interest.

## Supporting information



Appendix S1

Expanded View Figures PDF

Source Data for Expanded View

PDF+

Source Data for Figure 2

Source Data for Figure 3

Source Data for Figure 6

## Data Availability

OSC RNA‐seq and ChIP‐seq data are deposited at Gene Expression Omnibus (GSE) (GSE181802: https://www.ncbi.nlm.nih.gov/geo/query/acc.cgi?acc=GSE181802). Fly RNA‐seq data are also available at GSE (GSE205541: https://www.ncbi.nlm.nih.gov/geo/query/acc.cgi?acc=GSE205541). LC–MS/MS data are deposited at PRIDE (PXD026945: http://proteomecentral.proteomexchange.org/cgi/GetDataset?ID=PXD026945).
