# Supplementary material for: Lint‐O cooperates with L(3)mbt in target gene suppression to maintain homeostasis in fly ovary and brain
Source: EMBO Rep. 2022 Aug 22;23(10):e53813. doi: 10.15252/embr.202153813 (PMC9535798; doi:10.15252/embr.202153813)
Supplement: Supplementary file 2 — Expanded View Figures PDF [file EMBR-23-e53813-s002.pdf]

Expanded View Figures

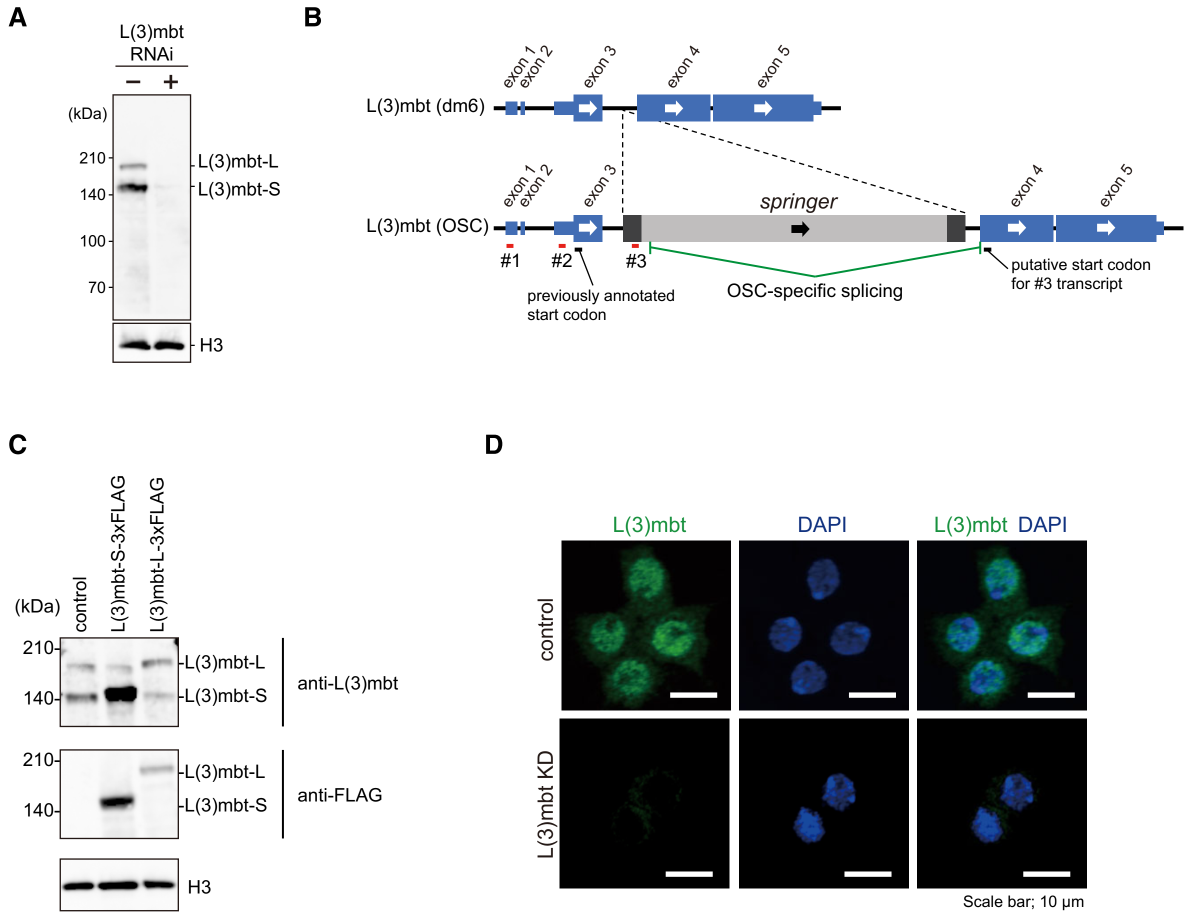

Figure EV1. Two isoforms of L(3)mbt, L(3)mbt-L, and L(3)mbt-S, in OSCs.

A Western blotting using an anti-L(3)mbt antibody that we raised in this study detected L(3)mbt as a doublet in the OSC nuclear lysates. The doublet disappeared upon L(3)mbt RNAi. Histone H3 (H3) served as a loading control.

B The exon-intron structures of the *l(3)mbt* gene in dm6 (provided by UCSC) and in OSCs (Sienski *et al*, 2012). Note that the *l(3)mbt* gene in OSCs has an LTR-type transposon, *springer*. RACE detected three different 5′ ends of *l(3)mbt* mRNA (#1, #2, and #3).

C Top: Western blotting using the anti-L(3)mbt antibody shows that L(3)mbt-L-3xFLAG and L(3)mbt-S-3xFLAG exogenously expressed in OSCs co-migrate with endogenous L(3)mbt-L and L(3)mbt-S (control), respectively. Middle: Western blotting was performed using anti-FLAG antibody. Bottom: Histone H3 (H3) was detected as a loading control.

D Immunofluorescence analysis using the anti-L(3)mbt antibody detected L(3)mbt (green) mostly in the OSC nuclei. The L(3)mbt signals disappeared upon L(3)mbt RNAi (KD). DAPI (blue) indicates the nuclei. Scale bar: 10 µm.

Source data are available online for this figure.

Figure EV2. Genomic distribution of L(3)mbt and the effects of L(3)mbt loss on the expression levels of piRNA factors in OSCs.

A Genomic browser views of L(3)mbt ChIP-seq signals. All fly chromosomes are shown. The *vasa*, *ago3*, and *aub* loci are indicated. The total number of L(3)mbt ChIP peaks is shown in upper right.

B PCA shows the correlation in the RNA-seq libraries. Control: GFP siRNA was used.

C–E The genomic regions harboring *qin*, *tej*, *boot*, and *CG9925* (C), *piwi*, *yb*, *armi*, and *zuc* (D), and *flam* (E).

▶

© 2022 The Authors

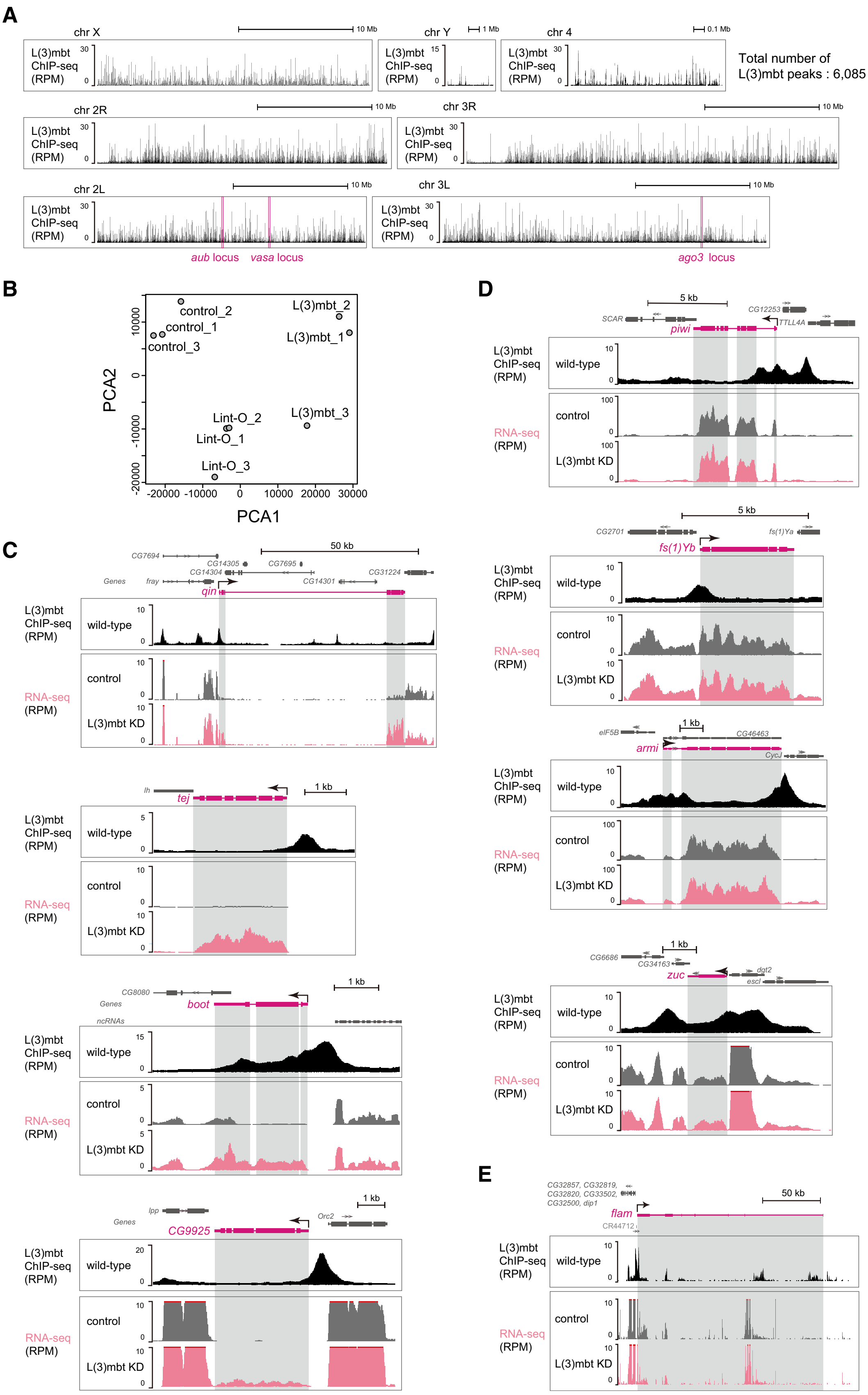

Figure EV2.

© 2022 The Authors

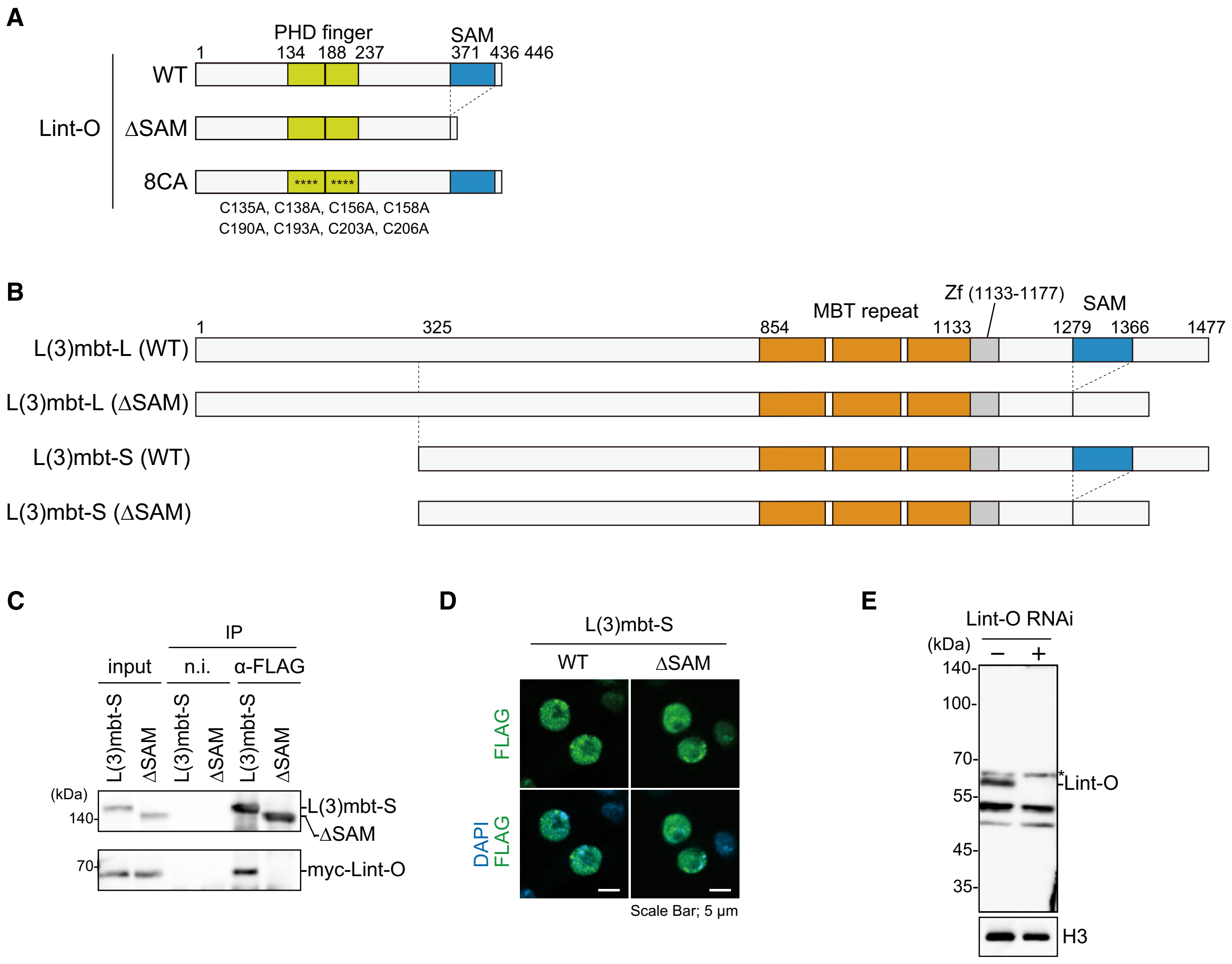

Figure EV3. Doman structures of L(3)mbt, Lint-O and their mutants and behavior of L(3)mbt-S in OSCs.

A Domain structures of WT Lint-O and its ΔSAM and 8CA mutants. The ΔSAM mutant is composed of Met1-Val371 and Ser437-Asp446. The Cys-to-Ala mutations in the 8CA mutant are indicated at the bottom (asterisks in the structure).

B Domain structures of WT L(3)mbt-L, WT L(3)mbt-S, and their ΔSAM mutants. The ΔSAM mutant of L(3)mbt-L is composed of Met1-Leu1278 and Val1367-Ser1477 of WT L(3)mbt-L. The ΔSAM mutant of L(3)mbt-S is composed of Met325-Leu1278 and Val1367-Ser1477 of WT L(3)mbt-L.

C IP/western blotting shows that WT L(3)mbt-S, but not its ΔSAM mutant, co-immunoprecipitated with Lint-O from the OSC lysates. n.i., nonimmune IgG.

D Subcellular localization of WT L(3)mbt-S and its ΔSAM mutant (green). Scale bar: 5 μm.

E Western blotting using the anti-Lint-O antibodies raised in this study. The Lint-O band (~60 kDa) was observed in normal OSCs (Lint-O RNAi−) but not in Lint-O-depleted OSCs (Lint-O RNAi+). Histone H3 (H3) was detected as a loading control. An asterisk shows the background.

Source data are available online for this figure.

© 2022 The Authors

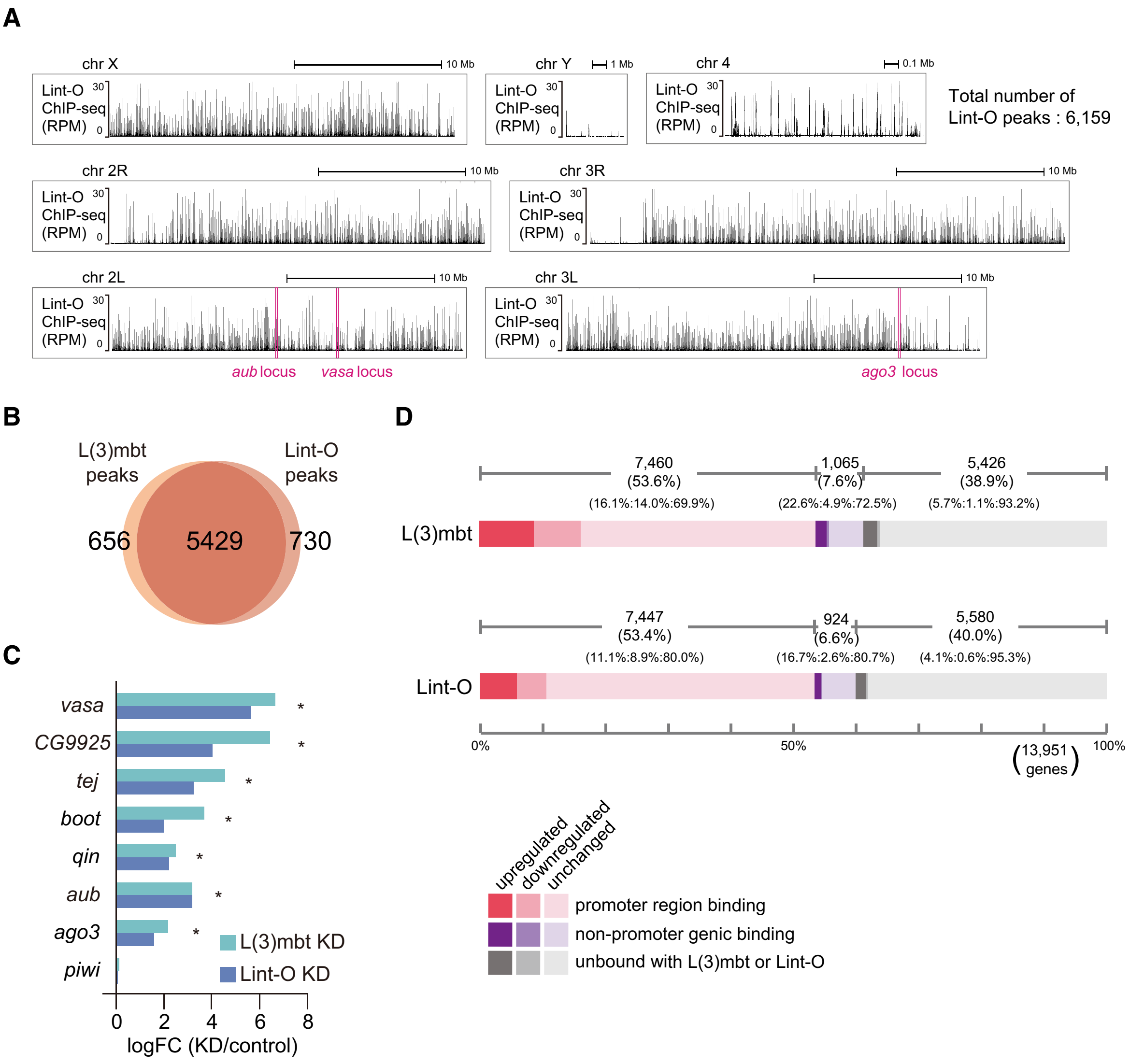

Figure EV4. Lint-O OSC ChIP-seq and RNA-seq before and after Lint-O depletion in OSCs.

A Genomic browser views of the Lint-O ChIP-seq reads. All fly chromosomes are shown. The *y*-axis shows the number of RPM. The *vasa*, *ago3*, and *aub* loci are indicated. The total number of Lint-O ChIP peaks is shown in upper right.

B Overlap between Lint-O ChIP peaks in (A) and L(3)mbt ChIP peaks (Fig EV2A).

C *vasa*, *CG9925*, *tej*, *boot*, *qin*, *aub*, and *ago3* were identified as upregulated genes (* *q*-value <0.05) in RNA-seq analysis. The log fold change of the differential expression for each piRNA pathway gene was calculated based on the RNA-seq data from normal (control), L(3)mbt-depleted (KD), and Lint-O-depleted (KD) OSCs. The *y*-axis shows the ratio of the read counts between L(3)mbt- and Lint-O-depleted OSCs and the normal OSCs.

D Overview and comparison of the gene classification in Figs 1A and 4A.

© 2022 The Authors

Figure EV5. Lint-O ^{KO} ovaries and larval brains.

A Schema depicting the experiment. Eggs were transferred to 29°C after spawning at 25°C and incubated for 13 days. Ovaries were dissected from adult flies.
B–E Confocal images of brains of *y w*, *Lint-O^{KO}*, and *L(3)mbt^{ts1}* larvae (grown at 29°C) for Orb (B), Fas3 (C), Spectrin (Spec) (D), and F-actin (E). *Lint-O^{KO}* ovariole showed defects in follicle cell layer integrity. DAPI (blue): nuclei. Scale bars: 50 μm.
F The *Lint-O-Venus* fly line (*y w Lint-O-Venus*) was generated using the CRISPR /Cas9 system. The Venus sequence was inserted before the stop codon of Lint-O. The Lint-O-Venus signal (green) was detected in the nucleus of both germ and follicle cells in the ovaries. Nuclei were stained with DAPI (blue). Wild-type (*y w*) was used as a negative control. Scale bars: 50 μm.
G Confocal images of *y w*, *Lint-O^{KO}*, and *L(3)mbt^{ts1}* immunostained for Vasa (magenta) and MIRA (green). Merged images were also presented in Fig 6B. Scale bars: 100 μm.
H Enlarged views of insets in (G). Scale bars: 50 μm.
I Confocal images of *y w*, *Lint-O^{KO}*, and *L(3)mbt^{ts1}* immunostained for Vasa (magenta) and ELAV (green). Merged images were also presented in Fig 6B. Scale bars: 100 μm.
J Enlarged views of insets in (I). Scale bars: 100 μm.

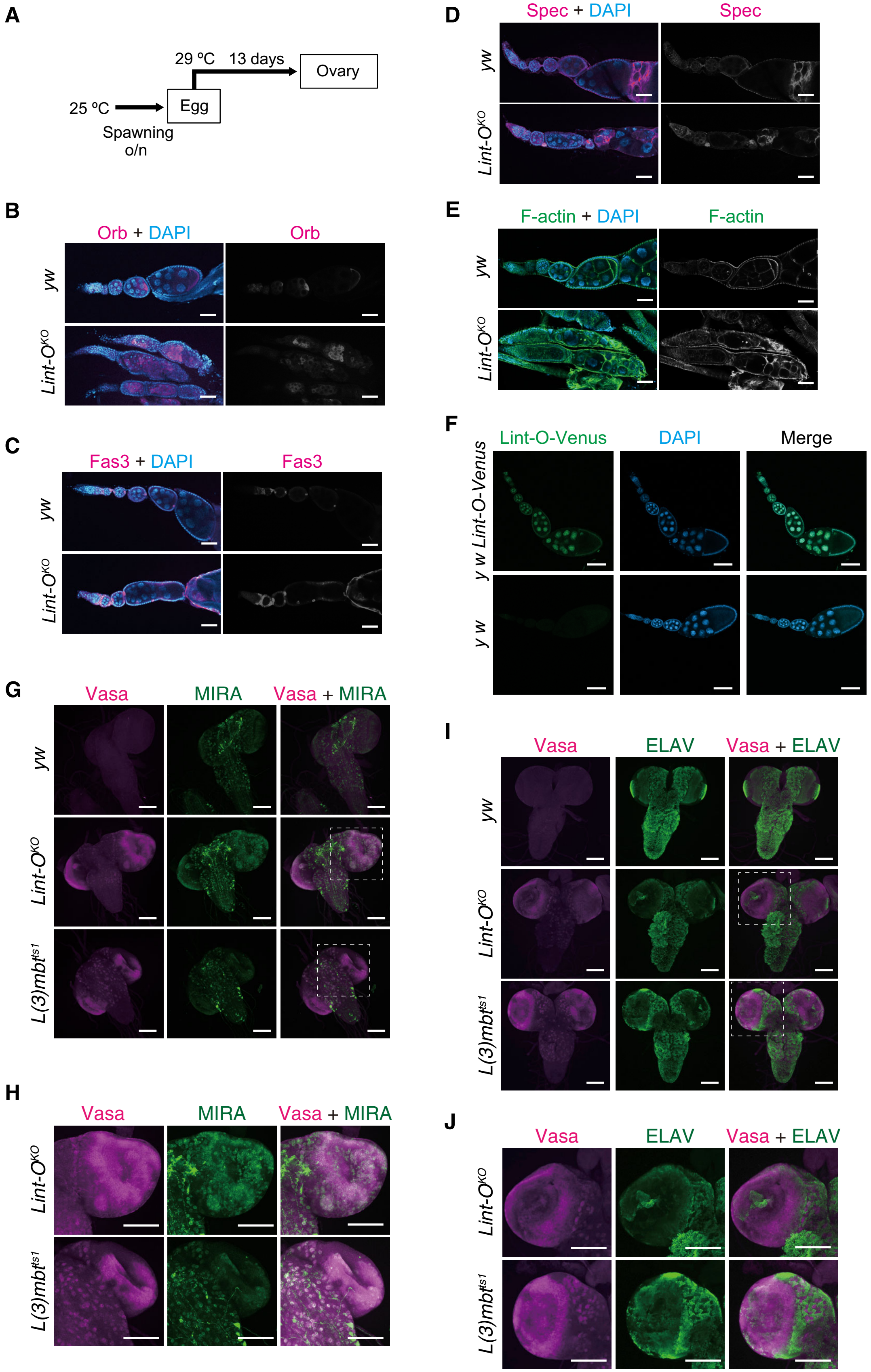

Figure EV5.

© 2022 The Authors
