# Supplementary material for: Lint‐O cooperates with L(3)mbt in target gene suppression to maintain homeostasis in fly ovary and brain
Source: EMBO Rep. 2022 Aug 22;23(10):e53813. doi: 10.15252/embr.202153813 (PMC9535798; doi:10.15252/embr.202153813)
Supplement: Supplementary file 4 — PDF+ [file EMBR-23-e53813-s005.pdf]

Article

SOURCE DATA | TRANSPARENT PROCESS | OPEN ACCESS

EMBO reports

Lint-O cooperates with L(3)mbt in target gene suppression to maintain homeostasis in fly ovary and brain

Hitomi Yamamoto-Matsuda[1,†], Keita Miyoshi[2,3,†], Mai Moritoh[1], Hikari Yoshitane[1], Yoshitaka Fukada[1], Kuniaki Saito[2,3,*], Soichiro Yamanaka[1,**] & Mikiko C Siomi[1,***]

Abstract

Loss-of-function mutations in *Drosophila lethal(3)malignant brain tumor* [l(3)mbt] cause ectopic expression of germline genes and brain tumors. Loss of L(3)mbt function in ovarian somatic cells (OSCs) aberrantly activates germ-specific piRNA amplification and leads to infertility. However, the underlying mechanism remains unclear. Here, ChIP-seq for L(3)mbt in cultured OSCs and RNA-seq before and after L(3)mbt depletion shows that L(3)mbt genomic binding is not necessarily linked to gene regulation and that L(3) mbt controls piRNA pathway genes in multiple ways. Lack of known L(3)mbt co-repressors, such as Lint-1, has little effect on the levels of piRNA amplifiers. Identification of L(3)mbt interactors in OSCs and subsequent analysis reveals CG2662 as a novel co-regulator of L(3)mbt, termed "L(3)mbt interactor in OSCs" (Lint-O). Most of the L(3)mbt-bound piRNA amplifier genes are also bound by Lint-O in a similar fashion. Loss of Lint-O impacts the levels of piRNA amplifiers, similar to the lack of L(3)mbt. The *lint-O*-deficient flies exhibit female sterility and tumorous brains. Thus, L(3)mbt and its novel co-suppressor Lint-O cooperate in suppressing target genes to maintain homeostasis in the ovary and brain.

Keywords fertility; gene regulation; L(3)mbt; Lint-O; piRNA
Subject Categories Chromatin, Transcription & Genomics; RNA Biology
DOI 10.15252/embr.202153813 | Received 16 August 2021 | Revised 8 August 2022 | Accepted 9 August 2022 | Published online 22 August 2022
EMBO Reports (2022) 23: e53813

Introduction

Temperature-sensitive mutations introduced into the *Drosophila* tumor suppressor *lethal(3)malignant brain tumor* [*l(3)mbt*] cause malignant growth of adult optic neuroblasts and ganglion mother cells in the larval brain (Gateff *et al*, 1993; Janic *et al*, 2010). At restrictive temperatures, the mutant flies die at the larval stage, while at permissive temperatures, they are viable but infertile (Coux *et al*, 2018). The expression of L(3)mbt in follicle cells in the ovary is particularly crucial for normal oogenesis to occur.

Genome-wide RNA sequencing (RNA-seq) in the *l(3)mbt* tumorous brain has identified genes under the control of this tumor suppressor, collectively known as the malignant brain tumor signature (MBTS) (Janic *et al*, 2010). MBTS includes germline genes required for the production and amplification of PIWI-interacting RNAs (piRNAs), such as one of the PIWI members *aubergine* (*aub*) and the DEAD-box RNA helicase *vasa*. Forced attenuation of the aberrant expression of these genes in brain tumors makes the tumorous tissue return to normal (Janic *et al*, 2010). This indicates that the function of L(3)mbt in repressing piRNA factors in nongonadal somatic tissues is directly linked to tumor suppression, particularly in the brain.

The piRNAs are a subset of small RNAs enriched in the germline where they silence transposons (Iwasaki *et al*, 2015; Czech *et al*, 2018; Yamashiro & Siomi, 2018; Ozata *et al*, 2019). Upon abrogation of piRNA function, transposons under the control of piRNAs can move within the germline genome. This causes DNA damage, which leads to failure in germline development, resulting in infertility (Schüpbach & Wieschaus, 1991; Klattenhoff *et al*, 2007). Thus, piRNA-mediated transposon silencing is indispensable for a wide range of animals to produce offspring through sexual reproduction.

In cultured ovarian somatic cells (OSCs), which correspond to follicle cells in the ovary, all piRNAs are loaded onto Piwi, one of three PIWI members, giving rise to the piRNA-induced silencing complex (piRISC) (Saito *et al*, 2009). Piwi-piRISC is then localized to the nucleus where it represses transposons transcriptionally by reducing the level of RNA polymerase II at the target loci and/or inducing heterochromatinization around these loci (Yin & Lin, 2007; Klenov *et al*, 2011; Shpiz *et al*, 2011; Wang & Elgin, 2011; Sienski *et al*, 2012; Yashiro *et al*, 2018; Onishi *et al*, 2020, 2021). By contrast, in ovarian germ cells (OGCs), piRNAs are loaded onto all three

1 Department of Biological Sciences, Graduate School of Science, The University of Tokyo, Tokyo, Japan
2 Department of Chromosome Science, National Institute of Genetics, Research Organization of Information and Systems, Shizuoka, Japan
3 Department of Genetics, School of Life Science, SOKENDAI, Shizuoka, Japan
*Corresponding author. Tel: +81 55 981 6823; E-mail: saitok@nig.ac.jp
**Corresponding author. Tel: +81 3 5841 4387; E-mail: soichiro.yamanaka@bs.s.u-tokyo.ac.jp
***Corresponding author. Tel: +81 3 5841 4386; E-mail: siomim@bs.s.u-tokyo.ac.jp
†These authors contributed equally to this work

© 2022 The Authors. Published under the terms of the CC BY 4.0 license.

PIWI members: Piwi, Aub, and Argonaute3 (AGO3). Piwi-bound piRNAs in both OSCs and OGCs are biased toward the antisense orientation of transposon mRNAs and, as in OSCs, Piwi-piRISC in OGCs represses transposons transcriptionally by targeting nascent transcripts while they are being synthesized on the genome (Saito *et al*, 2006; Vagin *et al*, 2006; Czech *et al*, 2018). Aub-bound piRNAs are also biased toward the antisense orientation, but Aub-piRISC silences transposons post-transcriptionally in the cytoplasm by cleaving transposon mRNAs, depending on the endonuclease activity that Aub exhibits (Iwasaki *et al*, 2015; Czech *et al*, 2018). AGO3 also has endonuclease activity, but AGO3-bound piRNAs are mostly sense to transposon mRNAs; thus, AGO3 cleaves transposon transcripts in the antisense orientation. RNAs fragmented by AGO3 are subsequently consumed as precursors for Aub-bound piRNAs. In this way, Aub-piRISCs are amplified. Similarly, Aub-dependent RNA cleavage amplifies AGO3-piRISCs. Aub and AGO3 continue this chain reaction, known as the ping-pong cycle, thereby accumulating a great number of piRISCs in the OGCs (Brennecke *et al*, 2007; Gunawardane *et al*, 2007; Czech *et al*, 2018).

OSCs do not operate the ping-pong cycle because of the lack of Aub and AGO3 expression. Other factors essential for piRNA amplification, such as Vasa, are also not expressed in OSCs (Malone *et al*, 2009; Saito *et al*, 2009). However, *l(3)mbt* is expressed in OSCs (Sumiyoshi *et al*, 2016). As noted above, piRNA factors were aberrantly expressed in the *l(3)mbt* brains; thus, we reasoned that *l(3)mbt* depletion in OSCs would allow the cells to express Aub, AGO3, and Vasa, along with other germ-specific ping-pong factors, amplifying piRNAs that act in post-transcriptional silencing. Indeed, CRISPR-Cas9-mediated genome editing to knock out *l(3)mbt* in OSCs facilitated the expression of Aub and AGO3 (Sumiyoshi *et al*, 2016). Vasa and other piRNA amplifiers have also been detected in L(3)mbt-lacking OSCs (*i.e.*, Δmbt-OSCs), where Aub and AGO3 initiate the ping-pong cycle in a manner dependent on Vasa. We hypothesized that L(3)mbt controls genes involved in the piRNA amplification in OSCs, similar to the manner in which it suppresses germline genes in the brain to inhibit tumorigenesis. However, the mechanism by which L(3)mbt controls the target genes in both tissues remains unknown.

In this study, we conducted chromatin immunoprecipitation sequencing (ChIP-seq) for L(3)mbt in cultured OSCs (Saito *et al*, 2009) concurrently with RNA-seq in the presence and absence of L(3)mbt and found that L(3)mbt controls the expression of piRNA pathway genes in multiple fashions. This function of L(3)mbt does not largely depend on known L(3)mbt co-repressors, such as Lint-1 and Myb (Lewis *et al*, 2004; Georlette *et al*, 2007; Meier *et al*, 2012). We then sought and analyzed L(3)mbt interactors in OSCs and identified CG2662 as a novel L(3)mbt co-repressor, which was termed "L(3)mbt-interacting protein in OSCs" (Lint-O). Comparison of L(3)mbt and Lint-O ChIP-seq reads revealed that the L(3)mbt-bound piRNA amplifier genes were mostly bound with Lint-O. The piRNA amplifiers derepressed by L(3)mbt depletion were similarly derepressed by Lint-O depletion. We also found that Lint-O was unstable in the absence of L(3)mbt, but not *vice versa*, suggesting distinct functionalities of L(3)mbt and Lint-O despite their tight relationship in gene regulatory function. The *lint-O* knockout flies, *Lint-O^{KO}*, produced in this study exhibited female sterility at permissive temperatures and developed tumorous brain at restrictive temperatures, similar to the *l(3)mbt*-deficient flies. We argue that Lint-O is a novel co-suppressor of L(3)mbt that cooperates tightly with L(3)mbt to regulate genes, such as piRNA amplification genes, to maintain female fertility and suppress brain tumors.

Results

OSCs express two L(3)mbt variants, L(3)mbt-L and L(3)mbt-S

To conduct genome-wide ChIP-seq for L(3)mbt in OSCs, we first produced an anti-L(3)mbt monoclonal antibody. Western blotting using this antibody detected L(3)mbt as a doublet in OSC lysates (Fig EV1A). Both bands disappeared upon RNA interference (RNAi) treatment for L(3)mbt (Fig EV1A), suggesting that both are L(3)mbt. Hereafter, we refer to the ~ 190 and ~ 150 kDa bands as L(3)mbt-L and L(3)mbt-S, respectively.

A previous study showed that the *l(3)mbt* gene bears multiple isoforms of which the expression levels change during development (Wismar *et al*, 1995). We thus inferred that L(3)mbt-L and L(3)mbt-S may correspond to two isoforms. To examine whether this inference is correct, we performed rapid amplification of cDNA ends (RACE) experiments using total RNAs isolated from OSCs. Three different 5′ ends of *l(3)mbt* mRNA were detected (#1–3, Fig EV1B). Two distal 5′ ends (#1 and #2) were located within the 5′-untranslated region (UTR) of the *l(3)mbt* RNA transcript annotated in FlyBase (FBtr0085175). By contrast, the most proximal 5′ end (#3) was not within the annotated *l(3)mbt* sequence. Rather, it was found to be within the 5′ long terminal repeat (LTR) of *springer* retrotransposon inserted into the third intron of *l(3)mbt* in the OSC genome (Sienski *et al*, 2012). This *l(3)mbt* isoform consists of a portion of *springer* (294 bases: nucleotides #152–445) and exons 4 and 5 of the *l(3)mbt* gene (Fig EV1B). Western blotting showed that L(3)mbt expressed from the authentic cDNA and its truncated version in OSCs comigrated with endogenous L(3)mbt-L and L(3)mbt-S, respectively (Fig EV1C). Thus, L(3)mbt-L corresponds to the full-length (FL) L(3)mbt, and L(3)mbt-S is its truncated form, which highly likely starts with Met325 of L(3)mbt-L. The level of piRNAs against *springer* is negligible in OSCs (Sienski *et al*, 2012). Therefore, the allele encoding L(3)mbt-S is spared from piRNA-mediated regulation, enabling the expression in OSCs. Immunofluorescence of OSCs using anti-L(3)mbt antibody detected the L(3)mbt signals nearly exclusively in the nucleus (Fig EV1D), suggesting that both L(3)mbt isoforms are localized to the nucleus, similarly to L(3)mbt in other cell types such as neuroblasts (Richter *et al*, 2011) and cultured Kc cells (Meier *et al*, 2012).

L(3)mbt controls piRNA amplifiers in multiple ways in OSCs

We performed L(3)mbt ChIP-seq in OSCs using the anti-L(3)mbt monoclonal antibody. The experiment was conducted twice, and statistical analysis confirmed the high correlation between the two libraries (Appendix Fig S1A). An overview of the ChIP-seq reads mapped on the *Drosophila* genome is presented in Fig EV2A. We then extracted ChIP-seq reads corresponding to 13,951 protein-coding genes in Flybase by allowing the reads to map the promoter region of each gene and analyze. Note that the promoter region in this study refers to the genomic region spanning from 0.35 kilobase (kb) upstream of the transcriptional start site (TSS) to 0.1 kb downstream of the TSS, based on the previously reported DNA-binding status of L(3)mbt in *Drosophila* larval tissues (Richter *et al*, 2011). This analysis showed that L(3)mbt can bind to 8,525 of 13,951 protein-coding

 © 2022 The Authors

genes (61.1%) (Fig 1A). We further classified these genes according to where in each gene L(3)mbt is bound. This revealed that 7,460 genes had promoter region binding of L(3)mbt (53.5% in 13,951 genes). We designated this group as “promoter region binding.” The remaining 1,065 genes were designated as “nonpromoter genic binding” (7.6%). The 5,426 genes that did not belong to either group were designated as “unbound with L(3)mbt” (38.9%).

Comparison of the ChIP-seq reads with the OSC RNA-seq reads before and after L(3)mbt depletion revealed that the expression levels of 1,202 genes among the 7,460 “promoter region binding” genes were upregulated by L(3)mbt loss (16.1%), while 1,044 genes were downregulated (14.0%) (Fig 1A). Note that RNA-seq was conducted three times, and principal component analysis (PCA) confirmed the high correlation in the libraries (Fig EV2B). We previously showed that seven germ-specific piRNA biogenesis genes - *vasa*, *aub*, *ago3*, *qin*, *tejas* (*tej*), *bootlegger* (*boot*), and *CG9925* - were among the genes prominently upregulated by L(3)mbt depletion (Sumiyoshi *et al*, 2016). In this study, we found that five of these genes - *vasa*, *qin*, *tej*, *boot*, and *CG9925* - were within the “upregulated” category of the “promoter region binding” group

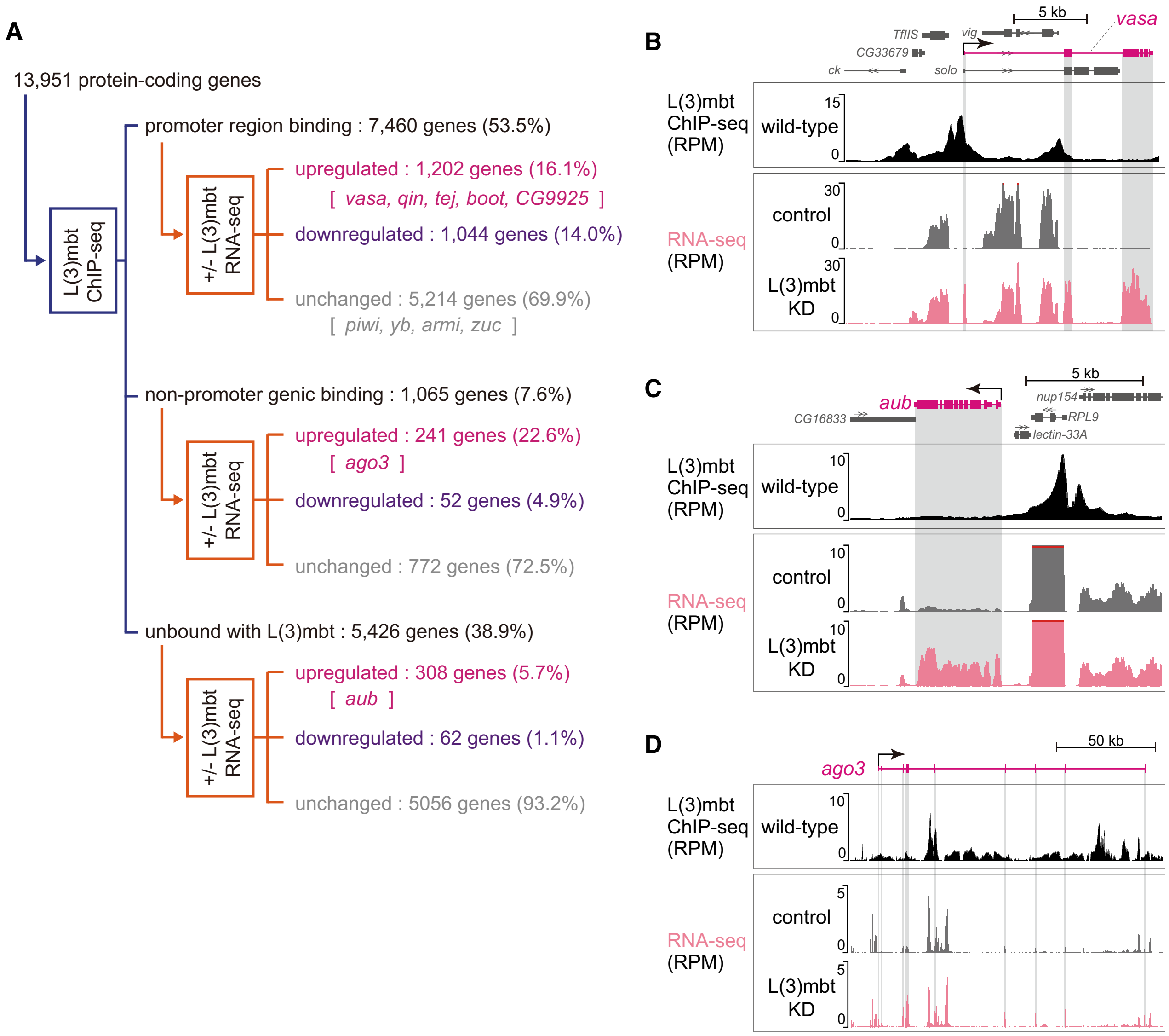

Figure 1. L(3)mbt controls target gene expression in complex fashions.

A The 13,951 protein-coding genes of *Drosophila* were classified into “promoter region binding,” “nonpromoter genic binding,” and “unbound with L(3)mbt” in accordance with the L(3)mbt ChIP-seq reads, and were subsequently divided into “upregulated,” “downregulated,” and “unchanged” in accordance with the RNA-seq reads from OSCs before and after L(3)mbt depletion [+/— L(3)mbt]. Representatives of piRNA factors are indicated within the groups. ChIP-seq was performed twice technically and RNA-seq three times.

B–D The genomic regions harboring *vasa* (B), *aub* (C), and *ago3* (D). The gene structure is shown at the top. The arrow depicts the TSS and the orientation of transcription. The L(3)mbt ChIP-seq reads in normal OSCs (wild-type) and RNA-seq reads from L(3)mbt-depleted [L(3)mbt KD] and control OSCs are shown under the gene structure. The shading in gray corresponds to exons. The *y*-axis shows the number of reads per million mapped reads (RPM).

© 2022 The Authors

(Fig 1A). The patterns of binding of L(3)mbt to these genes and the RNA-seq signals at the loci are shown in Fig 1B (*vasa*) and Fig EV2C (*qin*, *tej*, *boot*, and *CG9925*).

The key somatic piRNA factors, *piwi*, *female sterile(1)Yb* (*yb*), *armitage* (*armi*), and *zucchini* (*zuc*), all of which are intrinsically expressed in normal OSCs, belonged to the "promoter region binding" group (Fig 1A). Their expression levels were unchanged by the loss of L(3)mbt (Fig EV2D), agreeing with our previous observation (Sumiyoshi *et al*, 2016). We previously showed that Piwi expression in OSCs depends on the transcriptional factor Traffic jam (Tj) (Saito *et al*, 2009), indicating that as long as Tj is functional, Piwi will be expressed regardless of the presence or absence of L(3)mbt. It seems that the regulation of *piwi* via Tj takes precedence over the regulation via L(3)mbt.

Likewise, the promoter of *flamenco* (*flam*), the piRNA cluster responsible for bearing most piRNAs within OSCs (Brennecke *et al*, 2007), was bound with L(3)mbt via the promoter, but the expression level was not impacted by the lack of L(3)mbt (Fig EV2E). An earlier study showed that the expression of *flam* depends on the transcriptional factor Cubitus interruptus (Goriaux *et al*, 2014). L(3)mbt binds to a relatively wide range of genes, but for genes functioning in OSCs, there appear to be activators that override the repression by L(3)mbt.

The *aub* and *ago3* genes were classified into the "unbound with L(3)mbt" and "nonpromoter genic binding" categories, respectively (Fig 1A). Both genes were upregulated by L(3)mbt depletion (Fig 1C and D), as expected based on our earlier observation (Sumiyoshi *et al*, 2016). At the *aub* locus, the L(3)mbt binding was concentrated in the upstream part of *aub*, but all sites were distant from the TSS (Fig 1C). From the perspective of neighboring genes, the L(3)mbt signals were rich in the promoter regions of *nup154* and *RPL9*. However, the expression levels were not changed by the lack of L(3)mbt (Fig 1C). In this regard, *nup154* and *RPL9* can be considered as equivalents of *piwi* and *yb*. The L(3)mbt ChIP signals at the *ago3* gene were biased to introns but not to the promoter (Fig 1D). It seems that L(3)mbt genomic binding is not always linked to gene regulation and that L(3)mbt regulates piRNA pathway genes in multiple fashions.

L(3)mbt ChIP-seq reads and OSC RNA-seq reads before and after L(3)mbt depletion were also classified based on the distance from most proximal TSS (Appendix Fig S1B and C). Note that the "L(3)mbt in the TSS region" group corresponds to the "promoter region binding" group in Fig 1A.

Known L(3)mbt co-suppressors are likely irrelevant for L(3)mbt-driven gene regulation in OSCs

L(3)mbt resides in two repressive chromatin complexes, the LINT complex (Meier *et al*, 2012) and the dREAM complex [*a.k.a.* the Myb–MuvB (MMB) complex] (Lewis *et al*, 2004; Georlette *et al*, 2007; Blanchard *et al*, 2014). An earlier genetic study showed that the L(3)mbt function in the ovaries requires Lint-1, a component of the LINT complex but is independent of the dREAM complex (Coux *et al*, 2018). To understand in which of these two complexes L(3)mbt exerts its function in OSCs, we depleted components of the two complexes by RNAi (Appendix Fig S2A and B) and examined how these treatments affected the levels of *aub*, *ago3*, *piwi*, *and vasa*. The expression of *aub* was upregulated by the loss of Lint-1 and CoRest, both of which are LINT complex components, but this upregulation was milder than that following L(3)mbt loss (Fig 2A). Upon depletion of E2F2, Mip120, and Mip130, all of which are contained within the dREAM complex, the level of *aub* was barely changed. Knockdown of Myb in the dREAM complex slightly upregulated *aub*, but the effect was not as great as that of Lint-1 or CoRest knockdown (Fig 2A). These findings suggest that L(3)mbt function in regulating the *aub* gene in OSCs is independent of the dREAM complex but dependent on the LINT complex, similar to the circumstances in the ovaries, but with lower extent. The changes in the level of *ago3* under these conditions were similar to those of *aub* (Appendix Fig S2C). The levels of *piwi* remained stable under each condition, as expected (Appendix Fig S2D). In sharp contrast, the increases in the expression of *vasa* following the loss of all of these known L(3)mbt co-factors were much lower than that following L(3)mbt depletion (Fig 2B). One plausible scenario is that, when L(3)mbt controls target genes via the promoter (*e.g.*, *vasa*), it is almost independent of both LINT and dREAM complexes, but when L(3) controls target genes via regions other than the promoter (*e.g.*, *aub* and *ago3*), it may subtly depend on the LINT complex.

Lint-O is a novel co-repressor of L(3)mbt in OSCs

Our findings above prompted us to identify novel L(3)mbt co-suppressor(s). To this end, we isolated the L(3)mbt complex from OSCs before and after L(3)mbt RNAi (Fig 2C) and forwarded the materials for mass spectrometric analysis. Peptides corresponding to 100 proteins were obtained from normal OSCs (Fig 2D and Appendix Table S1). The number of peptides corresponding to 21 proteins among these 100 proteins was reduced four-fold or more by L(3)mbt depletion (Appendix Table S1). Exclusion of the ribosomal proteins and L(3)mbt reduced the number of candidate proteins to 17. Gene Ontology (GO) analysis categorized 7 of the 17 proteins as DNA-binding and chromatin regulation factors (Fig 2D and E).

The effects of Lint-1 deficiency on *aub*, *vasa*, and *ago3* were already examined (Fig 2A and B and Appendix Fig S2C). Histone H3.3A and RNA polymerase II subunit 3 (Rpb3) seemed to be rather general factors and were therefore not considered to be strong candidates. We then examined the contribution of four other factors, CG2662, CG2199, proliferation disrupter (Prod), and Ada2a-containing complex component 3 (Atac3), in the regulation of *vasa* and *aub*. Quantitative reverse-transcription PCR (RT–qPCR) showed that the level of *vasa* was increased upon the depletion of CG2662, similar to that of L(3)mbt, but was unchanged by the depletion of CG2199, Prod, or Atac3 (Fig 2F and Appendix Fig S2E). The level of *aub* was also influenced by CG2662 loss as efficiently as L(3)mbt loss (Fig 2G). These findings suggested that CG2662 is a co-suppressor of L(3)mbt more closely related to it than any known L(3)mbt co-suppressors, including Lint-1. Thus, we termed CG2662 "L(3)mbt-interacting protein in OSCs" (Lint-O) and continued our investigation to reveal the functional relationship between L(3)mbt and Lint-O.

The L(3)mbt-Lint-O interaction is necessary for piRNA pathway gene regulation

L(3)mbt contains three malignant brain tumor (MBT) repeats responsible for binding methylated histone marks (Wismar *et al*, 1995; Bonasio *et al*, 2010), a C2H2-type zinc finger, and a sterile

 © 2022 The Authors

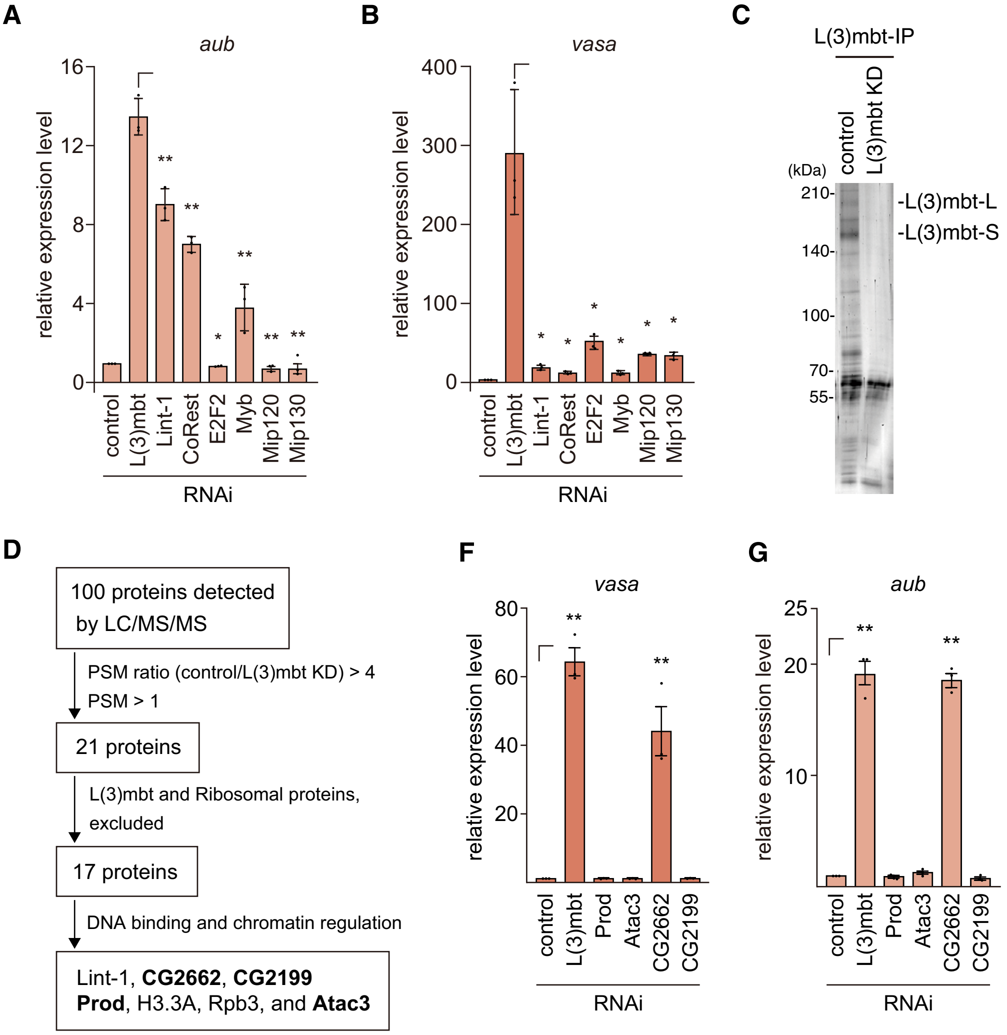

E

Protein	SPS	PSM	Function
Lint-1	37.046	54	L(3)mbt co-suppressor
**CG2662**	14.463	14	Unknown
**CG2199**	3.391	2	Unknown
**Prod**	1.992	2	DNA binding
Rpb3	2.102	3	RNA polymerase II subunit
H3.3A	1.259	2	Nucleosome component
**Atac3**	0.94	2	Chromatin remodeling

Figure 2. CG2662/Lint-O binds to and functions with L(3)mbt in gene regulation.

A, B The mRNA levels of *aub* (A) and *vasa* (B) were quantified upon the depletion (RNAi) of L(3)mbt, Lint-1, CoRest, E2F2, Myb, Mip120, and Mip130, and were compared to those in normal OSCs (control). Data represent the mean ± SE ($n = 3$ biological replicates). The P values were calculated with the t-test. $^{*}P < 0.05$, $^{**}P < 0.01$. All t-tests were performed against samples with ⌈ symbol.

C Proteins immunoprecipitated with the anti-L(3)mbt antibody from the OSC lysates before (control) and after L(3)mbt knockdown (KD) were silver-stained. The bands corresponding to L(3)mbt-L and L(3)mbt-S are indicated.

D Flow chart of the identification of L(3)mbt interactors in OSCs. All peptides obtained from LC–MS/MS are listed in Appendix Table S1.

E Summary of the sum of the −log posterior error probability (Sum Pep Score: SPS), peptide spectra match values (PSM), and known functions of the proteins in (D). Mass spectrometry analysis was performed on biological duplicates.

F, G The mRNA levels of *vasa* (F) and *aub* (G) were quantified upon the depletion (RNAi) of L(3)mbt, Atac3, Prod, CG2662/Lint-O, and CG2199, and were compared to those in normal OSCs (control). Data represent the mean ± SE ($n = 3$ biological replicates). The P values were calculated with the t-test. $^{**}P < 0.01$. All t-tests were performed against samples with ⌈ symbol.

Source data are available online for this figure.

© 2022 The Authors

alpha motif (SAM) domain that acts in protein–protein interactions (Kim & Bowie, 2003) (Fig 3A). Lint-O consists of two plant homeodomain (PHD) finger domains and a SAM domain (Fig 3A). The PHD finger is often found in chromatin-associated proteins that read histone H3 marks such as H3K4me2/3 to regulate target gene expression (Sanchez & Zhou, 2011).

To confirm the association between L(3)mbt and Lint-O, we ectopically expressed wild-type (WT) Lint-O, tagged with 3x FLAG at the C-terminus, in OSCs. The L(3)mbt complex immunopurified from the OSC lysates was then probed with anti-FLAG and anti-L(3)mbt antibodies. WT Lint-O signal was detected along with that of L(3)mbt (Fig 3B). However, when the Lint-O mutant lacking the SAM domain, ΔSAM (Fig EV3A), was expressed instead of WT Lint-O, the mutant failed to co-immunoprecipitate with L(3)mbt (Fig 3B). The ΔSAM mutant was localized to the nucleus as was the WT control (Fig 3C). In subsequent experiments, both L(3)mbt-L and L(3)mbt-S, but not their ΔSAM mutants, bound to Lint-O (Figs 3D and EV3B and C), although all those proteins were localized to the nucleus (Figs 3E and EV3D). These findings confirm the L(3)mbt-Lint-O association in OSCs and indicate that their interaction depends on the SAM domain of each of the two proteins.

We next investigated whether the L(3)mbt-Lint-O interaction is important for regulating target gene expression. RT–qPCR showed that *vasa* and *aub* were upregulated in Lint-O-lacking OSCs (Fig 3F and G). We then ectopically expressed RNAi-resistant WT Lint-O in these cells. This treatment rescued the defects caused by the loss of endogenous Lint-O (Fig 3F and G). When the ΔSAM mutant, which was also RNAi-resistant, was expressed, the *vasa* and *aub* genes were not re-silenced (Fig 3F and G). These findings indicated that the L(3)mbt-Lint-O interaction via the SAM domain is essential for the cooperative function of L(3)mbt and Lint-O in regulating the gene targets.

These rescue assays were also performed using another Lint-O mutant, 8CA, where eight cysteines within the two PHD finger domains were altered to alanine (Fig EV3A). This mutant was not expected to re-repress *vasa* and *aub* because other PHD finger-containing proteins have been shown to anchor zinc ions via the cysteines and this ion anchoring is essential for these proteins to exert their functions (Kalkhoven *et al*, 2002). However, contrary to our expectations, the 8CA mutant re-repressed *vasa* and *aub* to an extent similar to that of the WT (Fig 3F and G). Thus, Lint-O may not require zinc ions to regulate its target genes. The 8CA mutant was able to interact with L(3)mbt (Fig 3H) and localize to the nucleus (Fig 3I). However, when the PHD finger domains were removed from Lint-O (Fig EV3A), the ΔPHD mutant was unable to interact with L(3)mbt (Fig 3H) although its nuclear localization was unaffected by the lack of PHD domains (Fig 3I). These results support the idea that the ability of Lint-O to associate with chromatin via the PHD domains influences the L(3)mbt-Lint-O interaction. This idea was further supported by the observation that the binding activity of L(3)mbt to the *vasa* promoter was significantly weakened by the absence of Lint-O (Fig 3J). The binding activity of Lint-O to L(3)mbt appeared to be slightly increased by the 8CA mutation (Fig 3H). This may be because the mutation stabilized the binding of Lint-O to chromatin.

In the course of our study, we noticed that, in the absence of L(3)mbt, endogenous Lint-O was barely detected by western blotting using anti-Lint-O antibodies raised in this study (Figs 3K and EV3E). The level of *lint-O* mRNA was not impacted by the lack of L(3)mbt (Fig 3L). By contrast, Lint-O depletion little influenced the level of L(3)mbt (Fig 3K). Thus, the L(3)mbt-Lint-O interaction is required for the stabilization of Lint-O but not of L(3)mbt. These results support the idea that L(3)mbt and Lint-O have distinct functions even though they bind to each other and cooperate in gene regulation in OSCs.

L(3)mbt and Lint-O cooperate in regulating the expression of piRNA amplifiers in OSCs

We next conducted Lint-O ChIP-seq in OSCs using the anti-Lint-O antibodies. The experiment was conducted twice, and statistical analysis confirmed the high correlation between the two libraries (Appendix Fig S3A). An overview of ChIP-seq reads mapped on the

Figure 3. The L(3)mbt-Lint-O interaction is crucial for the repression of target genes.

A Domain structures of L(3)mbt and Lint-O. L(3)mbt has three MBT repeats (orange), a C2H2-type zinc finger (Zf) (gray), and a SAM domain (blue). Lint-O has two PHD finger domains (green) and a SAM domain (blue).

B Immunoprecipitation (IP)/western blotting shows that WT Lint-O, but not its ΔSAM mutant (Fig EV3A), co-immunoprecipitated with L(3)mbt from the OSC lysates. n.i., nonimmune IgG.

C Subcellular localization of WT Lint-O and its ΔSAM mutant (green). Both proteins were localized to the nuclei (DAPI: blue). Scale bar: 5 μm.

D IP/western blotting shows that WT L(3)mbt-L, but not its ΔSAM mutant (Fig EV3B), co-immunoprecipitated with Lint-O from the OSC lysates. n.i., nonimmune IgG.

E Subcellular localization of WT L(3)mbt-L and its ΔSAM mutant (green). Both proteins were localized to the nuclei (DAPI: blue). Scale bar: 5 μm.

F, G The mRNA levels of *vasa* (F) and *aub* (G) were quantified upon ectopic expression of EGFP, and WT Lint-O and its ΔSAM and 8CA mutants (Fig EV3A), in Lint-O-lacking OSCs (Lint-O RNAi) and were compared to those in normal OSCs (control). Data represent the mean ± SE ($n = 3$ biological replicates). The P values were calculated with the t-test. *$P < 0.05$. All t-tests were performed against samples with ⌈ symbol.

H IP/western blotting shows that L(3)mbt co-immunoprecipitated with WT Lint-O and its 8CA mutant, but not with ΔPHD mutant (Fig EV3A). An asterisk shows the background.

I Subcellular localization of WT Lint-O and its 8CA and ΔPHD mutants (green). All proteins were localized to the nuclei (DAPI: blue). Scale bar: 5 μm.

J ChIP–qPCR shows that L(3)mbt binding to the *vasa* promoter was weakened after the loss of Lint-O. Data represent the mean ± SE ($n = 3$ biological replicates). The P values were calculated with the t-test. **$P < 0.01$. All t-tests were performed against samples with ⌈ symbol.

K Western blotting showing the amounts of L(3)mbt, Lint-O, and histone H3 (H3) in normal (control), L(3)mbt-depleted (RNAi), and Lint-O-depleted (RNAi) OSCs. Asterisks show background signals as in Fig EV3E.

L The *lint-O* mRNA levels in normal (control), L(3)mbt-depleted (RNAi), and Lint-O-depleted (RNAi) OSCs are shown by fragments per kilobase million (FPKM). Data represent the mean ± SE ($n = 3$ biological replicates). The P values were calculated with the t-test. *$P < 0.05$, **$P < 0.01$. All t-tests were performed against samples with ⌈ symbol.

Source data are available online for this figure.

© 2022 The Authors

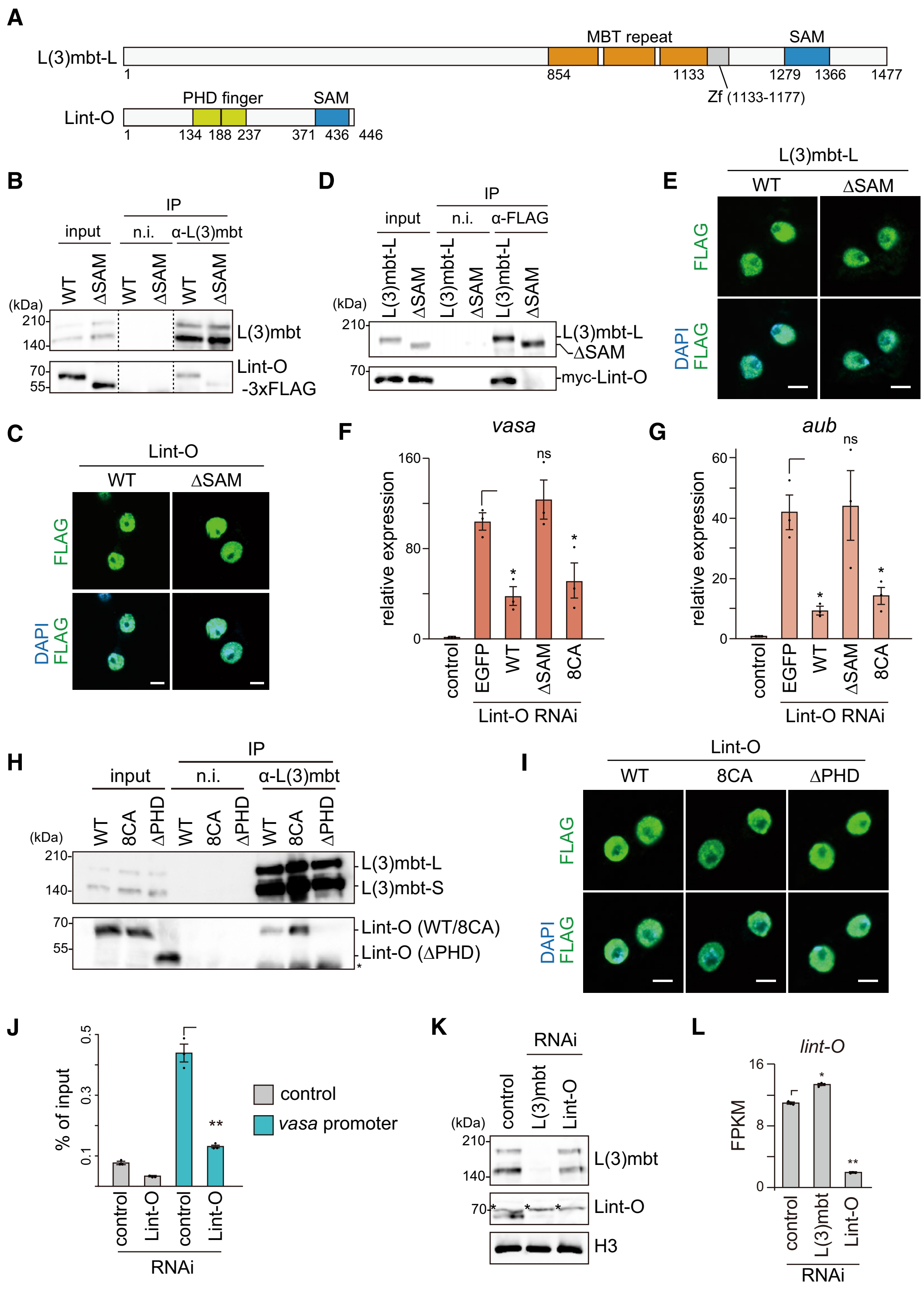

Figure 3.

Drosophila genome is presented in Fig EV4A. The degree of overlap between Lint-O peaks and L(3)mbt peaks is indicated in Fig EV4B. Further analysis showed that Lint-O bound to 7,447 protein-coding genes via their promoter regions (53.4% of a total of 13,951 genes) ("promoter region binding" in Fig 4A). Comparison of the ChIP-seq reads with the OSC RNA-seq reads before and after Lint-O depletion (Fig EV2B) revealed that 827 genes of the 7,447 "promoter region binding" group were upregulated by the loss of Lint-O (11.1%), whereas 663 genes were downregulated (8.9%) (Fig 4A). The *vasa*, *qin*, *tej*, *boot*, and *CG9925* genes belonged to the "upregulated" category as they belonged to the equivalent in L(3)mbt ChIP-seq (Fig 1A). The expression levels of the other 5,957 genes were little

impacted by Lint-O depletion (80.0%) (Fig 4A). The *piwi*, *yb*, *armi*, and *zuc* genes belonged to this category as they belonged to the counterpart in Fig 1A.

A total of 924 of 13,951 protein-coding genes were classified into the "nonpromoter genic binding" group (6.6%) (Fig 4A). Of these, 154 genes were upregulated by the loss of Lint-O (16.7%) and the *ago3* gene was in this category. In addition, 5,580 genes were "unbound with Lint-O" (40.0%) (Fig 4A). Of these, 231 genes were upregulated by the loss of Lint-O (4.1%), and *aub* belonged to this category. The browser views of Lint-O ChIP-seq and RNA-seq in the presence and absence of Lint-O at the *vasa*, *aub*, and *ago3* loci are shown in Fig 4B–D. Basically, the Lint-O

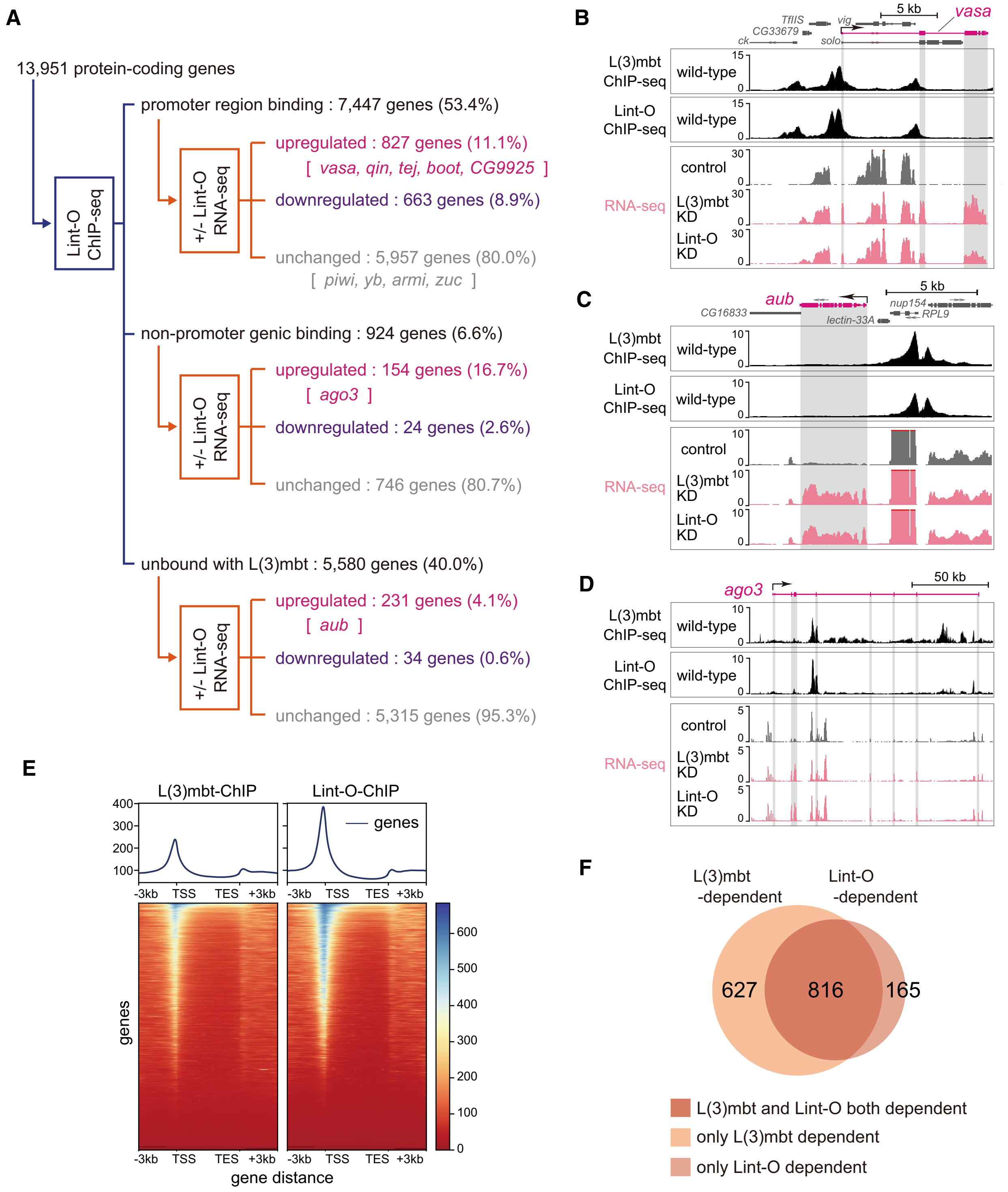

Figure 4.

 © 2022 The Authors

Figure 4. L(3)mbt and Lint-O share genomic binding to orchestrate gene control in OSCs.

A The 13,951 protein-coding genes of *Drosophila* were classified into “promoter region binding,” “nonpromoter genic binding,” and “unbound with Lint-O” in accordance with the Lint-O ChIP-seq reads, and were subsequently divided into “upregulated,” “downregulated,” and “unchanged” in accordance with the RNA-seq reads from the OSCs before and after Lint-O depletion (+/− Lint-O). Representatives of piRNA factors are indicated. ChIP-seq was performed twice technically and RNA-seq three times.

B–D The genomic regions harboring *vasa* (B), *aub* (C), and *ago3* (D) are indicated. The L(3)mbt and Lint-O ChIP-seq reads and the RNA-seq reads from normal (control), L(3)mbt-depleted, and Lint-1-depleted OSCs are shown. The L(3)mbt ChIP-seq reads and the RNA-seq reads from normal (control) and L(3)mbt-depleted were also shown in Fig 1B–D.

E Heatmaps show the L(3)mbt ChIP and Lint-O ChIP scores calculated by deeptools (Ramírez *et al*, 2016) within each gene body and the extended regions (*i.e.*, 3 kb upstream of the TSS and 3 kb downstream of the TES). The length of all the genes is normalized to be a constant value, 5 kb. The summary plots show the average score.

F Venn diagram showing the overlap between L(3)mbt- and Lint-O-regulated genes.

genomic binding patterns are nearly identical to those of L(3)mbt, further indicating the collaborative functions of the two proteins in piRNA amplifier gene regulation.

The five “promoter region binding” genes (*i.e.*, *vasa*, *CG9925*, *tej*, *boot*, and *qin*) and two “nonpromoter genic binding” genes (*i.e.*, *aub* and *ago3*) were similarly upregulated in L(3)mbt- and Lint-O-depleted OSCs (Fig EV4C). In each case, the lack of Lint-O appeared less influential than that of L(3)mbt. Such a trend was also observed in the number of genes affected by the lack of L(3)mbt/Lint-O (based on RNA-seq) (Fig EV4D); namely, the number of genes influenced by the lack of L(3)mbt was slightly higher than that influenced by the lack of Lint-O. This may be related to the observation that Lint-O becomes unstable upon the lack of L(3)mbt (Fig 3K).

Lint-O ChIP-seq reads and OSC RNA-seq reads before and after Lint-O depletion was also classified based on the distance from most proximal TSS (Appendix Fig S3B). We also analyzed the binding frequencies of L(3)mbt and Lint-O to the TSS and transcriptional end sites (TES) of the protein-coding genes. The histogram indicated that both proteins tend to bind strongly to the TSS and weakly to the TES and downstream regions (Fig 4E). L(3)mbt and Lint-O may rarely repress target genes by binding to introns (*e.g.*, *ago3*) or upstream regions far from the TSS (*e.g.*, *aub*). The regulation of *aub* and/or *ago3* by L(3)mbt and Lint-O may also be indirectly mediated by transcription factors or other regulators that are directly activated by the loss of L(3)mbt/Lint-O.

A previous study showed that brain tumors in *l(3)mbt* mutant flies originated from deregulation of target genes in the Salvador-Warts-Hippo (SWH) pathway (Richter *et al*, 2011). Another study asserted that inappropriate expression of Nanos (Nos), a translational repressor and key regulator of germline fate, in ovarian follicular (somatic) cells was the main cause of defects in germline development observed in the *l(3)mbt* mutants (Coux *et al*, 2018). ChIP-seq and RNA-seq at two genes under the control of the SWH pathway, *drosophila inhibitor of apoptosis 1* and *expanded*, showed that both L(3)mbt and Lint-O bound to the promoters of the two genes, albeit weakly, but the mRNA levels were barely changed by the loss of L(3)mbt or Lint-O (Appendix Fig S3C). This suggested that dependence on the L(3)mbt/Lint-O functions varies among cells. The *nos* promoter was bound with L(3)mbt and Lint-O relatively strongly and the mRNA level was upregulated following L(3)mbt or Lint-O depletion, but the expression was still far lower than that of *nos* in the ovary in the FlyAtlas 2 (Leader *et al*, 2018) (Appendix Fig S3D and E). The expression of *nos* may be activated when OSCs reside in the vicinity of germ cells in the ovary.

L(3)mbt and Lint-O may function independently of each other in gene regulation

MBTS is not restricted to germ-specific piRNA factors (Janic *et al*, 2010). For the next part of our study, we collected 1,443 protein-coding genes under the control of L(3)mbt [*i.e.*, genes that were bound with L(3)mbt, irrespective of whether this was promoter binding or nonpromoter binding, and upregulated by the L(3)mbt depletion] (Fig 1A) and 981 genes under the control of Lint-O (*i.e.*, genes that were bound with Lint-O, irrespective of whether this was promoter binding and nonpromoter binding, and upregulated by the loss of Lint-O) (Fig 4A), and compared the gene pools. The Venn diagram showed that 816 genes were shared between the two groups (Fig 4F). We attempted to sort these genes according to GO terms, but the statistical analysis did not enrich for significant terms. We next focused on 627 genes that were suppressed via the L(3)mbt binding but in a Lint-O-independent matter. These genes could be controlled by the combination of L(3)mbt and co-factors other than Lint-O [*i.e.*, “L(3)mbt-dependent and Lint-O-independent”]. However, no significant GO terms were found. A similar result was obtained for “L(3)mbt-independent and Lint-O-dependent” genes (165 genes). We also focused on downregulated genes. We found that 439 genes were downregulated by depletion of both L(3)mbt and Lint-O (Appendix Fig S3F). Again, no significant terms were found. L(3)mbt/Lint-O appears to regulate a wide variety of intracellular pathway genes in OSCs.

Lint-O is essential for ovarian morphogenesis and oocyte development

To investigate the importance of Lint-O function in flies, we generated *lint-O*-deficient mutants by applying the CRISPR-Cas9 system. A guide RNA was designed to introduce an indel mutation in the *lint-O* coding sequence (Fig 5A). A mutant fly harboring a single-nucleotide deletion was obtained. In this mutant, the *lint-O* expression was severely attenuated (Fig 5B); thus, the *lint-O* mutant was designated $Lint\text{-}O^{KO}$. The mutant flies were viable at permissive temperatures but exhibited female infertility at 29°C (Fig 5C) as the temperature-sensitive *l(3)mbt* mutant, $L(3)mbt^{ts1}$, showed female sterility at permissive temperatures (Coux *et al*, 2018).

The $Lint\text{-}O^{KO}$ ovaries appeared to be morphologically abnormal at 29°C. To investigate this in more detail, we immunostained the mutant ovaries with antibodies specific for Vasa, oo18 RNA-binding protein (Orb), Fasciclin 3 (Fas3), and Spectrin (Spec), and with phalloidin, a chemical that specifically binds to filamentous actin (F-

© 2022 The Authors

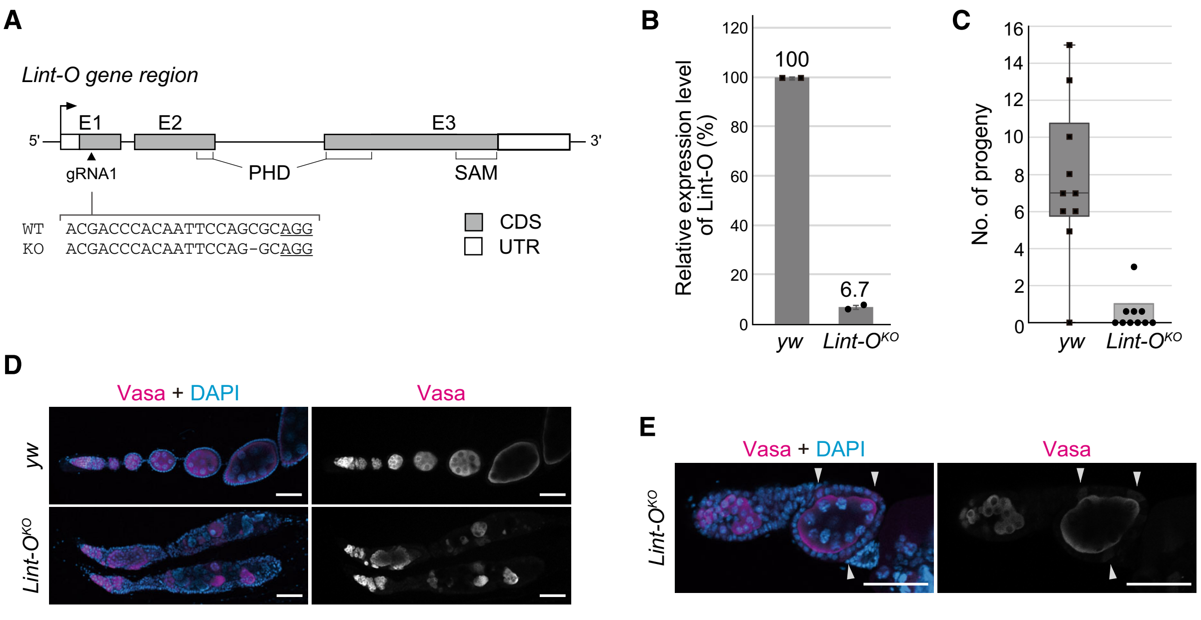

Figure 5. The lack of Lint-O in flies leads to abnormal ovarian morphology and female sterility.

A CRISPR-mediated generation of *lint-O* knockout (KO). Genomic structure of *lint-O* is shown with gRNA targeting *lint-O* exon 1 (triangle). Sequences of *lint-O* DNA in WT (*y w*) (complementary to the gRNA sequence) and the KO mutant, *Lint-O*^{KO}, are shown. PAM: protospacer adjacent motif (underlined). E1-3: Exons 1–3. CDS: protein-coding sequence. UTR: untranslated region.

B RT–qPCR analysis shows the mRNA levels of *lint-O* in *y w* and *Lint-O*^{KO} ovaries. Data are expressed as mean and error bars represent SD. n = 2 biological replicates.

C The numbers of progeny in *y w* and *Lint-O*^{KO} are shown. Ten independent crosses were performed. Boxplot central bands, upper edges of boxes, lower edges of boxes, upper whiskers, and lower whiskers show median, third quartile, first quartile, maxima, and minima, respectively.

D Confocal images of *y w* and *Lint-O*^{KO} ovaries immunostained for Vasa. Scale bar: 50 µm.

E Confocal images of *y w* and *Lint-O*^{KO} ovarioles immunostained for Vasa. Vasa was ectopically expressed in follicle cells (white arrowheads). Scale bar: 50 µm.

actin). Vasa has been shown to be ectopically expressed in cultured OSCs upon L(3)mbt depletion and somatic cells in *L(3)mbt*^{ts1} ovaries (Sumiyoshi *et al*, 2016; Coux *et al*, 2018). Similarly, Vasa was detected in somatic (follicle) cells in the *Lint-O*^{KO} ovaries at 29°C (Figs 5D and E and EV5A).

The expression of Orb in the *yellow white* (*y w*) ovaries that we employed as a WT control was restricted to the developing oocyte at the posterior of egg chambers (Fig EV5B), as reported previously (Lantz *et al*, 1994). However, in the ovaries of *Lint-O*^{KO}, Orb was mislocalized to the anterior region of the egg chamber, where a few cells had gathered abnormally (Fig EV5B). Fas3 is a cell adhesion molecule abundant in polar cells during the late stages of oogenesis (Ruohola *et al*, 1991). The polar cells in *y w* localize to the edge of the anterior–posterior axis of the follicular cells (Fig EV5C). By contrast, the egg chamber of *Lint-O*^{KO} had excessive and ectopic polar cells. It seemed that ovarioles were fused during ovary morphogenesis in the absence of *lint-O* expression. Spec localizes to somatic cell membranes and the spectrosome/fusome while the basic F-actin polymer generates a dynamic cytoskeletal network (Theurkauf *et al*, 1993; Lin *et al*, 1994). Spec and F-actin immunostaining signals indicated that the egg chambers were indeed fused in *Lint-O*^{KO} at restrictive temperatures (Fig EV5D and E). These findings indicate that Lint-O plays an essential role in ovarian morphogenesis and oocyte development, similar to L(3)mbt (Coux *et al*, 2018).

When fly ovaries were immunostained with anti-Lint-O antibody, no signal was detected. Therefore, the gene encoding Venus was knocked in at the *lint-O* genomic loci. Fluorescent signals were detected in both germ and somatic cells (Fig EV5F). It is not yet known whether the ovarian phenotype described above is due to loss of Lint-O expression in germ cells, somatic cells, or both. We infer that somatic cell expression of Lint-O may be important for the maintenance of ovarian morphology, similar to L(3)mbt (Coux *et al*, 2018).

L(3)mbt and Lint-O cooperatively control target genes in the brain to suppress tumorigenesis

When L(3)mbt was depleted in the third-stage (L3) larvae, germline genes such as *vasa*, *piwi*, and *aub* were ectopically expressed in the brain, resulting in tumorigenesis (Janic *et al*, 2010; Richter *et al*, 2011). Remarkably, western blotting showed that Vasa, Piwi, and Aub proteins were ectopically expressed in the brain of *Lint-O*^{KO} L3 larvae (Fig 6A). Immunofluorescence detected Vasa relatively strongly in Miranda (MIRA)-positive neuroblast cells (Betschinger *et al*, 2006) but not in ELAV-positive neuronal cells (Robinow & White, 1991) (Figs 6B and EV5G–J). Lint-O may repress Vasa expression in undifferentiated cells but not in terminally differentiated cells. The *l(3)mbt* mutants ectopically expressed Vasa particularly in the outer proliferative center (OPC) and in the central brain (CB) neuroblasts (Janic *et al*, 2010; Richter *et al*, 2011). Similar cell specificity was observed for the *Lint-O*^{KO} L3 larval brain (Fig EV5G–J). In addition, the *Lint-O*^{KO} brain was enlarged, as was the *L(3)mbt*^{ts1} brain, although it was slightly smaller (Fig 6C). These results support the intriguing concept that Lint-O plays an important role in L(3)mbt-mediated inhibition of tumorigenesis in the brain.

© 2022 The Authors

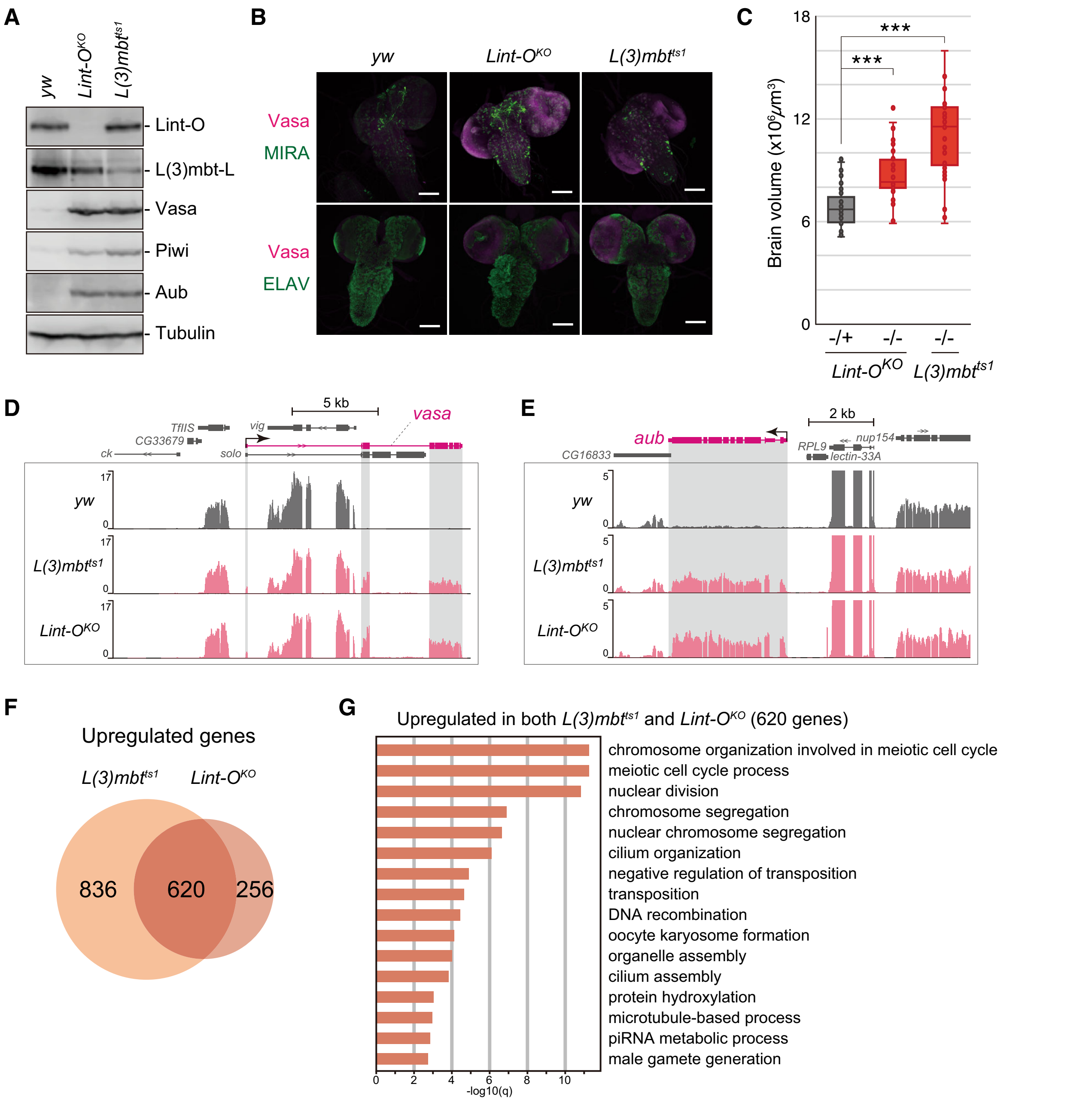

Figure 6. L(3)mbt and Lint-O cooperatively control target genes in the brain.

A Western blotting shows that Vasa, Piwi, and Aub were ectopically expressed in *Lint-O*^{KO} and *L(3)mbt*^{ts1} larval brains at 29°C. Anti-Lint-O, anti-L(3)mbt, anti-Vasa, andi-Piwi, anti-Aub, and anti-Tubulin antibodies were used.

B Confocal images of *y w*, *Lint-O*^{KO}, and *L(3)mbt*^{ts1} immunostained for Vasa (magenta), MIRA (green), and ELAV (green). Scale bar: 100 μm.

C Quantification of brain lobe volume for the following genotypes: *Lint-O*^{KO} −/+ (n = 42 biological replicates), *Lint-O*^{KO} −/− (n = 36 biological replicates), and *L(3) mbt*^{ts1} −/− (n = 34 biological replicates). Boxplot central bands, upper edges of boxes, lower edges of boxes, upper whiskers and lower whiskers show median, third quartile, first quartile, maxima, and minima, respectively. *P* values were calculated by the Student's *t*-test. (****P*-value < 0.001).

D, E The genomic regions harboring the *vasa* (D) and *aub* (E) genes. The RNA-seq reads in *y w*, *L(3)mbt*^{ts1}, and *Lint-O*^{KO} brains are shown. The shading in gray corresponds to exons. The *y*-axis shows the number of RPM. RNA-seq samples were biological triplicates.

F Venn diagram shows that 620 protein-coding genes are shared with the *l(3)mbt* and *lint-O* libraries.

G High-ranking GO terms for the 620 protein-coding genes in (F).

Source data are available online for this figure.

We next performed genome-wide RNA-seq in the L3 brains from *y w*, *L(3)mbt*^{ts1}, and *Lint-O*^{KO}. The mapping of the RNA-seq reads onto *vasa* and *aub* confirmed their derepression in *L(3)mbt*^{ts1} and *Lint-O*^{KO} (Fig 6D and E). Computational analysis revealed that 620 genes were upregulated commonly in *L(3)mbt*^{ts1} and *Lint-O*^{KO} (Fig 6F). GO analysis of these genes showed that the terms with high statistical values

[q-value (−log10) > 10] included "chromosome organization," "meiotic cell cycle," and "nuclear division." The term of "piRNA metabolic process" also appeared [q-value (−log 10) = 2.86] (Fig 6G). These results indicate that L(3)mbt/Lint-O tends to regulate genes that function in specific intracellular pathways in the brain, such as the piRNA pathway. In agreement with this, not only the *vasa* and *aub* genes but also other germline-specific piRNA factors (*e.g.*, *piwi*, *spindle-E*, *krimper*, *tej*, *sister of Yb*, *vreteno*, and *nxf2*) appeared in the gene set (GSE181802). On the other hand, no significant GO terms were found for the L(3)mbt-dependent and Lint-O-independent genes (836 genes) and the L(3)mbt-independent and Lint-O-dependent genes (256 genes). These findings suggest that L(3)mbt and Lint-O may regulate some specific genes independently from each other, but they do not seem to be aimed at regulating any special pathways.

Discussion

The loss-of-function mutations in the *Drosophila l(3)mbt* gene cause malignant tumor growth in the brain. This finding was first reported in 1982 (Gateff, 1982). Since then, a number of studies have been conducted to elucidate the function of L(3)mbt and to determine its causal relationship with tumorigenesis. Through this, multiple co-factors, such as Lint-1, CoRest, and Myb, were identified (Lewis *et al*, 2004; Georlette *et al*, 2007; Meier *et al*, 2012). However, until the present study, Lint-O had never been reported to be an L(3)mbt co-suppressor, showing the complexity of the L(3)mbt-mediated gene regulatory pathway. What was the key leading to the discovery of Lint-O in this study? One plausible answer is the use of OSCs, a cultured cell line composed solely of somatic (follicular) cells derived from *Drosophila* ovary, not from the brain.

Upon the lack of L(3)mbt, OSCs aberrantly induced the expression of germ-specific genes including piRNA amplifiers and activated the ping-pong pathway, in which piRNAs innately generated in naïve OSCs were actually amplified (Sumiyoshi *et al*, 2016). The *Drosophila* brain autonomously initiated piRNA biogenesis upon the loss of *l(3)mbt* function (Janic *et al*, 2010). Strikingly, when piRNA biogenesis was forcibly attenuated, the tumorigenesis ceased. We previously attempted L(3)mbt knockdown in nongonadal somatic Schneider 2 (S2) cells to examine whether it activated piRNA biogenesis. However, piRNA biogenesis was not carried out because necessary factors were not sufficiently activated. This implies that OSCs and the brain, particularly CB neuroblasts that became Vasa-positive upon *l(3)mbt* deficiency (Janic *et al*, 2010; Richter *et al*, 2011; this study), have some commonality, but S2 cells do not. At present, it is unclear what this commonality consists of materially, besides the two main players L(3)mbt and Lint-O. However, the susceptibility of both cells to the loss of function of L(3)mbt and Lint-O is likely to be almost identical. In other words, the degree and mechanism of cooperative gene regulation between L(3)mbt and Lint-O, and the balance with other gene regulatory means, are considered to be equivalent in the two cases. If there was a CB-derived cultured cell line, we could carry out similar experiments in both cells in parallel and compare the outcomes to obtain an understanding of the commonality. However, no such cell line appears to be available in the field of *Drosophila* research.

In this study, by performing genome-wide ChIP-seq and RNA-seq in parallel in OSCs, we were able to understand the implications of the genomic binding of L(3)mbt, such as which binding is actually responsible for the expression of the gene targets. Similar assays can be conducted using the ovary, for example, but its tissue is composed of germarium and egg chambers at different stages of development. Egg chambers are a mixture of nurse cells and oocytes, with follicular cells surrounding them. The follicular cells normally lack Vasa and Aub, and only in abnormal situations such as the depletion of L(3)mbt and/or Lint-O, these proteins are aberrantly upregulated. The nurse cells express Vasa irrespective of the presence or absence of L(3)mbt or Lint-O. With this complexity, it would not be easy to interpret the experimental results accurately and obtain a correct understanding. This reminds us of the usefulness of *in vivo* and *ex vivo* systems and the importance of combining the two.

Lint-1 was present in the L(3)mbt interactors in OSCs. This led us to speculate that L(3)mbt interacts with not only Lint-O but also Lint-1 to assemble a highly ordered complex to regulate the target genes in a cooperative manner. However, the *lint-O* brain was tumorigenic, similar to the *l(3)mbt* brain (but it showed slightly less brain enlargement). This implies that L(3)mbt and Lint-1, likely as the LINT complex with CoRest, contribute less to the regulation of germline genes in the brain. The situation could be similar in OSCs. Another plausible idea is that, in the absence of Lint-O, Lint-1 may become unstable as Lint-O became unstable in the absence of L(3)mbt. Given our present understanding, this is not an unreasonable proposition, but further research is required to properly understand the relationship among these three proteins.

The *l(3)mbt* gene is conserved from nematodes to humans, although the domain structures may not be strictly identical among them (Bonasio *et al*, 2010). Humans express four L(3)mbt orthologs, L3MBTL1 to L3MBTL4. Of these, L3MBTL1, L3MBTL3, and L3MBTL4 contain three MBT repeats, a zinc finger, and a SAM domain, similar to *Drosophila* L(3)mbt, but L3MBTL2 contains four MBT repeats and a zinc finger but lacks the SAM domain (Boccuni *et al*, 2003; Bateman, 2019). According to their peptide sequences, the protein sizes of human L3MBTLs are approximately half that of the fly L(3)mbt (Boccuni *et al*, 2003; Bateman, 2019). These inter-species differences suggest that L(3)mbt orthologs may exert their function(s) in different ways in individual organisms.

Are there any human orthologs of Lint-O? Previous proteomics analysis identified the sterile alpha motif domain containing 13 (SAMD13) as an interactor of human L(3)MBTL3 (Hauri *et al*, 2016; Huttlin *et al*, 2017). SAMD13 has a SAM domain but lacks the PHD domain that Lint-O has, but it is recognized as a human ortholog of Lint-O based on a BLAST search. Although this study did not go so far as to remark the function of the PHD domain of Lint-O, we found that the PHD domain is required for the functional expression of Lint-O. In this regard, SAMD13 may not be the human version of Lint-O. One possibility is that factors that bind to SAMD13 may have a PHD domain or something similar. However, this is also a matter of conjecture, and further research is needed before any conclusions are drawn. It is still unclear whether loss of L3MBTLs causes brain tumors or infertility in humans. It is also unclear whether loss of SAMD13 causes serious damage to the human brain and germline. Future research results on these interesting issues are awaited.

Previous studies showed that L(3)mbt binds to insulator elements of several insulator factors such as CP190, CTCF, and BEAF-32 (Richter *et al*, 2011; Bortle *et al*, 2012). Although these factors did not appear as interacting factors for L(3)mbt in OSCs in this

© 2022 The Authors

study (Fig 2E), it is possible that L(3)mbt and Lint-O regulate gene expression through higher chromatin organization in addition to binding to the gene body. *ago3* may be one of the genes regulated by chromatin organization.

Materials and Methods

Cell culture

OSCs (Saito *et al*, 2009; RRID:CVCL_IY73) were cultured at 26°C in Shields and Sang M3 Insect Medium (US Biological) supplemented with 10% fly extract (Saito *et al*, 2009), 10% fetal bovine serum (Funakoshi), 10 mU/ml insulin, and 0.6 mg/ml glutathione.

Plasmid construction

To construct L(3)mbt-L-3xFLAG, L(3)mbt-S-3xFLAG, myc-Lint-O- and Lint-O-3xFLAG-expressing plasmids, a full-length L(3)mbt-L/-S/ Lint-O cDNA was amplified from cDNA library produced from total RNAs of OSCs using First Strand cDNA Synthesis Kit (Roche). The 5′-end of L(3)mbt-L/S was determined by 5′ RACE using the SMARTer RACE 5′/3′ Kit (Clontech). Then, amplified cDNA was cloned into vectors that have the promoter of actin and myc/ 3xFLAG tags using NEBuilder HiFi DNA Assembly Cloning Kit (NEB). For the rescue assays, Lint-O-3xFLAG expressing plasmids were modified to be siRNA-resistant using inverse PCR and infusion. The ΔSAM and ΔPHD mutants were produced by inverse PCR on L (3)mbt-L/S-3xFLAG and Lint-O-3xFLAG (siRNA-resistant) and ligation. The Lint-O-8CA mutant was produced by mutagenesis using inverse PCR on Lint-O-3xFLAG (siRNA-resistant) and ligation. Primers used are summarized in Appendix Table S2.

RNAi and plasmid transfection

For performing RNAi, up to 6 × 10^6 trypsinized OSCs were prepared and suspended in 20 μl of Solution SF from the Cell Line Nucleofector Kit SF (Lonza Bioscience) to which 400 pmol siRNA duplex was added. Transfection was performed with the Nucleofector 96-well Shuttle Device (Lonza Bioscience). For performing transfection of the plasmids along with siRNAs, up to 1 × 10^7 trypsinized OSCs were prepared and suspended in buffer [180 mM sodium phosphate buffer for Church and Gilbert hybridization (pH 7.2) containing 15 mM $MgCl_2$, 5 mM KCl, and 50 mM D-mannitol], modified from the original protocol (Nye *et al*, 2014). A total of 6 μg of the plasmids and 600 pmol of the siRNA duplex were added to the suspended cells, and transfection was performed with the Nucleofector 2b Device (Lonza Bioscience) using the N-020 program. Transfected cells were cultured at 26°C in a fresh OSC medium for 48 h for further experiments. For the rescue assays, RNAi-treated cells were incubated for 48 h before co-transfection of the plasmids and siRNA duplex. After co-transfection, the cells were cultured for another 48 h. The sequences of siRNA duplexes are listed in Appendix Table S2.

Production of antibodies

The recombinant L(3)mbt peptide (Lys401-Thr600) tagged with glutathione S-transferase [GST-L(3)mbt] was purified from *E. coli* and used for mouse immunization. The production and selection of hybridomas were performed as described in a previous report (Nishida *et al*, 2015). To produce the anti-Lint-O antibody, a part of the protein (Gln340-Ser353) was used to immunize rabbits. The antibody was purified from the serum and then dialyzed against PBS. The Lint-O peptide and antibody were prepared by Eurofins Scientific. A Lint-O peptide (Met1-Arg130) fused to 6 x His was expressed in and purified from *E. coli*, and then injected into 8-week-old female Tsl:BALB/cCr[GF] mice (Sankyo Lab) for immunization. The blood serum was used for experiments using flies. Mouse procedures were approved by the Animal Research Committee of the University of Tokyo (Animal plan16-3).

Nuclear extraction and immunoprecipitation

Nuclear lysates were prepared and immunoprecipitation was performed as described previously (Onishi *et al*, 2020). Briefly, cells were suspended in hypotonic buffer [10 mM HEPES-KOH (pH 7.3), 10 mM KCl, 1.5 mM $MgCl_2$, 0.5 mM dithiothreitol (DTT), 2 μg/ml pepstatin A, 2 μg/ml leupeptin, and 0.5% aprotinin], and the cells were fractured by passing them three times through a 25-gauge needle. The nuclear fraction was first centrifuged at 400 *g* for 10 min, washed with hypotonic buffer by centrifugation at 13,600 *g* for 20 min, and resuspended in chromatin co-immunoprecipitation (co-IP) buffer [50 mM HEPES-KOH (pH 7.3), 200 mM KCl, 1 mM EDTA, 1% Triton X-100, 0.1% sodium deoxycholate, 2 μg/ml pepstatin A, 2 μg/ml leupeptin, and 0.5% aprotinin]. The nuclear fraction was sonicated on ice using a Branson Digital Sonifier and then centrifuged at 20,000 *g* for 20 min at 4°C. The supernatant was collected as the nuclear extract. For immunoprecipitation, the nuclear lysate was incubated with antibodies bound to Dynabeads Protein G (Thermo Fisher Scientific) at 4°C for 2 h. The beads were washed five times with co-IP buffer. Proteins were eluted from the beads by heating at 95°C for 3 min in buffer containing SDS. After electrophoresis, the samples for LC–MS/MS were visualized by silver staining using SilverQuest (Invitrogen) or processed for western blotting.

Western blotting

Western blotting was performed as described previously (Miyoshi *et al*, 2005). For primary antibodies, anti-L(3)mbt (culture supernatant from the hybridoma) (this study), anti-Lint-O (rabbit blood serum, 1:10 dilution) (this study), anti-FLAG (SIGMA M2, 1:5,000 dilution), anti-Vasa (8E12, purified, 1:1,000 dilution) (Nishida *et al*, 2015), anti-Piwi (4D2 cultured supernatant) (Saito *et al*, 2006), anti-Aub (4D10, purified, 1:1,000 dilution) (Nishida *et al*, 2015), anti-Tubulin [E7 culture supernatant, Developmental Studies Hybridoma Bank (DSHB)], anti-histone H3 (Abcam ca#ab1791, 1:2,000 dilution), and anti-Myc monoclonal (Fujifilm Wako Pure Chemical 9E10, 1:1,000 dilution) antibodies were used.

Immunofluorescence

Immunofluorescence was performed as described previously (Saito *et al*, 2010). The primary antibodies used in this study were anti-L (3)mbt (1:500 dilution) and anti-FLAG monoclonal (MBL FLA-1, 1:1,000 dilution, and SIGMA M2, 1:1,000 dilution) antibodies. The secondary antibodies used were Alexa 488-conjugated anti-mouse

© 2022 The Authors

antibody (Thermo Fisher Scientific, 1:500 dilution). The cells were mounted with the VECTASHIELD Mounting Medium and stained with DAPI (Vector Laboratories), followed by observation using an LSM 710 or LSM 980 laser scanning confocal microscope (Carl Zeiss). Immunostaining of ovaries was performed following standard procedures. Fixation of ovaries was carried out with 4% paraformaldehyde phosphate buffer. Mouse anti-Vasa (1:500 dilution) (Nishida *et al*, 2015), mouse anti-Spectrin (1 μg/ml, 3A9, DSHB), mouse anti-Fas3 (1 μg/ml, 7G10, DSHB), and mouse anti-Orb (1 μg/ml, 4H8, DSHB) antibodies were used as primary antibodies. The specificity of the staining for F-actin was verified by Phalloidin-iFluor488 (1:1,000 dilution, #23115, AAT Bioquest). Larval brains were dissected in cold PBS and fixed in 4% paraformaldehyde for 40 min. Samples were incubated with primary antibodies diluted in PBS containing 0.3% Triton X-100 and 5% BSA (PBSTB) overnight at 4°C. Primary antibodies used were as follows: mouse anti-Vasa (8E12, 1:50 dilution) (Nishida *et al*, 2015), rat anti-MIRA (1:500 dilution, ab197788, Abcam), and rat anti-ELAV (1:100 dilution, 7E8A10, DSHB). Brains were washed three times for 10 min in PBST and then incubated in secondary antibodies overnight at 4°C. Secondary antibodies were used as follows: Alexa 546-conjugated anti-mouse IgG (1:1,000 dilution, Molecular Probes) or Alexa 488-conjugated anti-rat IgG (1:1,000 dilution, Molecular Probes). Samples were incubated with secondary antibodies overnight at 4°C. After washing three times for 10 min in PBST, samples were mounted in the VECTASHIELD Mounting Medium and stained with DAPI (Vector Laboratories). Images of ovaries and larval brains were collected using a confocal microscope (Zeiss LSM900).

RNA extraction and RT–qPCR

Total RNAs were extracted from OSCs with the ISOGEN II reagent (Nippon Gene). After digestion of the contaminated DNAs by DNase (Life Technologies) and subsequent purification, a cDNA library was prepared using the reverse-transcription kit ReverTra Ace (Toyobo). For RT–qPCR, cDNAs or DNA products from ChIP were amplified using the StepOnePlus Real-Time PCR System (Applied Biosystems) with PCR enzymes and the PowerUp SYBR Green Master Mix (Thermo Fisher Scientific). The efficiency of qPCR amplification was calculated based on the slope of the standard curve. After determining the amplification efficiency values (between 95% and 105%), the relative steady-state RNA levels were calculated from the threshold cycle for amplification. The sequences of primers are listed in Appendix Table S2.

Preparation of protein samples for LC–MS/MS

For the preparation of immunoprecipitated samples for LC–MS/MS, the anti-L(3)mbt antibody used for immunoprecipitation was cross-linked to beads by dimethyl pimelimidate (Thermo Fisher Scientific). Immunoprecipitation was performed using control OSCs and L(3)mbt knockdown (KD) OSCs. The cells were washed after incubation six times with 800 mM NaCl in HEMG buffer [25 mM HEPES-KOH (pH 7.3), 12.5 mM $MgCl_2$, 0.1% Nonidet P-40, 0.1 mM EDTA, 20% glycerol, 2 μg/ml pepstatin A, 2 μg/ml leupeptin, and 0.5% aprotinin] and then twice with 200 mM NaCl in HEMG buffer. The immunoprecipitants were eluted in elution buffer containing 125 mM Tris–HCl (pH 6.8), 4% SDS, and 0.01% bromophenol blue by heating at 70°C for 10 min. The products were precipitated in acetone containing 20% trichloroacetic acid. The same experiment was performed again to generate a replicate. The pellets containing immunoprecipitated samples were lysed in 100 μl of PTS buffer [100 mM NH_4HCO_3, 12 mM sodium deoxycholate, 12 mM sodium N-lauroylsarcosinate, and phosphatase inhibitor cocktails 2 and 3 (Sigma-Aldrich)]. Each sample in the PTS buffer was reduced with 10 mM DTT at 60°C for 30 min and then alkylated by incubation with 22 mM iodoacetamide at 37°C for 30 min in the dark. Next, the samples were diluted with 100 mM NH_4HCO_3 solution up to a volume of 500 μl and digested with 0.4 μg of trypsin (Roche) by incubation at 37°C for 18 h in the dark. After the digestion, an equal volume of ethyl acetate was added to the samples, and the mixture was acidified with 0.5% TFA and mixed to transfer the detergents into the organic phase. After the samples were centrifuged at 15,700 *g* for 1 min at room temperature, the aqueous phases containing the peptides were collected. The samples were concentrated by a centrifugal evaporator (Eyela) and desalted using a MonoSpin C18 column (GL Sciences). The eluted products were dried prior to LC–MS/MS analysis.

LC–MS/MS analysis

The dried and desalted peptides were dissolved in distilled water containing 2% acetonitrile and 0.1% TFA. The LC–MS/MS analyses were performed using a mass spectrometer (Q Exactive Plus, Thermo Fisher Scientific) equipped with a nano ultra-high-performance liquid chromatography system (Dionex Ultimate 3000, Thermo Fisher Scientific). The peptides were loaded onto the LC–MS/MS system with a trap column (0.3 × 5 mm, L-column, octadecylsilyl groups; Chemicals Evaluation and Research Institute) and a capillary column (0.1 × 150 mm, L-column, octadecylsilyl groups, Chemicals Evaluation and Research Institute) at a flow rate of 10 μl/min. The loaded peptides were separated by a gradient using the mobile phases A (1% formic acid in distilled water) and B (1% formic acid in acetonitrile) at a flow rate of 300 nl/min (2% to 35% B in 133 min, 35% to 50% B in 20 min, 50% to 95% B in 2 min, 95% B for 10 min, 95% to 2% B in 0.1 min and 2% B for 10 min). The eluted peptides were electrosprayed (2.0 kV) and introduced into the MS equipment (positive ion mode, data-dependent MS/MS). Each of the most intense precursor ions (the top 10) was isolated and fragmented by higher collision energy dissociation with normalized collision energy (27%). For full MS scans, the scan range was set to 350–1,500 *m/z* at a resolution of 70,000, and the AGC target was set to 3e6 with a maximum injection time of 60 ms. For the MS/MS scans, the precursor isolation window was set to 1.6 *m/z* at a resolution of 17,500 and the AGC target was set to 5e5 with a maximum injection time of 60 ms. The Orbitrap mass analyzer was operated with the "lock mass" option to perform shotgun detection with high accuracy. The raw spectra were extracted using Proteome Discoverer 2.2 (Thermo Fisher Scientific) and searched against the *Drosophila* UniProt database (TaxID = 7,227 and subtaxonomies) with the following settings: the parameter for the cleavage was set to trypsin, and a maximum missed number of cleavages of two were allowed. The mass tolerances were set to 10 ppm for the precursor ion and 0.02 Da for the fragment ion. As for protein modifications, we set carbamidomethylation (+ 57.021 Da) at Cys as a fixed modification of the peptide, oxidation (+ 15.995 Da) at Met as

 © 2022 The Authors

a dynamic (nonfixed) modification of the peptide, and acetylation (+ 42.011 Da) at the N-terminus as a dynamic modification of the protein terminus. The amount of each peptide was semi-quantified using the peak area with Precursor Ions Quantifier in Proteome Discoverer 2.2.

ChIP and ChIP-seq library preparation

For chromatin immunoprecipitation, cultured OSCs (1×10^7 cells/reaction) were fixed by incubation with an OSC medium including 0.75% formaldehyde for 5 min at room temperature, following which glycine was added to the medium (final concentration, 125 mM) to stop the fixation. Cells were scraped and suspended with hypotonic buffer [25 mM HEPES-KOH (pH 7.3), 1.5 mM $MgCl_2$, 10 mM KCl, 1 mM DTT, 0.1% NP-40, 2 μg/ml pepstatin A, 2 μg/ml leupeptin, and 0.5% aprotinin] and the nuclei were centrifuged into pellets at 1,000 *g* for 10 min. The nuclei were diluted in sonication buffer [50 mM HEPES-KOH (pH 7.3), 140 mM NaCl, 1% Triton X-100, 0.1% sodium deoxycholate, 0.1% SDS, 1 mM EDTA, 2 μg/ml pepstatin A, 2 μg/ml leupeptin, and 0.5% aprotinin] and sonicated with a Covaris S220 Focused ultrasonicator for 10 min at 4°C. The settings used for the sonication were as follows: peak power 140, duty factor 5.0, and 200 cycle/burst. Next, the lysates were centrifuged at 20,000 *g* for 20 min, and the supernatants were collected for immunoprecipitation. Anti-L(3)mbt and anti-Lint-O antibodies were added to the supernatants and incubated for 16 h at 4°C. Dynabeads Protein G (Thermo Fisher Scientific) was added to each sample and incubated for 1 h at 4°C. After incubation, beads were washed once with low-salt buffer [20 mM Tris–HCl (pH 8.0), 150 mM NaCl, 2 mM EDTA, 0.1% Triton X-100, 0.1% SDS, and 1 mM phenylmethylsulfonyl fluoride (PMSF)], high-salt buffer [20 mM Tris–HCl (pH 8.0), 500 mM NaCl, 2 mM EDTA, 0.1% Triton X-100, 0.1% SDS, and 1 mM PMSF], and LiCl buffer [20 mM Tris–HCl (pH 8.0), 200 mM LiCl, 2 mM EDTA, 1% NP-40, 1% sodium deoxycholate, and 1 mM PMSF], and twice with TE buffer [20 mM Tris–HCl (pH 8.0), 1 mM EDTA, and 1 mM PMSF]. The beads were dissolved in elution buffer [50 mM Tris–HCl (pH 8.0), 10 mM EDTA, and 1% SDS] and incubated at 65°C for 30 min. The supernatant containing the immunoprecipitated materials was collected, to which NaCl was added to a final concentration of 200 mM, and the supernatant was incubated at 65°C for 8 h for decrosslinking. Next, 2 μl of RNase A (10 mg/ml) was added to the samples and incubated at 37°C for 30 min to digest the RNAs, and 5 μl of proteinase K (20 mg/ml) was added and incubated at 55°C for 1 h to digest the proteins. The DNA was purified with the FastGene Gel/PCR Extraction Kit (Nippon Genetics), in accordance with the manufacturer's protocol and eluted in nuclease-free water. DNA products from ChIP were amplified using the StepOnePlus Real-Time PCR System (Applied Biosystems) with PCR enzymes and the PowerUp SYBR Green Master Mix (Thermo Fisher Scientific) and qualified for ChIP-seq libraries. ChIP-seq libraries were prepared in accordance with the instructions accompanying the NEBNext Ultra II FS DNA Library Prep Kit for Illumina (New England BioLabs). In the adaptor ligation step, the original adaptor reagent was diluted 10-fold. After adapter ligation, the DNA was purified with AMPure XP beads (Beckman Coulter) for size selection. Then, the DNA was PCR-amplified with Illumina primers for 13–16 cycles and the library fragments of ~ 325 bp (insert plus adaptor and the PCR primer sequences) were purified with AMPure XP beads (Beckman Coulter). Duplicate samples were prepared for each condition. The purified DNA was captured on an Illumina flow cell for cluster generation. The libraries were sequenced on the Illumina HiSeq in accordance with the manufacturer's protocols. The ratio of the abundances of the peptides [control/L(3)mbt KD > 4] and the peptide spectrum matches (> 1) were used for candidate selection.

RNA-seq library preparation

The ribosomal RNAs were excluded from the total RNAs using a Ribo-Zero rRNA Removal Kit (Human/Mouse/Rat), in accordance with Illumina's protocol, and the RNA-seq libraries were prepared following the instructions accompanying the TruSeq Stranded mRNA Library Prep Kit (Illumina). Poly-A selected mRNAs were submitted for library preparation. Triplicate samples were prepared for each RNAi-treated condition. Libraries were sequenced on the Illumina HiSeq following the manufacturer's protocol.

Computational analysis of RNA-seq and ChIP-seq

For ChIP-seq analysis, the sequence reads were mapped to the genome of *Drosophila melanogaster* (dm6) using Bowtie 2 (Langmead & Salzberg, 2012). Only unique mapped reads were used in the analysis and PCR amplicons were excluded using Picard tools (https://broadinstitute.github.io/picard/). Peak calling was completed with MACS2 (Zhang *et al*, 2008) with default settings. From the output file of peak coordinate, the peaks with log10-converted *q*-value over −100 were removed. Pearson's correlation coefficient between L(3)mbt ChIP duplicate reads mapped on merged peaks was 0.983 and that between Lint-O ChIP duplicate reads mapped on merged peaks was 0.981. Common peaks, not merged peaks, between the duplicate samples were selected and used for further analysis. Peaks were annotated with in-house scripts according to dmel-all-r6.43.gtf (ftp://ftp.flybase.org/genomes/dmel/current/gtf/). For heatmaps and summary plots, ChIP scores were calculated by deeptools (Ramírez *et al*, 2016). Bigwig files of ChIP were produced by the bamCoverage program of deeptools and matrix data were calculated by the computeMatrix program of deeptools using bigwig files and plotted. Motif detection was performed using the peak bed files produced by MACS2 and findMotifsGenome.pl program of Homer (Heinz *et al*, 2010). For RNA-seq analysis, sequence reads mapped to rRNAs were excluded and the remaining reads were mapped to the genome of *Drosophila melanogaster* (dm6) using HISAT2 (Kim *et al*, 2015). Differential expression analysis was performed using the triplicate samples and DESeq2 package in R (Love *et al*, 2014) The resulting lists of differentially expressed genes [FDR (false discovery rate) < 0.01] were categorized into upregulated and downregulated genes by the fold change of the expression and applied for further analysis (Appendix Table S3). PCA analysis of RNA-seq samples of OSC was performed in R by using the dataset of reads for each gene normalized by million mapped reads. GO analysis of the biological process shown in Fig 6G was performed using clusterProfiler package in R (Yu *et al*, 2012; Wu *et al*, 2021).

Fly stock

The *yellow white* (*y^1 w^{1118}*) strain was used as a WT strain. The mutants used in this study were: *y^1 w^{1118}*, *y^1 w^{1118} Lint-O^{KO}/FM7, Kr*

© 2022 The Authors

> *GFP* (this study), *w*; L(3)mbt^{ts1}/TM6b* (gift from C. Gonzalez). A mutant allele of *Lint-O^{KO}* was generated using the transgenic CRISPR-Cas9 technique as previously described (Kondo & Ueda, 2013) The following strains were used for the mutagenesis: *y^1 v^1 nos-phiC31; attP40* host (NIG-FLY stock TBX-0002), *y^2 cho^2 v^1; Sp hs-hid/CyO* (NIG-FLY stock TBX-0009), *y^1 w^{1118}*; +; *attP2{nos-cas9}* (Kondo *et al*, 2020), and *Df(1)JA27/FM7c*, *P{w[+mC]=GAL4-Kr.C} DC1*, *P{w[+mC]=UAS-GFP S65T}DC5*, *sn[+]* (Bloomington Drosophila Stock Center #5193). Mutant files were validated by PCR and sequencing of the target region. Oligo DNA sequences are shown in Appendix Table S2. Genotypes of flies used in this study are shown in Appendix Table S4.

RNA extraction from flies and RT–qPCR

Total RNAs were extracted from ovaries using ISOGEN (Nippon Gene). RT–qPCR was performed as reported previously (Saito *et al*, 2009). In accordance with the manufacturer's instructions, 1 µg of total RNA from each sample was used to reverse-transcribe target sequences using a Transcriptor First Strand cDNA Synthesis Kit (Roche). The resulting cDNAs were amplified with a TB Green Premix Ex Taq II (Takara). The primers used are shown in Appendix Table S2.

Generation of the Lint-O-Venus strain

The *Lint-O-Venus* strain was generated by CRISPR/Cas9-mediated targeted transgene integration (Kondo *et al*, 2020). The gene encoding Venus fluorescent protein was inserted immediately in front of the stop codon of the *Lint-O* gene so that Lint-O-Venus was translated as a C-terminal fusion protein. The donor vector (pBS-Lint-Oarm-Venus-3xP3-dsRed-Express2) carried approximately 800 bp and 500 bp homology arms on the left and left sides of a knock-in cassette comprising genes encoding Venus and 3xP3-dsRed-Express2 flanked by loxP sites from pPV-RF3 (Kondo *et al*, 2020). The donor and gRNA vector (pBFv-U6.2-Lint-O-gRNA5) containing a guide RNA were co-injected into fertilized eggs laid by *nos*-Cas9 flies. Transformants were selected by eye-specific red fluorescence of the 3xP3-dsRed-Express2 transgene. Homozygous Lint-O-Venus strain was used for the microscopy analysis. DNA oligo sequences for plasmid vectors are shown in Appendix Table S2.

Female fertility assay

Adults after eclosion were immediately transferred to 29°C and incubated for 2 days. Single females were then incubated with three *y^1 w^{1118}* control males at 29°C for 5 days for mating. Ten independent crosses were performed. The number of adult offspring per cross was counted.

L3 larval brain

L3 larval brains were isolated from *y^1 w^{1118}*, *y^1 w^{1118} Lint-O^{KO}/FM7 Kr > GFP*, *y^1 w^{1118} Lint-O^{KO}*, and *L(3)mbt^{ts1}* grown at 29°C for 5–6 days after spawning at 25°C, and used for western blotting, volume calculation, immunostaining, and RNA-seq analysis. The orthogonal major and minor axes across the brain lobes were measured using an SZX16 stereo microscope and cellSens standard (Olympus). The volume (V) of brain lobes was calculated as $V = 4/3\pi ab^2$, where a is the major semiaxis and b is the minor semiaxis. Significance was calculated by the Student's t-test.

Data availability

OSC RNA-seq and ChIP-seq data are deposited at Gene Expression Omnibus (GSE) (GSE181802: https://www.ncbi.nlm.nih.gov/geo/query/acc.cgi?acc = GSE181802). Fly RNA-seq data are also available at GSE (GSE205541: https://www.ncbi.nlm.nih.gov/geo/query/acc.cgi?acc = GSE205541). LC–MS/MS data are deposited at PRIDE (PXD026945: http://proteomecentral.proteomexchange.org/cgi/GetDataset?ID = PXD026945).

Expanded View for this article is available online.

Acknowledgements
We thank K. Sato, Y.W. Iwasaki, and H. Ishizu for advice on bioinformatic analysis, and M. Ariura, N. Arakaki, and T. Miyoshi for technical assistance. We thank A. Gonzalez for sharing the *l(3)mbt* mutant flies. We thank members of the Saito and the Siomi laboratories, particularly K.M. Nishida for advice on producing antibodies and H. Siomi for helpful discussions. We also thank K. Shirahige for advice on ChIP-seq. This work was supported by grants from the Ministry of Education, Culture, Sports, Science and Technology (MEXT) of Japan to M.C.S. (19H05466), Y.F. (17H06096), and H.Y. (19H03175 and 21H05130), K.S. (18H02379 and 21H00236), K.M. (18H06056 and 20H03188), and partly from the Japan Agency for Medical Research and Development to H.Y. (PRIME; 17937210). H.Y.-M. was supported by the Japan Society for the Promotion of Science.

Author contributions
Hitomi Yamamoto-Matsuda: Data curation; investigation; writing – original draft; writing – review and editing. **Keita Miyoshi:** Funding acquisition; investigation; writing – review and editing. **Mai Moritoh:** Investigation; writing – review and editing. **Hikari Yoshitane:** Funding acquisition; investigation; methodology. **Yoshitaka Fukada:** Funding acquisition; methodology. **Kuniaki Saito:** Conceptualization; supervision; funding acquisition; writing – review and editing. **Soichiro Yamanaka:** Data curation; supervision; investigation; writing – original draft; writing – review and editing. **Mikiko, C Siomi:** Conceptualization; supervision; funding acquisition; writing – original draft; writing – review and editing.

In addition to the CRediT author contributions listed above, the contributions in detail are:
HY-M and MM performed biochemical experiments. HY-M and SY performed bioinformatic analysis. HY and YF performed mass spectrometric analysis. KM and KS produced *lint-O* mutant flies and analyzed them. HY-M, KM, KS, SY, and MCS conceived the project and designed the experiments. All authors contributed to the writing of the manuscript.

Disclosure and competing interest statement
The authors declare that they have no conflict of interest.

References

Bateman A (2019) UniProt: a worldwide hub of protein knowledge. *Nucleic Acids Res* 47: D506 – D515

© 2022 The Authors

Betschinger J, Mechtler K, Knoblich JA (2006) Asymmetric segregation of the tumor suppressor brat regulates self-renewal in *Drosophila* neural stem cells. *Cell* 124: 1241 – 1253

Blanchard DP, Georlette D, Antoszewski L, Botchan MR (2014) Chromatin reader L(3)mbt requires the Myb–MuvB/DREAM transcriptional regulatory complex for chromosomal recruitment. *Proc Natl Acad Sci U S A* 111: E4234 – E4243

Boccuni P, MacGrogan D, Scandura JM, Nimer SD (2003) The human L(3)MBT polycomb group protein is a transcriptional repressor and interacts physically and functionally with TEL (ETV6). *J Biol Chem* 278: 15412 – 15420

Bonasio R, Lecona E, Reinberg D (2010) MBT domain proteins in development and disease. *Semin Cell Dev Biol* 21: 221 – 230

Bortle KV, Ramos E, Takenaka N, Yang J, Wahi JE, Corces VG (2012) *Drosophila* CTCF tandemly aligns with other insulator proteins at the borders of H3K27me3 domains. *Genome Res* 22: 2176 – 2187

Brennecke J, Aravin AA, Stark A, Dus M, Kellis M, Sachidanandam R, Hannon GJ (2007) Discrete small RNA-generating loci as master regulators of transposon activity in *Drosophila*. *Cell* 128: 1089 – 1103

Coux RX, Teixeira FK, Lehmann R (2018) L(3)mbt and the LINT complex safeguard cellular identity in the *Drosophila* ovary. *Development* 145: dev160721

Czech B, Munafò M, Ciabrelli F, Eastwood EL, Fabry MH, Kneuss E, Hannon GJ (2018) piRNA-guided genome defense: from biogenesis to silencing. *Annu Rev Genet* 52: 131 – 157

Gateff E (1982) Cancer, genes, and development: the *Drosophila* case. *Adv Cancer Res* 37: 33 – 74

Gateff E, Löffler T, Wismar J (1993) A temperature-sensitive brain tumor suppressor mutation of *Drosophila melanogaster*: developmental studies and molecular localization of the gene. *Mech Dev* 41: 15 – 31

Georlette D, Ahn S, MacAlpine DM, Cheung E, Lewis PW, Beall EL, Bell SP, Speed T, Manak JR, Botchan MR (2007) Genomic profiling and expression studies reveal both positive and negative activities for the *Drosophila* Myb-MuvB/dREAM complex in proliferating cells. *Genes Dev* 21: 2880 – 2896

Goriaux C, Desset S, Renaud Y, Vaury C, Brasset E (2014) Transcriptional properties and splicing of the *flamenco* piRNA cluster. *EMBO Rep* 15: 411 – 418

Gunawardane LS, Saito K, Nishida KM, Miyoshi K, Kawamura Y, Nagami T, Siomi H, Siomi MC (2007) A slicer-mediated mechanism for repeat-associated siRNA 5′ end formation in *Drosophila*. *Science* 315: 1587 – 1590

Hauri S, Comoglio F, Seimiya M, Gerstung M, Glatter T, Hansen K, Aebersold R, Paro R, Gstaiger M, Beisel C (2016) A high-density map for navigating the human polycomb complexome. *Cell Rep* 17: 583 – 595

Heinz S, Benner C, Spann N, Bertolino E, Lin YC, Laslo P, Cheng JX, Murre C, Singh H, Glass CK (2010) Simple combinations of lineage-determining transcription factors prime *cis*-regulatory elements required for macrophage and B cell identities. *Mol Cell* 38: 576 – 589

Huttlin EL, Bruckner RJ, Paulo JA, Cannon JR, Ting L, Baltier K, Colby G, Gebreab F, Gygi MP, Parzen H *et al* (2017) Architecture of the human interactome defines protein communities and disease networks. *Nature* 545: 505 – 509

Iwasaki YW, Siomi MC, Siomi H (2015) PIWI-interacting RNA: its biogenesis and functions. *Annu Rev Biochem* 84: 405 – 433

Janic A, Mendizabal L, Llamazares S, Rossell D, Gonzalez C (2010) Ectopic expression of germline genes drives malignant brain tumor growth in *Drosophila*. *Science* 330: 1824 – 1827

Kalkhoven E, Teunissen H, Houweling A, Verrijzer CP, Zantema A (2002) The PHD type zinc finger is an integral part of the CBP acetyltransferase domain. *Mol Cell Biol* 22: 1961 – 1970

Kim CA, Bowie JU (2003) SAM domains: uniform structure, diversity of function. *Trends Biochem Sci* 28: 625 – 628

Kim D, Langmead B, Salzberg SL (2015) HISAT: a fast spliced aligner with low memory requirements. *Nat Methods* 12: 357 – 360

Klattenhoff C, Bratu DP, McGinnis-Schultz N, Koppetsch BS, Cook HA, Theurkauf WE (2007) *Drosophila* rasiRNA pathway mutations disrupt embryonic axis specification through activation of an ATR/Chk2 DNA damage response. *Dev Cell* 12: 45 – 55

Klenov MS, Sokolova OA, Yakushev EY, Stolyarenko AD, Mikhaleva EA, Lavrov SA, Gvozdev VA (2011) Separation of stem cell maintenance and transposon silencing functions of Piwi protein. *Proc Natl Acad Sci U S A* 108: 18760 – 18765

Kondo S, Ueda R (2013) Highly improved gene targeting by germline-specific Cas9 expression in *Drosophila*. *Genetics* 195: 715 – 721

Kondo S, Takahashi T, Yamagata N, Imanishi Y, Katow H, Hiramatsu S, Lynn K, Abe A, Kumaraswamy A, Tanimoto H (2020) Neurochemical organization of the *Drosophila* brain visualized by endogenously tagged neurotransmitter receptors. *Cell Rep* 30: 284 – 297.e5

Langmead B, Salzberg S (2012) Fast gapped-read alignment with Bowtie2. *Nat Methods* 9: 357 – 359

Lantz V, Chang JS, Horabin JI, Bopp D, Schedl P (1994) The *Drosophila orb* RNA-binding protein is required for the formation of the egg chamber and establishment of polarity. *Genes Dev* 8: 598 – 613

Leader DP, Krause SA, Pandit A, Davies SA, Dow JAT (2018) FlyAtlas 2: A new version of the *Drosophila melanogaster* expression atlas with RNA-Seq, miRNA-Seq and sex-specific data. *Nucleic Acids Res* 46: D809 – D815

Lewis PW, Beall EL, Fleischer TC, Georlette D, Link AJ, Botchan MR (2004) Identification of a *Drosophila* Myb–E2F2/RBF transcriptional repressor complex. *Genes Dev* 18: 2929 – 2940

Lin H, Yue L, Spradling AC (1994) The *Drosophila* fusome, a germline-specific organelle, contains membrane skeletal proteins and functions in cyst formation. *Development* 120: 947 – 956

Love MI, Huber W, Anders S (2014) Moderated estimation of fold change and dispersion for RNA-seq data with DESeq2. *Genome Biol* 15: 550

Malone CD, Brennecke J, Dus M, Stark A, McCombie WR, Sachidanandam R, Hannon GJ (2009) Specialized piRNA pathways act in germline and somatic tissues of the *Drosophila* ovary. *Cell* 137: 522 – 535

Meier K, Mathieu EL, Finkernagel F, Reuter LM, Scharfe M, Doehlemann G, Jarek M, Brehm A (2012) LINT, a novel dL(3)mbt-containing complex, represses malignant brain tumour signature genes. *PLoS Genet* 8: e1002676

Miyoshi K, Tsukumo H, Nagami T, Siomi H, Siomi MC (2005) Slicer function of *Drosophila* Argonautes and its involvement in RISC formation. *Genes Dev* 19: 2837 – 2848

Nishida KM, Iwasaki YW, Murota Y, Nagao A, Mannen T, Kato Y, Siomi H, Siomi MC (2015) Respective functions of two distinct Siwi complexes assembled during PIWI-interacting RNA biogenesis in *Bombyx* germ cells. *Cell Rep* 10: 193 – 203

Nye J, Buster DW, Rogers GC (2014) The use of cultured *Drosophila* cells for studying the microtubule cytoskeleton. *Methods Mol Biol* 1136: 81 – 101

Onishi R, Sato K, Murano K, Negishi L, Siomi H, Siomi MC (2020) Piwi suppresses transcription of Brahma-dependent transposons via Maelstrom in ovarian somatic cells. *Sci Adv* 6: eaaz7420

Onishi R, Yamanaka S, Siomi MC (2021) piRNA- and siRNA-mediated transcriptional repression in *Drosophila*, mice, and yeast: New insights and biodiversity. *EMBO Rep* 22: e53062

Ozata DM, Gainetdinov I, Zoch A, O'Carroll D, Zamore PD (2019) PIWI-interacting RNAs: small RNAs with big functions. *Nat Rev Genet* 20: 89 – 108

© 2022 The Authors

Ramírez F, Ryan DP, Grüning B, Bhardwaj V, Kilpert F, Richter AS, Heyne S, Dündar F, Manke T (2016) deepTools2: a next generation web server for deep-sequencing data analysis. *Nucleic Acids Res* 44: W160 – W165

Richter C, Oktaba K, Steinmann J, Müller J, Knoblich JA (2011) The tumour suppressor L(3)mbt inhibits neuroepithelial proliferation and acts on insulator elements. *Nat Cell Biol* 13: 1029 – 1039

Robinow S, White K (1991) Characterization and spatial distribution of the ELAV protein during *Drosophila melanogaster* development. *J Neurobiol* 22: 443 – 461

Ruohola H, Bremer KA, Baker D, Swedlow JR, Jan LY, Jan YN (1991) Role of neurogenic genes in establishment of follicle cell fate and oocyte polarity during oogenesis in *Drosophila*. *Cell* 66: 433 – 449

Saito K, Nishida KM, Mori T, Kawamura Y, Miyoshi K, Nagami T, Siomi H, Siomi MC (2006) Specific association of Piwi with rasiRNAs derived from retrotransposons and heterochromatic regions in the *Drosophila* genome. *Genes Dev* 20: 2214 – 2222

Saito K, Inagaki S, Mituyama T, Kawamura Y, Ono Y, Sakota E, Kotani H, Asai K, Siomi H, Siomi MC (2009) A regulatory circuit for *piwi* by the large Maf gene *traffic jam* in *Drosophila*. *Nature* 461: 1296 – 1299

Saito K, Ishizu H, Komai M, Kotani H, Kawamura Y, Nishida KM, Siomi H, Siomi MC (2010) Roles for the Yb body components Armitage and Yb in primary piRNA biogenesis in *Drosophila*. *Genes Dev* 24: 2493 – 2498

Sanchez R, Zhou MM (2011) The PHD finger: a versatile epigenome reader. *Trends Biochem Sci* 36: 364 – 372

Schüpbach T, Wieschaus E (1991) Female sterile mutations on the second chromosome of *Drosophila melanogaster*. II. Mutations blocking oogenesis or altering egg morphology. *Genetics* 129: 1119 – 1136

Shpiz S, Olovnikov I, Sergeeva A, Lavrov S, Abramov Y, Savitsky M, Kalmykova A (2011) Mechanism of the piRNA-mediated silencing of *Drosophila* telomeric retrotransposons. *Nucleic Acids Res* 39: 8703 – 8711

Sienski G, Dönertas D, Brennecke J (2012) Transcriptional silencing of transposons by Piwi and Maelstrom and its impact on chromatin state and gene expression. *Cell* 151: 964 – 980

Sumiyoshi T, Sato K, Yamamoto H, Iwasaki YW, Siomi H, Siomi MC (2016) Loss of *l(3)mbt* leads to acquisition of the ping-pong cycle in *Drosophila* ovarian somatic cells. *Genes Dev* 30: 1617 – 1622

Theurkauf WE, Alberts BM, Jan YN, Jongens TA (1993) A central role for microtubules in the differentiation of *Drosophila* oocytes. *Development* 118: 1169 – 1180

Vagin VV, Sigova A, Li C, Seitz H, Gvozdev V, Zamore PD (2006) A distinct small RNA pathway silences selfish genetic elements in the germline. *Science* 313: 320 – 324

Wang SH, Elgin SCR (2011) *Drosophila* Piwi functions downstream of piRNA production mediating a chromatin-based transposon silencing mechanism in female germ line. *Proc Natl Acad Sci U S A* 108: 21164 – 21169

Wismar J, Löffler T, Habtemichael N, Vef O, Geißen M, Zirwes R, Altmeyer W, Sass H, Gateff E (1995) The *Drosophila melanogaster* tumor suppressor gene *lethal(3)malignant brain tumor* encodes a proline-rich protein with a novel zinc finger. *Mech Dev* 53: 141 – 154

Wu T, Hu E, Xu S, Chen M, Guo P, Dai Z, Feng T, Zhou L, Tang W, Zhan L *et al* (2021) clusterProfiler 4.0: a universal enrichment tool for interpreting omics data. *Innovation* 2: 100141

Yamashiro H, Siomi MC (2018) PIWI-interacting RNA in *Drosophila*: biogenesis, transposon regulation, and beyond. *Chem Rev* 118: 4404 – 4421

Yashiro R, Murota Y, Nishida KM, Yamashiro H, Fujii K, Ogai A, Yamanaka S, Negishi L, Siomi H, Siomi MC (2018) Piwi nuclear localization and its regulatory mechanism in *Drosophila* ovarian somatic cells. *Cell Rep* 23: 3647 – 3657

Yin H, Lin H (2007) An epigenetic activation role of Piwi and a Piwi-associated piRNA in *Drosophila melanogaster*. *Nature* 450: 304 – 308

Yu G, Wang LG, Han Y, He QY (2012) clusterProfiler: an R package for comparing biological themes among gene clusters. *Omics* 16: 284 – 287

Zhang Y, Liu T, Meyer CA, Eeckhoute J, Johnson DS, Bernstein BE, Nusbaum C, Myers RM, Brown M, Li W *et al* (2008) Model-based analysis of ChIP-Seq (MACS). *Genome Biol* 9: R137

License: This is an open access article under the terms of the Creative Commons Attribution License, which permits use, distribution and reproduction in any medium, provided the original work is properly cited.

 © 2022 The Authors

Expanded View Figures

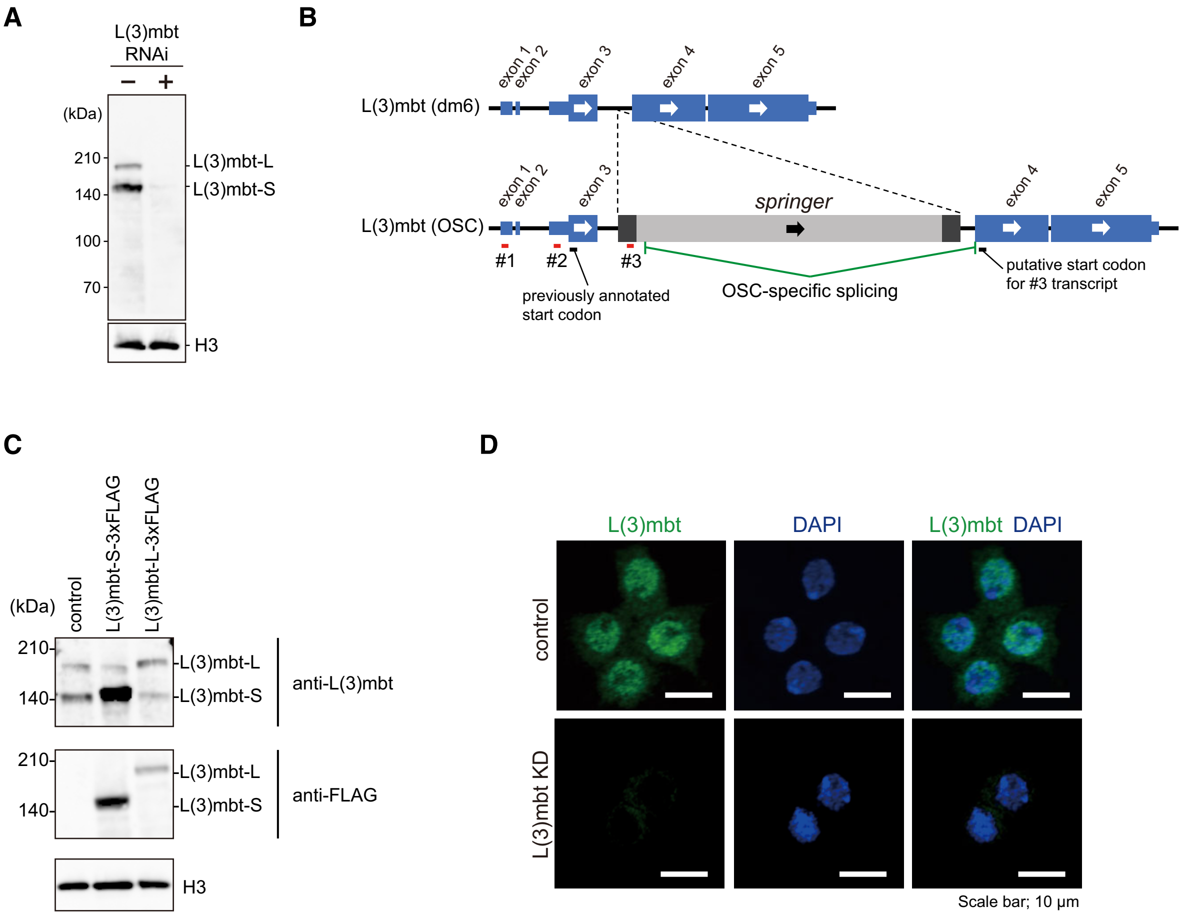

Figure EV1. Two isoforms of L(3)mbt, L(3)mbt-L, and L(3)mbt-S, in OSCs.

A Western blotting using an anti-L(3)mbt antibody that we raised in this study detected L(3)mbt as a doublet in the OSC nuclear lysates. The doublet disappeared upon L(3)mbt RNAi. Histone H3 (H3) served as a loading control.

B The exon-intron structures of the *l(3)mbt* gene in dm6 (provided by UCSC) and in OSCs (Sienski *et al*, 2012). Note that the *l(3)mbt* gene in OSCs has an LTR-type transposon, *springer*. RACE detected three different 5′ ends of *l(3)mbt* mRNA (#1, #2, and #3).

C Top: Western blotting using the anti-L(3)mbt antibody shows that L(3)mbt-L-3xFLAG and L(3)mbt-S-3xFLAG exogenously expressed in OSCs co-migrate with endogenous L(3)mbt-L and L(3)mbt-S (control), respectively. Middle: Western blotting was performed using anti-FLAG antibody. Bottom: Histone H3 (H3) was detected as a loading control.

D Immunofluorescence analysis using the anti-L(3)mbt antibody detected L(3)mbt (green) mostly in the OSC nuclei. The L(3)mbt signals disappeared upon L(3)mbt RNAi (KD). DAPI (blue) indicates the nuclei. Scale bar: 10 μm.

Source data are available online for this figure.

Figure EV2. Genomic distribution of L(3)mbt and the effects of L(3)mbt loss on the expression levels of piRNA factors in OSCs.

A Genomic browser views of L(3)mbt ChIP-seq signals. All fly chromosomes are shown. The *vasa*, *ago3*, and *aub* loci are indicated. The total number of L(3)mbt ChIP peaks is shown in upper right.

B PCA shows the correlation in the RNA-seq libraries. Control: GFP siRNA was used.

C–E The genomic regions harboring *qin*, *tej*, *boot*, and *CG9925* (C), *piwi*, *yb*, *armi*, and *zuc* (D), and *flam* (E).

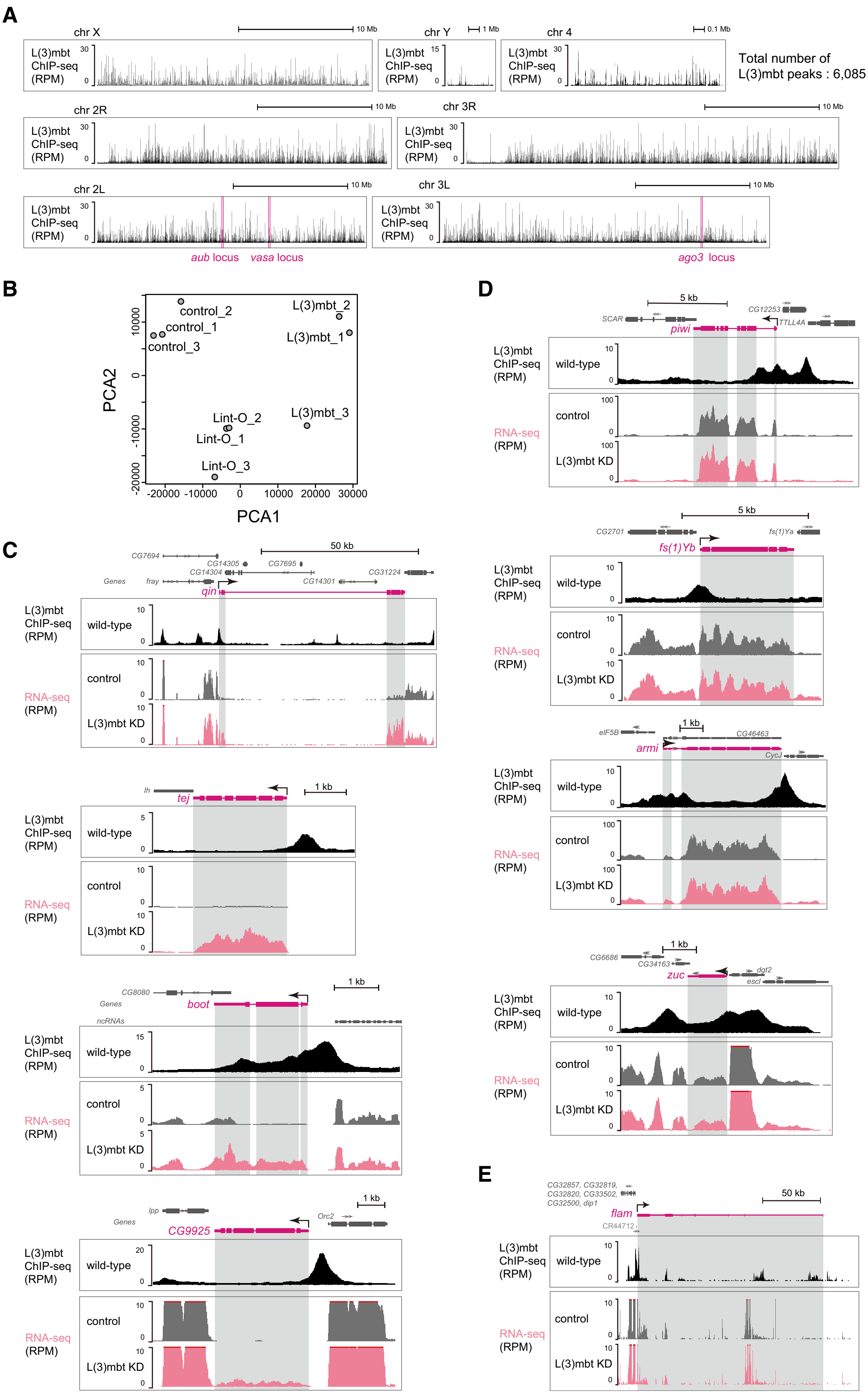

Figure EV2.

© 2022 The Authors

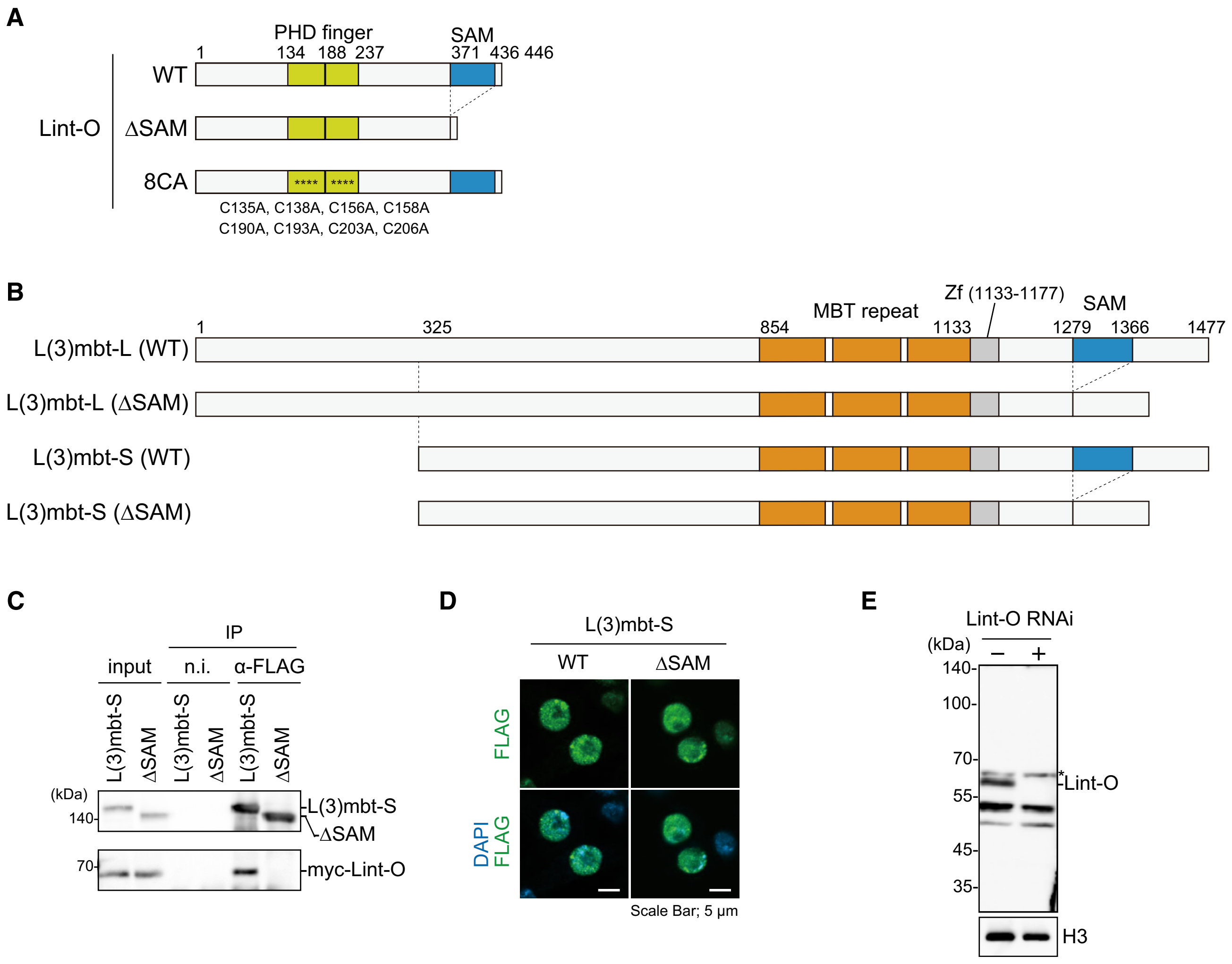

Figure EV3. Doman structures of L(3)mbt, Lint-O and their mutants and behavior of L(3)mbt-S in OSCs.

A Domain structures of WT Lint-O and its ΔSAM and 8CA mutants. The ΔSAM mutant is composed of Met1-Val371 and Ser437-Asp446. The Cys-to-Ala mutations in the 8CA mutant are indicated at the bottom (asterisks in the structure).

B Domain structures of WT L(3)mbt-L, WT L(3)mbt-S, and their ΔSAM mutants. The ΔSAM mutant of L(3)mbt-L is composed of Met1-Leu1278 and Val1367-Ser1477 of WT L(3)mbt-L. The ΔSAM mutant of L(3)mbt-S is composed of Met325-Leu1278 and Val1367-Ser1477 of WT L(3)mbt-L.

C IP/western blotting shows that WT L(3)mbt-S, but not its ΔSAM mutant, co-immunoprecipitated with Lint-O from the OSC lysates. n.i., nonimmune IgG.

D Subcellular localization of WT L(3)mbt-S and its ΔSAM mutant (green). Scale bar: 5 μm.

E Western blotting using the anti-Lint-O antibodies raised in this study. The Lint-O band (~60 kDa) was observed in normal OSCs (Lint-O RNAi−) but not in Lint-O-depleted OSCs (Lint-O RNAi+). Histone H3 (H3) was detected as a loading control. An asterisk shows the background.

Source data are available online for this figure.

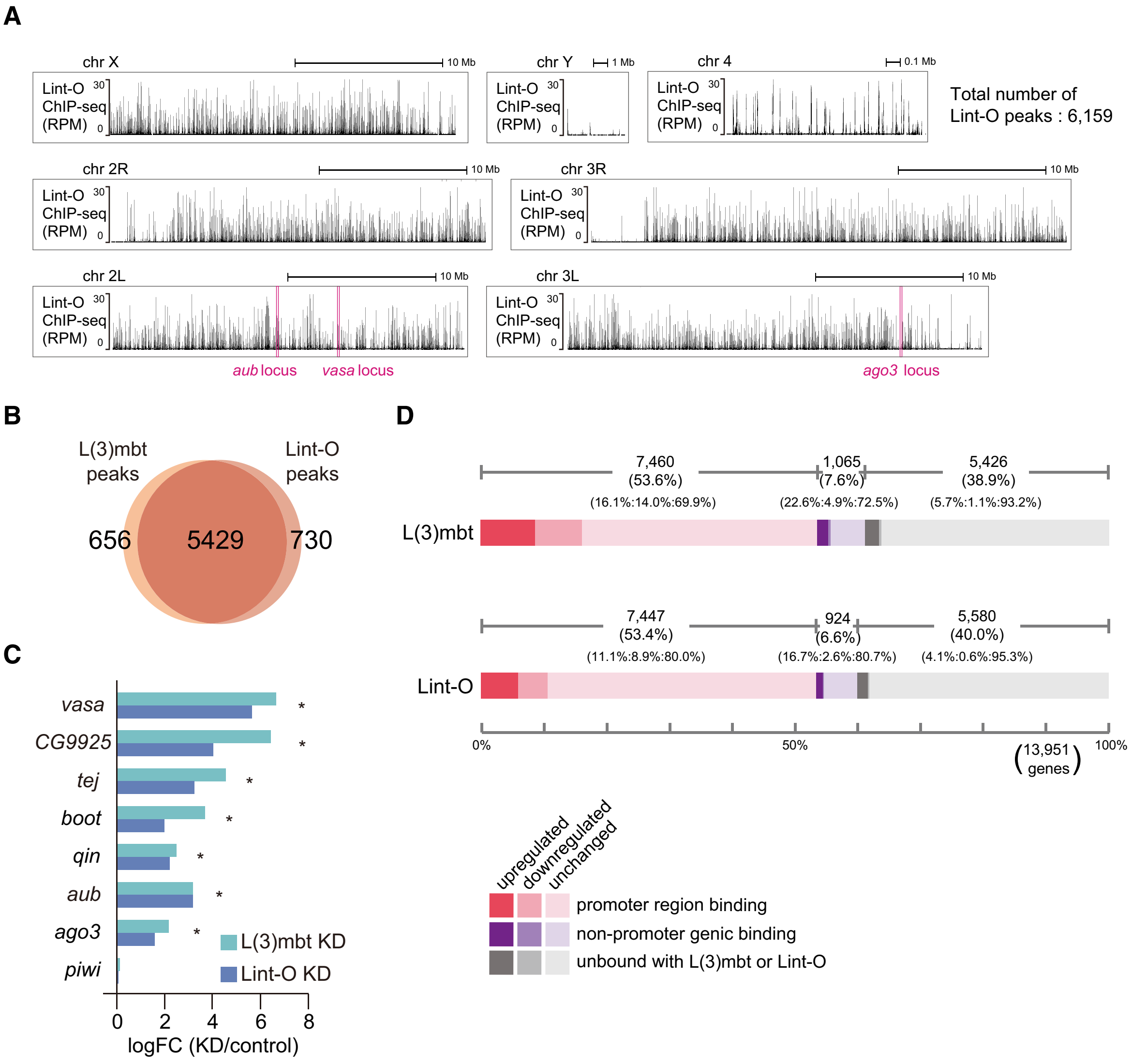

Figure EV4. Lint-O OSC ChIP-seq and RNA-seq before and after Lint-O depletion in OSCs.

A Genomic browser views of the Lint-O ChIP-seq reads. All fly chromosomes are shown. The *y*-axis shows the number of RPM. The *vasa*, *ago3*, and *aub* loci are indicated. The total number of Lint-O ChIP peaks is shown in upper right.

B Overlap between Lint-O ChIP peaks in (A) and L(3)mbt ChIP peaks (Fig EV2A).

C *vasa*, *CG9925*, *tej*, *boot*, *qin*, *aub*, and *ago3* were identified as upregulated genes (* *q*-value <0.05) in RNA-seq analysis. The log fold change of the differential expression for each piRNA pathway gene was calculated based on the RNA-seq data from normal (control), L(3)mbt-depleted (KD), and Lint-O-depleted (KD) OSCs. The *y*-axis shows the ratio of the read counts between L(3)mbt- and Lint-O-depleted OSCs and the normal OSCs.

D Overview and comparison of the gene classification in Figs 1A and 4A.

 © 2022 The Authors

Figure EV5. Lint-O ^{KO} ovaries and larval brains.

A Schema depicting the experiment. Eggs were transferred to 29°C after spawning at 25°C and incubated for 13 days. Ovaries were dissected from adult flies.

B–E Confocal images of brains of *y w*, *Lint-O^{KO}*, and *L(3)mbt^{ts1}* larvae (grown at 29°C) for Orb (B), Fas3 (C), Spectrin (Spec) (D), and F-actin (E). *Lint-O^{KO}* ovariole showed defects in follicle cell layer integrity. DAPI (blue): nuclei. Scale bars: 50 µm.

F The *Lint-O-Venus* fly line (*y w Lint-O-Venus*) was generated using the CRISPR /Cas9 system. The Venus sequence was inserted before the stop codon of Lint-O. The Lint-O-Venus signal (green) was detected in the nucleus of both germ and follicle cells in the ovaries. Nuclei were stained with DAPI (blue). Wild-type (*y w*) was used as a negative control. Scale bars: 50 µm.

G Confocal images of *y w*, *Lint-O^{KO}*, and *L(3)mbt^{ts1}* immunostained for Vasa (magenta) and MIRA (green). Merged images were also presented in Fig 6B. Scale bars: 100 µm.

H Enlarged views of insets in (G). Scale bars: 50 µm.

I Confocal images of *y w*, *Lint-O^{KO}*, and *L(3)mbt^{ts1}* immunostained for Vasa (magenta) and ELAV (green). Merged images were also presented in Fig 6B. Scale bars: 100 µm.

J Enlarged views of insets in (I). Scale bars: 100 µm.

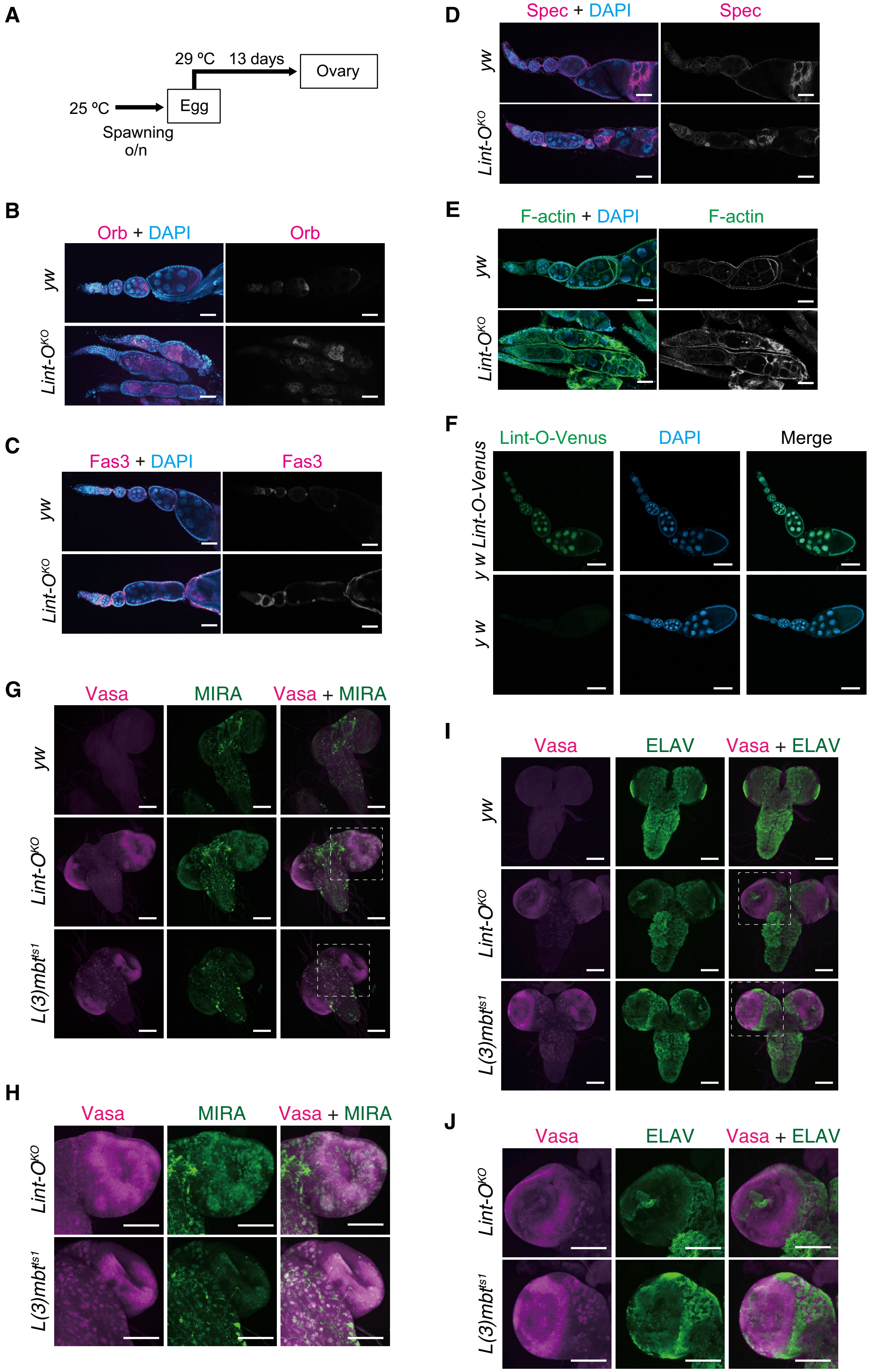

Figure EV5.

© 2022 The Authors

Appendix PDF

Table of Contents

**Appendix Figure S1**	Relationship between L(3)mbt ChIP peaks and TSS	 2
**Appendix Figure S2**	The mRNA levels of *AGO3* and *piwi* upon the depletion of L(3)mbt interactors in OSCs	 3
**Appendix Figure S3**	Genes bound and controlled by Lint-O	 4
**Appendix Table S1**	List of proteins in LC-MS/MS analysis	 5
**Appendix Table S2**	List of siRNAs and primers	 6
**Appendix Table S3**	List of genes differentially expressed in L(3)mbt KD OSC or Lint-O KD OSC	 9
**Appendix Table S4**	List of fly strains	 16

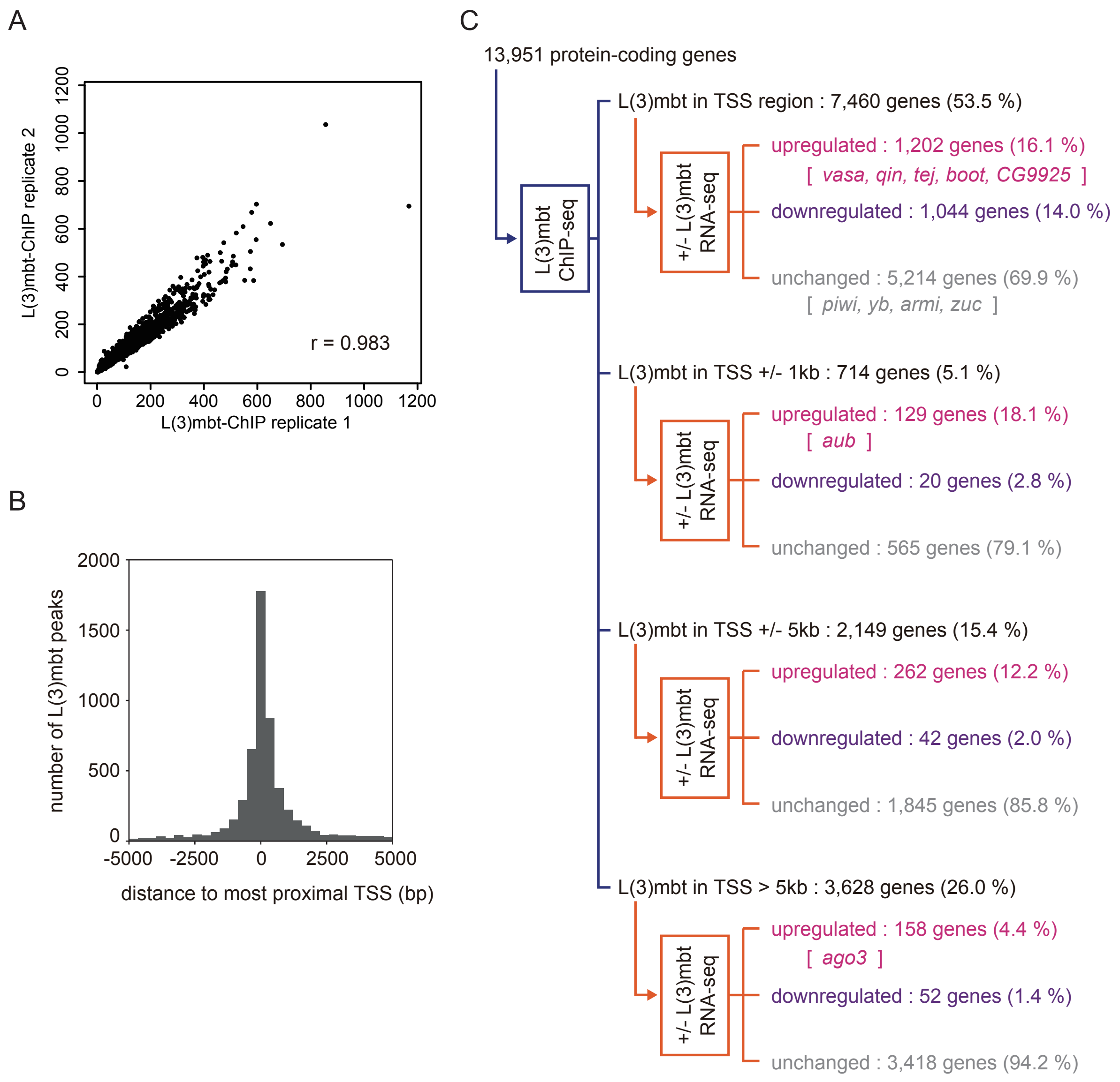

Appendix Figure S1. Relationship between L(3)mbt ChIP peaks and TSS.

(A) Scatter plot of sequenced reads, mapped onto each L(3)mbt ChIP peak defined by MACS2 for each replicate. Each dot denotes a detected ChIP peak. (B) Distribution of L(3)mbt ChIP peaks is shown according to the distance to most proximal TSS. (C) The 13,951 protein-coding genes of *Drosophila* were classified into "L (3)mbt in TSS region," "L(3)mbt in TSS +/- 1kb," "L(3)mbt in TSS +/- 5kb," and "L(3)mbt in TSS > 5kb," in accordance with the L(3)mbt ChIP-seq signals, and were subsequently divided into "upregulated," "downregulated," and "unchanged" in accordance with the RNA-seq reads from OSCs before and after L(3)mbt depletion [+/− L(3)mbt]. Representatives of piRNA factors are indicated within the groups. Note that the "L(3)mbt in TSS region" corresponds to the "promoter region binding" in Fig 1A.

A

L(3)mbt-containing complex	components
LINT	L(3)mbt, Lint-1, CoRest
dREAM/MMB	L(3)mbt, Myb, E2F2, RBF, p53, Mip120, Mip130, Mip140

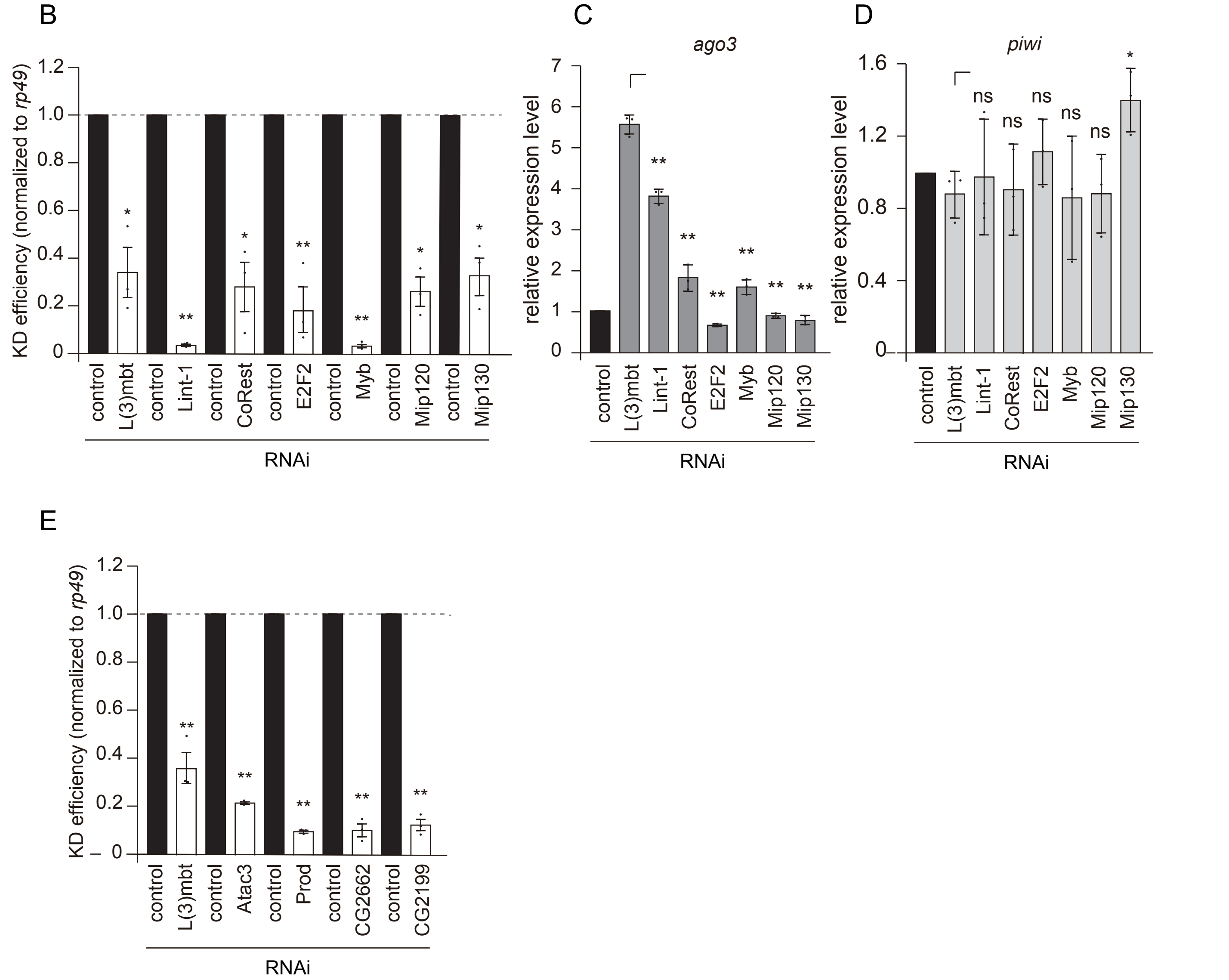

Appendix Figure S2. The mRNA levels of *ago3* and *piwi* upon the depletion of L(3)mbt interactors in OSCs.

(A) Known L(3)mbt cofactors within the LINT and dREAM complexes (Lewis et al. 2004; Meier et al. 2012; Blanchard et al, 2014). (B) Knockdown (KD) efficiencies of members of the LINT and dREAM complexes were verified by RT-qPCR. Control: EGFP siRNA was used. Data represent the mean ± SE (n = 3). The *p* values were calculated with the t-test. **p*: < 0.05, ***p*: < 0.01. (C, D) The mRNA levels of *ago3* (C) and *piwi* (D) were quantified in L(3)mbt-, Lint-1-, CoRest-, E2F2-, Myb-, Mip120-, and Mip130-depleted (RNAi) OSCs, and were compared with those in normal (control) OSCs. (E) KD efficiencies were verified by RT-qPCR for the indicated genes.

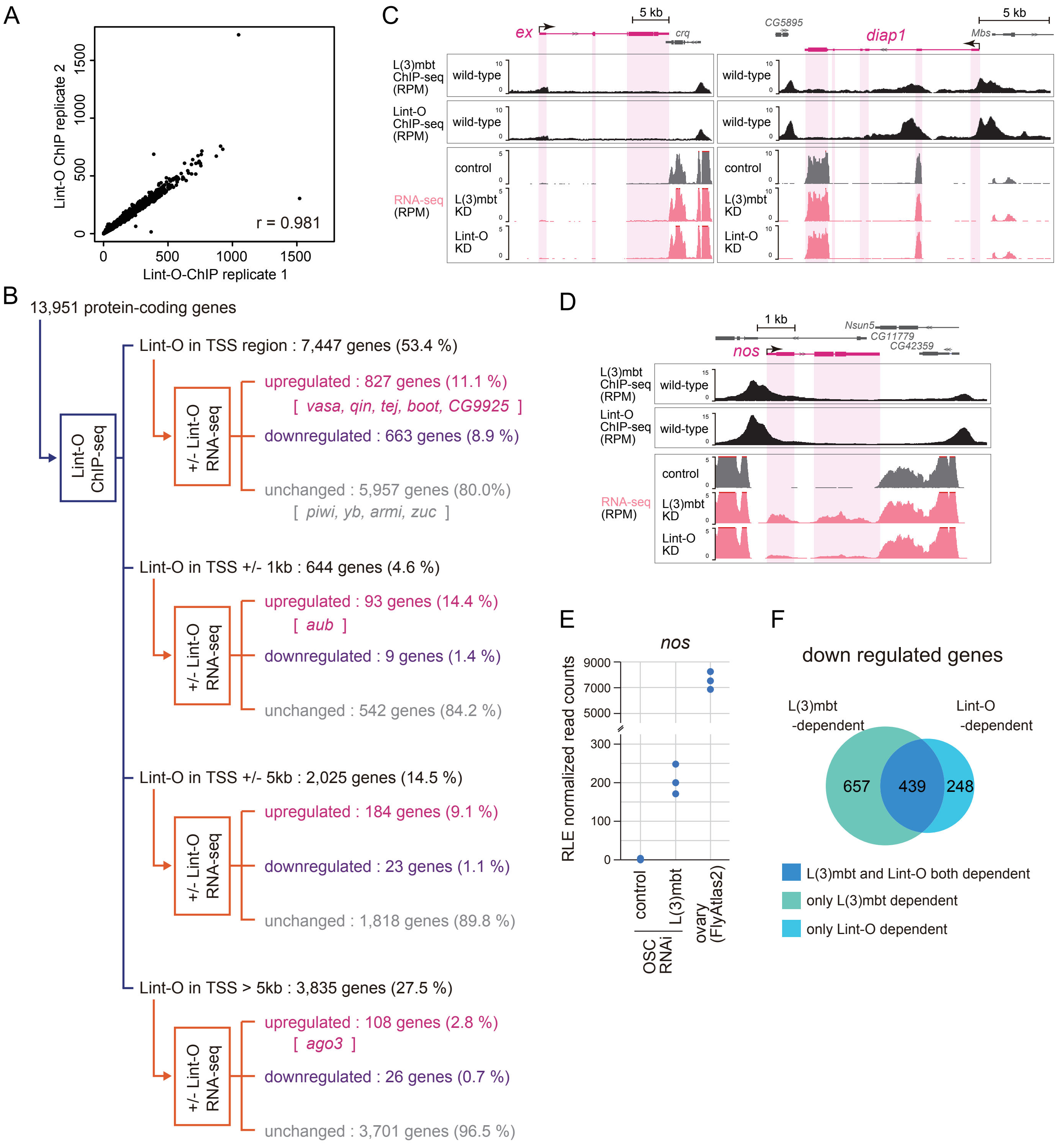

Appendix Figure S3. Genes bound and controlled by Lint-O.

(A) Scatter plot of sequenced reads, mapped onto each Lint-O ChIP peak defined by MACS2 for each replicate. Each dot denotes a detected ChIP peak. (B) The 13,951 protein-coding genes of Drosophila were classified into "Lint-O in TSS region," "Lint-O in TSS +/- 1kb," "Lint-O in TSS +/- 5kb," and "Lint-O in TSS > 5kb," in accordance with the Lint-O ChIP-seq reads, and were subsequently divided into "upregulated," "downregulated," and "unchanged" in accordance with the RNA-seq reads from OSCs before and after Lint-O depletion [+/− Lint-O]. Representatives of piRNA factors are indicated within the groups. Note that the "Lint-O in TSS region" corresponds to the "promoter region binding" in Fig 4A. (C, D) The genomic regions harboring the *diap1* (C), *ex* (C), and *nos* (D) genes. The L (3)mbt and Lint-O ChIP-seq reads in normal OSCs (wild-type) and RNA-seq reads from L(3)mbt- and Lint-O-depleted [L(3)mbt KD and Lint-O KD] and control OSCs are shown. The shades in gray correspond to exons. The *y*-axis shows the number of RPM. (E) The levels of *nos* mRNA in the ovary and cultured OSCs following L(3)mbt depletion are shown. (F) Venn diagram shows that 439 protein-coding genes are commonly present in the l(3)mbt and lint-O libraries as downregulated genes.

Appendix Table S1. List of proteins in LC/MS/MS analysis. excluded in first selection, excluded in second selection, excluded in third selection, and final selected proteins

				Abundances (control)		Abundances (L(3)mbt KD)		
Accession	Description	Sum PEP Scor	PSMs	1	2	1	2	FC of (abundances)
E8NH29	LD46071p (Fra	92.506	778	128.5	50.4	91.6	129.5	0.809136137
Q9VZ22	L(3)mbt intera	37.046	54	326	70.2	1.9	1.9	104.2631579
Q9VB52	LD05287p OS=	23.163	17	381.4	18.6	0	0	∞
E4NKN3	GM13963p OS=	17.992	61	247.3	68.8	32.5	51.5	3.763095238
L0MQ04	ATP synthase	16.843	25	39.2	281.2	38.5	41.1	4.025125628
O76866	EG:100G10.6 p	14.463	14	383.8	14.2	0	1.9	209.4736842
Q9VLK1	U3 small nucle	14.345	9	274.4	0	27.3	98.3	2.184713376
P56538	Eukaryotic tra	12.677	17	38.8	163.9	188.8	8.6	1.026849037
P29844	Endoplasmic r	12.448	13	168.8	90.3	85.5	55.4	1.838892832
Q9VJD1	Glucosidase 2	11.363	13	131.1	176.4	35.6	56.9	3.324324324
A0A0B4KHY6	Ets at 97D, iso	10.881	7	164.5	94.6	107.7	33.2	1.838892832
Q0ZHI2	L23A ribosoma	9.293	5	242.3	54.7	80.4	22.7	2.880698351
Q9VUZ0	Translocon-as	8.169	15	38.8	262.7	67.1	31.4	3.060913706
P17704	40S ribosomal	7.807	14	253	52.9	41.8	52.3	3.250797024
D2NUK9	Hoi-polloi, isof	7.659	18	174.1	161.8	21.2	42.9	5.24024961
A0A0B4LH50	Actin 87E, isof	7.612	18	3.1	193.1	121.8	82	0.962708538
P55830	40S ribosomal	7.37	8	93.7	223.6	28.3	54.4	3.836759371
M9NE89	Lamin, isoform	7.079	7	3.9	214.1	68	114	1.197802198
P50887	60S ribosomal	6.947	51	146.9	95.5	127.6	30	1.538071066
C0HKA1	40S ribosomal	6.738	7	130	136.5	103.4	30.1	1.996254682
Q06559	40S ribosomal	6.598	7	182.4	100.9	56.7	60	2.427592117
Q9VPQ2	DnaJ homolog	5.88	14	90.7	173	94.3	42	1.934702861
X2JF59	Thioredoxin pe	4.989	8	0	261.5	62	76.5	1.888086643
Q7JZ53	CG4866 OS=D	4.883	1	218.1	0	181.9	0	1.199010445
Q9VGN9	Probable ribos	4.666	2	205.6	0	194.4	0	1.057613169
P08736	Elongation fac	4.588	12	39.6	193.4	102.3	64.8	1.394374626
A0A023GQA5	Lethal (2) 37C	4.276	2	0	357.9	0	42.1	8.501187648
Q9VTP4	60S ribosomal	3.676	2	30.2	270.2	85.3	14.3	3.016064257
A0A0B4K6N1	40S ribosomal	3.528	1	400	0	0	0	∞
P11147	Heat shock 70	3.513	6	61.3	218.8	0	119.9	2.336113428
P46222	60S ribosomal	3.448	2	400	0	0	0	∞
H0RNL4	FI17108p1 (Fra	3.391	2	327.7	0	72.3	0	4.532503458
B5RIJ9	FI02875p (Frag	2.925	1	0	338.7	0	61.3	5.525285481
Q8STI6	RE53401p OS=	2.831	1	400	0	0	0	∞
P13469	DNA-binding p	2.563	1	0	0	0	0	-
Q8T479	AT12489p OS=	2.553	1	0	331.4	0	68.6	4.83090379
A0A0F7J3E9	MIP06027p1 O	2.524	2	225.9	53.4	90.1	30.5	2.315920398
Q9V597	60S ribosomal	2.51	1	195.5	0	204.5	0	0.95599022
P41374	Eukaryotic tra	2.424	1	63.9	259.1	0	77	4.194805195
A0A0B4KH25	Histone H2A C	2.41	6	193.5	85.5	79.8	41.2	2.305785124
Q7KLW6	LD06837p (Fra	2.318	1	0	400	0	0	∞
Q4V5X9	IP07888p OS=D	2.316	3	287.3	0	112.7	0	2.549245785
A0A023T5E7	GEO01108p1 C	2.224	2	123.8	114.4	86.7	75.1	1.472187886
P02283	Histone H2B C	2.166	10	238	41.1	75	45.9	2.308519438
O97183	GH07456p OS=	2.102	3	33.6	305.3	19.7	41.3	5.555737705
A0A0B4LG52	GEO08239p1 C	2.05	2	108.9	139.2	0	152	1.632236842
Q7JNE1	Non-histone c	1.992	2	0	346.7	0	53.3	6.504690432
Q08473	RNA-binding p	1.98	3	68.2	197.7	46.7	87.4	1.98284862
Q9VWD9	LD38919p OS=	1.957	2	213	187	0	0	∞
C6SV50	40S ribosomal	1.919	2	134.6	216.8	0	48.6	7.230452675
A8DY43	Uncharacterize	1.858	2	0	0	0	0	-
Q9VHG5	Insulator bindi	1.854	11	87.5	86.9	212.1	13.6	0.772707133
A9UN96	LD25519p OS=	1.853	1	400	0	0	0	∞
P54397	39 kDa FK506-	1.795	1	400	0	0	0	∞
P06603	Tubulin alpha-	1.781	1	56.1	0	343.9	0	0.163128817
Q7K5M2	GH05836p OS=	1.748	2	88.3	177.5	80.1	54.1	1.980625931
P09180	60S ribosomal	1.604	1	186.5	0	213.5	0	0.8735363
B7ZWP7	RH14172p (Fra	1.601	1	91	159.3	81.7	68	1.672010688
M9PFN1	RuvB-like heli	1.576	2	12.3	211.3	94.6	81.9	1.266855524
Q7KRI2	Longitudinals l	1.34	1	16.7	313.9	69.4	0	4.763688761
Q9VHX4	LD24679p OS=	1.314	3	27.4	127.3	134.1	111.2	0.630656339
Q8IR16	No on or off tr	1.28	2	305.8	0	94.2	0	3.246284501
C0HL66	Histone H3.3A	1.259	2	400	0	0	0	∞
Q7K4B2	CG7845, isofor	1.259	1	0	256.3	0	143.7	1.783576896
Q9VSW7	LD21662p OS=	1.228	2	0	355.3	0	44.7	7.948545861
Q9I7W5	Something abo	1.154	1	400	0	0	0	∞
Q9VQ29	Cytochrome b-	1.145	1	0	371.1	0	28.9	12.84083045
Q9V7A4	Integrin alpha-	1.136	4	400	0	0	0	∞
A8DYH1	Muscle wasted	1.073	14	22.8	332.2	11.6	33.4	7.888888889
Q9V3P3	LD45860p OS=	1.067	2	0	341.5	0	58.5	5.837606838
Q9VA91	40S ribosomal	1.049	1	400	0	0	0	∞
A0A0B4LFD9	GEO04460p1 C	1.044	1	364.3	0	35.7	0	10.20448179
A0A0B4KFV4	Uncharacterize	1.037	2	12.3	188.2	118.8	80.7	1.005012531
Q9VMT4	GH15768p OS=	0.998	5	0	0	400	0	0
M9PDW8	Uncharacterize	0.99	11	400	0	0	0	∞
I1V500	Supercoiling fa	0.961	1	33.9	294.2	71.9	0	4.563282337
Q9VVH5	AT13736p OS=	0.947	1	266.9	0	133.1	0	2.005259204
A0A0B4KF15	Mangetout, iso	0.942	5	400	0	0	0	∞
M9NFF9	Ada2a-contain	0.94	2	207.2	114.8	22.2	55.8	4.128205128
Q24156	Transcription f	0.937	2	84.5	56.9	232.6	25.9	0.547001934
Q94901	RNA-binding p	0.919	1	400	0	0	0	∞
B7Z073	SOSS complex	0.898	1	0	400	0	0	∞
J7K9E8	Sin3A (Fragme	0.852	1	0	35.2	0	364.8	0.096491228
Q5BI31	LD15043p OS=	0.839	2	0	115.5	180	104.5	0.405975395
Q9VLP4	Protein mitosh	0.838	1	400	0	0	0	∞
F0JAS9	TD01073p (Fra	0.828	8	0	0	400	0	0
Q9VNA3	GH21273p OS=	0.824	1	376.6	0	23.4	0	16.09401709
P26686	Serine-arginine	0.815	1	400	0	0	0	∞
Q95U54	GH06271p OS=	0.813	4	0	0	400	0	0
E1JHR0	Drongo, isofor	0.764	1	0	0	400	0	0
Q967T6	Gag protein OS	0.757	1	155.3	136.4	0	108.3	2.693444137
A1ZBL0	Tubulin beta c	0.757	2	0	196.1	0	203.9	0.961745954
M9Z4Q3	MIP26177p1 O	0.754	1	0	0	0	0	-
Q8MT08	Dynein regulat	0.738	1	0	400	0	0	∞
Q59E37	Uncharacterize	0.735	2	0	0	400	0	0
Q9W1R1	GH10609p OS=	0.725	1	141.1	0	258.9	0	0.544998069
Q9VVN4	ATR-interactin	0.719	1	0	305.1	0	94.9	3.214963119
Q86PC1	RE03224p OS=	0.68	1	0	0	400	0	0
P20007	Phosphoenolpy	0.675	9	0	0	400	0	0
C6SUW3	LD13662p OS=	0.644	1	0	240.9	0	159.1	1.514142049

Appendix Table S2. List of siRNAs and primers. RNA is shown in italics.

experiment	name	sense	antisense
RNAi in OSCs	siEGFP	*GGCAAGCUGACCCUGAAGU* TT	*ACUUCAGGGUCAGCUUGCC* TT
	siL(3)mbt	*CCUCUGGAGAUGUCCGUAA* TT	*UUACGGACAUCUCCAGAGG* TT
	siLint-1	*GCAGAAUCUCCACCAGUAA* TT	*UUACUGGUGGAGAUUCUGC* TT
	siCoRest	*AACGAGGAGUCUGAUAACGAU* TT	*AUCGUUAUCAGACUCCUCGUU* TT
	siE2F2	*CCAAAUCUUGGACGUACA* TT	*UGUACGUCCAAGAUUUUGG* TT
	siMyb	*GGAGAACGACCAUUCCAU* TT	*AUGGAAUGGUCGAUUCUCC* TT
	siMip120	*GUCAUCUUUGUCCAGAAGU* TT	*UAUGUGGACCACGUUCUUC* TT
	siMip130	*GGACUCCGUUUCCAUGCUA* TT	*UAGCAUGGAAACGGAGUCC* TT
	siAtac3	*CGAAUGGAUGUCCUAGAAA* UU	*UUUCUAGGACAUCCAUUCG* UU
	siProd	*CGAUUGGAAGAACACCACU* UU	*AGUGGUGUUCUUCCAAUCG* UU
	siCG2662(siLint-O)	*GAUUGAGAAGCUAGCCAAA* UU	*UUUGGCUAGCUUCUCAAUC* UU
	siCG2199	*CAGACGUACUGGAGACCAU* UU	*AUGGUCUCCAGUACGUCUG* UU
**experiment**	**name**	**forward primer (5' > 3')**	**reverse primer (5' > 3')**
RT-qPCR	L(3)mbt	AGGAGACGTCTTTCGCTTGG	AGGAGACGTCTTTCGCTTGG
	Lint-1	CGCCTCAACGATAGCCTACA	TATCACGAGCCATCACCGAC
	CoRest	CCAGTTCCACGGGAAGAGC	CTGGCTATGGATTTGTCTGGC
	E2F2	CCAAGGACCAAGAGAACTAC	ATACGACCTATTCGAGGGAT
	Myb	TTGAAGATATGGATCTGCGG	GACTCTTGTGTGGAGTTTCT
	Mip120	GAAACCAATACCCAGCAGTA	TCTGGACAAAGATGACTTGG
	Mip130	CTGCCAGATTAAGATCTCCC	AAGTTCTCCGACACAATCTC
	Atac3	CGATGGCACAAAGCTAGTCA	CTTGTCCCCTAAGCGAACTG
	Prod	ATGTCGAGGAGGATCCCCAA	ACGCGGCGCATTTTTAACTT
	CG2662 (siLint-O)	AAGTGTGGCAAAAGCGAACC	ACGTTGCATAACCGGCAGTA
	CG2199	TGCAAAAAGAGGTGCGATGC	ATGGGATGCTTCTGTGGGTG
	Vasa	AGCCGTCCAACGATGCA	AACATCGCTGCCGGTCA
	Aub	ACGGCATTTGTGACGAGTAG	ACGTCCACGAGCAATTACAG
	AGO3	CTGCATTTGTGGCCTCCATA	GGGGAGTTTGCCATTCCTTT
	Piwi	CAAGGCCGGATAATTGGACA	CCATCGCTCGGAGTGGTAAG
	control(heterochromatin)	CAGTTGATGGGATGAATTTGG	TGCCTGTGGTTCTATCCAAAC
	vasa promoter	TGAATGAATCACTTAGGTTGCTTG	TGGTGGAATTTCCCATTGTGC
	rp49	CCGCTTCAAGGGACAGTATCTG	ATCTCGCCGCAGTAAACGC
RT-qPCR using flies	rp49	CCGCTTCAAGGGACAGTATCTG	ATCTCGCCGCAGTAAACGC
	CG2662 (Lint-O)	CCAAGGACGAAGACAAGGAG	CGAGGGATCGTACACCTGTT

Sequence Validation of Lint-O fly mutant	CG2662gRNA1_seq	AACTGATCTCCTTATCCAACCGATGGAAGG	GCTGCTCCGAAACAGGACTGGTTGTGGAG
**experiment**	**name**	**sequence**	**vector**
Expression vector for OSC	L3mbt-ATGR	CCCGATCCGGGGTCTCTGG	pAcF-L(3)mbt-S-3xFLAG
	L3mbt-ATG4	ATGAGCCTAATTAAATCCAAGCCCG	pAcF-L(3)mbt-S-3xFLAG
	pAcM-Lint-O_F	AACAAGCTTGGTACCAAACAATCCAAGGACGAAGA	pAcM-Lint-O
	pAcM-Lint-O_R	GACTCGAGCGGCCGCCTAATCTAGCCAGGCGAGGC	pAcM-Lint-O
	pAcM_F	GCGGCCGCTCGAGTCTAGAG	pAcM
	pAcM_R	GGTACCAAGCTTGTTCAGGT	pAcM
	pAc-C3F_F	CTCGAGGACTACAAAGACCA	pAc-C3F_vector
	pAc-C3F_R	CATGGTGGCTAGCCCGATCC	pAc-C3F_vector
	pAc-L3mbt-3xFLAG_F	GGGCTAGCCACCATGCTGCCATTGTCGATGGCCAG	pAc-L3mbt-3xFLAG
	pAc-L3mbt-3xFLAG_R	TTTGTAGTCCTCGAGAGAGGACGTGCGCAAGGGCG	pAc-L3mbt-3xFLAG
	pAc-Lint-O-3xFLAG_F	GGGCTAGCCACCATGAAACAATCCAAGGACGAAGA	pAc-Lint-O-3xFLAG
	pAc-Lint-O-3xFLAG_R	TTTGTAGTCCTCGAGATCTAGCCAGGCGAGGCCAA	pAc-Lint-O-3xFLAG
	siRNA resistant_F	AAAATCGAAAAACTGGCTAAGCGTAGACAGCCCTT	pAc-Lint-O_siRNA_resistant
	siRNA resistant_R	CTTAGCCAGTTTTTCGATTTTCGCTGGCTTTTCTT	pAc-Lint-O_siRNA_resistant
	8CA1_vector_F	GCCCGGTTAGCCAACGTGAGGGGGCCA	pAc-Lint-O_8CA-3xFLAG
	8CA1_vector_R	GGCCTTCTGGGCCGTGTCGAAGTTGCG	pAc-Lint-O_8CA-3xFLAG
	8CA1_insert_F	CGGCCCAGAAGGCCGGCAAAAGCGAACCCA	pAc-Lint-O_8CA-3xFLAG
	8CA1_insert_R	GTTGGCTAACCGGGCGTAGCGACACTTGTC	pAc-Lint-O_8CA-3xFLAG
	8CA2_vector_F	GCCAGTTTAGCCGTGGATGCCTATCAC	pAc-Lint-O_8CA-3xFLAG
	8CA2_vector_R	GGCGCCCTTGGCAGTGAGGAAGTTGCT	pAc-Lint-O_8CA-3xFLAG
	8CA2_insert_F	ACTGCCAAGGGCGCCATGCAGAAATGGCAC	pAc-Lint-O_8CA-3xFLAG
	8CA2_insert_R	CACGGCTAAACTGGCAATGGAAAGACCTGG	pAc-Lint-O_8CA-3xFLAG
	Lint-O-ΔSAM-3xFLAG_F	AAGCCATCCATCCAATGACGTTGGCCTCGC	pAc-Lint-O-ΔSAM-3xFLAG
	Lint-O-ΔSAM-3xFLAG_R	CGTCATTGGATGGATGGCTTCTTCTCTCTA	pAc-Lint-O-ΔSAM-3xFLAG
	Lint-O-ΔPHD-3xFLAG_F	CTTCGCAACTTCGACGCGTGTAACATCGGC	pAc-Lint-O-ΔPHD-3xFLAG
	Lint-O-ΔPHD-3xFLAG_R	GCCGATGTTACACGCGTCGAAGTTGCGAAG	pAc-Lint-O-ΔPHD-3xFLAG
	L(3)mbt-L/S_ΔSAM_F	GGACCACAGTTCCTGGTCGTCTGCAAGTTTGATTC	pAcF-L(3)mbt-L/S_ΔSAM
	L(3)mbt-L/S_ΔSAM_R	AAACTTGCAGACGACCAGGAACTGTGGTCCATAGT	pAcF-L(3)mbt-L/S_ΔSAM

experiment	name	sequence	vector
Lint-O fly mutagenesis	gRNA_CG2662_1F	CTTCGACGACCCACAATTCCAGCGC	pBFv-U6.2-Lint-O-gRNA1
	gRNA_CG2662_1R	AAACGCGCTGGAATTGTGGGTCGTC	pBFv-U6.2-Lint-O-gRNA1
Lint-O-Venus transgenic fly construction	Lint-O gRNA5_F	CTTCGACATTAGGTGCTAAGGAGGT	pBFv-U6.2-Lint-O-gRNA5
	Lint-O gRNA5_R	AAACACCTCCTTAGCACCTAATGTC	pBFv-U6.2-Lint-O-gRNA5
	Lint-O 5'arm_F	GCTTGATATCGAATTCCTTGCCGATCACGTTCCACAAC	pBS-Lint-Oarm-Venus-3xP3-dsRed-Express2
	Lint-O 5'arm_R	AGTTGGGGGCGTAGGATCTAGCCAGGCGAGGCCAACGTC	pBS-Lint-Oarm-Venus-3xP3-dsRed-Express2
	Lint-O 3'arm_F	TAGTATAGGAACTTCCGTCCCCACCCACCTCCTTAGCAC	pBS-Lint-Oarm-Venus-3xP3-dsRed-Express2
	Lint-O 3'arm_R	CGGGCTGCAGGAATTC TAAAAGGCGTATTTTACTCCATC	pBS-Lint-Oarm-Venus-3xP3-dsRed-Express2

Appendix Table S3. Genes upregulated in L(3)mbt KD

FBgn0000053	FBgn0004919	FBgn0020391	FBgn0028916	FBgn0030742	FBgn0032105	FBgn0033431	FBgn0034988	FBgn0036503	FBgn0037853
FBgn0000071	FBgn0005390	FBgn0021800	FBgn0028983	FBgn0030778	FBgn0032123	FBgn0033438	FBgn0035019	FBgn0036536	FBgn0037885
FBgn0000084	FBgn0005558	FBgn0022160	FBgn0028984	FBgn0030793	FBgn0032135	FBgn0033446	FBgn0035032	FBgn0036565	FBgn0037896
FBgn0000108	FBgn0005631	FBgn0022359	FBgn0028996	FBgn0030816	FBgn0032161	FBgn0033448	FBgn0035034	FBgn0036574	FBgn0037902
FBgn0000158	FBgn0005660	FBgn0022702	FBgn0028997	FBgn0030841	FBgn0032178	FBgn0033451	FBgn0035035	FBgn0036612	FBgn0037921
FBgn0000180	FBgn0005696	FBgn0022709	FBgn0029002	FBgn0030847	FBgn0032192	FBgn0033453	FBgn0035056	FBgn0036622	FBgn0037930
FBgn0000246	FBgn0010014	FBgn0022768	FBgn0029094	FBgn0030864	FBgn0032210	FBgn0033459	FBgn0035073	FBgn0036640	FBgn0037950
FBgn0000289	FBgn0010097	FBgn0022770	FBgn0029105	FBgn0030869	FBgn0032211	FBgn0033468	FBgn0035086	FBgn0036641	FBgn0037999
FBgn0000299	FBgn0010226	FBgn0022960	FBgn0029131	FBgn0030876	FBgn0032213	FBgn0033486	FBgn0035094	FBgn0036652	FBgn0038016
FBgn0000317	FBgn0010246	FBgn0023023	FBgn0029137	FBgn0030884	FBgn0032221	FBgn0033538	FBgn0035146	FBgn0036687	FBgn0038037
FBgn0000320	FBgn0010263	FBgn0023094	FBgn0029147	FBgn0030937	FBgn0032228	FBgn0033554	FBgn0035151	FBgn0036689	FBgn0038038
FBgn0000346	FBgn0010314	FBgn0023097	FBgn0029508	FBgn0030941	FBgn0032233	FBgn0033555	FBgn0035157	FBgn0036698	FBgn0038042
FBgn0000422	FBgn0010317	FBgn0023171	FBgn0029514	FBgn0030955	FBgn0032249	FBgn0033627	FBgn0035167	FBgn0036732	FBgn0038045
FBgn0000463	FBgn0010342	FBgn0023179	FBgn0029573	FBgn0030968	FBgn0032294	FBgn0033654	FBgn0035169	FBgn0036738	FBgn0038047
FBgn0000533	FBgn0010382	FBgn0023395	FBgn0029606	FBgn0030994	FBgn0032297	FBgn0033686	FBgn0035181	FBgn0036765	FBgn0038053
FBgn0000536	FBgn0010391	FBgn0023509	FBgn0029694	FBgn0030997	FBgn0032364	FBgn0033715	FBgn0035265	FBgn0036773	FBgn0038105
FBgn0000557	FBgn0010395	FBgn0023515	FBgn0029708	FBgn0031002	FBgn0032382	FBgn0033748	FBgn0035273	FBgn0036801	FBgn0038115
FBgn0000588	FBgn0010407	FBgn0023520	FBgn0029710	FBgn0031018	FBgn0032400	FBgn0033749	FBgn0035321	FBgn0036810	FBgn0038144
FBgn0000615	FBgn0010441	FBgn0023537	FBgn0029712	FBgn0031037	FBgn0032402	FBgn0033753	FBgn0035325	FBgn0036813	FBgn0038191
FBgn0000633	FBgn0010470	FBgn0023540	FBgn0029722	FBgn0031053	FBgn0032428	FBgn0033767	FBgn0035390	FBgn0036815	FBgn0038250
FBgn0000636	FBgn0010482	FBgn0024177	FBgn0029723	FBgn0031066	FBgn0032447	FBgn0033785	FBgn0035407	FBgn0036820	FBgn0038251
FBgn0000644	FBgn0010602	FBgn0024236	FBgn0029733	FBgn0031091	FBgn0032452	FBgn0033799	FBgn0035410	FBgn0036821	FBgn0038252
FBgn0000658	FBgn0011206	FBgn0024320	FBgn0029737	FBgn0031104	FBgn0032474	FBgn0033807	FBgn0035415	FBgn0036822	FBgn0038256
FBgn0000721	FBgn0011225	FBgn0024366	FBgn0029752	FBgn0031116	FBgn0032475	FBgn0033814	FBgn0035434	FBgn0036824	FBgn0038273
FBgn0000996	FBgn0011274	FBgn0024432	FBgn0029754	FBgn0031148	FBgn0032484	FBgn0033815	FBgn0035445	FBgn0036875	FBgn0038277
FBgn0001104	FBgn0011286	FBgn0024732	FBgn0029765	FBgn0031161	FBgn0032519	FBgn0033846	FBgn0035452	FBgn0036896	FBgn0038299
FBgn0001120	FBgn0011293	FBgn0024913	FBgn0029791	FBgn0031170	FBgn0032520	FBgn0033876	FBgn0035471	FBgn0036927	FBgn0038311
FBgn0001128	FBgn0011606	FBgn0024920	FBgn0029823	FBgn0031171	FBgn0032521	FBgn0033890	FBgn0035476	FBgn0036929	FBgn0038312
FBgn0001137	FBgn0011660	FBgn0024984	FBgn0029830	FBgn0031188	FBgn0032598	FBgn0033903	FBgn0035477	FBgn0036956	FBgn0038316
FBgn0001142	FBgn0011661	FBgn0024991	FBgn0029834	FBgn0031190	FBgn0032603	FBgn0033913	FBgn0035497	FBgn0036958	FBgn0038321
FBgn0001222	FBgn0011704	FBgn0024995	FBgn0029843	FBgn0031191	FBgn0032652	FBgn0033921	FBgn0035505	FBgn0036969	FBgn0038349
FBgn0001224	FBgn0011705	FBgn0025382	FBgn0029861	FBgn0031220	FBgn0032666	FBgn0033960	FBgn0035515	FBgn0036980	FBgn0038419
FBgn0001225	FBgn0011722	FBgn0025390	FBgn0029867	FBgn0031228	FBgn0032680	FBgn0033987	FBgn0035521	FBgn0036984	FBgn0038431
FBgn0001226	FBgn0011761	FBgn0025638	FBgn0029892	FBgn0031232	FBgn0032698	FBgn0034013	FBgn0035523	FBgn0037000	FBgn0038452
FBgn0001229	FBgn0011766	FBgn0025640	FBgn0029911	FBgn0031239	FBgn0032706	FBgn0034051	FBgn0035539	FBgn0037023	FBgn0038453
FBgn0001230	FBgn0011770	FBgn0025641	FBgn0029924	FBgn0031245	FBgn0032713	FBgn0034067	FBgn0035575	FBgn0037050	FBgn0038467
FBgn0001233	FBgn0012037	FBgn0025682	FBgn0029930	FBgn0031252	FBgn0032727	FBgn0034075	FBgn0035578	FBgn0037060	FBgn0038476
FBgn0001248	FBgn0013305	FBgn0025693	FBgn0029937	FBgn0031254	FBgn0032775	FBgn0034081	FBgn0035587	FBgn0037063	FBgn0038490
FBgn0001311	FBgn0013548	FBgn0025743	FBgn0029942	FBgn0031255	FBgn0032779	FBgn0034091	FBgn0035623	FBgn0037086	FBgn0038540
FBgn0002567	FBgn0013563	FBgn0025814	FBgn0029970	FBgn0031260	FBgn0032797	FBgn0034095	FBgn0035645	FBgn0037092	FBgn0038550
FBgn0002773	FBgn0013726	FBgn0025837	FBgn0029974	FBgn0031263	FBgn0032798	FBgn0034106	FBgn0035677	FBgn0037107	FBgn0038575
FBgn0002838	FBgn0013753	FBgn0026085	FBgn0029975	FBgn0031264	FBgn0032800	FBgn0034117	FBgn0035696	FBgn0037130	FBgn0038581
FBgn0002878	FBgn0013763	FBgn0026176	FBgn0029977	FBgn0031313	FBgn0032813	FBgn0034162	FBgn0035710	FBgn0037182	FBgn0038596
FBgn0002924	FBgn0013764	FBgn0026239	FBgn0029993	FBgn0031315	FBgn0032815	FBgn0034187	FBgn0035711	FBgn0037183	FBgn0038608
FBgn0002937	FBgn0013770	FBgn0026257	FBgn0030003	FBgn0031317	FBgn0032817	FBgn0034198	FBgn0035719	FBgn0037220	FBgn0038612
FBgn0002948	FBgn0013773	FBgn0026263	FBgn0030009	FBgn0031320	FBgn0032821	FBgn0034199	FBgn0035751	FBgn0037225	FBgn0038613
FBgn0002962	FBgn0013810	FBgn0026374	FBgn0030011	FBgn0031381	FBgn0032850	FBgn0034221	FBgn0035767	FBgn0037234	FBgn0038638
FBgn0002973	FBgn0014031	FBgn0026562	FBgn0030051	FBgn0031384	FBgn0032864	FBgn0034255	FBgn0035770	FBgn0037238	FBgn0038679
FBgn0003016	FBgn0014133	FBgn0026592	FBgn0030099	FBgn0031413	FBgn0032881	FBgn0034269	FBgn0035825	FBgn0037239	FBgn0038682
FBgn0003046	FBgn0014143	FBgn0026593	FBgn0030207	FBgn0031451	FBgn0032884	FBgn0034270	FBgn0035829	FBgn0037254	FBgn0038690
FBgn0003091	FBgn0014269	FBgn0026616	FBgn0030218	FBgn0031460	FBgn0032889	FBgn0034299	FBgn0035847	FBgn0037295	FBgn0038691
FBgn0003114	FBgn0014342	FBgn0026872	FBgn0030230	FBgn0031484	FBgn0032895	FBgn0034304	FBgn0035904	FBgn0037296	FBgn0038815
FBgn0003162	FBgn0014857	FBgn0026874	FBgn0030245	FBgn0031489	FBgn0032897	FBgn0034312	FBgn0035907	FBgn0037315	FBgn0038830
FBgn0003250	FBgn0014869	FBgn0027108	FBgn0030290	FBgn0031490	FBgn0032900	FBgn0034335	FBgn0035918	FBgn0037338	FBgn0038878
FBgn0003301	FBgn0014931	FBgn0027280	FBgn0030313	FBgn0031520	FBgn0032906	FBgn0034371	FBgn0035936	FBgn0037354	FBgn0038912
FBgn0003353	FBgn0015283	FBgn0027329	FBgn0030347	FBgn0031571	FBgn0032907	FBgn0034411	FBgn0035943	FBgn0037364	FBgn0038916
FBgn0003371	FBgn0015519	FBgn0027348	FBgn0030362	FBgn0031630	FBgn0032946	FBgn0034417	FBgn0035944	FBgn0037436	FBgn0038925
FBgn0003390	FBgn0015520	FBgn0027491	FBgn0030396	FBgn0031636	FBgn0032955	FBgn0034420	FBgn0035945	FBgn0037469	FBgn0038928
FBgn0003391	FBgn0015558	FBgn0027494	FBgn0030412	FBgn0031637	FBgn0032957	FBgn0034425	FBgn0035957	FBgn0037518	FBgn0038975
FBgn0003423	FBgn0015600	FBgn0027500	FBgn0030421	FBgn0031645	FBgn0032986	FBgn0034436	FBgn0035959	FBgn0037525	FBgn0038978
FBgn0003435	FBgn0015602	FBgn0027550	FBgn0030432	FBgn0031646	FBgn0033015	FBgn0034452	FBgn0035964	FBgn0037562	FBgn0039002
FBgn0003462	FBgn0015622	FBgn0027552	FBgn0030457	FBgn0031677	FBgn0033020	FBgn0034500	FBgn0035977	FBgn0037578	FBgn0039044
FBgn0003527	FBgn0015625	FBgn0027560	FBgn0030459	FBgn0031678	FBgn0033031	FBgn0034583	FBgn0035995	FBgn0037579	FBgn0039058
FBgn0003567	FBgn0015664	FBgn0027585	FBgn0030468	FBgn0031716	FBgn0033033	FBgn0034592	FBgn0036031	FBgn0037581	FBgn0039108
FBgn0003655	FBgn0015773	FBgn0027588	FBgn0030478	FBgn0031717	FBgn0033048	FBgn0034606	FBgn0036106	FBgn0037584	FBgn0039110
FBgn0003733	FBgn0015789	FBgn0027600	FBgn0030484	FBgn0031740	FBgn0033101	FBgn0034614	FBgn0036121	FBgn0037618	FBgn0039114
FBgn0003888	FBgn0015801	FBgn0027654	FBgn0030485	FBgn0031741	FBgn0033134	FBgn0034628	FBgn0036144	FBgn0037623	FBgn0039125
FBgn0003950	FBgn0015924	FBgn0028379	FBgn0030501	FBgn0031762	FBgn0033159	FBgn0034643	FBgn0036147	FBgn0037654	FBgn0039129
FBgn0004055	FBgn0015926	FBgn0028408	FBgn0030508	FBgn0031775	FBgn0033174	FBgn0034647	FBgn0036168	FBgn0037661	FBgn0039180
FBgn0004108	FBgn0015929	FBgn0028425	FBgn0030520	FBgn0031816	FBgn0033177	FBgn0034694	FBgn0036194	FBgn0037664	FBgn0039189
FBgn0004133	FBgn0016036	FBgn0028434	FBgn0030545	FBgn0031829	FBgn0033188	FBgn0034709	FBgn0036202	FBgn0037669	FBgn0039251
FBgn0004167	FBgn0016038	FBgn0028490	FBgn0030551	FBgn0031844	FBgn0033192	FBgn0034718	FBgn0036206	FBgn0037670	FBgn0039265
FBgn0004360	FBgn0016054	FBgn0028523	FBgn0030555	FBgn0031850	FBgn0033204	FBgn0034720	FBgn0036224	FBgn0037679	FBgn0039277
FBgn0004379	FBgn0016075	FBgn0028525	FBgn0030574	FBgn0031874	FBgn0033212	FBgn0034725	FBgn0036272	FBgn0037690	FBgn0039286
FBgn0004397	FBgn0016122	FBgn0028540	FBgn0030597	FBgn0031886	FBgn0033214	FBgn0034750	FBgn0036279	FBgn0037715	FBgn0039356
FBgn0004400	FBgn0016123	FBgn0028546	FBgn0030600	FBgn0031896	FBgn0033215	FBgn0034753	FBgn0036288	FBgn0037721	FBgn0039504
FBgn0004432	FBgn0016131	FBgn0028561	FBgn0030607	FBgn0031905	FBgn0033233	FBgn0034797	FBgn0036298	FBgn0037736	FBgn0039507
FBgn0004456	FBgn0016715	FBgn0028563	FBgn0030629	FBgn0031910	FBgn0033234	FBgn0034804	FBgn0036330	FBgn0037747	FBgn0039559
FBgn0004581	FBgn0016794	FBgn0028572	FBgn0030630	FBgn0031920	FBgn0033261	FBgn0034816	FBgn0036354	FBgn0037749	FBgn0039561
FBgn0004607	FBgn0017577	FBgn0028579	FBgn0030647	FBgn0031952	FBgn0033274	FBgn0034850	FBgn0036368	FBgn0037754	FBgn0039564
FBgn0004611	FBgn0019985	FBgn0028582	FBgn0030657	FBgn0031975	FBgn0033312	FBgn0034880	FBgn0036381	FBgn0037759	FBgn0039622
FBgn0004623	FBgn0020257	FBgn0028622	FBgn0030660	FBgn0031986	FBgn0033373	FBgn0034885	FBgn0036391	FBgn0037779	FBgn0039640
FBgn0004650	FBgn0020294	FBgn0028671	FBgn0030683	FBgn0032006	FBgn0033374	FBgn0034935	FBgn0036397	FBgn0037807	FBgn0039644
FBgn0004797	FBgn0020312	FBgn0028675	FBgn0030706	FBgn0032010	FBgn0033381	FBgn0034943	FBgn0036405	FBgn0037841	FBgn0039651
FBgn0004882	FBgn0020372	FBgn0028743	FBgn0030716	FBgn0032013	FBgn0033382	FBgn0034950	FBgn0036433	FBgn0037844	FBgn0039665
FBgn0004885	FBgn0020385	FBgn0028746	FBgn0030721	FBgn0032029	FBgn0033388	FBgn0034956	FBgn0036461	FBgn0037846	FBgn0039666
FBgn0004913	FBgn0020389	FBgn0028862	FBgn0030723	FBgn0032079	FBgn0033402	FBgn0034957	FBgn0036486	FBgn0037849	FBgn0039667

FBgn0039681	FBgn0051140	FBgn0085223	FBgn0262719	FBgn0010453	FBgn0035168	FBgn0085483	FBgn0029856	FBgn0034279	FBgn0039911
FBgn0039688	FBgn0051141	FBgn0085236	FBgn0262738	FBgn0010548	FBgn0035234	FBgn0086704	FBgn0029863	FBgn0034406	FBgn0039915
FBgn0039702	FBgn0051145	FBgn0085317	FBgn0262820	FBgn0011289	FBgn0035260	FBgn0250816	FBgn0029986	FBgn0034413	FBgn0039927
FBgn0039714	FBgn0051156	FBgn0085319	FBgn0262937	FBgn0013576	FBgn0035264	FBgn0259101	FBgn0029987	FBgn0034476	FBgn0039932
FBgn0039737	FBgn0051195	FBgn0085382	FBgn0262985	FBgn0013767	FBgn0035282	FBgn0259108	FBgn0030005	FBgn0034530	FBgn0040338
FBgn0039752	FBgn0051217	FBgn0085384	FBgn0263022	FBgn0013995	FBgn0035308	FBgn0259111	FBgn0030028	FBgn0034538	FBgn0040350
FBgn0039755	FBgn0051220	FBgn0085405	FBgn0263023	FBgn0015032	FBgn0035724	FBgn0259163	FBgn0030073	FBgn0034662	FBgn0040697
FBgn0039774	FBgn0051248	FBgn0085438	FBgn0263029	FBgn0015569	FBgn0035865	FBgn0259226	FBgn0030234	FBgn0034761	FBgn0041233
FBgn0039827	FBgn0051251	FBgn0086254	FBgn0263076	FBgn0020277	FBgn0035903	FBgn0259927	FBgn0030244	FBgn0034920	FBgn0041710
FBgn0039844	FBgn0051344	FBgn0086356	FBgn0263106	FBgn0020415	FBgn0035917	FBgn0260003	FBgn0030310	FBgn0034942	FBgn0042179
FBgn0039848	FBgn0051370	FBgn0086357	FBgn0263111	FBgn0020513	FBgn0036040	FBgn0260942	FBgn0030326	FBgn0034972	FBgn0042696
FBgn0039883	FBgn0051373	FBgn0086361	FBgn0263143	FBgn0023524	FBgn0036195	FBgn0261016	FBgn0030510	FBgn0034973	FBgn0043043
FBgn0039896	FBgn0051431	FBgn0086384	FBgn0263234	FBgn0024941	FBgn0036282	FBgn0261446	FBgn0030596	FBgn0035104	FBgn0045980
FBgn0039900	FBgn0051436	FBgn0086408	FBgn0263235	FBgn0025616	FBgn0036364	FBgn0261563	FBgn0030653	FBgn0035286	FBgn0050076
FBgn0039916	FBgn0051475	FBgn0086450	FBgn0263240	FBgn0025702	FBgn0036479	FBgn0261859	FBgn0030691	FBgn0035317	FBgn0050418
FBgn0040009	FBgn0051516	FBgn0086690	FBgn0263258	FBgn0025878	FBgn0036568	FBgn0261996	FBgn0030697	FBgn0035348	FBgn0050460
FBgn0040070	FBgn0051523	FBgn0086691	FBgn0263260	FBgn0026061	FBgn0036727	FBgn0262029	FBgn0030725	FBgn0035412	FBgn0051002
FBgn0040096	FBgn0051548	FBgn0086695	FBgn0263355	FBgn0026144	FBgn0036768	FBgn0262057	FBgn0030748	FBgn0035490	FBgn0051017
FBgn0040102	FBgn0051619	FBgn0086898	FBgn0263395	FBgn0026439	FBgn0036769	FBgn0262477	FBgn0030846	FBgn0035504	FBgn0051321
FBgn0040153	FBgn0051636	FBgn0086909	FBgn0263397	FBgn0026721	FBgn0036806	FBgn0262509	FBgn0030859	FBgn0035571	FBgn0051464
FBgn0040308	FBgn0051658	FBgn0087002	FBgn0263601	FBgn0027111	FBgn0036816	FBgn0262531	FBgn0031004	FBgn0035880	FBgn0051719
FBgn0040321	FBgn0051674	FBgn0087007	FBgn0263974	FBgn0027356	FBgn0036978	FBgn0262624	FBgn0031005	FBgn0035941	FBgn0051997
FBgn0040322	FBgn0051718	FBgn0087035	FBgn0264305	FBgn0027596	FBgn0037563	FBgn0262717	FBgn0031080	FBgn0035985	FBgn0052006
FBgn0040333	FBgn0051720	FBgn0243486	FBgn0264442	FBgn0027657	FBgn0037678	FBgn0262867	FBgn0031155	FBgn0036131	FBgn0052037
FBgn0040348	FBgn0051789	FBgn0250732	FBgn0264443	FBgn0028491	FBgn0037714	FBgn0262871	FBgn0031157	FBgn0036145	FBgn0052073
FBgn0040351	FBgn0051812	FBgn0250848	FBgn0264832	FBgn0028499	FBgn0037750	FBgn0263776	FBgn0031184	FBgn0036208	FBgn0052354
FBgn0040382	FBgn0051955	FBgn0259140	FBgn0264897	FBgn0028899	FBgn0037751	FBgn0264386	FBgn0031196	FBgn0036264	FBgn0052364
FBgn0040384	FBgn0051974	FBgn0259143	FBgn0264953	FBgn0030001	FBgn0037906	FBgn0264489	FBgn0031257	FBgn0036287	FBgn0052461
FBgn0040465	FBgn0051989	FBgn0259168	FBgn0265052	FBgn0030085	FBgn0037916	FBgn0264502	FBgn0031337	FBgn0036410	FBgn0052580
FBgn0040477	FBgn0051999	FBgn0259173	FBgn0265182	FBgn0030148	FBgn0037972	FBgn0264815	FBgn0031343	FBgn0036419	FBgn0052783
FBgn0040507	FBgn0052068	FBgn0259185	FBgn0265262	FBgn0030271	FBgn0038017	FBgn0264895	FBgn0031548	FBgn0036428	FBgn0053481
FBgn0040524	FBgn0052082	FBgn0259195	FBgn0265276	FBgn0030309	FBgn0038071	FBgn0265045	FBgn0031632	FBgn0036454	FBgn0058470
FBgn0040551	FBgn0052088	FBgn0259224	FBgn0265575	FBgn0030357	FBgn0038076	FBgn0265274	FBgn0031689	FBgn0036547	FBgn0063923
FBgn0040666	FBgn0052177	FBgn0259238	FBgn0265598	FBgn0030367	FBgn0038079	FBgn0265726	FBgn0031715	FBgn0036575	FBgn0065110
FBgn0040670	FBgn0052190	FBgn0259242	FBgn0265725	FBgn0030467	FBgn0038088	FBgn0266129	FBgn0031737	FBgn0036752	FBgn0069969
FBgn0040752	FBgn0052195	FBgn0259683	FBgn0266000	FBgn0030590	FBgn0038290	FBgn0266756	FBgn0031746	FBgn0036877	FBgn0085245
FBgn0040759	FBgn0052280	FBgn0259685	FBgn0266101	FBgn0030734	FBgn0038330	FBgn0267339	FBgn0031751	FBgn0036891	FBgn0085261
FBgn0040823	FBgn0052313	FBgn0259714	FBgn0266418	FBgn0030746	FBgn0038420	FBgn0283451	FBgn0031770	FBgn0036910	FBgn0085298
FBgn0040837	FBgn0052365	FBgn0259734	FBgn0266421	FBgn0030798	FBgn0038492	FBgn0284435	FBgn0031869	FBgn0036979	FBgn0085400
FBgn0040900	FBgn0052368	FBgn0259740	FBgn0266465	FBgn0030993	FBgn0038602	FBgn0000044	FBgn0031974	FBgn0036993	FBgn0085409
FBgn0041102	FBgn0052373	FBgn0259745	FBgn0266521	FBgn0030998	FBgn0038652	FBgn0000046	FBgn0031998	FBgn0036997	FBgn0085417
FBgn0041180	FBgn0052436	FBgn0259791	FBgn0266709	FBgn0031097	FBgn0038828	FBgn0000146	FBgn0032048	FBgn0037028	FBgn0085427
FBgn0041186	FBgn0052476	FBgn0260388	FBgn0266719	FBgn0031195	FBgn0038881	FBgn0000153	FBgn0032058	FBgn0037042	FBgn0085428
FBgn0041205	FBgn0052483	FBgn0260399	FBgn0266757	FBgn0031240	FBgn0039094	FBgn0000473	FBgn0032082	FBgn0037222	FBgn0085475
FBgn0041582	FBgn0052485	FBgn0260462	FBgn0266801	FBgn0031414	FBgn0039099	FBgn0000826	FBgn0032140	FBgn0037276	FBgn0087040
FBgn0041706	FBgn0052506	FBgn0260653	FBgn0267698	FBgn0031515	FBgn0039201	FBgn0001090	FBgn0032225	FBgn0037304	FBgn0250869
FBgn0041723	FBgn0052549	FBgn0260743	FBgn0267792	FBgn0031634	FBgn0039415	FBgn0001216	FBgn0032252	FBgn0037347	FBgn0250904
FBgn0042178	FBgn0052557	FBgn0260766	FBgn0267828	FBgn0031745	FBgn0039500	FBgn0001259	FBgn0032253	FBgn0037375	FBgn0250907
FBgn0042185	FBgn0052579	FBgn0260795	FBgn0267967	FBgn0031894	FBgn0039503	FBgn0003137	FBgn0032322	FBgn0037513	FBgn0259196
FBgn0043001	FBgn0052625	FBgn0260874	FBgn0283440	FBgn0031961	FBgn0039594	FBgn0003187	FBgn0032350	FBgn0037515	FBgn0259682
FBgn0043575	FBgn0052626	FBgn0260932	FBgn0283442	FBgn0031973	FBgn0039734	FBgn0003292	FBgn0032374	FBgn0037659	FBgn0259977
FBgn0043792	FBgn0052676	FBgn0260933	FBgn0283509	FBgn0032003	FBgn0039789	FBgn0003308	FBgn0032375	FBgn0037683	FBgn0260386
FBgn0043900	FBgn0052677	FBgn0260943	FBgn0283545	FBgn0032040	FBgn0039886	FBgn0003312	FBgn0032601	FBgn0037835	FBgn0260430
FBgn0044871	FBgn0052679	FBgn0260986	FBgn0284221	FBgn0032055	FBgn0039897	FBgn0003486	FBgn0032660	FBgn0037915	FBgn0260446
FBgn0045064	FBgn0052706	FBgn0260991	FBgn0284243	FBgn0032078	FBgn0040071	FBgn0003886	FBgn0032683	FBgn0037934	FBgn0260658
FBgn0045823	FBgn0052813	FBgn0261041	FBgn0284408	FBgn0032218	FBgn0040091	FBgn0003889	FBgn0032774	FBgn0037960	FBgn0260746
FBgn0045842	FBgn0052814	FBgn0261053	FBgn0284442	FBgn0032262	FBgn0040260	FBgn0003890	FBgn0032783	FBgn0037973	FBgn0260780
FBgn0046258	FBgn0052982	FBgn0261085	FBgn0286222	FBgn0032345	FBgn0040349	FBgn0004118	FBgn0032788	FBgn0037974	FBgn0261393
FBgn0046763	FBgn0053017	FBgn0261086	FBgn0286507	FBgn0032381	FBgn0040658	FBgn0004512	FBgn0032803	FBgn0038067	FBgn0261561
FBgn0046878	FBgn0053062	FBgn0261243	FBgn0286778	FBgn0032422	FBgn0041096	FBgn0004878	FBgn0032820	FBgn0038172	FBgn0261999
FBgn0050015	FBgn0053087	FBgn0261261	FBgn0286788	FBgn0032473	FBgn0041234	FBgn0005666	FBgn0032910	FBgn0038247	FBgn0262473
FBgn0050016	FBgn0053099	FBgn0261262	FBgn0287186	FBgn0032726	FBgn0042133	FBgn0010105	FBgn0032973	FBgn0038260	FBgn0262607
FBgn0050069	FBgn0053108	FBgn0261341	FBgn0287695	FBgn0032749	FBgn0043806	FBgn0010241	FBgn0033051	FBgn0038261	FBgn0262686
FBgn0050085	FBgn0053156	FBgn0261363	FBgn0287767	FBgn0032752	FBgn0050115	FBgn0010258	FBgn0033079	FBgn0038279	FBgn0262794
FBgn0050089	FBgn0053177	FBgn0261385	FBgn0287831	FBgn0033096	FBgn0050195	FBgn0010473	FBgn0033115	FBgn0038292	FBgn0262814
FBgn0050091	FBgn0053207	FBgn0261445	FBgn0000075	FBgn0033203	FBgn0050485	FBgn0011296	FBgn0033132	FBgn0038315	FBgn0263132
FBgn0050106	FBgn0053229	FBgn0261549	FBgn0000244	FBgn0033222	FBgn0050502	FBgn0011828	FBgn0033221	FBgn0038373	FBgn0263772
FBgn0050151	FBgn0053289	FBgn0261584	FBgn0000256	FBgn0033252	FBgn0051161	FBgn0013348	FBgn0033229	FBgn0038397	FBgn0264542
FBgn0050156	FBgn0053468	FBgn0261625	FBgn0000477	FBgn0033421	FBgn0051202	FBgn0013813	FBgn0033257	FBgn0038407	FBgn0264748
FBgn0050289	FBgn0053469	FBgn0261649	FBgn0000927	FBgn0033504	FBgn0051231	FBgn0014018	FBgn0033268	FBgn0038435	FBgn0264894
FBgn0050296	FBgn0053494	FBgn0261673	FBgn0001263	FBgn0033610	FBgn0051279	FBgn0014395	FBgn0033387	FBgn0038603	FBgn0264979
FBgn0050359	FBgn0053542	FBgn0261794	FBgn0001991	FBgn0033657	FBgn0051706	FBgn0014417	FBgn0033494	FBgn0038740	FBgn0265048
FBgn0050379	FBgn0053556	FBgn0261952	FBgn0002778	FBgn0033872	FBgn0051721	FBgn0020307	FBgn0033518	FBgn0038784	FBgn0265767
FBgn0050380	FBgn0053653	FBgn0261955	FBgn0003028	FBgn0033963	FBgn0052036	FBgn0020377	FBgn0033519	FBgn0038814	FBgn0266534
FBgn0050383	FBgn0053679	FBgn0261985	FBgn0003149	FBgn0034070	FBgn0052703	FBgn0020379	FBgn0033520	FBgn0038894	FBgn0266566
FBgn0050403	FBgn0053775	FBgn0261987	FBgn0003159	FBgn0034094	FBgn0052792	FBgn0020414	FBgn0033580	FBgn0038957	FBgn0267408
FBgn0050414	FBgn0053792	FBgn0262035	FBgn0003174	FBgn0034247	FBgn0052815	FBgn0023090	FBgn0033628	FBgn0038959	FBgn0285896
FBgn0050424	FBgn0053926	FBgn0262101	FBgn0003447	FBgn0034278	FBgn0052816	FBgn0023416	FBgn0033631	FBgn0039006	FBgn0286071
FBgn0050438	FBgn0053969	FBgn0262364	FBgn0003460	FBgn0034437	FBgn0052944	FBgn0023495	FBgn0033658	FBgn0039015	FBgn0286785
FBgn0050440	FBgn0054012	FBgn0262508	FBgn0003475	FBgn0034489	FBgn0053003	FBgn0024189	FBgn0033659	FBgn0039051	FBgn0287209
FBgn0050466	FBgn0054021	FBgn0262515	FBgn0003719	FBgn0034497	FBgn0053257	FBgn0024836	FBgn0033763	FBgn0039073	
FBgn0050484	FBgn0054033	FBgn0262524	FBgn0003867	FBgn0034602	FBgn0053519	FBgn0026255	FBgn0033773	FBgn0039178	
FBgn0051021	FBgn0058006	FBgn0262559	FBgn0004047	FBgn0034639	FBgn0054031	FBgn0026376	FBgn0033875	FBgn0039272	
FBgn0051041	FBgn0058298	FBgn0262562	FBgn0004198	FBgn0034706	FBgn0061356	FBgn0026393	FBgn0033917	FBgn0039282	
FBgn0051053	FBgn0062413	FBgn0262563	FBgn0004514	FBgn0034928	FBgn0085421	FBgn0027073	FBgn0034047	FBgn0039294	
FBgn0051072	FBgn0065032	FBgn0262579	FBgn0004577	FBgn0034974	FBgn0085425	FBgn0027521	FBgn0034052	FBgn0039321	
FBgn0051075	FBgn0067102	FBgn0262598	FBgn0004646	FBgn0034985	FBgn0085431	FBgn0027570	FBgn0034053	FBgn0039629	
FBgn0051100	FBgn0083940	FBgn0262599	FBgn0004649	FBgn0035103	FBgn0085440	FBgn0027611	FBgn0034156	FBgn0039655	
FBgn0051125	FBgn0083956	FBgn0262685	FBgn0005564	FBgn0035154	FBgn0085446	FBgn0028979	FBgn0034275	FBgn0039678	

Appendix Table S3. Genes downregulated in L(3)mbt KD

FBgn0000036	FBgn0010265	FBgn0023081	FBgn0027836	FBgn0031169	FBgn0033086	FBgn0034543	FBgn0036035	FBgn0037648	FBgn0039274
FBgn0000064	FBgn0010340	FBgn0023091	FBgn0027873	FBgn0031233	FBgn0033088	FBgn0034617	FBgn0036038	FBgn0037652	FBgn0039303
FBgn0000116	FBgn0010348	FBgn0023143	FBgn0027932	FBgn0031238	FBgn0033089	FBgn0034631	FBgn0036058	FBgn0037686	FBgn0039306
FBgn0000181	FBgn0010355	FBgn0023169	FBgn0028331	FBgn0031256	FBgn0033184	FBgn0034641	FBgn0036063	FBgn0037710	FBgn0039338
FBgn0000247	FBgn0010383	FBgn0023507	FBgn0028341	FBgn0031281	FBgn0033209	FBgn0034650	FBgn0036124	FBgn0037723	FBgn0039349
FBgn0000261	FBgn0010409	FBgn0023510	FBgn0028427	FBgn0031282	FBgn0033225	FBgn0034703	FBgn0036126	FBgn0037741	FBgn0039358
FBgn0000283	FBgn0010504	FBgn0023514	FBgn0028429	FBgn0031286	FBgn0033226	FBgn0034707	FBgn0036136	FBgn0037743	FBgn0039359
FBgn0000319	FBgn0010520	FBgn0023519	FBgn0028473	FBgn0031307	FBgn0033235	FBgn0034728	FBgn0036141	FBgn0037778	FBgn0039406
FBgn0000339	FBgn0010551	FBgn0023521	FBgn0028479	FBgn0031321	FBgn0033244	FBgn0034741	FBgn0036173	FBgn0037794	FBgn0039419
FBgn0000377	FBgn0010611	FBgn0023527	FBgn0028484	FBgn0031360	FBgn0033304	FBgn0034792	FBgn0036187	FBgn0037810	FBgn0039464
FBgn0000395	FBgn0010651	FBgn0024245	FBgn0028500	FBgn0031456	FBgn0033352	FBgn0034849	FBgn0036207	FBgn0037852	FBgn0039555
FBgn0000426	FBgn0010770	FBgn0024277	FBgn0028504	FBgn0031497	FBgn0033354	FBgn0034858	FBgn0036237	FBgn0037882	FBgn0039558
FBgn0000455	FBgn0010772	FBgn0024285	FBgn0028506	FBgn0031500	FBgn0033357	FBgn0034909	FBgn0036254	FBgn0037891	FBgn0039600
FBgn0000559	FBgn0010786	FBgn0024329	FBgn0028509	FBgn0031516	FBgn0033376	FBgn0034915	FBgn0036257	FBgn0037899	FBgn0039627
FBgn0000560	FBgn0010803	FBgn0024330	FBgn0028527	FBgn0031540	FBgn0033379	FBgn0034918	FBgn0036258	FBgn0037901	FBgn0039632
FBgn0000566	FBgn0010926	FBgn0024556	FBgn0028530	FBgn0031597	FBgn0033395	FBgn0034936	FBgn0036301	FBgn0037926	FBgn0039636
FBgn0000579	FBgn0011204	FBgn0024698	FBgn0028538	FBgn0031603	FBgn0033428	FBgn0034938	FBgn0036309	FBgn0037933	FBgn0039654
FBgn0000635	FBgn0011361	FBgn0024806	FBgn0028550	FBgn0031639	FBgn0033454	FBgn0034940	FBgn0036318	FBgn0037955	FBgn0039726
FBgn0001087	FBgn0011591	FBgn0024958	FBgn0028577	FBgn0031643	FBgn0033460	FBgn0034948	FBgn0036324	FBgn0037958	FBgn0039727
FBgn0001092	FBgn0011638	FBgn0024983	FBgn0028647	FBgn0031651	FBgn0033467	FBgn0034964	FBgn0036333	FBgn0037979	FBgn0039740
FBgn0001098	FBgn0011708	FBgn0024992	FBgn0028689	FBgn0031657	FBgn0033476	FBgn0035001	FBgn0036386	FBgn0037998	FBgn0039754
FBgn0001149	FBgn0011737	FBgn0025366	FBgn0028690	FBgn0031659	FBgn0033483	FBgn0035020	FBgn0036451	FBgn0038039	FBgn0039756
FBgn0001168	FBgn0011769	FBgn0025582	FBgn0028697	FBgn0031660	FBgn0033507	FBgn0035039	FBgn0036460	FBgn0038057	FBgn0039765
FBgn0001179	FBgn0011771	FBgn0025628	FBgn0028707	FBgn0031710	FBgn0033528	FBgn0035049	FBgn0036484	FBgn0038110	FBgn0039828
FBgn0001186	FBgn0011817	FBgn0025631	FBgn0028744	FBgn0031805	FBgn0033540	FBgn0035063	FBgn0036500	FBgn0038183	FBgn0039868
FBgn0001202	FBgn0012036	FBgn0025674	FBgn0028919	FBgn0031820	FBgn0033544	FBgn0035083	FBgn0036502	FBgn0038194	FBgn0039870
FBgn0001247	FBgn0013432	FBgn0025742	FBgn0028926	FBgn0031832	FBgn0033547	FBgn0035085	FBgn0036509	FBgn0038195	FBgn0039902
FBgn0001258	FBgn0013435	FBgn0025781	FBgn0028962	FBgn0031851	FBgn0033548	FBgn0035087	FBgn0036514	FBgn0038235	FBgn0039920
FBgn0001308	FBgn0013531	FBgn0025802	FBgn0028982	FBgn0031881	FBgn0033551	FBgn0035106	FBgn0036522	FBgn0038269	FBgn0039929
FBgn0001316	FBgn0013749	FBgn0025809	FBgn0028990	FBgn0031885	FBgn0033556	FBgn0035121	FBgn0036570	FBgn0038271	FBgn0039993
FBgn0001324	FBgn0013750	FBgn0025830	FBgn0029006	FBgn0031893	FBgn0033557	FBgn0035131	FBgn0036571	FBgn0038272	FBgn0040011
FBgn0001341	FBgn0013756	FBgn0025832	FBgn0029114	FBgn0031904	FBgn0033571	FBgn0035137	FBgn0036579	FBgn0038275	FBgn0040022
FBgn0001565	FBgn0013762	FBgn0026015	FBgn0029117	FBgn0031914	FBgn0033592	FBgn0035142	FBgn0036614	FBgn0038294	FBgn0040064
FBgn0001983	FBgn0014007	FBgn0026084	FBgn0029133	FBgn0031951	FBgn0033638	FBgn0035165	FBgn0036623	FBgn0038344	FBgn0040228
FBgn0002031	FBgn0014010	FBgn0026088	FBgn0029664	FBgn0031969	FBgn0033652	FBgn0035173	FBgn0036627	FBgn0038369	FBgn0040236
FBgn0002413	FBgn0014011	FBgn0026089	FBgn0029688	FBgn0031985	FBgn0033661	FBgn0035189	FBgn0036668	FBgn0038376	FBgn0040271
FBgn0002441	FBgn0014075	FBgn0026147	FBgn0029689	FBgn0032004	FBgn0033672	FBgn0035203	FBgn0036684	FBgn0038389	FBgn0040294
FBgn0002566	FBgn0014366	FBgn0026149	FBgn0029706	FBgn0032016	FBgn0033688	FBgn0035205	FBgn0036686	FBgn0038426	FBgn0040389
FBgn0002579	FBgn0014427	FBgn0026179	FBgn0029718	FBgn0032026	FBgn0033698	FBgn0035227	FBgn0036702	FBgn0038432	FBgn0040467
FBgn0002593	FBgn0015037	FBgn0026181	FBgn0029755	FBgn0032033	FBgn0033713	FBgn0035232	FBgn0036754	FBgn0038454	FBgn0041094
FBgn0002643	FBgn0015221	FBgn0026196	FBgn0029785	FBgn0032034	FBgn0033738	FBgn0035236	FBgn0036805	FBgn0038455	FBgn0041147
FBgn0002780	FBgn0015222	FBgn0026250	FBgn0029804	FBgn0032077	FBgn0033741	FBgn0035295	FBgn0036811	FBgn0038473	FBgn0041588
FBgn0002887	FBgn0015229	FBgn0026252	FBgn0029870	FBgn0032138	FBgn0033754	FBgn0035334	FBgn0036814	FBgn0038488	FBgn0041604
FBgn0003008	FBgn0015245	FBgn0026262	FBgn0029885	FBgn0032168	FBgn0033766	FBgn0035336	FBgn0036828	FBgn0038491	FBgn0041627
FBgn0003062	FBgn0015268	FBgn0026317	FBgn0029895	FBgn0032208	FBgn0033769	FBgn0035393	FBgn0036837	FBgn0038578	FBgn0041629
FBgn0003189	FBgn0015269	FBgn0026373	FBgn0029959	FBgn0032217	FBgn0033808	FBgn0035402	FBgn0036838	FBgn0038585	FBgn0041789
FBgn0003204	FBgn0015279	FBgn0026378	FBgn0029996	FBgn0032222	FBgn0033816	FBgn0035420	FBgn0036853	FBgn0038593	FBgn0042083
FBgn0003231	FBgn0015288	FBgn0026380	FBgn0030018	FBgn0032229	FBgn0033842	FBgn0035422	FBgn0036886	FBgn0038651	FBgn0042134
FBgn0003257	FBgn0015338	FBgn0026417	FBgn0030052	FBgn0032230	FBgn0033859	FBgn0035438	FBgn0036892	FBgn0038666	FBgn0042213
FBgn0003274	FBgn0015477	FBgn0026582	FBgn0030060	FBgn0032236	FBgn0033900	FBgn0035440	FBgn0036900	FBgn0038672	FBgn0042712
FBgn0003278	FBgn0015521	FBgn0026630	FBgn0030061	FBgn0032242	FBgn0033907	FBgn0035462	FBgn0036916	FBgn0038742	FBgn0044047
FBgn0003360	FBgn0015527	FBgn0026634	FBgn0030067	FBgn0032243	FBgn0033918	FBgn0035499	FBgn0036920	FBgn0038745	FBgn0044323
FBgn0003366	FBgn0015754	FBgn0026666	FBgn0030092	FBgn0032248	FBgn0033924	FBgn0035524	FBgn0036922	FBgn0038746	FBgn0044452
FBgn0003401	FBgn0015765	FBgn0026751	FBgn0030136	FBgn0032261	FBgn0033984	FBgn0035541	FBgn0037008	FBgn0038834	FBgn0046685
FBgn0003559	FBgn0015791	FBgn0026787	FBgn0030177	FBgn0032296	FBgn0033989	FBgn0035588	FBgn0037011	FBgn0038853	FBgn0050000
FBgn0003656	FBgn0015794	FBgn0026869	FBgn0030183	FBgn0032321	FBgn0033995	FBgn0035589	FBgn0037012	FBgn0038854	FBgn0050022
FBgn0003716	FBgn0015795	FBgn0027055	FBgn0030291	FBgn0032395	FBgn0034001	FBgn0035591	FBgn0037019	FBgn0038857	FBgn0050035
FBgn0003891	FBgn0015797	FBgn0027057	FBgn0030321	FBgn0032397	FBgn0034027	FBgn0035600	FBgn0037071	FBgn0038858	FBgn0050159
FBgn0003941	FBgn0015829	FBgn0027066	FBgn0030406	FBgn0032398	FBgn0034029	FBgn0035603	FBgn0037073	FBgn0038869	FBgn0050169
FBgn0003984	FBgn0015925	FBgn0027079	FBgn0030431	FBgn0032408	FBgn0034054	FBgn0035630	FBgn0037105	FBgn0038870	FBgn0050342
FBgn0004087	FBgn0016081	FBgn0027080	FBgn0030479	FBgn0032445	FBgn0034073	FBgn0035656	FBgn0037137	FBgn0038889	FBgn0050349
FBgn0004367	FBgn0016641	FBgn0027081	FBgn0030482	FBgn0032479	FBgn0034087	FBgn0035688	FBgn0037151	FBgn0038890	FBgn0050394
FBgn0004387	FBgn0016685	FBgn0027082	FBgn0030519	FBgn0032487	FBgn0034109	FBgn0035703	FBgn0037206	FBgn0038893	FBgn0050404
FBgn0004390	FBgn0016701	FBgn0027086	FBgn0030569	FBgn0032597	FBgn0034110	FBgn0035714	FBgn0037231	FBgn0038953	FBgn0050410
FBgn0004404	FBgn0016726	FBgn0027087	FBgn0030588	FBgn0032620	FBgn0034113	FBgn0035722	FBgn0037232	FBgn0038964	FBgn0050423
FBgn0004465	FBgn0016797	FBgn0027088	FBgn0030603	FBgn0032634	FBgn0034118	FBgn0035725	FBgn0037327	FBgn0038968	FBgn0050476
FBgn0004556	FBgn0016917	FBgn0027090	FBgn0030634	FBgn0032656	FBgn0034158	FBgn0035727	FBgn0037328	FBgn0038989	FBgn0050491
FBgn0004638	FBgn0016930	FBgn0027091	FBgn0030641	FBgn0032670	FBgn0034180	FBgn0035754	FBgn0037345	FBgn0039004	FBgn0050493
FBgn0004876	FBgn0017578	FBgn0027093	FBgn0030654	FBgn0032690	FBgn0034191	FBgn0035760	FBgn0037382	FBgn0039043	FBgn0051111
FBgn0004893	FBgn0020236	FBgn0027094	FBgn0030658	FBgn0032699	FBgn0034214	FBgn0035763	FBgn0037391	FBgn0039055	FBgn0051133
FBgn0004901	FBgn0020278	FBgn0027101	FBgn0030671	FBgn0032724	FBgn0034223	FBgn0035802	FBgn0037487	FBgn0039056	FBgn0051223
FBgn0004907	FBgn0020445	FBgn0027296	FBgn0030676	FBgn0032728	FBgn0034243	FBgn0035805	FBgn0037489	FBgn0039109	FBgn0051224
FBgn0004908	FBgn0020497	FBgn0027330	FBgn0030687	FBgn0032731	FBgn0034246	FBgn0035833	FBgn0037529	FBgn0039120	FBgn0051278
FBgn0004926	FBgn0020622	FBgn0027493	FBgn0030692	FBgn0032781	FBgn0034258	FBgn0035851	FBgn0037536	FBgn0039136	FBgn0051357
FBgn0005278	FBgn0020764	FBgn0027503	FBgn0030720	FBgn0032799	FBgn0034259	FBgn0035868	FBgn0037537	FBgn0039139	FBgn0051361
FBgn0005533	FBgn0020767	FBgn0027508	FBgn0030739	FBgn0032811	FBgn0034271	FBgn0035877	FBgn0037541	FBgn0039145	FBgn0051368
FBgn0005612	FBgn0021764	FBgn0027525	FBgn0030752	FBgn0032849	FBgn0034315	FBgn0035879	FBgn0037543	FBgn0039155	FBgn0051522
FBgn0005616	FBgn0021768	FBgn0027526	FBgn0030805	FBgn0032886	FBgn0034351	FBgn0035901	FBgn0037549	FBgn0039156	FBgn0051678
FBgn0005624	FBgn0021796	FBgn0027554	FBgn0030809	FBgn0032891	FBgn0034361	FBgn0035923	FBgn0037551	FBgn0039157	FBgn0051755
FBgn0005632	FBgn0021874	FBgn0027568	FBgn0030850	FBgn0032919	FBgn0034392	FBgn0035953	FBgn0037561	FBgn0039175	FBgn0051759
FBgn0005640	FBgn0021979	FBgn0027569	FBgn0030894	FBgn0032923	FBgn0034400	FBgn0035969	FBgn0037565	FBgn0039205	FBgn0051852
FBgn0005672	FBgn0021995	FBgn0027583	FBgn0030969	FBgn0032925	FBgn0034401	FBgn0035986	FBgn0037580	FBgn0039207	FBgn0052039
FBgn0005674	FBgn0022029	FBgn0027594	FBgn0031040	FBgn0032956	FBgn0034418	FBgn0035996	FBgn0037603	FBgn0039223	FBgn0052069
FBgn0005777	FBgn0022069	FBgn0027615	FBgn0031051	FBgn0032974	FBgn0034447	FBgn0036000	FBgn0037608	FBgn0039252	FBgn0052075
FBgn0010038	FBgn0022238	FBgn0027655	FBgn0031114	FBgn0032988	FBgn0034493	FBgn0036007	FBgn0037609	FBgn0039254	FBgn0052104
FBgn0010220	FBgn0022942	FBgn0027780	FBgn0031118	FBgn0033038	FBgn0034494	FBgn0036028	FBgn0037637	FBgn0039259	FBgn0052113
FBgn0010235	FBgn0023000	FBgn0027794	FBgn0031144	FBgn0033052	FBgn0034542	FBgn0036030	FBgn0037638	FBgn0039271	FBgn0052296

FBgn0052344
FBgn0052423
FBgn0052521
FBgn0052533
FBgn0052594
FBgn0052600
FBgn0052649
FBgn0052672
FBgn0053052
FBgn0053100
FBgn0053129
FBgn0053158
FBgn0053180
FBgn0061469
FBgn0061476
FBgn0063492
FBgn0063493
FBgn0064225
FBgn0064766
FBgn0066084
FBgn0085192
FBgn0085294
FBgn0085339
FBgn0085376
FBgn0085437
FBgn0085451
FBgn0086130
FBgn0086251
FBgn0086347
FBgn0086371
FBgn0086443
FBgn0086446
FBgn0086451
FBgn0086472
FBgn0086679
FBgn0086687
FBgn0086706
FBgn0086710
FBgn0086711
FBgn0086895
FBgn0086912
FBgn0243511
FBgn0250785
FBgn0250814
FBgn0250868
FBgn0259152
FBgn0259170
FBgn0259481
FBgn0259711
FBgn0259742
FBgn0259821
FBgn0259832
FBgn0259937
FBgn0259984
FBgn0260012
FBgn0260442
FBgn0260456
FBgn0260632
FBgn0260648
FBgn0260745
FBgn0260755
FBgn0260855
FBgn0260857
FBgn0260965
FBgn0260990
FBgn0261020
FBgn0261049
FBgn0261068
FBgn0261258
FBgn0261274
FBgn0261276
FBgn0261285
FBgn0261286
FBgn0261380
FBgn0261444
FBgn0261530
FBgn0261535
FBgn0261550
FBgn0261554
FBgn0261560
FBgn0261565
FBgn0261574
FBgn0261596
FBgn0261599
FBgn0261602
FBgn0261800
FBgn0261931
FBgn0261933
FBgn0261934
FBgn0261938
FBgn0261984
FBgn0262115
FBgn0262117
FBgn0262125
FBgn0262126
FBgn0262160
FBgn0262166
FBgn0262527
FBgn0262582
FBgn0262601
FBgn0262647
FBgn0262733
FBgn0262954
FBgn0262955
FBgn0263110
FBgn0263120
FBgn0263200
FBgn0263316
FBgn0263346
FBgn0263351
FBgn0263510
FBgn0263594
FBgn0263599
FBgn0263605
FBgn0263606
FBgn0263740
FBgn0263929
FBgn0263995
FBgn0264006
FBgn0264294
FBgn0264325
FBgn0264560
FBgn0264751
FBgn0264978
FBgn0265003
FBgn0265187
FBgn0265413
FBgn0265574
FBgn0265998
FBgn0266019
FBgn0266053
FBgn0266268
FBgn0266284
FBgn0266409
FBgn0266411
FBgn0266581
FBgn0266668
FBgn0266673
FBgn0266674
FBgn0266696
FBgn0266717
FBgn0266722
FBgn0266723
FBgn0267350
FBgn0267385
FBgn0267976
FBgn0270926
FBgn0283427
FBgn0283468
FBgn0283472
FBgn0283473
FBgn0283510
FBgn0283659
FBgn0283680
FBgn0283681
FBgn0283724
FBgn0283741
FBgn0284245
FBgn0284246
FBgn0284248
FBgn0284252
FBgn0285911
FBgn0285954
FBgn0285955
FBgn0285962
FBgn0286075
FBgn0286786
FBgn0286813
FBgn0286898
FBgn0287225
FBgn0287478
FBgn0287631
FBgn0287774
FBgn0288310
FBgn0000326
FBgn0002069
FBgn0003507
FBgn0017581
FBgn0024183
FBgn0026160
FBgn0028496
FBgn0029879
FBgn0029896
FBgn0030156
FBgn0030870
FBgn0031011
FBgn0031530
FBgn0031568
FBgn0031673
FBgn0032723
FBgn0034389
FBgn0034501
FBgn0034734
FBgn0034903
FBgn0035023
FBgn0035470
FBgn0037613
FBgn0037672
FBgn0037989
FBgn0038720
FBgn0039209
FBgn0039257
FBgn0039266
FBgn0039829
FBgn0039858
FBgn0040299
FBgn0040496
FBgn0043841
FBgn0050428
FBgn0051525
FBgn0052311
FBgn0062978
FBgn0082585
FBgn0086906
FBgn0243512
FBgn0250753
FBgn0259245
FBgn0259935
FBgn0259938
FBgn0260660
FBgn0263219
FBgn0263256
FBgn0264907
FBgn0266099
FBgn0267796
FBgn0285963
FBgn0000037
FBgn0002576
FBgn0002645
FBgn0002783
FBgn0002930
FBgn0003285
FBgn0003751
FBgn0005626
FBgn0019990
FBgn0027505
FBgn0028888
FBgn0029697
FBgn0030438
FBgn0030693
FBgn0030882
FBgn0030960
FBgn0030983
FBgn0031227
FBgn0031728
FBgn0031857
FBgn0031888
FBgn0032139
FBgn0032258
FBgn0032259
FBgn0032645
FBgn0032969
FBgn0033190
FBgn0033696
FBgn0033697
FBgn0033725
FBgn0033888
FBgn0035281
FBgn0035383
FBgn0036018
FBgn0036512
FBgn0036597
FBgn0036665
FBgn0036970
FBgn0038660
FBgn0038680
FBgn0039494
FBgn0039704
FBgn0040079
FBgn0040251
FBgn0040256
FBgn0040259
FBgn0040609
FBgn0042173
FBgn0045843
FBgn0051547
FBgn0051642
FBgn0051876
FBgn0052641
FBgn0052655
FBgn0063491
FBgn0085249
FBgn0085407
FBgn0263116
FBgn0264699
FBgn0266261
FBgn0266431
FBgn0286782

Appendix Table S3. Genes upregulated in Lint-O KD

FBgn0000046	FBgn0014930	FBgn0029911	FBgn0031844	FBgn0033453	FBgn0035959	FBgn0038038	FBgn0041205	FBgn0058298	FBgn0263258
FBgn0000053	FBgn0014931	FBgn0029924	FBgn0031874	FBgn0033468	FBgn0035968	FBgn0038042	FBgn0041706	FBgn0062413	FBgn0263355
FBgn0000084	FBgn0015520	FBgn0029970	FBgn0031886	FBgn0033555	FBgn0036004	FBgn0038045	FBgn0042178	FBgn0065032	FBgn0263395
FBgn0000108	FBgn0015558	FBgn0029974	FBgn0031896	FBgn0033584	FBgn0036106	FBgn0038053	FBgn0042185	FBgn0083956	FBgn0263601
FBgn0000158	FBgn0015801	FBgn0029975	FBgn0031905	FBgn0033627	FBgn0036121	FBgn0038105	FBgn0043792	FBgn0083962	FBgn0263974
FBgn0000246	FBgn0015924	FBgn0029977	FBgn0031910	FBgn0033683	FBgn0036133	FBgn0038191	FBgn0043884	FBgn0085223	FBgn0264089
FBgn0000299	FBgn0015926	FBgn0030011	FBgn0031952	FBgn0033686	FBgn0036144	FBgn0038250	FBgn0045064	FBgn0085317	FBgn0264305
FBgn0000317	FBgn0016036	FBgn0030099	FBgn0031968	FBgn0033715	FBgn0036147	FBgn0038252	FBgn0046258	FBgn0085425	FBgn0264324
FBgn0000346	FBgn0016075	FBgn0030114	FBgn0031975	FBgn0033748	FBgn0036206	FBgn0038273	FBgn0046763	FBgn0085428	FBgn0264326
FBgn0000414	FBgn0016122	FBgn0030189	FBgn0031986	FBgn0033749	FBgn0036271	FBgn0038277	FBgn0046878	FBgn0085438	FBgn0264832
FBgn0000422	FBgn0017577	FBgn0030207	FBgn0031998	FBgn0033767	FBgn0036272	FBgn0038299	FBgn0050015	FBgn0085440	FBgn0264897
FBgn0000533	FBgn0019985	FBgn0030245	FBgn0032006	FBgn0033785	FBgn0036279	FBgn0038311	FBgn0050016	FBgn0085446	FBgn0264953
FBgn0000557	FBgn0020257	FBgn0030266	FBgn0032013	FBgn0033799	FBgn0036330	FBgn0038312	FBgn0050069	FBgn0086254	FBgn0265182
FBgn0000615	FBgn0020312	FBgn0030274	FBgn0032029	FBgn0033807	FBgn0036368	FBgn0038318	FBgn0050085	FBgn0086690	FBgn0265262
FBgn0000644	FBgn0020372	FBgn0030290	FBgn0032078	FBgn0033814	FBgn0036374	FBgn0038349	FBgn0050091	FBgn0086691	FBgn0265523
FBgn0000996	FBgn0020385	FBgn0030347	FBgn0032079	FBgn0033876	FBgn0036381	FBgn0038419	FBgn0050195	FBgn0086898	FBgn0265575
FBgn0001120	FBgn0020389	FBgn0030357	FBgn0032117	FBgn0033913	FBgn0036405	FBgn0038431	FBgn0050296	FBgn0086909	FBgn0265725
FBgn0001222	FBgn0020513	FBgn0030362	FBgn0032120	FBgn0033921	FBgn0036503	FBgn0038463	FBgn0050344	FBgn0087002	FBgn0266083
FBgn0001224	FBgn0021776	FBgn0030396	FBgn0032123	FBgn0033960	FBgn0036612	FBgn0038465	FBgn0050380	FBgn0087035	FBgn0266101
FBgn0001225	FBgn0022160	FBgn0030412	FBgn0032161	FBgn0033987	FBgn0036622	FBgn0038490	FBgn0050383	FBgn0250788	FBgn0266418
FBgn0001226	FBgn0022702	FBgn0030432	FBgn0032167	FBgn0034013	FBgn0036640	FBgn0038540	FBgn0050403	FBgn0250789	FBgn0266421
FBgn0001229	FBgn0022768	FBgn0030457	FBgn0032192	FBgn0034067	FBgn0036652	FBgn0038550	FBgn0050414	FBgn0250848	FBgn0266521
FBgn0001230	FBgn0022774	FBgn0030478	FBgn0032211	FBgn0034075	FBgn0036663	FBgn0038575	FBgn0050424	FBgn0259140	FBgn0266557
FBgn0001248	FBgn0022981	FBgn0030484	FBgn0032213	FBgn0034095	FBgn0036689	FBgn0038581	FBgn0050438	FBgn0259173	FBgn0266717
FBgn0001311	FBgn0023023	FBgn0030508	FBgn0032219	FBgn0034106	FBgn0036698	FBgn0038612	FBgn0050440	FBgn0259185	FBgn0266719
FBgn0002431	FBgn0023094	FBgn0030510	FBgn0032221	FBgn0034117	FBgn0036764	FBgn0038652	FBgn0050441	FBgn0259195	FBgn0267002
FBgn0002441	FBgn0023172	FBgn0030521	FBgn0032228	FBgn0034162	FBgn0036773	FBgn0038680	FBgn0050466	FBgn0259224	FBgn0267339
FBgn0002526	FBgn0023458	FBgn0030545	FBgn0032233	FBgn0034179	FBgn0036801	FBgn0038691	FBgn0050484	FBgn0259226	FBgn0267348
FBgn0002567	FBgn0023537	FBgn0030551	FBgn0032249	FBgn0034187	FBgn0036810	FBgn0038814	FBgn0051002	FBgn0259238	FBgn0267792
FBgn0002773	FBgn0023540	FBgn0030597	FBgn0032294	FBgn0034199	FBgn0036813	FBgn0038815	FBgn0051021	FBgn0259242	FBgn0267828
FBgn0002878	FBgn0024150	FBgn0030600	FBgn0032364	FBgn0034221	FBgn0036824	FBgn0038830	FBgn0051053	FBgn0259683	FBgn0267967
FBgn0002937	FBgn0024177	FBgn0030629	FBgn0032402	FBgn0034255	FBgn0036849	FBgn0038912	FBgn0051072	FBgn0259685	FBgn0283427
FBgn0002962	FBgn0024320	FBgn0030647	FBgn0032428	FBgn0034300	FBgn0036896	FBgn0038916	FBgn0051075	FBgn0259734	FBgn0283440
FBgn0003048	FBgn0024366	FBgn0030660	FBgn0032447	FBgn0034371	FBgn0036927	FBgn0038966	FBgn0051125	FBgn0259735	FBgn0283442
FBgn0003114	FBgn0024836	FBgn0030674	FBgn0032452	FBgn0034420	FBgn0036942	FBgn0038978	FBgn0051140	FBgn0259740	FBgn0283509
FBgn0003187	FBgn0024841	FBgn0030683	FBgn0032456	FBgn0034452	FBgn0036956	FBgn0039002	FBgn0051141	FBgn0259791	FBgn0283545
FBgn0003218	FBgn0024913	FBgn0030706	FBgn0032475	FBgn0034500	FBgn0036969	FBgn0039044	FBgn0051217	FBgn0259992	FBgn0284408
FBgn0003250	FBgn0024984	FBgn0030716	FBgn0032519	FBgn0034592	FBgn0036980	FBgn0039075	FBgn0051248	FBgn0260388	FBgn0284442
FBgn0003292	FBgn0025382	FBgn0030723	FBgn0032520	FBgn0034606	FBgn0036984	FBgn0039114	FBgn0051321	FBgn0260442	FBgn0286222
FBgn0003301	FBgn0025641	FBgn0030742	FBgn0032598	FBgn0034628	FBgn0037023	FBgn0039129	FBgn0051344	FBgn0260766	FBgn0286785
FBgn0003353	FBgn0025686	FBgn0030793	FBgn0032668	FBgn0034694	FBgn0037046	FBgn0039180	FBgn0051373	FBgn0260779	FBgn0287186
FBgn0003390	FBgn0025837	FBgn0030816	FBgn0032680	FBgn0034718	FBgn0037050	FBgn0039251	FBgn0051431	FBgn0260794	FBgn0287695
FBgn0003391	FBgn0025885	FBgn0030841	FBgn0032713	FBgn0034725	FBgn0037063	FBgn0039265	FBgn0051516	FBgn0260795	FBgn0287725
FBgn0003462	FBgn0026059	FBgn0030847	FBgn0032727	FBgn0034733	FBgn0037086	FBgn0039272	FBgn0051619	FBgn0260932	FBgn0287873
FBgn0003527	FBgn0026148	FBgn0030876	FBgn0032752	FBgn0034753	FBgn0037092	FBgn0039277	FBgn0051636	FBgn0260933	FBgn0000075
FBgn0003655	FBgn0026176	FBgn0030884	FBgn0032779	FBgn0034797	FBgn0037107	FBgn0039356	FBgn0051658	FBgn0260970	FBgn0000927
FBgn0003733	FBgn0026239	FBgn0030937	FBgn0032788	FBgn0034804	FBgn0037142	FBgn0039507	FBgn0051674	FBgn0261041	FBgn0002778
FBgn0003950	FBgn0026263	FBgn0030955	FBgn0032798	FBgn0034816	FBgn0037183	FBgn0039564	FBgn0051718	FBgn0261086	FBgn0003028
FBgn0004167	FBgn0026374	FBgn0030968	FBgn0032800	FBgn0034850	FBgn0037234	FBgn0039622	FBgn0051720	FBgn0261243	FBgn0003046
FBgn0004360	FBgn0026874	FBgn0030994	FBgn0032813	FBgn0034880	FBgn0037239	FBgn0039640	FBgn0051789	FBgn0261260	FBgn0003149
FBgn0004400	FBgn0027280	FBgn0031053	FBgn0032815	FBgn0034935	FBgn0037254	FBgn0039644	FBgn0051989	FBgn0261261	FBgn0003162
FBgn0004432	FBgn0027491	FBgn0031091	FBgn0032817	FBgn0034950	FBgn0037296	FBgn0039666	FBgn0051999	FBgn0261262	FBgn0003174
FBgn0004449	FBgn0027500	FBgn0031097	FBgn0032820	FBgn0034988	FBgn0037315	FBgn0039688	FBgn0052068	FBgn0261363	FBgn0003396
FBgn0004581	FBgn0027552	FBgn0031104	FBgn0032821	FBgn0035035	FBgn0037338	FBgn0039702	FBgn0052088	FBgn0261397	FBgn0003447
FBgn0004611	FBgn0027560	FBgn0031116	FBgn0032864	FBgn0035073	FBgn0037354	FBgn0039714	FBgn0052177	FBgn0261445	FBgn0003719
FBgn0004647	FBgn0027570	FBgn0031117	FBgn0032881	FBgn0035113	FBgn0037364	FBgn0039752	FBgn0052195	FBgn0261547	FBgn0004047
FBgn0004650	FBgn0027580	FBgn0031161	FBgn0032884	FBgn0035151	FBgn0037436	FBgn0039755	FBgn0052280	FBgn0261574	FBgn0010222
FBgn0004882	FBgn0027600	FBgn0031170	FBgn0032889	FBgn0035167	FBgn0037465	FBgn0039774	FBgn0052313	FBgn0261617	FBgn0010263
FBgn0004885	FBgn0028379	FBgn0031171	FBgn0032907	FBgn0035169	FBgn0037513	FBgn0039827	FBgn0052365	FBgn0261671	FBgn0013767
FBgn0005558	FBgn0028408	FBgn0031191	FBgn0032955	FBgn0035265	FBgn0037562	FBgn0039863	FBgn0052368	FBgn0261794	FBgn0014018
FBgn0005631	FBgn0028525	FBgn0031228	FBgn0032957	FBgn0035287	FBgn0037579	FBgn0039883	FBgn0052373	FBgn0261797	FBgn0015569
FBgn0005633	FBgn0028540	FBgn0031232	FBgn0032986	FBgn0035308	FBgn0037581	FBgn0039896	FBgn0052436	FBgn0261802	FBgn0016977
FBgn0005660	FBgn0028546	FBgn0031239	FBgn0033020	FBgn0035321	FBgn0037623	FBgn0039900	FBgn0052451	FBgn0261836	FBgn0024189
FBgn0010014	FBgn0028563	FBgn0031245	FBgn0033031	FBgn0035356	FBgn0037654	FBgn0039908	FBgn0052457	FBgn0261955	FBgn0025616
FBgn0010051	FBgn0028579	FBgn0031254	FBgn0033033	FBgn0035407	FBgn0037664	FBgn0039916	FBgn0052476	FBgn0261985	FBgn0025702
FBgn0010097	FBgn0028670	FBgn0031263	FBgn0033048	FBgn0035410	FBgn0037669	FBgn0040009	FBgn0052483	FBgn0261987	FBgn0025878
FBgn0010226	FBgn0028671	FBgn0031264	FBgn0033101	FBgn0035445	FBgn0037690	FBgn0040102	FBgn0052485	FBgn0262101	FBgn0026061
FBgn0010246	FBgn0028916	FBgn0031313	FBgn0033159	FBgn0035452	FBgn0037721	FBgn0040212	FBgn0052506	FBgn0262123	FBgn0026439
FBgn0010317	FBgn0028983	FBgn0031315	FBgn0033174	FBgn0035477	FBgn0037736	FBgn0040308	FBgn0052557	FBgn0262364	FBgn0026721
FBgn0010406	FBgn0028984	FBgn0031317	FBgn0033177	FBgn0035515	FBgn0037747	FBgn0040321	FBgn0052579	FBgn0262562	FBgn0027111
FBgn0010407	FBgn0028996	FBgn0031384	FBgn0033188	FBgn0035521	FBgn0037749	FBgn0040322	FBgn0052625	FBgn0262563	FBgn0027596
FBgn0010482	FBgn0029105	FBgn0031413	FBgn0033199	FBgn0035523	FBgn0037754	FBgn0040323	FBgn0052626	FBgn0262599	FBgn0027611
FBgn0011206	FBgn0029131	FBgn0031451	FBgn0033204	FBgn0035575	FBgn0037759	FBgn0040333	FBgn0052706	FBgn0262614	FBgn0028491
FBgn0011230	FBgn0029147	FBgn0031489	FBgn0033214	FBgn0035578	FBgn0037841	FBgn0040348	FBgn0053017	FBgn0262686	FBgn0028899
FBgn0011286	FBgn0029507	FBgn0031490	FBgn0033215	FBgn0035587	FBgn0037844	FBgn0040382	FBgn0053087	FBgn0262719	FBgn0028979
FBgn0011293	FBgn0029514	FBgn0031520	FBgn0033261	FBgn0035623	FBgn0037846	FBgn0040384	FBgn0053108	FBgn0262738	FBgn0030001
FBgn0011606	FBgn0029573	FBgn0031609	FBgn0033274	FBgn0035677	FBgn0037849	FBgn0040465	FBgn0053156	FBgn0262782	FBgn0030085
FBgn0011704	FBgn0029685	FBgn0031630	FBgn0033312	FBgn0035724	FBgn0037853	FBgn0040491	FBgn0053289	FBgn0262820	FBgn0030367
FBgn0011705	FBgn0029694	FBgn0031636	FBgn0033373	FBgn0035751	FBgn0037885	FBgn0040507	FBgn0053469	FBgn0262985	FBgn0030890
FBgn0012037	FBgn0029708	FBgn0031637	FBgn0033374	FBgn0035761	FBgn0037896	FBgn0040524	FBgn0053494	FBgn0263022	FBgn0030993
FBgn0013733	FBgn0029712	FBgn0031646	FBgn0033381	FBgn0035767	FBgn0037897	FBgn0040551	FBgn0053558	FBgn0263023	FBgn0030998
FBgn0013763	FBgn0029723	FBgn0031677	FBgn0033388	FBgn0035770	FBgn0037921	FBgn0040670	FBgn0053653	FBgn0263029	FBgn0031195
FBgn0013773	FBgn0029754	FBgn0031717	FBgn0033431	FBgn0035847	FBgn0037944	FBgn0040752	FBgn0053679	FBgn0263076	FBgn0031240
FBgn0013810	FBgn0029765	FBgn0031740	FBgn0033438	FBgn0035896	FBgn0037950	FBgn0040759	FBgn0053792	FBgn0263143	FBgn0031414
FBgn0013981	FBgn0029830	FBgn0031762	FBgn0033446	FBgn0035907	FBgn0037972	FBgn0040823	FBgn0053926	FBgn0263232	FBgn0031634
FBgn0014031	FBgn0029834	FBgn0031814	FBgn0033448	FBgn0035943	FBgn0037999	FBgn0041102	FBgn0054012	FBgn0263234	FBgn0031961
FBgn0014342	FBgn0029892	FBgn0031829	FBgn0033451	FBgn0035957	FBgn0038037	FBgn0041180	FBgn0058006	FBgn0263240	FBgn0032010

FBgn0032040
FBgn0032055
FBgn0032218
FBgn0032345
FBgn0032726
FBgn0032749
FBgn0033096
FBgn0033110
FBgn0033203
FBgn0033222
FBgn0033246
FBgn0033657
FBgn0033904
FBgn0033963
FBgn0034052
FBgn0034094
FBgn0034247
FBgn0034489
FBgn0034497
FBgn0034706
FBgn0034943
FBgn0034966
FBgn0034974
FBgn0034985
FBgn0035034
FBgn0035094
FBgn0035103
FBgn0035234
FBgn0035264
FBgn0035505
FBgn0035612
FBgn0035903
FBgn0036195
FBgn0036282
FBgn0036479
FBgn0036568
FBgn0036727
FBgn0036806
FBgn0036875
FBgn0036978
FBgn0036993
FBgn0037276
FBgn0037618
FBgn0037714
FBgn0037717
FBgn0037750
FBgn0037751
FBgn0037916
FBgn0038076
FBgn0038079
FBgn0038290
FBgn0038330
FBgn0038602
FBgn0038740
FBgn0039201
FBgn0039415
FBgn0039500
FBgn0039594
FBgn0039651
FBgn0039789
FBgn0039886
FBgn0039897
FBgn0039932
FBgn0040091
FBgn0040096
FBgn0040372
FBgn0041234
FBgn0041723
FBgn0042133
FBgn0050502
FBgn0051161
FBgn0051202
FBgn0051231
FBgn0051279
FBgn0051706
FBgn0052036
FBgn0053099
FBgn0053257
FBgn0053468
FBgn0053519
FBgn0061356
FBgn0083960
FBgn0085384
FBgn0085421
FBgn0085431
FBgn0085434
FBgn0086906
FBgn0250816
FBgn0250869
FBgn0259108
FBgn0259216
FBgn0260874
FBgn0261016
FBgn0261859
FBgn0261996
FBgn0262057
FBgn0262477
FBgn0262509
FBgn0262531
FBgn0262867
FBgn0262871
FBgn0263111
FBgn0263219
FBgn0264815
FBgn0264895
FBgn0265045
FBgn0265052
FBgn0265274
FBgn0266129
FBgn0266709
FBgn0284435
FBgn0000146
FBgn0000153
FBgn0001259
FBgn0001296
FBgn0001991
FBgn0003137
FBgn0003308
FBgn0003475
FBgn0003486
FBgn0003886
FBgn0003890
FBgn0004118
FBgn0004512
FBgn0005427
FBgn0005666
FBgn0010241
FBgn0010258
FBgn0010473
FBgn0011296
FBgn0011722
FBgn0011828
FBgn0013348
FBgn0013813
FBgn0014000
FBgn0014395
FBgn0014396
FBgn0014417
FBgn0015032
FBgn0015519
FBgn0020379
FBgn0022709
FBgn0023416
FBgn0023495
FBgn0024732
FBgn0026144
FBgn0026562
FBgn0026593
FBgn0027070
FBgn0027073
FBgn0027521
FBgn0027550
FBgn0028988
FBgn0029091
FBgn0029710
FBgn0029856
FBgn0029986
FBgn0029987
FBgn0030028
FBgn0030244
FBgn0030310
FBgn0030590
FBgn0030653
FBgn0030691
FBgn0030748
FBgn0030859
FBgn0031004
FBgn0031018
FBgn0031157
FBgn0031184
FBgn0031255
FBgn0031343
FBgn0031515
FBgn0031548
FBgn0031715
FBgn0031716
FBgn0031737
FBgn0031869
FBgn0031887
FBgn0031920
FBgn0031974
FBgn0032003
FBgn0032082
FBgn0032178
FBgn0032225
FBgn0032252
FBgn0032253
FBgn0032262
FBgn0032374
FBgn0032375
FBgn0032422
FBgn0032601
FBgn0032683
FBgn0032774
FBgn0032783
FBgn0032803
FBgn0032900
FBgn0032972
FBgn0032973
FBgn0033051
FBgn0033079
FBgn0033113
FBgn0033115
FBgn0033221
FBgn0033234
FBgn0033268
FBgn0033387
FBgn0033658
FBgn0033659
FBgn0033753
FBgn0033763
FBgn0033875
FBgn0033917
FBgn0034047
FBgn0034279
FBgn0034413
FBgn0034530
FBgn0034538
FBgn0034602
FBgn0034662
FBgn0034789
FBgn0034928
FBgn0034956
FBgn0034957
FBgn0034972
FBgn0035056
FBgn0035104
FBgn0035286
FBgn0035317
FBgn0035412
FBgn0035434
FBgn0035571
FBgn0035880
FBgn0035904
FBgn0035985
FBgn0036040
FBgn0036131
FBgn0036168
FBgn0036208
FBgn0036454
FBgn0036547
FBgn0036575
FBgn0036687
FBgn0036738
FBgn0036752
FBgn0036765
FBgn0036890
FBgn0036891
FBgn0036910
FBgn0037028
FBgn0037060
FBgn0037222
FBgn0037225
FBgn0037304
FBgn0037347
FBgn0037375
FBgn0037563
FBgn0037659
FBgn0037807
FBgn0037835
FBgn0037915
FBgn0037934
FBgn0037973
FBgn0037974
FBgn0037975
FBgn0038017
FBgn0038067
FBgn0038260
FBgn0038261
FBgn0038315
FBgn0038407
FBgn0038435
FBgn0038492
FBgn0038530
FBgn0038761
FBgn0038957
FBgn0039006
FBgn0039124
FBgn0039282
FBgn0039294
FBgn0039321
FBgn0039504
FBgn0039629
FBgn0039655
FBgn0039678
FBgn0039818
FBgn0039911
FBgn0039915
FBgn0039927
FBgn0040370
FBgn0041233
FBgn0041710
FBgn0042696
FBgn0043043
FBgn0043575
FBgn0043806
FBgn0045980
FBgn0050345
FBgn0050485
FBgn0051017
FBgn0051370
FBgn0051436
FBgn0051463
FBgn0051464
FBgn0051997
FBgn0052006
FBgn0052037
FBgn0052073
FBgn0052702
FBgn0052783
FBgn0053481
FBgn0053775
FBgn0058470
FBgn0065110
FBgn0069969
FBgn0085245
FBgn0085298
FBgn0085483
FBgn0087040
FBgn0250819
FBgn0250904
FBgn0250907
FBgn0259977
FBgn0260430
FBgn0260658
FBgn0260866
FBgn0261341
FBgn0261393
FBgn0261561
FBgn0262035
FBgn0262794
FBgn0264443
FBgn0264894
FBgn0265048
FBgn0265137
FBgn0265417
FBgn0265767
FBgn0266534
FBgn0266566
FBgn0267408
FBgn0285896
FBgn0287209

Appendix Table S3. Genes downregulated in Lint-O KD

FBgn0000116	FBgn0011726	FBgn0026666	FBgn0031651	FBgn0034447	FBgn0036686	FBgn0039419	FBgn0261380	FBgn0030983
FBgn0000181	FBgn0011737	FBgn0027055	FBgn0031660	FBgn0034451	FBgn0036702	FBgn0039488	FBgn0261535	FBgn0031227
FBgn0000250	FBgn0011762	FBgn0027057	FBgn0031673	FBgn0034494	FBgn0036734	FBgn0039602	FBgn0261560	FBgn0031805
FBgn0000253	FBgn0013269	FBgn0027079	FBgn0031710	FBgn0034528	FBgn0036754	FBgn0039626	FBgn0261565	FBgn0031857
FBgn0000261	FBgn0013432	FBgn0027081	FBgn0031736	FBgn0034534	FBgn0036805	FBgn0039636	FBgn0261596	FBgn0031888
FBgn0000326	FBgn0014020	FBgn0027082	FBgn0031832	FBgn0034579	FBgn0036828	FBgn0039654	FBgn0261599	FBgn0033592
FBgn0000370	FBgn0014028	FBgn0027086	FBgn0031851	FBgn0034617	FBgn0036838	FBgn0039735	FBgn0261822	FBgn0033725
FBgn0000395	FBgn0014163	FBgn0027088	FBgn0031881	FBgn0034631	FBgn0036886	FBgn0039740	FBgn0261931	FBgn0035158
FBgn0000412	FBgn0014366	FBgn0027090	FBgn0031902	FBgn0034703	FBgn0036892	FBgn0039765	FBgn0261933	FBgn0035189
FBgn0000426	FBgn0014411	FBgn0027091	FBgn0031992	FBgn0034724	FBgn0036916	FBgn0039828	FBgn0261984	FBgn0035281
FBgn0000560	FBgn0014877	FBgn0027094	FBgn0032016	FBgn0034741	FBgn0036973	FBgn0039868	FBgn0262081	FBgn0035727
FBgn0000579	FBgn0015000	FBgn0027330	FBgn0032034	FBgn0034792	FBgn0037011	FBgn0039909	FBgn0262169	FBgn0036597
FBgn0001092	FBgn0015024	FBgn0027554	FBgn0032050	FBgn0034802	FBgn0037012	FBgn0039929	FBgn0262527	FBgn0037672
FBgn0001139	FBgn0015218	FBgn0027556	FBgn0032051	FBgn0034858	FBgn0037019	FBgn0039993	FBgn0262601	FBgn0037941
FBgn0001149	FBgn0015221	FBgn0027587	FBgn0032101	FBgn0034877	FBgn0037051	FBgn0040064	FBgn0262732	FBgn0040256
FBgn0001168	FBgn0015222	FBgn0027599	FBgn0032138	FBgn0034914	FBgn0037073	FBgn0040066	FBgn0262734	FBgn0040259
FBgn0001186	FBgn0015229	FBgn0027619	FBgn0032198	FBgn0034915	FBgn0037081	FBgn0040075	FBgn0262736	FBgn0040609
FBgn0001202	FBgn0015245	FBgn0027785	FBgn0032217	FBgn0034918	FBgn0037102	FBgn0040271	FBgn0262954	FBgn0041087
FBgn0001215	FBgn0015268	FBgn0027836	FBgn0032222	FBgn0034925	FBgn0037137	FBgn0040309	FBgn0263133	FBgn0042173
FBgn0001218	FBgn0015288	FBgn0027932	FBgn0032229	FBgn0034982	FBgn0037248	FBgn0040319	FBgn0263200	FBgn0043841
FBgn0001220	FBgn0015527	FBgn0028331	FBgn0032236	FBgn0035020	FBgn0037293	FBgn0040389	FBgn0263396	FBgn0051547
FBgn0001247	FBgn0015754	FBgn0028341	FBgn0032248	FBgn0035027	FBgn0037382	FBgn0040394	FBgn0263510	FBgn0063491
FBgn0001258	FBgn0015765	FBgn0028429	FBgn0032258	FBgn0035039	FBgn0037391	FBgn0040529	FBgn0263594	FBgn0259243
FBgn0001301	FBgn0015776	FBgn0028473	FBgn0032261	FBgn0035063	FBgn0037487	FBgn0040985	FBgn0263605	FBgn0263118
FBgn0001316	FBgn0015778	FBgn0028504	FBgn0032296	FBgn0035085	FBgn0037489	FBgn0041147	FBgn0263740	FBgn0264907
FBgn0001341	FBgn0015790	FBgn0028527	FBgn0032321	FBgn0035121	FBgn0037543	FBgn0041184	FBgn0263773	
FBgn0001961	FBgn0015795	FBgn0028690	FBgn0032346	FBgn0035131	FBgn0037555	FBgn0041210	FBgn0263929	
FBgn0002031	FBgn0015797	FBgn0028744	FBgn0032397	FBgn0035137	FBgn0037561	FBgn0041604	FBgn0264006	
FBgn0002413	FBgn0015829	FBgn0028836	FBgn0032408	FBgn0035165	FBgn0037608	FBgn0041629	FBgn0264294	
FBgn0002576	FBgn0016041	FBgn0028919	FBgn0032476	FBgn0035173	FBgn0037637	FBgn0042693	FBgn0265003	
FBgn0002780	FBgn0016685	FBgn0028990	FBgn0032487	FBgn0035205	FBgn0037652	FBgn0042712	FBgn0265297	
FBgn0003206	FBgn0016694	FBgn0029117	FBgn0032512	FBgn0035213	FBgn0037710	FBgn0044323	FBgn0265574	
FBgn0003231	FBgn0016797	FBgn0029133	FBgn0032514	FBgn0035232	FBgn0037743	FBgn0046214	FBgn0265935	
FBgn0003257	FBgn0017578	FBgn0029664	FBgn0032586	FBgn0035295	FBgn0037773	FBgn0046685	FBgn0266284	
FBgn0003274	FBgn0019624	FBgn0029672	FBgn0032587	FBgn0035336	FBgn0037794	FBgn0050000	FBgn0266581	
FBgn0003275	FBgn0020238	FBgn0029689	FBgn0032620	FBgn0035337	FBgn0037852	FBgn0050035	FBgn0266668	
FBgn0003278	FBgn0020278	FBgn0029755	FBgn0032703	FBgn0035346	FBgn0037891	FBgn0050104	FBgn0266669	
FBgn0003310	FBgn0020388	FBgn0029766	FBgn0032728	FBgn0035357	FBgn0037899	FBgn0050159	FBgn0266673	
FBgn0003360	FBgn0020414	FBgn0029785	FBgn0032731	FBgn0035383	FBgn0037958	FBgn0050349	FBgn0266720	
FBgn0003495	FBgn0020440	FBgn0029878	FBgn0032799	FBgn0035393	FBgn0038020	FBgn0050476	FBgn0267347	
FBgn0003512	FBgn0020443	FBgn0029887	FBgn0032849	FBgn0035422	FBgn0038057	FBgn0050493	FBgn0267385	
FBgn0003525	FBgn0020445	FBgn0029894	FBgn0032859	FBgn0035425	FBgn0038146	FBgn0051111	FBgn0267975	
FBgn0003660	FBgn0020647	FBgn0030037	FBgn0032886	FBgn0035438	FBgn0038194	FBgn0051223	FBgn0283472	
FBgn0003714	FBgn0021768	FBgn0030052	FBgn0032891	FBgn0035440	FBgn0038196	FBgn0051361	FBgn0283659	
FBgn0003748	FBgn0021874	FBgn0030060	FBgn0032901	FBgn0035462	FBgn0038224	FBgn0051363	FBgn0283680	
FBgn0003885	FBgn0021895	FBgn0030061	FBgn0032919	FBgn0035488	FBgn0038235	FBgn0051852	FBgn0283681	
FBgn0003888	FBgn0021995	FBgn0030067	FBgn0032925	FBgn0035519	FBgn0038271	FBgn0052066	FBgn0284246	
FBgn0003984	FBgn0022029	FBgn0030177	FBgn0032974	FBgn0035524	FBgn0038275	FBgn0052103	FBgn0284248	
FBgn0004050	FBgn0022069	FBgn0030208	FBgn0032981	FBgn0035541	FBgn0038294	FBgn0052176	FBgn0284251	
FBgn0004177	FBgn0022238	FBgn0030291	FBgn0032988	FBgn0035589	FBgn0038306	FBgn0052267	FBgn0284253	
FBgn0004359	FBgn0022288	FBgn0030321	FBgn0033000	FBgn0035591	FBgn0038307	FBgn0052344	FBgn0285937	
FBgn0004465	FBgn0022893	FBgn0030364	FBgn0033038	FBgn0035600	FBgn0038369	FBgn0052409	FBgn0286786	
FBgn0004556	FBgn0022959	FBgn0030479	FBgn0033226	FBgn0035603	FBgn0038400	FBgn0052418	FBgn0287225	
FBgn0004635	FBgn0022984	FBgn0030482	FBgn0033235	FBgn0035630	FBgn0038454	FBgn0052533	FBgn0288470	
FBgn0004638	FBgn0023000	FBgn0030569	FBgn0033260	FBgn0035656	FBgn0038455	FBgn0052649	FBgn0000308	
FBgn0004657	FBgn0023091	FBgn0030588	FBgn0033304	FBgn0035714	FBgn0038472	FBgn0052672	FBgn0002645	
FBgn0004828	FBgn0023177	FBgn0030598	FBgn0033379	FBgn0035760	FBgn0038473	FBgn0053100	FBgn0005775	
FBgn0004901	FBgn0023212	FBgn0030603	FBgn0033395	FBgn0035805	FBgn0038488	FBgn0053158	FBgn0014388	
FBgn0004907	FBgn0023214	FBgn0030605	FBgn0033428	FBgn0035853	FBgn0038519	FBgn0053180	FBgn0024183	
FBgn0004926	FBgn0023514	FBgn0030608	FBgn0033454	FBgn0035866	FBgn0038585	FBgn0053329	FBgn0026160	
FBgn0005278	FBgn0023519	FBgn0030672	FBgn0033483	FBgn0035877	FBgn0038593	FBgn0053542	FBgn0026319	
FBgn0005616	FBgn0023527	FBgn0030687	FBgn0033485	FBgn0035878	FBgn0038666	FBgn0062449	FBgn0028541	
FBgn0005648	FBgn0023542	FBgn0030692	FBgn0033507	FBgn0035901	FBgn0038678	FBgn0085339	FBgn0029896	
FBgn0005672	FBgn0024245	FBgn0030720	FBgn0033540	FBgn0036000	FBgn0038742	FBgn0086347	FBgn0030318	
FBgn0010038	FBgn0024321	FBgn0030752	FBgn0033544	FBgn0036018	FBgn0038746	FBgn0086443	FBgn0030870	
FBgn0010173	FBgn0024362	FBgn0030850	FBgn0033547	FBgn0036035	FBgn0038768	FBgn0086656	FBgn0032640	
FBgn0010220	FBgn0024364	FBgn0030853	FBgn0033548	FBgn0036038	FBgn0038787	FBgn0086711	FBgn0035023	
FBgn0010235	FBgn0024556	FBgn0030854	FBgn0033571	FBgn0036124	FBgn0038854	FBgn0086855	FBgn0035049	
FBgn0010238	FBgn0024993	FBgn0030863	FBgn0033649	FBgn0036126	FBgn0038869	FBgn0086904	FBgn0035101	
FBgn0010278	FBgn0025366	FBgn0030894	FBgn0033737	FBgn0036173	FBgn0038890	FBgn0086912	FBgn0035470	
FBgn0010333	FBgn0025394	FBgn0030904	FBgn0033741	FBgn0036187	FBgn0038893	FBgn0250785	FBgn0036316	
FBgn0010340	FBgn0025582	FBgn0030969	FBgn0033816	FBgn0036207	FBgn0038964	FBgn0259152	FBgn0039257	
FBgn0010348	FBgn0025608	FBgn0031040	FBgn0033907	FBgn0036237	FBgn0038989	FBgn0259481	FBgn0039829	
FBgn0010389	FBgn0025674	FBgn0031043	FBgn0033918	FBgn0036258	FBgn0039004	FBgn0259711	FBgn0039858	
FBgn0010409	FBgn0025781	FBgn0031047	FBgn0033989	FBgn0036333	FBgn0039109	FBgn0259742	FBgn0250753	
FBgn0010504	FBgn0025809	FBgn0031057	FBgn0034029	FBgn0036334	FBgn0039175	FBgn0259832	FBgn0263256	
FBgn0010551	FBgn0025830	FBgn0031114	FBgn0034049	FBgn0036500	FBgn0039205	FBgn0259878	FBgn0285963	
FBgn0010611	FBgn0025832	FBgn0031238	FBgn0034065	FBgn0036512	FBgn0039207	FBgn0259937	FBgn0286051	
FBgn0010651	FBgn0025879	FBgn0031281	FBgn0034086	FBgn0036514	FBgn0039223	FBgn0260407	FBgn0000037	
FBgn0010770	FBgn0026079	FBgn0031321	FBgn0034113	FBgn0036516	FBgn0039252	FBgn0260456	FBgn0002783	
FBgn0010774	FBgn0026084	FBgn0031392	FBgn0034118	FBgn0036522	FBgn0039259	FBgn0260632	FBgn0003285	
FBgn0010786	FBgn0026196	FBgn0031456	FBgn0034232	FBgn0036570	FBgn0039274	FBgn0260648	FBgn0004034	
FBgn0010803	FBgn0026250	FBgn0031493	FBgn0034243	FBgn0036580	FBgn0039303	FBgn0260755	FBgn0004436	
FBgn0010808	FBgn0026252	FBgn0031500	FBgn0034259	FBgn0036614	FBgn0039306	FBgn0260855	FBgn0015037	
FBgn0010926	FBgn0026317	FBgn0031540	FBgn0034345	FBgn0036627	FBgn0039350	FBgn0260936	FBgn0022349	
FBgn0011204	FBgn0026372	FBgn0031597	FBgn0034401	FBgn0036661	FBgn0039358	FBgn0261258	FBgn0029804	
FBgn0011638	FBgn0026378	FBgn0031643	FBgn0034432	FBgn0036666	FBgn0039407	FBgn0261286	FBgn0030373	

Appendix table S4

Related to Figs 5-6 and Figs EV5. Experimental genotypes used in this study.

Fig 5B-5E	
yw	y^{1} w^{1118}
Lint-O^{KO}	y^{1} w^{1118} Lint-O^{KO} / y^{1} w^{1118} Lint-O^{KO}
Fig 6A, 6B, 6D-6G	
yw	y^{1} w^{1118},<br>y^{1} w^{1118} / Y
Lint-O^{KO}	y^{1} w^{1118} Lint-O^{KO} / y^{1} w^{1118} Lint-O^{KO},<br>y^{1} w^{1118} Lint-O^{KO} / Y
L(3)mbt^{ts1}	w* ; + ; L(3)mbt^{ts1} / L(3)mbt^{ts1},<br>w* / Y ; + ; L(3)mbt^{ts1} / L(3)mbt^{ts1}
Fig 6C	
Lint-O^{KO} –/+	y^{1} w^{1118} Lint-O^{KO} / FM7 Kruppel>GFP,<br>FM7 Kruppel>GFP / FM7 Kruppel>GFP,<br>FM7 Kruppel>GFP / Y
Lint-O^{KO} –/–	y^{1} w^{1118} Lint-O^{KO} / y^{1} w^{1118} Lint-O^{KO},<br>y^{1} w^{1118} Lint-O^{KO} / Y
L(3)mbt^{ts1} –/–	w* ; + ; L(3)mbt^{ts1} / L(3)mbt^{ts1},<br>w* / Y ; + ; L(3)mbt^{ts1} / L(3)mbt^{ts1}
Fig EV5	
yw	y^{1} w^{1118},<br>y^{1} w^{1118} / Y
Lint-O^{KO}	y^{1} w^{1118} Lint-O^{KO} / y^{1} w^{1118} Lint-O^{KO},<br>y^{1} w^{1118} Lint-O^{KO} / Y
L(3)mbt^{ts1}	w* ; + ; L(3)mbt^{ts1} / L(3)mbt^{ts1},<br>w* / Y ; + ; L(3)mbt^{ts1} / L(3)mbt^{ts1}
yw Lint-O-Venus	y^{1} w^{1118} Lint-O-Venus / y^{1} w^{1118} Lint-O-Venus
