# Supplementary material for: Lint‐O cooperates with L(3)mbt in target gene suppression to maintain homeostasis in fly ovary and brain
Source: EMBO Rep. 2022 Aug 22;23(10):e53813. doi: 10.15252/embr.202153813 (PMC9535798; doi:10.15252/embr.202153813)
Supplement: Supplementary file 8 [file EMBR-23-e53813-s003.pdf]

EMBO reports

Lint-O cooperates with L(3)mbt in target gene suppression to maintain homeostasis in fly ovary and brain

Hitomi Yamamoto-Matsuda, Keita Miyoshi, Mai Moritoh, Hikari Yoshitane, Yoshitaka Fukada, Kuniaki Saito, Soichiro Yamanaka, and Mikiko Siomi
DOI: 10.15252/embr.202153813

Corresponding author(s): Mikiko Siomi (siomim@bs.s.u-tokyo.ac.jp) , Kuniaki Saito (saitok@nig.ac.jp), Soichiro Yamanaka (yamanaka@g.ecc.u-tokyo.ac.jp)

Review Timeline:

Submission Date:	7th Feb 22
Editorial Decision:	30th Mar 22
Revision Received:	4th Apr 22
Editorial Decision:	11th May 22
Revision Received:	14th Jun 22
Editorial Decision:	2nd Aug 22
Revision Received:	8th Aug 22
Accepted:	9th Aug 22

Editor: Esther Schnapp

Transaction Report:

(Note: With the exception of the correction of typographical or spelling errors that could be a source of ambiguity, letters and reports are not edited. Depending on transfer agreements, referee reports obtained elsewhere may or may not be included in this compilation. Referee reports are anonymous unless the Referee chooses to sign their reports.)

29th Mar 2022
Dear Mikiko,

Thank you for your patience while your manuscript was peer-reviewed at EMBO reports. We have now received 2 referee reports on it.

I am sorry to say that the evaluation of your manuscript is not a positive one. As you will see, both referees point out that no mechanistic insight is provided, that it remains unclear how Lint-O and L(3)mbt act on chromatin, and that the data presentation and analyses could be improved. Referee 1 rates the novelty and interest of the study "low" in the manuscript table that is directly sent to the editor, and referee 2 rates the technical quality "low".

Given these comments from 2 experts in the field, and the time that would be required to address them all, I am sorry to say that we have decided that we cannot offer to publish your manuscript.

While we cannot pursue this manuscript further at EMBO reports, we encourage you to transfer your study to our not-for-profit open-access sister journal, Life Science Alliance (LSA). We shared your manuscript and the accompanying reviews with LSA Executive Editor, Eric Sawey, who is interested in these findings, and would like to invite further consideration of this manuscript at LSA pending the following revisions: - Address Reviewer 1's minor points. - Address Reviewer 2's comments, excluding Major Comment # 6. We encourage you to use the link below to transfer your manuscript to LSA. You do not need to revise the manuscript before transferring it to LSA. Once you transfer, Dr. Sawey will email you an invitation to revise and resubmit, listing the same revision requests as mentioned above. Please feel free to reach out at e.sawey@life-science-alliance.org if you have any questions about the LSA journal, the transfer process or the revisions requested.

I am sorry that I cannot be more positive for EMBO reports, and hope that the transfer proposal to Life Science Alliance is a good way forward for you.

Best wishes,
Esther

Esther Schnapp, PhD
Senior Editor
EMBO reports

Referee #1:

This manuscript describes the identification of a new interactor of L(3)Mbt, Lint-O isolated from OSC cells. The data presented show: 1. That L(3)Mbt and Lint-O bind to the same gene regions and affect the expression of about 800 genes in OSC cells. These include a number of genes involved in piRNA regulation. 2. That L(3)Mbt and Lint-O interact with each other through the SAM domain and that Lint-O stability depends on its binding to L(3)Mbt. 3. That Lint-O KO has fertility and neural tumor phenotypes similar to those of L(3)Mbt.

Overall, the paper was generally well written and easy to follow and the authors don't overstate or overinterpret their findings. With the discovery of Lint-O the study largely extend previous knowledge but new mechanistic details are not revealed. We still do not know how L(3)Mbt or L(3)Mbt, Lint-O select their targets or the mechanism of the repressive effect on their targets.

The findings in the paper are interesting, however t would help if the authors could consider the following additional experiments:

Major points:
1. How do LINT-O and L(3)mbt interact at genomic loci? Does LINT-O help localize L(3)mbt to genomic sites, or vice versa? Can the authors perform ChIP-seq on L(3)mbt in LINT-O mutant cells to determine if loss of Lint-O prevents localization of L(3)mbt to shared target regions in the genome (such as the vasa promoter region). (The reverse experiment can likely not be performed to ask if L(3)mbt helps localize LINT-O to these regions, as loss of L(3)mbt destabilizes LINT-O protein).
2. Where is Lint-O expressed in the fly? Is the ovarian phenotype due to loss of germ line or somatic expression of Lint-O, or both (as was shown for L(3)Mbt? Does loss of piwi, vasa, aub, or nos prevent brain tumor growth in LINT-O mutants as it does in L(3)mbt mutants? Performing a quantitative measurement of brain volume (as in Figure 6C) in LINT-O mutants upon piwi, vasa, aub, and nos knockdowns would be helpful analysis for comparing L(3)mbt and LINT-O mutants.

Minor point:
1. In figure 3B, there is an increase in binding of the 8CA LINT-O mutant to L(3)mbt. Is this increase significant and can the

authors discuss this further? Does complete loss of the PHD Finger domain also result in a similar increase in binding?
2. A reorganization of the text that relates to figure 3 would be helpful for clarity of the data. For example, it would be helpful if the authors introduce the 8CA mutant when they talk about the IP in figure 3B, rather than later in the text.
3. When discussing expression of Aub or AGO3, is it possible that L(3)mbt has an indirect effect on their expression? Since these two genes were categorized in the "unbound" and "non-promoter mediated" groups, one possible hypothesis is that depletion of L(3)mbt activates a transcription factor or other regulator that may then impact AGO3/aub expression. This hypothesis should be discussed.

Referee #2:

Comments for Yamamoto-Matsuda et al., "Lint-O cooperates with L(3)mbt in gene regulation to maintain homeostasis in Drosophila ovary and brain"

The authors isolated the L(3)mbt complex from OSCs and ran mass spectrometric analysis revealing a new L(3)mbt interactor Lint-O, which interacts with L(3)mbt via SAM domains and negatively regulates expression of vasa, aub, and ago3 genes in somatic cells. They generated transgenic lint-o KO flies which exhibit sterile ovary and brain morphology similar to l(3)mbt KO flies and upregulate vasa and aub in somatic cells of both tissues. This part of the paper reveals a new finding about the L(3)mbt complex, its interactor Lint-O in repression of aub, vasa and ago3 expression. ChIP-seq and RNA-seq experiments show that the repressor function of L(3)mbt and Lint-O is only specific to a minority of genes while the majority of genes with L(3)mbt and Lint-O binding do not change in expression upon L(3)mbt or Lint-O depletion and some are downregulated. The results from these studies do not identify mechanisms behind these changes. It is still unclear whether these gene expression changes are a result of a direct loss of L(3)mbt or Lint-O repression at these genes or other causes. The genomics analysis in this paper is not convincing and many analyses and terminologies reported in the genomics part of the paper need to be modified or removed.

Major comments:

1) In this manuscript the authors report the RNA-seq results from L(3)mbt knock-down (KD) cells (RNAi). However, the same group previously published a paper where they generated an L(3)mbt-deletion line in OSCs using CRISPR/Cas9 (PMID: 27474440). Why haven't authors used this L(3)mbt-deletion OSC line instead of RNAi experiments?

2) Page 8: The authors say that "we further classified these genes according to where in each gene L(3)mbt is bound" and go on to group the genes into "promoter-mediated" and "non-promoted mediated" genes in Figure 1. It's misleading to call the genes as "promoter-mediated" simply because there is L(3)mbt ChIP-seq signal in the promoter region and the rest as "non-promoter mediated" just because the signal is not detected within the promoters especially given the fact that most of the L(3)mbt-promoter-bound genes do not change in expression upon L(3)mbt depletion. The terminology adds further confusion since genes termed as "non-promoter-mediated" genes could be promoter-mediated without L(3)mbt. In order to show that l(3)mbt is indeed performing a promoter-mediated gene regulation it is important to show this with experimental manipulation where the binding event is disturbed or with luciferase reporter assay and similar experiments. To avoid confusion, the authors should rephrase these terms to "promoter-region binding" and the "non-promoter genic binding" instead of saying "mediated" as it implies gene regulation. The "non-promoter genic binding" could be further subdivided into intronic and exonic or could be classified based on the distance to TSS (e.g., 1kb, 5kb to TSS etc).

3) Page 8: What fold-change values were used to classify upregulated, downregulated and unchanged genes? In the methods section the authors mention that FDR q-value of <0.01 was used, however the log2FC threshold for defining upregulated, downregulated and unchanged genes is not mentioned. It is also important to at least indicate the fold-change difference in gene expression for genes that are mentioned throughout the text (ago3, vasa, aub etc.). The authors should have these numbers from their edgeR analysis results. The differential gene expression results from edgeR should be included in the supplementary data or table.

4) Page 8 and Methods: In order to show consistency between RNA-seq replicates it is important to run the principal component analysis (PCA) to show how replicates group together in wild-type OSCs and in l(3)mbt-KD OSCs instead of simply looking at FPKM correlations.

5) Page 9: The authors mention that "L(3)mbt binds to a relatively wide range of genes, but for genes functioning in OSCs, there appears to be activators that override the repression by L(3)mbt." Yet, this still doesn't explain why there is almost as many promoter-L(3)mbt-bound genes that are down-regulated in response to L(3)mbt repletion (n=1044) as there are up-regulated genes (n=1202) if it indeed functions as a repressor. L(3)mbt was previously shown to also function as an insulator which could explain some of these effects (PMID: 21857667; PMID: 22722341). These could be secondary effects from L(3)mbt-induced repression of other genes.

6) Page 10: The authors state "One plausible scenario is that, when L(3)mbt controls target genes via the promoter (e.g., vasa),

it is almost independent of both LINT and dREAM complexes, but when L(3) controls target genes via regions other than the promoter (e.g., aub and AGO3), it may subtly depend on the LINT complex." -- Can the authors check the expression levels of the other promoter-L(3)mbt-bound genes such as tej, boot or qin in knock-down samples to test this hypothesis?

7) As with L(3)mbt in Figure 1, the Lint-O terminology for binding regions in Figure 4A should be revised from "promoter-mediated" to "promoter-bound" and from "non-promoter mediated" to "non-promoter genic binding" to clarify what the terms actually mean and to avoid any confusion.

8) How man ChIP-seq peaks are there for Lint-O ChIP-seq and how many of these are common with L(3)mbt ChIP-seq peaks and how many are unique? It would be helpful to have a heatmap to visualize all these regions and a Venn diagram to show peak overlaps.

9) On page 15, the authors claim that "L(3)mbt and Lint-O may rarely repress target genes by binding to introns (e.g., ago3) or upstream regions far from the TSS (e.g., aub)." -- Yet, their results show that the majority of promoter-bound regions by Lint-O and/or L(3)mbt do not change in expression and almost as many are downregulated as upregulated. The repression function alone can be hardly justified in this case let alone the effect of repression at distance. Moreover, the "non-promoter" group usually has >4-fold more upregulated genes than the down-regulated genes for both Lint-O and L(3)mbt. This would rather support the opposite claim that the intronic binding is more likely to be repressive than promoter binding. Since L(3)mbt also functions as an insulator it would be hard to infer the gene regulatory mechanisms such as direct repression from these analyses without any additional experiments such as deletion of the L(3)mbt binding sites at the promoter and non-promoter regions followed by RT-qPCR or RNA-seq of the genes or using artificial constructs such as luciferase reporter assays.

10) For Figure 4F it would be better to plot upregulated and downregulated genes separately and running GO analysis separately on these two groups instead of lumping all together into one "L(3)mbt/Lint-O-dependent" category. The upregulated genes might just belong to a different pathway than downregulated genes and combining them together could make GO associations weaker.

Minor comments:

a) Page 7: An overview of the ChIP-seq reads mapped on the Drosophila genome in Fig EV2A is not presented in an informative way. Instead, the authors should give a total number of L(3)mbt peaks, a distance distribution of L(3)mbt peaks to gene promoters (e.g. promoter, intronic, exonic, distal intergenic) and plot reads with a heatmap to visualize the L(3)mbt ChIP-seq signals between gene promoters in the way they do in Figure 1E.

b) Page 6 end of introduction: Lint-O is not a co-suppressor of L(3)mbt. This could imply that Lint-O suppresses L(3)mbt. Modify to "Lint-O interacts with L(3)mbt to suppress specific genes"?

c) The data in Figures 1B, 1C and 1D is redundant and included in the data in Figure 4B, 4C, and 4D since both show the same L(3)mbt ChIP-seq and L(3)mbt-KD RNA-seq data tracks. This should be clearly stated, or figures 1 and 4 could be combined into one figure instead or Lint-O ChIP-seq data moved to supplementary.

d) In Figure EV4F the authors compare FPKM from RNA-seq data of their experiments to the FlyAtlas 2 FPKM data which is not a recommended approach. The FPKM is used for "in-sample normalization" and can only be used to compare genes within one sample while "across samples normalized" reads are obtained with methods such as edgeR and Deseq. The authors compared their FPKM to a different dataset from a different group which was analysed with different tools. For a consistent and proper analysis, the authors must download and process the raw RNA-seq data in the same way they processed their own data and run "across sample normalization" together with their own data using tools such as edgeR or Deseq and only then can compare the data across samples and across different datasets.

e) Why only the L(3)mbt-S isoform was used in the co-IP experiment in Figure 3D? Would the L(3)mbt-L isoform similarly co-immunoprecipitate with Lint-O from the OSC lysates?

** As a service to authors, EMBO Press provides authors with the ability to transfer a manuscript that one journal cannot offer to publish to another journal, without the author having to upload the manuscript data again. To transfer your manuscript to another EMBO Press journal using this service, please click on Link Not Available

Referee #1:

Major points:

1) *How do LINT-O and L(3)mbt interact at genomic loci? Does LINT-O help localize L(3)mbt to genomic sites, or vice versa? Can the authors perform ChIP-seq on L(3)mbt in LINT-O mutant cells to determine if loss of Lint-O prevents localization of L(3)mbt to shared target regions in the genome (such as the vasa promoter region). (The reverse experiment can likely not be performed to ask if L(3)mbt helps localize LINT-O to these regions, as loss of L(3)mbt destabilizes LINT-O protein).*

We already performed ChIP-PCR and found that the loss of Lint-O significantly weakened the binding activity of L(3)mbt to the *vasa* promoter. This suggests that the genomic binding L(3)mbt is dependent on Lint-O. As the referee kindly noted, the reverse experiment deemed meaningless because Lint-O disappeared when L(3)mbt is no longer expressed.

2) *Where is Lint-O expressed in the fly? Is the ovarian phenotype due to loss of germ line or somatic expression of Lint-O, or both (as was shown for L(3)Mbt? Does loss of piwi, vasa, aub, or nos prevent brain tumor growth in LINT-O mutants as it does in L(3)mbt mutants? Performing a quantitative measurement of brain volume (as in Figure 6C) in LINT-O mutants upon piwi, vasa, aub, and nos knockdowns would be helpful analysis for comparing L(3)mbt and LINT-O mutants.*

We immunostained cultured OSCs and fly ovaries with anti-Lint-O antibody, but no signal was detected. Therefore, the gene encoding Venus was knocked in at the *lint-O* genomic loci and Lint-O-Venus was expressed in the ovaries. The fluorescent signals were detected in both germ and somatic cells, suggesting that both cell types express Lint-O. It is not yet known whether the ovarian phenotype is due to loss of Lint-O expression in germ, somatic, or both. The Ruth Lehmann group previously reported that "*somatic expression of L(3)mbt is important for ovarian morphological maintenance*" (Coux *et al. Development* 2018). Therefore, it is speculated that the ovarian defective phenotype caused by the loss of Lint-O is *due to the loss of "somatic" expression of Lint-O*. We can test this by experiments (although this experiment may take several months).

We are planning to quantitatively measure brain volume in *lint-O* mutants upon *piwi, vasa, aub,* or *nos* knockdown. However, it is very time consuming and beyond the scope of the manuscript, so will be reported separately in a future paper.

Minor points:

1. In figure 3B, there is an increase in binding of the 8CA LINT-O mutant to L(3)mbt. Is this increase significant and can the authors discuss this further? Does complete loss of the PHD Finger domain also result in a similar increase in binding?

At this point, we do not know the reason for the increased binding of the Lint-O 8CA mutant to L(3)mbt. We will discuss it in the revised manuscript upon investigating how the binding of Lint-O to L(3)mbt is influenced by the complete loss of the PHD finger domain.

2. A reorganization of the text that relates to figure 3 would be helpful for clarity of the data. For example, it would be helpful if the authors introduce the 8CA mutant when they talk about the IP in figure 3B, rather than later in the text.

We will reorganize the text accordingly.

3. When discussing expression of Aub or AGO3, is it possible that L(3)mbt has an indirect effect on their expression? Since these two genes were categorized in the "unbound" and "non-promoter mediated" groups, one possible hypothesis is that depletion of L(3)mbt activates a transcription factor or other regulator that may then impact AGO3/aub expression. This hypothesis should be discussed.

The possibility that depletion of L(3)mbt activates transcription factors and/or other regulators that impact AGO3/Aub expression will be discussed in the revised manuscript.

Referee #2:

Major comments:

1) *In this manuscript the authors report the RNA-seq results from L(3)mbt knock-down (KD) cells (RNAi). However, the same group previously published a paper where they generated an L(3)mbt-deletion line in OSCs using CRISPR/Cas9 (PMID: 27474440). Why haven't authors used this L(3)mbt-deletion OSC line instead of RNAi experiments?*

The reason was that we wanted to compare RNA-seq of Lint-O-knockdown OSCs with that of L(3)mbt-knockdown OSCs. We do not yet have a Lint-O-deletion OSC line for comparison.

2) *Page 8: The authors say that "we further classified these genes according to where in each gene L(3)mbt is bound" and go on to group the genes into "promoter-mediated" and "non-promoted mediated" genes in Figure 1. It's misleading to call the*

genes as "promoter-mediated" simply because there is L(3)mbt ChIP-seq signal in the promoter region and the rest as "non-promoter mediated" just because the signal is not detected within the promoters especially given the fact that most of the L(3)mbt-promoter-bound genes do not change in expression upon L(3)mbt depletion. The terminology adds further confusion since genes termed as "non-promoter-mediated" genes could be promoter-mediated without L(3)mbt. In order to show that l(3)mbt is indeed performing a promoter-mediated gene regulation it is important to show this with experimental manipulation where the binding event is disturbed or with luciferase reporter assay and similar experiments. To avoid confusion, the authors should rephrase these terms to "promoter-region binding" and the "non-promoter genic binding" instead of saying "mediated" as it implies gene regulation. The "non-promoter genic binding" could be further subdivided into intronic and exonic or could be classified based on the distance to TSS (e.g., 1kb, 5kb to TSS etc).

To avoid unnecessary confusion, we will rephrase *"promoter-mediated"* to "*promoter-region binding*" and *"non-promoter-mediated"* to "*non-promoter genic binding*" in accordance with the referee's suggestions. Also, we will subdivide the "*non-promoter genic binding*" further into intronic and exonic.

3) *Page 8: What fold-change values were used to classify upregulated, downregulated and unchanged genes? In the methods section the authors mention that FDR q-value of <0.01 was used, however the log2FC threshold for defining upregulated, downregulated and unchanged genes is not mentioned. It is also important to at least indicate the fold-change difference in gene expression for genes that are mentioned throughout the text (ago3, vasa, aub etc.). The authors should have these numbers from their edgeR analysis results. The differential gene expression results from edgeR should be included in the supplementary data or table.*

In the RNA-seq analysis, genes were extracted only by the threshold of q-value, and if the fold change was greater than 0, the gene was categorized as "upregulated", and conversely, if the fold change was less than 0, the gene was categorized as "downregulated". Others were categorized as "unchanged". We will indicate the fold-change difference in gene expression for genes that are mentioned throughout the revised text (i.e., *AGO3*, *vasa*, *aub*, and others). The differential gene expression results from edgeR will also be included as the supplementary data.

4) *Page 8 and Methods: In order to show consistency between RNA-seq replicates it is important to run the principal component analysis (PCA) to show how replicates*

group together in wild-type OSCs and in l(3)mbt-KD OSCs instead of simply looking at FPKM correlations.

We will show consistency between RNA-seq replicates by running the principal component analysis (PCA).

5) *Page 9: The authors mention that "L(3)mbt binds to a relatively wide range of genes, but for genes functioning in OSCs, there appears to be activators that override the repression by L(3)mbt." Yet, this still doesn't explain why there is almost as many promoter-L(3)mbt-bound genes that are down-regulated in response to L(3)mbt repletion (n=1044) as there are up-regulated genes (n=1202) if it indeed functions as a repressor. L(3)mbt was previously shown to also function as an insulator which could explain some of these effects (PMID: 21857667; PMID: 22722341). These could be secondary effects from L(3)mbt-induced repression of other genes.*

The issue of insulator effects will be discussed in the revised manuscript by citing the papers (PMID: 21857667 and PMID: 22722341).

6) *Page 10: The authors state "One plausible scenario is that, when L(3)mbt controls target genes via the promoter (e.g., vasa), it is almost independent of both LINT and dREAM complexes, but when L(3) controls target genes via regions other than the promoter (e.g., aub and AGO3), it may subtly depend on the LINT complex." -- Can the authors check the expression levels of the other promoter-L(3)mbt-bound genes such as tej, boot or qin in knock-down samples to test this hypothesis?*

It can be done. However, extending the scope to *tej*, *boot or qin* does not cover all relevant factors and thus does not get us out of the realm of prediction.

7) *As with L(3)mbt in Figure 1, the Lint-O terminology for binding regions in Figure 4A should be revised from "promoter-mediated" to "promoter-bound" and from "non-promoter mediated" to "non-promoter genic binding" to clarify what the terms actually mean and to avoid any confusion.*

We will change the terms accordingly.

8) *How man ChIP-seq peaks are there for Lint-O ChIP-seq and how many of these are common with L(3)mbt ChIP-seq peaks and how many are unique? It would be helpful to have a heatmap to visualize all these regions and a Venn diagram to show peak overlaps.*

The heatmap was already shown in Figure 4E. In addition, we will include a Venn diagram to show peak overlaps (with numbers).

9) *On page 15, the authors claim that "L(3)mbt and Lint-O may rarely repress target genes by binding to introns (e.g., ago3) or upstream regions far from the TSS (e.g., aub)." -- Yet, their results show that the majority of promoter-bound regions by Lint-O and/or L(3)mbt do not change in expression and almost as many are downregulated as upregulated. The repression function alone can be hardly justified in this case let alone the effect of repression at distance. Moreover, the "non-promoter" group usually has >4-fold more upregulated genes than the down-regulated genes for both Lint-O and L(3)mbt. This would rather support the opposite claim that the intronic binding is more likely to be repressive than promoter binding. Since L(3)mbt also functions as an insulator it would be hard to infer the gene regulatory mechanisms such as direct repression from these analyses without any additional experiments such as deletion of the L(3)mbt binding sites at the promoter and non-promoter regions followed by RT-qPCR or RNA-seq of the genes or using artificial constructs such as luciferase reporter assays.*

This study was not intended to compare "intronic binding" vs "promoter binding" to see which is more repressive. Therefore, we do not understand clearly why the referee stated "*This would rather support the opposite claim that the intronic binding is more likely to be repressive than promoter binding."*

A similar question was raised by Referee1 as to whether L(3)mbt/Lint-O regulation is direct or indirect (see Referee1 minor comment #3). As answered above, the possibility of "indirect" issue will be discussed in the revised manuscript. Even if we have obtained some insight by performing a *luc* assay using the promoter of one gene (e.g., *vasa*), it remains to be unclear whether the same can be said for other genes, even in the same category, so the *luc* assay may be feasible but not relevant in this case.

10) *For Figure 4F it would be better to plot upregulated and downregulated genes separately and running GO analysis separately on these two groups instead of lumping all together into one "L(3)mbt/Lint-O-dependent" category. The upregulated genes might just belong to a different pathway than downregulated genes and combining them together could make GO associations weaker.*

In the revised manuscript, we will plot upregulated and downregulated genes separately and run GO analysis separately on these two groups.

Minor comments:

a) *Page 7: An overview of the ChIP-seq reads mapped on the Drosophila genome in Fig EV2A is not presented in an informative way. Instead, the authors should give a total number of L(3)mbt peaks, a distance distribution of L(3)mbt peaks to gene promoters (e.g. promoter, intronic, exonic, distal intergenic) and plot reads with a heatmap to visualize the L(3)mbt ChIP-seq signals between gene promoters in the way they do in Figure 1E.*

We will analyze genes accordingly and show data in the revised manuscript.

b) *Page 6 end of introduction: Lint-O is not a co-suppressor of L(3)mbt. This could imply that Lint-O suppresses L(3)mbt. Modify to "Lint-O interacts with L(3)mbt to suppress specific genes"?*

Lint-1 was claimed as a L(3)mbt co-repressor, but it did not mean that Lint-1 suppresses L(3)mbt. Therefore, we will leave the original statement as it is.

c) *The data in Figures 1B, 1C and 1D is redundant and included in the data in Figure 4B, 4C, and 4D since both show the same L(3)mbt ChIP-seq and L(3)mbt-KD RNA-seq data tracks. This should be clearly stated, or figures 1 and 4 could be combined into one figure instead or Lint-O ChIP-seq data moved to supplementary.*

In Figures 4B, 4C and, 4D, L(3)mbt ChIP-seq and L(3)mbt-KD RNA-seq data tracks (Figures 1B, 1C, and 1D) are re-displayed simply to compare them with Lint-O ChIP-seq and Lint-O-KD RNA-seq data tracks. This will be clearly stated in the revised figure legends.

d) *In Figure EV4F the authors compare FPKM from RNA-seq data of their experiments to the FlyAtlas 2 FPKM data which is not a recommended approach. The FPKM is used for "in-sample normalization" and can only be used to compare genes within one sample while "across samples normalized" reads are obtained with methods such as edgeR and Deseq. The authors compared their FPKM to a different dataset from a different group which was analysed with different tools. For a consistent and proper analysis, the authors must download and process the raw RNA-seq data in the same way they processed their own data and run "across sample normalization" together with their own data using tools such as edgeR or Deseq and only then can compare the data across samples and across different datasets.*

We believe that the referee meant the data in Figure EV4G but not EV4F. We will reanalyze the *nos* data according to the proposed methods.

e) Why only the L(3)mbt-S isoform was used in the co-IP experiment in Figure 3D? Would the L(3)mbt-L isoform similarly co-immunoprecipitate with Lint-O from the OSC lysates?

We previously performed co-IP experiments with the L(3)mbt-L isoform. Because the results were almost identical to those of L(3)mbt-S, only the L(3)mbt-S data were shown.

Dear Mikiko,

I am sending the official decision letter again here. I see now that you have 2 different emails. Which one should we use?
Thanks, Esther

We have now received the comments from both referees on your proposed revision plan. Both referees agree that your revisions are good, and I would therefore like to invite you to revise your manuscript for EMBO reports.

Please note that the referee concerns must be fully addressed and their suggestions taken on board. Please address all referee concerns in a complete point-by-point response. Acceptance of the manuscript will depend on a positive outcome of a second round of review. It is EMBO reports policy to allow a single round of major revision only and acceptance or rejection of the manuscript will therefore depend on the completeness of your responses included in the next, final version of the manuscript.

We realize that it is difficult to revise to a specific deadline. In the interest of protecting the conceptual advance provided by the work, we recommend a revision within 3 months (11th Aug 2022). Please discuss the revision progress ahead of this time with the editor if you require more time to complete the revisions.

IMPORTANT NOTE: we perform an initial quality control of all revised manuscripts before re-review. Your manuscript will FAIL this control and the handling will be DELAYED if the following APPLIES:
1) A data availability section providing access to data deposited in public databases is missing. If you have not deposited any data, please add a sentence to the data availability section that explains that.
2) Your manuscript contains statistics and error bars based on n=2. Please use scatter blots in these cases. No statistics should be calculated if n=2.

When submitting your revised manuscript, please carefully review the instructions that follow below. Failure to include requested items will delay the evaluation of your revision.

1) a .docx formatted version of the manuscript text (including legends for main figures, EV figures and tables). Please make sure that the changes are highlighted to be clearly visible.

2) individual production quality figure files as .eps, .tif, .jpg (one file per figure). See https://wol-prod-cdn.literatumonline.com/pb-assets/embo-site/EMBOPress_Figure_Guidelines_061115-1561436025777.pdf for more info on how to prepare your figures.

3) We replaced Supplementary Information with Expanded View (EV) Figures and Tables that are collapsible/expandable online. A maximum of 5 EV Figures can be typeset. EV Figures should be cited as 'Figure EV1, Figure EV2" etc... in the text and their respective legends should be included in the main text after the legends of regular figures.

- For the figures that you do NOT wish to display as Expanded View figures, they should be bundled together with their legends in a single PDF file called *Appendix*, which should start with a short Table of Content. Appendix figures should be referred to in the main text as: "Appendix Figure S1, Appendix Figure S2" etc. See detailed instructions regarding expanded view here:

- Additional Tables/Datasets should be labeled and referred to as Table EV1, Dataset EV1, etc. Legends have to be provided in a separate tab in case of .xls files. Alternatively, the legend can be supplied as a separate text file (README) and zipped together with the Table/Dataset file.

4) a .docx formatted letter INCLUDING the reviewers' reports and your detailed point-by-point responses to their comments. As part of the EMBO Press transparent editorial process, the point-by-point response is part of the Review Process File (RPF), which will be published alongside your paper.

5) a complete author checklist, which you can download from our author guidelines . Please insert information in the checklist that is also reflected in the manuscript. The completed author checklist will also be part of the RPF.

6) Please note that all corresponding authors are required to supply an ORCID ID for their name upon submission of a revised manuscript (). Please find instructions on how to link your ORCID ID to your account in our manuscript tracking system in our Author guidelines

7) Before submitting your revision, primary datasets produced in this study need to be deposited in an appropriate public database (see https://www.embopress.org/page/journal/14693178/authorguide#datadeposition). Please remember to provide a reviewer password if the datasets are not yet public. The accession numbers and database should be listed in a formal "Data Availability" section placed after Materials & Method (see also https://www.embopress.org/page/journal/14693178/authorguide#datadeposition). Please note that the Data Availability Section

is restricted to new primary data that are part of this study. * Note - All links should resolve to a page where the data can be accessed. *
If your study has not produced novel datasets, please mention this fact in the Data Availability Section.

8) We would also encourage you to include the source data for figure panels that show essential data. Numerical data should be provided as individual .xls or .csv files (including a tab describing the data). For blots or microscopy, uncropped images should be submitted (using a zip archive if multiple images need to be supplied for one panel). Additional information on source data and instruction on how to label the files are available at .

9) Our journal also encourages inclusion of *data citations in the reference list* to directly cite datasets that were re-used and obtained from public databases. Data citations in the article text are distinct from normal bibliographical citations and should directly link to the database records from which the data can be accessed. In the main text, data citations are formatted as follows: "Data ref: Smith et al, 2001" or "Data ref: NCBI Sequence Read Archive PRJNA342805, 2017". In the Reference list, data citations must be labeled with "[DATASET]". A data reference must provide the database name, accession number/identifiers and a resolvable link to the landing page from which the data can be accessed at the end of the reference. Further instructions are available at https://www.embopress.org/page/journal/14693178/authorguide#referencesformat

10) Regarding data quantification (see Figure Legends:
https://www.embopress.org/page/journal/14693178/authorguide#figureformat)

The following points must be specified in each figure legend:

- the name of the statistical test used to generate error bars and P values,

- the number (n) of independent experiments (please specify technical or biological replicates) underlying each data point,

- the nature of the bars and error bars (s.d., s.e.m.),

- If the data are obtained from n {less than or equal to} 2, use scatter blots showing the individual data points.

Discussion of statistical methodology can be reported in the materials and methods section, but figure legends should contain a basic description of n, P and the test applied.

- Please also include scale bars in all microscopy images.

11) The journal requires a statement specifying whether or not authors have competing interests (defined as all potential or actual interests that could be perceived to influence the presentation or interpretation of an article). In case of competing interests, this must be specified in your disclosure statement. Further information: https://www.embopress.org/competing-interests

We would also welcome the submission of cover suggestions, or motifs to be used by our Graphics Illustrator in designing a cover.

As part of the EMBO publication's Transparent Editorial Process, EMBO reports publishes online a Review Process File (RPF) to accompany accepted manuscripts. This File will be published in conjunction with your paper and will include the referee reports, your point-by-point response and all pertinent correspondence relating to the manuscript.

You are able to opt out of this by letting the editorial office know (emboreports@embo.org). If you do opt out, the Review Process File link will point to the following statement: "No Review Process File is available with this article, as the authors have chosen not to make the review process public in this case."

I look forward to seeing a revised form of your manuscript when it is ready. Please use this link to submit your revision:
https://embor.msubmit.net/cgi-bin/main.plex

Best wishes,
Esther

Esther Schnapp, PhD
Senior Editor
EMBO reports

Referee #1:

I have reviewed the response and planned experiments by Siomi et al and think these changes will improve manuscript. The identification of Lint-O is interesting and with additional analysis appropriate for EMBO reports.

Referee #2:

The authors have not made the proposed changes to the manuscript in their rebuttal however they should be allowed to resubmit once the proposed changes are made to the major comments 2, 3, 4, 5, 7, 8, and 10 and the minor comments a), c), and d) as promised in their rebuttal.

Referee 1 brought up a good point about Lint-O and L(3)mbt interaction at genomic loci in the Major point 1. The authors' response to the Referee 1's Major point 1 says that they performed ChIP-PCR and found that the loss of Lint-O significantly weakened the binding activity of L(3)mbt to the vasa promoter. These ChIP-PCR results are important and should be reported in the revised manuscript.

In the manuscript Page 10 (also major comment 6) they mention that "when L(3)mbt controls target genes via the promoter (e.g., vasa), it is almost independent of both LINT and dREAM complexes". This seems to be at odds with the above-mentioned ChIP-PCR results where the say that loss of Lint-O significantly weakened the binding activity of L(3)mbt to vasa promoter (as they mention in response to Referee 1 major point 1). The authors should report their ChIP-PCR results in the revised manuscript and should be careful when discussing these results.

With regard to their response to the major comment 9. In the manuscript page 15 the authors claim that "L(3)mbt and Lint-O may rarely repress target genes by binding to introns (e.g., ago3) or upstream regions far from the TSS (e.g., aub)." It's important to note that L(3)mbt and Lint-O binding alone is not enough to state that they are repressive as they may also function as insulators. The authors responded stating that the study was not intended to compare intronic binding to promoter binding. The authors should be careful in such interpretations if they don't have the supporting data or the supporting analysis to back it up.

Regarding to their response to the minor comment e). They should report results from the co-IP experiment with the L(3)mbt-L isoform in the revised manuscript

Referee #1:

Major points:

1) *How do LINT-O and L(3)mbt interact at genomic loci? Does LINT-O help localize L(3)mbt to genomic sites, or vice versa? Can the authors perform ChIP-seq on L(3)mbt in LINT-O mutant cells to determine if loss of Lint-O prevents localization of L(3)mbt to shared target regions in the genome (such as the vasa promoter region). (The reverse experiment can likely not be performed to ask if L(3)mbt helps localize LINT-O to these regions, as loss of L(3)mbt destabilizes LINT-O protein).*

We thank the reviewer for pointing this out. We performed ChIP-PCR and found that the loss of Lint-O significantly weakened the binding activity of L(3)mbt to the *vasa* promoter. This suggests that the genomic binding L(3)mbt is dependent on Lint-O. The result was included as revised Fig 3J. As the referee kindly noted, the reverse experiment deemed meaningless because Lint-O disappeared when L(3)mbt is no longer expressed.

2) *Where is Lint-O expressed in the fly? Is the ovarian phenotype due to loss of germ line or somatic expression of Lint-O, or both (as was shown for L(3)Mbt? Does loss of piwi, vasa, aub, or nos prevent brain tumor growth in LINT-O mutants as it does in L(3)mbt mutants? Performing a quantitative measurement of brain volume (as in Figure 6C) in LINT-O mutants upon piwi, vasa, aub, and nos knockdowns would be helpful analysis for comparing L(3)mbt and LINT-O mutants.*

We thank the reviewer for pointing these out. We immunostained fly ovaries with anti-Lint-O antibodies, but no signal was detected. The antibodies also failed to immunostain cultured OSCs, suggesting that they are not suitable for immunostaining. Therefore, the gene encoding Venus was knocked in at the *lint-O* genomic loci and Lint-O-Venus was expressed in the ovaries. Fluorescent signals were detected in both germ and somatic cells, suggesting that both cell types express Lint-O. The result was presented as Fig EV5F. The method was included in the revised text (page 35).

It is not yet known whether the ovarian phenotype is due to loss of Lint-O expression in germ, somatic, or both. In fact, we attempted to knock down Lint-O specifically in follicle cells in the ovary using the UAS-Lint-O shRNA line and the Tj-Gal4 line, but Lint-O knockdown (KD) flies could not survive to the adult stage after transfer to 29°C upon spawning at 25 ºC. Most of the Lint-O KD flies died at the larval stage. The Ruth Lehmann group previously reported in their paper (Coux *et al. Development* 2018) that "*somatic expression of L(3)mbt is important for ovarian morphological maintenance*." Therefore, it is speculated that the ovarian defective phenotype caused by the loss of

Lint-O is *due to the loss of "somatic" expression of Lint-O*. We discussed this in the revised text (page 19).

We are planning to quantitatively measure brain volume in *lint-O* mutants upon *piwi, vasa, aub,* or *nos* knockdown. However, it is very time consuming and beyond the scope of the manuscript, so will be reported separately in a future paper.

Minor points:

1) *In figure 3B, there is an increase in binding of the 8CA LINT-O mutant to L(3)mbt. Is this increase significant and can the authors discuss this further? Does complete loss of the PHD Finger domain also result in a similar increase in binding?*

We thank the reviewer for pointing this out. We generated the Lint-O mutant lacking the PHD finger domains (ΔPHD) and examined its binding activity to L(3)mbt. The ΔPHD mutant failed to bind to L(3)mbt (revised Fig 3H). It is known that the SAM domain serves as a protein-protein interaction domain and the PHD finger domain acts as chromatin binding. Based on these, we proposed the idea that the ability of Lint-O to associate with chromatin via the PHD domains influences the L(3)mbt–Lint-O interaction (page 13 in the revised text). We noticed that the 8CA mutation introduced in the PHD domains slightly but constantly strengthened the L(3)mbt–Lint-O interaction. We discussed this issue in the revised text (page 13).

2) *A reorganization of the text that relates to figure 3 would be helpful for clarity of the data. For example, it would be helpful if the authors introduce the 8CA mutant when they talk about the IP in figure 3B, rather than later in the text.*

We thank the reviewer for this suggestion. We modified the text related to Fig 3 accordingly (parts on pages 12-14 in the revised text). Figs 3 and EV3 (related to Fig 3) were reorganized upon addition of new data.

3) *When discussing expression of Aub or AGO3, is it possible that L(3)mbt has an indirect effect on their expression? Since these two genes were categorized in the "unbound" and "non-promoter mediated" groups, one possible hypothesis is that depletion of L(3)mbt activates a transcription factor or other regulator that may then impact AGO3/aub expression. This hypothesis should be discussed.*

We thank the reviewer for pointing this out. The possibility that depletion of L(3)mbt activates transcription factors and/or other regulators that impact *AGO3/aub* expression was discussed in the revised text (page 16).

Referee #2:

Major comments:

1) *In this manuscript the authors report the RNA-seq results from L(3)mbt knock-down (KD) cells (RNAi). However, the same group previously published a paper where they generated an L(3)mbt-deletion line in OSCs using CRISPR/Cas9 (PMID: 27474440). Why haven't authors used this L(3)mbt-deletion OSC line instead of RNAi experiments?*

The reason was that we wanted to compare RNA-seq of Lint-O-knockdown OSCs with that of L(3)mbt-knockdown OSCs. We do not yet have a Lint-O-deletion OSC line for comparison.

2) *Page 8: The authors say that "we further classified these genes according to where in each gene L(3)mbt is bound" and go on to group the genes into "promoter-mediated" and "non-promoted mediated" genes in Figure 1. It's misleading to call the genes as "promoter-mediated" simply because there is L(3)mbt ChIP-seq signal in the promoter region and the rest as "non-promoter mediated" just because the signal is not detected within the promoters especially given the fact that most of the L(3)mbt-promoter-bound genes do not change in expression upon L(3)mbt depletion. The terminology adds further confusion since genes termed as "non-promoter-mediated" genes could be promoter-mediated without L(3)mbt. In order to show that l(3)mbt is indeed performing a promoter-mediated gene regulation it is important to show this with experimental manipulation where the binding event is disturbed or with luciferase reporter assay and similar experiments. To avoid confusion, the authors should rephrase these terms to "promoter-region binding" and the "non-promoter genic binding" instead of saying "mediated" as it implies gene regulation. The "non-promoter genic binding" could be further subdivided into intronic and exonic or could be classified based on the distance to TSS (e.g., 1kb, 5kb to TSS etc).*

We thank the reviewer for pointing this out. To avoid unnecessary confusion, we rephrased *"promoter-mediated"* to "*promoter region binding*" and *"non-promoter-mediated"* to "*non-promoter genic binding*" throughout the revised manuscript. Also, we further subdivided protein coding genes based on the distance to most proximal TSS. The results were included as Appendix Fig S1B and C.

3) *Page 8: What fold-change values were used to classify upregulated, downregulated and unchanged genes? In the methods section the authors mention that FDR q-value of <0.01 was used, however the log2FC threshold for defining upregulated, downregulated and unchanged genes is not mentioned. It is also important to at least indicate the fold-change difference in gene expression for genes that are mentioned throughout the text (ago3, vasa, aub etc.). The authors should have these numbers from their edgeR analysis results. The differential gene expression results from edgeR should be included in the supplementary data or table.*

In RNA-seq analysis, genes were extracted only by the threshold of *q*-value, and if the fold change was greater than 0, the gene was categorized as "upregulated", and conversely, if the fold change was less than 0, the gene was categorized as "downregulated". Others were categorized as "unchanged". We originally indicated the fold-change difference in gene expression for *vasa, CG9925, tej, boot, qin, aub, AGO3*, and *piwi* (original Fig EV4C). Differential expressed genes were listed in Appendix Table S3.

4) *Page 8 and Methods: In order to show consistency between RNA-seq replicates it is important to run the principal component analysis (PCA) to show how replicates group together in wild-type OSCs and in l(3)mbt-KD OSCs instead of simply looking at FPKM correlations.*

We thank the reviewer for this suggestion. To show consistency between RNA-seq replicates, we run the principal component analysis (PCA). The results were indicated as revised Fig EV2B.

5) *Page 9: The authors mention that "L(3)mbt binds to a relatively wide range of genes, but for genes functioning in OSCs, there appears to be activators that override the repression by L(3)mbt." Yet, this still doesn't explain why there is almost as many promoter-L(3)mbt-bound genes that are down-regulated in response to L(3)mbt repletion (n=1044) as there are up-regulated genes (n=1202) if it indeed functions as a repressor. L(3)mbt was previously shown to also function as an insulator which could explain some of these effects (PMID: 21857667; PMID: 22722341). These could be secondary effects from L(3)mbt-induced repression of other genes.*

We thank the reviewer for this suggestion. The issue of insulator effect was discussed in the revised text (page 23) by citing the two papers.

6) *Page 10: The authors state "One plausible scenario is that, when L(3)mbt controls target genes via the promoter (e.g., vasa), it is almost independent of both LINT and dREAM complexes, but when L(3) controls target genes via regions other than the promoter (e.g., aub and AGO3), it may subtly depend on the LINT complex." -- Can the authors check the expression levels of the other promoter-L(3)mbt-bound genes such as tej, boot or qin in knock-down samples to test this hypothesis?*

We thank the reviewer for pointing this out. We believe we can do it. However, extending the scope to *tej*, *boot or qin* does not cover all relevant factors and thus does not get us out of the realm of prediction.

7) *As with L(3)mbt in Figure 1, the Lint-O terminology for binding regions in Figure 4A should be revised from "promoter-mediated" to "promoter-bound" and from "non-promoter mediated" to "non-promoter genic binding" to clarify what the terms actually mean and to avoid any confusion.*

The changes were made accordingly.

8) *How man ChIP-seq peaks are there for Lint-O ChIP-seq and how many of these are common with L(3)mbt ChIP-seq peaks and how many are unique? It would be helpful to have a heatmap to visualize all these regions and a Venn diagram to show peak overlaps.*

The heatmap was already shown in original Fig 4E. We included the Venn diagram to show ChIP peak overlaps (with numbers) (revised Fig EV4B).

9) *On page 15, the authors claim that "L(3)mbt and Lint-O may rarely repress target genes by binding to introns (e.g., ago3) or upstream regions far from the TSS (e.g., aub)." -- Yet, their results show that the majority of promoter-bound regions by Lint-O and/or L(3)mbt do not change in expression and almost as many are downregulated as upregulated. The repression function alone can be hardly justified in this case let alone the effect of repression at distance. Moreover, the "non-promoter" group usually has >4-fold more upregulated genes than the down-regulated genes for both Lint-O and L(3)mbt. This would rather support the opposite claim that the intronic binding is more likely to be repressive than promoter binding. Since L(3)mbt also functions as an insulator it would be hard to infer the gene regulatory mechanisms such as direct repression from these analyses without any additional experiments such as deletion of the L(3)mbt binding sites at the*

promoter and non-promoter regions followed by RT-qPCR or RNA-seq of the genes or using artificial constructs such as luciferase reporter assays.

We thank the reviewer for pointing this out. This study was not intended to compare "intronic binding" vs "promoter binding" to see which is more repressive. Therefore, we do not understand clearly why the referee stated "*This would rather support the opposite claim that the intronic binding is more likely to be repressive than promoter binding.*"

A similar question was raised by Referee1 as to whether L(3)mbt/Lint-O regulation is direct or indirect (see Referee1 Minor comment #3). As noted above, the possibility of "indirect" issue was noted in the revised text (page 16). Even if we have obtained some insight by performing a *luc* assay using the promoter of one gene (*e.g., vasa*), it remains to be unclear whether the same can be said for other genes, even in the same category, so the *luc* assay may be feasible but not relevant in this case.

10) *For Figure 4F it would be better to plot upregulated and downregulated genes separately and running GO analysis separately on these two groups instead of lumping all together into one "L(3)mbt/Lint-O-dependent" category. The upregulated genes might just belong to a different pathway than downregulated genes and combining them together could make GO associations weaker.*

We thank the reviewer for this suggestion. We plotted upregulated and downregulated genes separately and run GO analysis separately on these two groups. The Venn diagram of "upregulated genes" (original Fig 4F) showed that 816 genes were shared between L(3)mbr-dependent and Lint-O-dependent groups. However, as originally noted in the original text (page 16), GO analysis did not enrich significant terms. We performed similar analysis for downregulated genes. The Venn diagram shows that 439 genes were shared between L(3)mbr-dependent and Lint-O-dependent groups. The diagram was included as Appendix Fig S3F. Again, GO analysis did not enrich significant terms.

Minor comments:

a) *Page 7: An overview of the ChIP-seq reads mapped on the Drosophila genome in Fig EV2A is not presented in an informative way. Instead, the authors should give a total number of L(3)mbt peaks, a distance distribution of L(3)mbt peaks to gene promoters (e.g. promoter, intronic, exonic, distal intergenic) and plot reads with a heatmap to visualize the L(3)mbt ChIP-seq signals between gene promoters in the way they do in Figure 1E.*

We thank the reviewer for this comment. The total number of L(3)mbt peaks was given in Fig EV2A. Fig EV2A was retained because of the inclusion of “total number” and the comprehensive display of genome-wide signals. Distance distribution of L(3)mbt peaks from most proximal TSS was analyzed and the result was shown as Appendix Fig S1B. An attempt was made to analyze the signals between gene promoters. However, this proved difficult because the promoters of individual genes were not precisely identified.

b) *Page 6 end of introduction: Lint-O is not a co-suppressor of L(3)mbt. This could imply that Lint-O suppresses L(3)mbt. Modify to "Lint-O interacts with L(3)mbt to suppress specific genes"?*

Lint-1 was claimed as a L(3)mbt co-repressor, but it did not mean that Lint-1 suppresses L(3)mbt. Therefore, we left the original statement as it was.

c) *The data in Figures 1B, 1C and 1D is redundant and included in the data in Figure 4B, 4C, and 4D since both show the same L(3)mbt ChIP-seq and L(3)mbt-KD RNA-seq data tracks. This should be clearly stated, or figures 1 and 4 could be combined into one figure instead or Lint-O ChIP-seq data moved to supplementary.*

We thank the reviewer for pointing this out. In Fig 4B–D, L(3)mbt ChIP-seq and L(3)mbt-KD RNA-seq data tracks (Fig 1B–D) were re-displayed simply to show them along with Lint-O ChIP-seq and Lint-O-KD RNA-seq data tracks. This was now stated in the revised figure legends (page 47).

d) *In Figure EV4F the authors compare FPKM from RNA-seq data of their experiments to the FlyAtlas 2 FPKM data which is not a recommended approach. The FPKM is used for "in-sample normalization" and can only be used to compare genes within one sample while "across samples normalized" reads are obtained with methods such as edgeR and Deseq. The authors compared their FPKM to a different dataset from a different group which was analysed with different tools. For a consistent and proper analysis, the authors must download and process the raw RNA-seq data in the same way they processed their own data and run "across sample normalization" together with their own data using tools such as edgeR or Deseq and only then can compare the data across samples and across different datasets.*

We thank the reviewer for pointing this out. We believe that the referee meant the data in Fig EV4G but not EV4F. We reanalyzed the *nos* data as suggested (Appendix Fig S3E).

e) Why only the L(3)mbt-S isoform was used in the co-IP experiment in Figure 3D? Would the L(3)mbt-L isoform similarly co-immunoprecipitate with Lint-O from the OSC lysates?

We thank the reviewer for pointing this out. We found that L(3)mbt-L also co-immunoprecipitated with Lint-O from the OSC lysates. The results were included as revised Fig 3D and E. The L(3)mbt-S results were moved to revised Fig EV3C and D.

Additional comments:

Referee 1 brought up a good point about Lint-O and L(3)mbt interaction at genomic loci in the Major point 1. The authors' response to the Referee 1's Major point 1 says that they performed ChIP-PCR and found that the loss of Lint-O significantly weakened the binding activity of L(3)mbt to the vasa promoter. These ChIP-PCR results are important and should be reported in the revised manuscript.

We thank the reviewer for pointing this out. The result was included as revised Fig 3J.

In the manuscript Page 10 (also major comment 6) they mention that "when L(3)mbt controls target genes via the promoter (e.g., vasa), it is almost independent of both LINT and dREAM complexes". This seems to be at odds with the above-mentioned ChIP-PCR results where the say that loss of Lint-O significantly weakened the binding activity of L(3)mbt to vasa promoter (as they mention in response to Referee 1 major point 1). The authors should report their ChIP-PCR results in the revised manuscript and should be careful when discussing these results.

We thank the reviewer for pointing this out. The loss of LINT and dREAM complexes (*e.g.,* Lint-1, CoRest, Myb, Mip120, and Mip130) had only a minor effect on the *vasa* level compared to loss of L(3)mbt (original Fig 2B). Thus, our original statement was not contradicted.

With regard to their response to the major comment 9. In the manuscript page 15 the authors claim that "L(3)mbt and Lint-O may rarely repress target genes by binding to introns (e.g., ago3) or upstream regions far from the TSS (e.g., aub)." It's important to note that L(3)mbt and Lint-O binding alone is not enough to state that they are repressive as they may also function as insulators. The authors responded stating that the study was not intended to compare intronic binding to promoter binding. The authors should be careful in such interpretations if they don't have the supporting data or the supporting analysis to back it up.

We found this comment related to Major comment #5 of this referee. The issue of insulator effects was discussed in the revised text (page 23).

Regarding to their response to the minor comment e). They should report results from the co-IP experiment with the L(3)mbt-L isoform in the revised manuscript.

As noted above, the L(3)mbt-L results were included as revised Fig 3D and E. The L(3)mbt-S results were moved to revised Fig EV3C and D.

Dear Mikiko,

Thank you for the submission of your revised manuscript. We have now received the enclosed reports from the referees that were asked to assess it. Referee 2 still has a few minor suggestions that I would like you to incorporate before we can proceed with the official acceptance of your manuscript.

A few editorial requests will also need to be addressed:

- Please reduce the number of keywords to 5.

- Please update/correct the conflict of interest subheading to "Disclosure and competing interest statement"

- The APPENDIX table of content is missing page numbers, please add. Appendix Table S3 is uploaded twice, please correct.

- I attach to this email a related ms file with comments by our data editors. Please address all comments in the final ms.

I would like to suggest some changes to the title and abstract that needs to be written in present tense. Please let me know whether you agree with the following:

Lint-O cooperates with L(3)mbt in target gene suppression to maintain homeostasis in fly ovary and brain

Loss-of-function mutations in Drosophila lethal(3)malignant brain tumor [l(3)mbt] cause ectopic expression of germline genes and brain tumors. Loss of L(3)mbt function in ovarian somatic cells (OSCs) aberrantly activates germline-specific piRNA amplification and leads to infertility. However, the underlying mechanism remains unclear. Here, ChIP-seq for L(3)mbt in cultured OSCs and RNA-seq before and after L(3)mbt depletion shows that L(3)mbt genomic binding is not necessarily linked to gene regulation and that L(3)mbt controls piRNA genes in multiple ways. Lack of known L(3)mbt co-repressors, such as Lint-1, has little effect on the levels of piRNA amplifiers. Identification of L(3)mbt interactors in OSCs and subsequent analysis reveals CG2662 as a novel co-regulator of L(3)mbt, termed "L(3)mbt-interactor in OSCs" (Lint-O). Most of the L(3)mbt-bound piRNA amplifier genes are also bound by Lint-O in a similar fashion. Loss of Lint-O impacts the levels of piRNA amplifiers, similar to the lack of L(3)mbt. The lint-O-deficient flies exhibit female sterility and tumorous brains. Thus, L(3)mbt and its novel co-suppressor Lint-O cooperate in suppressing target genes to maintain homeostasis in the ovary and brain.

EMBO press papers are accompanied online by A) a short (1-2 sentences) summary of the findings and their significance, B) 2-3 bullet points highlighting key results and C) a synopsis image that is exactly 550 pixels wide and 200-600 pixels high (the height is variable). You can either show a model or key data in the synopsis image. Please note that text needs to be readable at the final size. Please send us this information along with the revised manuscript

I look forward to seeing a final version of your manuscript as soon as possible. Please use this link to submit your revision: https://embor.msubmit.net/cgi-bin/main.plex

Best,
Esther

Esther Schnapp, PhD
Senior Editor
EMBO reports

Referee #1:

The authors addressed all questions. This study identifies an important new component in l(3)mbt function as a regulator of cell fate and especially germline fate maintenance and will thus be interesting to a range of readers.

Referee #2:

The authors have adequately addressed the reviewer comments.
Some minor comments:
1. Clarify that Figure 3J is a CHIP-PCR experiment

2. Follow the correct gene nomenclature throughout the text. e.g. ago3 should not be capitalized while other gene names are in small caps

3. q-values are missing for fold-changes in Figure EV4C. Do all the fold-changes shown pass q-value threshold for significance? Maybe add q-values beside the bars

4. Throughout the text "piRNA gene" should be better renamed to "piRNA pathway gene" or "piRNA pathway factor" to not confuse with piRNA producing genomic loci

Referee #1:

The authors addressed all questions. This study identifies an important new component in l(3)mbt function as a regulator of cell fate and especially germline fate maintenance and will thus be interesting to a range of readers.

We thank this referee for the positive comments.

Referee #2:

The authors have adequately addressed the reviewer comments.

We thank this referee for the positive comment.

Some minor comments:

1. Clarify that Figure 3J is a CHIP-PCR experiment.

We clarified that Figure 3J is a ChIP-qPCR experiment (page 47).

2. Follow the correct gene nomenclature throughout the text. e.g. ago3 should not be capitalized while other gene names are in small caps.

AGO3 was changed to *ago3* throughout the manuscript.

3. q-values are missing for fold-changes in Figure EV4C. Do all the fold-changes shown pass q-value threshold for significance? Maybe add q-values beside the bars.

We added *q*-values in the legend of Figure EV4C (page 49). All the fold-changes shown passed *q*-value threshold for significance. Asterisks that mean "q-value < 0.05" were shown beside the bars.

4. Throughout the text "piRNA gene" should be better renamed to "piRNA pathway gene" or "piRNA pathway factor" to not confuse with piRNA producing genomic loci.

"piRNA gene" was renamed to "piRNA pathway gene" (or "piRNA biogenesis factor" depending on the context) throughout the text.

Prof. Mikiko Siomi
The University of Tokyo
Biological Sciences
2-1-16 Yayoi
Bunkyo-ku, Tokyo 113-0032
Japan

Dear Prof. Siomi,

I am very pleased to accept your manuscript for publication in the next available issue of EMBO reports. Thank you for your contribution to our journal.

At the end of this email I include important information about how to proceed. Please ensure that you take the time to read the information and complete and return the necessary forms to allow us to publish your manuscript as quickly as possible.

As part of the EMBO publication's Transparent Editorial Process, EMBO reports publishes online a Review Process File to accompany accepted manuscripts. As you are aware, this File will be published in conjunction with your paper and will include the referee reports, your point-by-point response and all pertinent correspondence relating to the manuscript.

If you do NOT want this File to be published, please inform the editorial office within 2 days, if you have not done so already, otherwise the File will be published by default [contact: emboreports@embo.org]. If you do opt out, the Review Process File link will point to the following statement: "No Review Process File is available with this article, as the authors have chosen not to make the review process public in this case."

Should you be planning a Press Release on your article, please get in contact with emboreports@wiley.com as early as possible, in order to coordinate publication and release dates.

Thank you again for your contribution to EMBO reports and congratulations on a successful publication. Please consider us again in the future for your most exciting work.

Yours sincerely,

Esther Schnapp, PhD
Senior Editor
EMBO reports

****

THINGS TO DO NOW:

Please note that you will be contacted by Wiley Author Services to complete licensing and payment information. The required 'Page Charges Authorization Form' is available here: https://www.embopress.org/pb-assets/embo-site/er_apc.pdf - please download and complete the form and return to embopressproduction@wiley.com

You will receive proofs by e-mail approximately 2-3 weeks after all relevant files have been sent to our Production Office; you should return your corrections within 2 days of receiving the proofs.

Please inform us if there is likely to be any difficulty in reaching you at the above address at that time. Failure to meet our deadlines may result in a delay of publication, or publication without your corrections.

All further communications concerning your paper should quote reference number EMBOR-2021-53813V6 and be addressed to emboreports@wiley.com.

Should you be planning a Press Release on your article, please get in contact with emboreports@wiley.com as early as possible, in order to coordinate publication and release dates.

EMBO Press Author Checklist

Corresponding Author Name: Mikiko C. Siomi, Kuniaki Saito, Soichiro Yamanaka	
Journal Submitted to: EMBO Reports	
Manuscript Number: EMBOR-2021-53813V4	

USEFUL LINKS FOR COMPLETING THIS FORM
The EMBO Journal - Author Guidelines
EMBO Reports - Author Guidelines
Molecular Systems Biology - Author Guidelines
EMBO Molecular Medicine - Author Guidelines

Reporting Checklist for Life Science Articles (updated January 2022)
This checklist is adapted from Materials Design Analysis Reporting (MDAR) Checklist for Authors. MDAR establishes a minimum set of requirements in transparent reporting in the life sciences (see Statement of Task: 10.31222/osf.io/9sm4x). Please follow the journal's guidelines in preparing your manuscript.
Please note that a copy of this checklist will be published alongside your article.

Abridged guidelines for figures
1. Data
The data shown in figures should satisfy the following conditions:
- the data were obtained and processed according to the field's best practice and are presented to reflect the results of the experiments in an accurate and unbiased manner.
- ideally, figure panels should include only measurements that are directly comparable to each other and obtained with the same assay.
- plots include clearly labeled error bars for independent experiments and sample sizes. Unless justified, error bars should not be shown for technical replicates.
- if n<5, the individual data points from each experiment should be plotted. Any statistical test employed should be justified.
- Source Data should be included to report the data underlying figures according to the guidelines set out in the authorship guidelines on Data Presentation.

2. Captions
Each figure caption should contain the following information, for each panel where they are relevant:
- a specification of the experimental system investigated (eg cell line, species name).
- the assay(s) and method(s) used to carry out the reported observations and measurements.
- an explicit mention of the biological and chemical entity(ies) that are being measured.
- an explicit mention of the biological and chemical entity(ies) that are altered/varied/perturbed in a controlled manner.
- the exact sample size (n) for each experimental group/condition, given as a number, not a range;
- a description of the sample collection allowing the reader to understand whether the samples represent technical or biological replicates (including how many animals, litters, cultures, etc.).
- a statement of how many times the experiment shown was independently replicated in the laboratory.
- definitions of statistical methods and measures:
  - common tests, such as t-test (please specify whether paired vs. unpaired), simple χ2 tests, Wilcoxon and Mann-Whitney tests, can be unambiguously identified by name only, but more complex techniques should be described in the methods section;
  - are tests one-sided or two-sided?
  - are there adjustments for multiple comparisons?
  - exact statistical test results, e.g., P values = x but not P values < x;
  - definition of 'center values' as median or average;
  - definition of error bars as s.d. or s.e.m.

Please complete ALL of the questions below.
Select "Not Applicable" only when the requested information is not relevant for your study.

Materials

Newly Created Materials	Information included in the manuscript?	In which section is the information available? (Reagents and Tools Table, Materials and Methods, Figures, Data Availability Section)
New materials and reagents need to be available; do any restrictions apply?	Yes	Materials and Methods. There are no restrictions.

Antibodies	Information included in the manuscript?	In which section is the information available? (Reagents and Tools Table, Materials and Methods, Figures, Data Availability Section)
For **antibodies** provide the following information:<br>- Commercial antibodies: RRID (if possible) or supplier name, catalogue number and or/clone number<br>- Non-commercial: RRID or citation	Yes	Materials and Methods

DNA and RNA sequences	Information included in the manuscript?	In which section is the information available? (Reagents and Tools Table, Materials and Methods, Figures, Data Availability Section)
**Short novel DNA or RNA including primers, probes**: provide the sequences.	Yes	Appendix Table S2

Cell materials	Information included in the manuscript?	In which section is the information available? (Reagents and Tools Table, Materials and Methods, Figures, Data Availability Section)
**Cell lines:** Provide species information, strain. Provide accession number in repository **OR** supplier name, catalog number, clone number, and/**OR** RRID.	Yes	Materials and Methods
**Primary cultures:** Provide species, strain, sex of origin, genetic modification status.	Not Applicable	
Report if the cell lines were recently **authenticated** (e.g., by STR profiling) and tested for mycoplasma contamination.	Not Applicable	

Experimental animals	Information included in the manuscript?	In which section is the information available? (Reagents and Tools Table, Materials and Methods, Figures, Data Availability Section)
**Laboratory animals or Model organisms:** Provide species, strain, sex, age, genetic modification status. Provide accession number in repository **OR** supplier name, catalog number, clone number, **OR** RRID.	Yes	Materials and Methods
**Animal observed in or captured from the field:** Provide species, sex, and age where possible.	Not Applicable	
Please detail **housing and husbandry conditions**.	Not Applicable	

Plants and microbes	Information included in the manuscript?	In which section is the information available? (Reagents and Tools Table, Materials and Methods, Figures, Data Availability Section)
**Plants:** provide species and strain, ecotype and cultivar where relevant, unique accession number if available, and source (including location for collected wild specimens).	Not Applicable	
**Microbes:** provide species and strain, unique accession number if available, and source.	Not Applicable	

Human research participants	Information included in the manuscript?	In which section is the information available? (Reagents and Tools Table, Materials and Methods, Figures, Data Availability Section)
If collected and within the bounds of privacy constraints report on age, sex and gender or ethnicity for all study participants.	Not Applicable	

Core facilities	Information included in the manuscript?	In which section is the information available? (Reagents and Tools Table, Materials and Methods, Figures, Data Availability Section)
If your work benefited from core facilities, was their service mentioned in the acknowledgments section?	Yes	Acknowledgements

Design

Study protocol	Information included in the manuscript?	In which section is the information available? (Reagents and Tools Table, Materials and Methods, Figures, Data Availability Section)
If study protocol has been **pre-registered, provide DOI in the manuscript**. For clinical trials, provide the trial registration number **OR** cite DOI.	Not Applicable	
Report the **clinical trial registration number** (at ClinicalTrials.gov or equivalent), where applicable.	Not Applicable	

Laboratory protocol	Information included in the manuscript?	In which section is the information available? (Reagents and Tools Table, Materials and Methods, Figures, Data Availability Section)
Provide DOI OR other citation details if **external detailed step-by-step protocols** are available.	Yes	Materials and Methods

Experimental study design and statistics	Information included in the manuscript?	In which section is the information available? (Reagents and Tools Table, Materials and Methods, Figures, Data Availability Section)
Include a statement about **sample size** estimate even if no statistical methods were used.	Yes	Results, Figures, Figure legends, Materials and Methods
Were any steps taken to minimize the effects of subjective bias when allocating animals/samples to treatment (e.g. **randomization procedure**)? If yes, have they been described?	Not Applicable	
Include a statement about **blinding** even if no blinding was done.	Not Applicable	
Describe **inclusion/exclusion criteria** if samples or animals were excluded from the analysis. Were the criteria pre-established?<br><br>If sample or data points were omitted from analysis, report if this was due to attrition or intentional exclusion and provide justification.	Not Applicable	
For every figure, are **statistical tests** justified as appropriate? Do the data meet the assumptions of the tests (e.g., normal distribution)? Describe any methods used to assess it. Is there an estimate of variation within each group of data? Is the variance similar between the groups that are being statistically compared?	Yes	Materials and Methods

Sample definition and in-laboratory replication	Information included in the manuscript?	In which section is the information available? (Reagents and Tools Table, Materials and Methods, Figures, Data Availability Section)
In the figure legends: state number of times the experiment was **replicated** in laboratory.	Yes	Figure legends
In the figure legends: define whether data describe **technical or biological replicates**.	Yes	Figure legends

Ethics

Ethics	Information included in the manuscript?	In which section is the information available? (Reagents and Tools Table, Materials and Methods, Figures, Data Availability Section)
Studies involving **human participants**: State details of **authority granting ethics approval** (IRB or equivalent committee(s), provide reference number for approval.	Not Applicable	
Studies involving **human participants**: Include a statement confirming that **informed consent** was obtained from all subjects and that the experiments conformed to the principles set out in the WMA Declaration of Helsinki and the Department of Health and Human Services Belmont Report.	Not Applicable	
Studies involving **human participants:** For publication of **patient photos**, include a statement confirming that consent to publish was obtained.	Not Applicable	
Studies involving experimental **animals**: State details of **authority granting ethics approval** (IRB or equivalent committee(s), provide reference number for approval. Include a statement of compliance with ethical regulations.	Yes	Materials and Methods
Studies involving **specimen and field samples:** State if relevant **permits** obtained, provide details of authority approving study; if none were required, explain why.	Not Applicable	

Dual Use Research of Concern (DURC)	Information included in the manuscript?	In which section is the information available? (Reagents and Tools Table, Materials and Methods, Figures, Data Availability Section)
Could your study fall under dual use research restrictions? Please check biosecurity documents and list of **select agents and toxins** (CDC): https://www.selectagents.gov/sat/list.htm	Not Applicable	
If you used a select agent, is the security level of the lab appropriate and reported in the manuscript?	Not Applicable	
If a study is subject to dual use research of concern regulations, is the name of the **authority granting approval and reference number** for the regulatory approval provided in the manuscript?	Not Applicable	

Reporting

The MDAR framework recommends adoption of discipline-specific guidelines, established and endorsed through community initiatives. Journals have their own policy about requiring specific guidelines and recommendations to complement MDAR.

Adherence to community standards	Information included in the manuscript?	In which section is the information available? (Reagents and Tools Table, Materials and Methods, Figures, Data Availability Section)
State if relevant guidelines or checklists (e.g., **ICMJE, MIBBI, ARRIVE, PRISMA**) have been followed or provided.	Not Applicable	
For **tumor marker prognostic studies**, we recommend that you follow the **REMARK** reporting guidelines (see link list at top right). See author guidelines, under 'Reporting Guidelines'. Please confirm you have followed these guidelines.	Not Applicable	
For **phase II and III randomized controlled trials**, please refer to the **CONSORT** flow diagram (see link list at top right) and submit the CONSORT checklist (see link list at top right) with your submission. See author guidelines, under 'Reporting Guidelines'. Please confirm you have submitted this list.	Not Applicable	

Data Availability

Data availability	Information included in the manuscript?	In which section is the information available? (Reagents and Tools Table, Materials and Methods, Figures, Data Availability Section)
Have **primary datasets** been deposited according to the journal's guidelines (see 'Data Deposition' section) and the respective accession numbers provided in the Data Availability Section?	Yes	Data Availability Section
Were **human clinical and genomic datasets** deposited in a public access-controlled repository in accordance to ethical obligations to the patients and to the applicable consent agreement?	Not Applicable	
Are **computational models** that are central and integral to a study available without restrictions in a machine-readable form? Were the relevant accession numbers or links provided?	Yes	Materials and Methods
If publicly available data were reused, provide the respective **data citations in the reference list**.	Not Applicable	
